# Novel metastatic models of esophageal adenocarcinoma derived from FLO-1 cells highlight the importance of E-cadherin in cancer metastasis

**DOI:** 10.18632/oncotarget.13391

**Published:** 2016-11-16

**Authors:** David S. Liu, Sanne J.M. Hoefnagel, Oliver M. Fisher, Kausilia K. Krishnadath, Karen G. Montgomery, Rita A. Busuttil, Andrew J. Colebatch, Matthew Read, Cuong P. Duong, Wayne A. Phillips, Nicholas J. Clemons

**Affiliations:** ^1^ Division of Cancer Research, Peter MacCallum Cancer Centre, Melbourne, Victoria, 3000, Australia; ^2^ Division of Cancer Surgery, Peter MacCallum Cancer Centre, Melbourne, Victoria, 3000, Australia; ^3^ Sir Peter MacCallum Department of Oncology, The University of Melbourne, Parkville, Victoria, 3010, Australia; ^4^ Laboratory for Experimental Oncology and Radiobiology, Center for Experimental and Molecular Medicine, Academic Medical Center, Amsterdam, 1105 AZ, The Netherlands; ^5^ Gastroesophageal Cancer Program, St Vincent's Centre for Applied Medical Research, Darlinghurst, New South Wales, 2010, Australia; ^6^ Department of Gastroenterology and Hepatology, Academic Medical Center, Amsterdam, 1105 AZ, The Netherlands; ^7^ The University of Melbourne Department of Medicine, Royal Melbourne Hospital, Parkville, Victoria, 3010, Australia; ^8^ University of Melbourne Department of Surgery, St Vincent's Hospital, Fitzroy, Victoria, 3065, Australia

**Keywords:** metastasis, esophageal cancer, CDH1, E-cadherin, animal models

## Abstract

There is currently a paucity of preclinical models available to study the metastatic process in esophageal cancer. Here we report FLO-1, and its isogenic derivative FLO-1^LM^, as two spontaneously metastatic cell line models of human esophageal adenocarcinoma. We show that FLO-1 has undergone epithelial-mesenchymal transition and metastasizes following subcutaneous injection in mice. FLO-1^LM^, derived from a FLO-1 liver metastasis, has markedly enhanced proliferative, clonogenic, anti-apoptotic, invasive, immune-tolerant and metastatic potential. Genome-wide RNAseq profiling revealed a significant enrichment of metastasis-related pathways in FLO-1^LM^ cells. Moreover, *CDH1*, which encodes the adhesion molecule E-cadherin, was the most significantly downregulated gene in FLO-1^LM^ compared to FLO-1. Consistent with this, repression of E-cadherin expression in FLO-1 cells resulted in increased metastatic activity. Importantly, reduced E-cadherin expression is commonly reported in esophageal adenocarcinoma and independently predicts poor patient survival. Collectively, these findings highlight the biological importance of E-cadherin activity in the pathogenesis of metastatic esophageal adenocarcinoma and validate the utility of FLO-1 parental and FLO-1^LM^ cells as preclinical models of metastasis in this disease.

## INTRODUCTION

Metastatic esophageal cancer has an exceedingly poor prognosis with a median survival of under 12 months [1]. Treatment options for these patients are limited and often ineffective [2]. The fact that over 70% of patients present with *de novo* metastatic disease or develop metastases after their diagnosis [3], highlights the need to improve our understanding of this pathological process in order to deliver better patient care.

Epithelial to mesenchymal transition (EMT) has been shown to play an important role in promoting metastasis in epithelium-derived carcinomas [4]. EMT involves changes at the genomic, epigenomic, transcriptomic and proteomic levels both intrinsic and extrinsic to the cancer cell [5]. These alterations affect signaling pathways that ultimately enable cancer cells to invade locally, traverse the systemic circulation and colonize distant sites [4]. In esophageal cancer, how these molecular events interact to promote metastasis remains poorly understood.

Metastatic models of esophageal cancer are scarce and difficult to establish. As a result, most investigators typically use *in vitro* assays only [6, 7]. Of those that are conducted in animals, intravenous or intracardiac injections are often used to seed cancer cells into distant organs [8, 9]. These methods however, fail to mimic the full metastatic process which occurs in patients and thus risk obscuring translatable insights into the biology of metastasis. Therefore, spontaneously metastatic models of human esophageal cancer would be extremely valuable for understanding the metastatic process.

To date, a limited number of spontaneously metastatic animal models of esophageal cancer have been reported [10–13]. These models however, pose several key challenges. Firstly, they involve surgery to the esophagus which may result in heavy bleeding, organ perforation, anastomotic leakage and death. Indeed, the reported postoperative mortality for Levrat's rodent surgical reflux model is at least 30% [13]. Secondly, the metastatic phenotype is not robust or reproducible, with the rate of metastasis varying between 0–78% across different studies [11, 13–16]. Thirdly, the duration from surgery or cancer cell inoculation to micro-metastasis is over 40 weeks in some models [13, 15]. These limitations therefore significantly hinder the use of these models for scientific discovery.

Models that develop timely and robust spontaneous metastasis without the need for invasive surgery would have significant preclinical utility. In this study, we show that FLO-1, a human esophageal adenocarcinoma (EAC) cell line, develops spontaneous metastasis following subcutaneous inoculation in mice. From this, we derived a highly metastatic and aggressive subline which, in combination with parental FLO-1, provides important insights into potential mechanisms underlying metastasis in esophageal cancer.

## RESULTS

### FLO-1 spontaneously metastasizes in NOD-SCID IL-2Rγ^KO^ (NSG) mice

**Figure 1:**
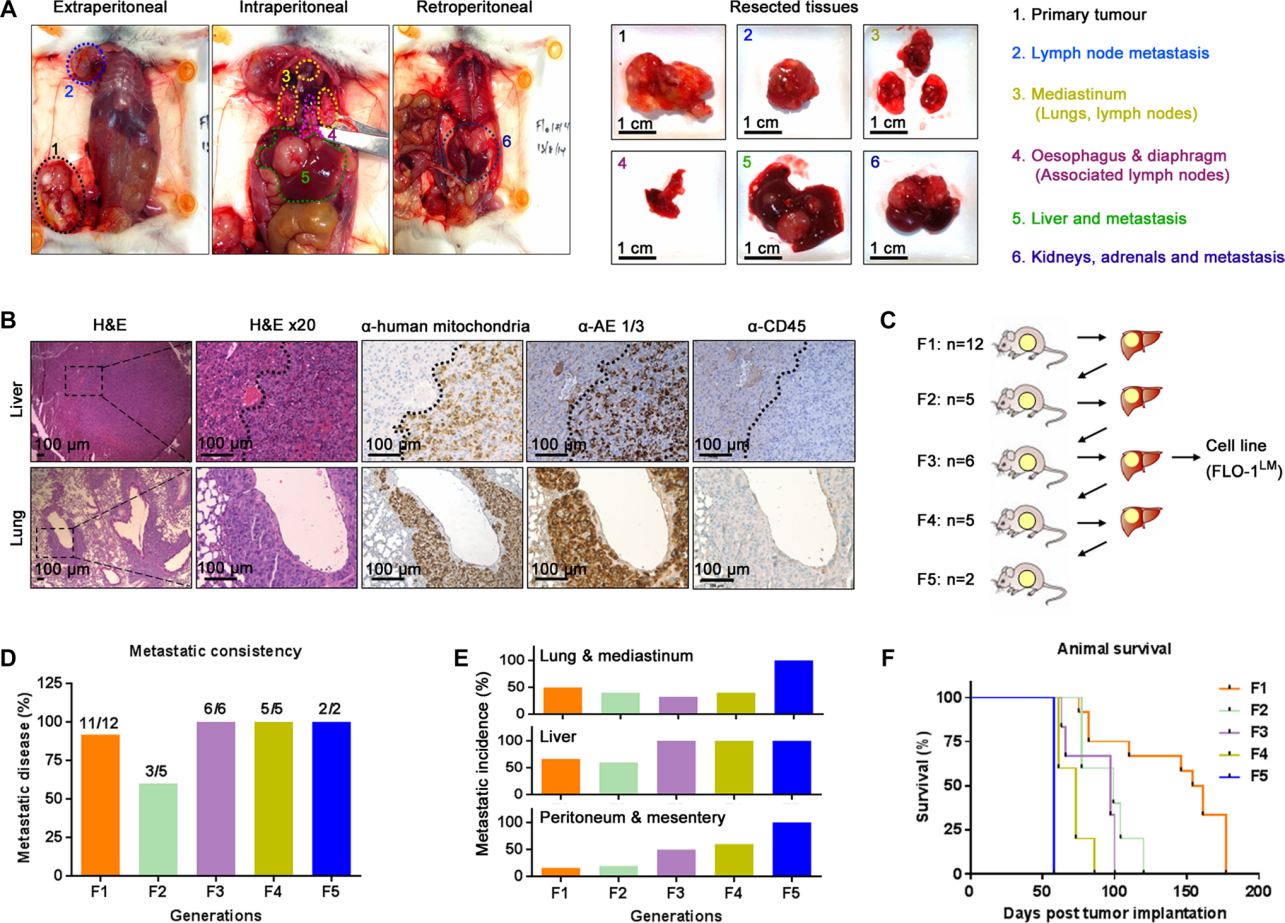
FLO-1 spontaneously metastasizes in NOD-SCID IL-2Rγ^KO^ (NSG) mice (**A**) Necropsy examination at ethical endpoint of an NSG mouse subcutaneously injected with 5 million FLO-1 cells. Widespread nodal and organ metastases demonstrated *in vivo* (Left) and *ex vivo* (Right). (**B**) Representative H&E and immunohistochemistry staining of the liver (Above) and lungs (Below) from mouse in (A). (**C**) Diagrammatic representation of passaging liver metastases across multiple generations of NSG mice. The number of recipient mice is shown per generation. (**D**–**E**) Frequency (D) and distribution (E) of metastasis over 5 generations of mice. (**F**) Survival curves of mice from each generation. See also Supplementary Figure S1.

Spontaneously metastatic models of human esophageal cancer are lacking. To address this area of need, we subcutaneously injected 8 human esophageal cancer cell lines into mice with different levels of immunocompetency to determine whether they are tumorigenic and spontaneously metastatic (Table 1). A cell line was deemed non-tumorigenic if the injection site remained tumor-free 6 months post injection. Once subcutaneous tumors reached endpoint volume, necropsy was performed on all animals to search for evidence of macro-metastasis. We found that all 8 cell lines were tumorigenic in NSG mice. However, depending on the cell line, tumorigenicity decreased with increasing host immunocompetency (Table 1). Notably, macro-metastases were only evident in NSG mice injected with the EAC cell line, FLO-1 (Figure 1A). The location of these metastases mirrored those seen in EAC patients, with tumors predominately present in the lung, liver, peritoneum and mediastinal lymph nodes (Figure 1A). Interestingly, we observed that the mammary artery ipsilateral to the subcutaneous tumor was consistently wider (Supplementary Figure S1A–S1B) and had more distributaries (Supplementary Figure S1C) than its contralateral counterpart. Furthermore, we also noted that metastases to the axillary lymph node, whilst relatively uncommon, always occurred ipsilateral to the subcutaneous tumor. These findings suggest that FLO-1 cells are able to metastasize via both lymphatic and haematological routes. To verify that these macro-metastases were indeed derived from FLO-1 cells, we demonstrated that tumors in the liver and lung stained positively for human mitochondria and pan-cytokeratin (Figure 1B). As NSG mice are at risk of developing *de novo* lymphomas [17], we also performed CD45 immunohistochemistry to exclude the possibility that these metastatic deposits were murine lymphoma in origin (Figure 1B). To enhance the metastatic phenotype of FLO-1, we subcutaneously passaged liver metastases over 5 consecutive generations in NSG mice (Figure 1C). We observed that with each successive generation the metastatic frequency increased, reaching 100% by the third passage (Figure 1D). Furthermore, the distribution of metastases became more homogenous with increasing passages (Figure 1E), and consequently, animals succumbed much earlier to their disease (Figure 1F).

**Table 1:** Tumorigenicity and metastatic potential of esophageal cancer cell lines

Cell line	Tumor of origin^a^						Xenograft		
	Histology	Location	Differentiation	Stage	CRT treatment	Mouse strain	Tumorigenic^b^	Metastatic^b^
FLO-1	EAC	Distal ^1^/_3_	Poor	pT2N1M0	No	NSG NOD-SCID SCID Nudes	12/12 5/5 0/5 0/5	11/12 0/5 0/5 0/5
Eso26	EAC	GEJ	Poor	pT4N1M1	No	NSG NOD-SCID SCID Nudes	3/3 ND ND 10/10	0/3 ND ND 0/10
OANC1	EAC	Distal^1^/_3_	Moderate	pT2N2M0	Yes	NSG NOD-SCID SCID Nudes	5/5 5/5 5/5 5/5	0/5 0/5 0/5 0/5
OE33	EAC	Distal^1^/_3_	Poor	pT3N0M0	No	NSG NOD-SCID SCID Nudes	5/5 5/5 5/5 5/5	0/5 0/5 0/5 0/5
JH-EsoAd1	EAC	Distal^1^/_3_	Moderate	pT3N0M0	No	NSG NOD-SCID SCID Nudes	10/10 5/5 0/5 0/5	0/10 0/5 0/5 0/5
OE19	EAC	GEJ	Moderate	pT3N1M0	No	NSG NOD-SCID SCID Nudes	3/3 5/5 5/5 10/10	0/3 0/5 0/5 0/10
TE7	ESCC	Mid ^1^/_3_	Poor	Not reported	No	NSG NOD-SCID SCID Nudes	10/10 15/15 5/5 0/5	0/10 0/15 0/5 0/5
OE21	ESCC	Mid ^1^/_3_	Moderate	pT3N0M0	No	NSG NOD-SCID SCID Nudes	5/5 0/5 0/5 0/5	0/5 0/5 0/5 0/5

a Tumor of origin data was retrieved from American Type Culture Collection, European Collection of Cell Cultures, Boonstra et al. [96, 97], Alvarez et al. [98], and Clemons et al. [93].

b For tumorigenicity and metastatic columns, numerator = outcome, denominator = total mice tested.

Esophageal adenocarcinoma (EAC), esophageal squamous cell carcinoma (ESCC), gastro-esophageal junction (GEJ), chemo-radiotherapy (CRT), non-obese diabetic (NOD), severe combined immunodeficiency (SCID), NOD-SCID IL-2Rγ^KO^ (NSG), not determined (ND).

### FLO-1 cells exhibit a mesenchymal phenotype

**Figure 2:**
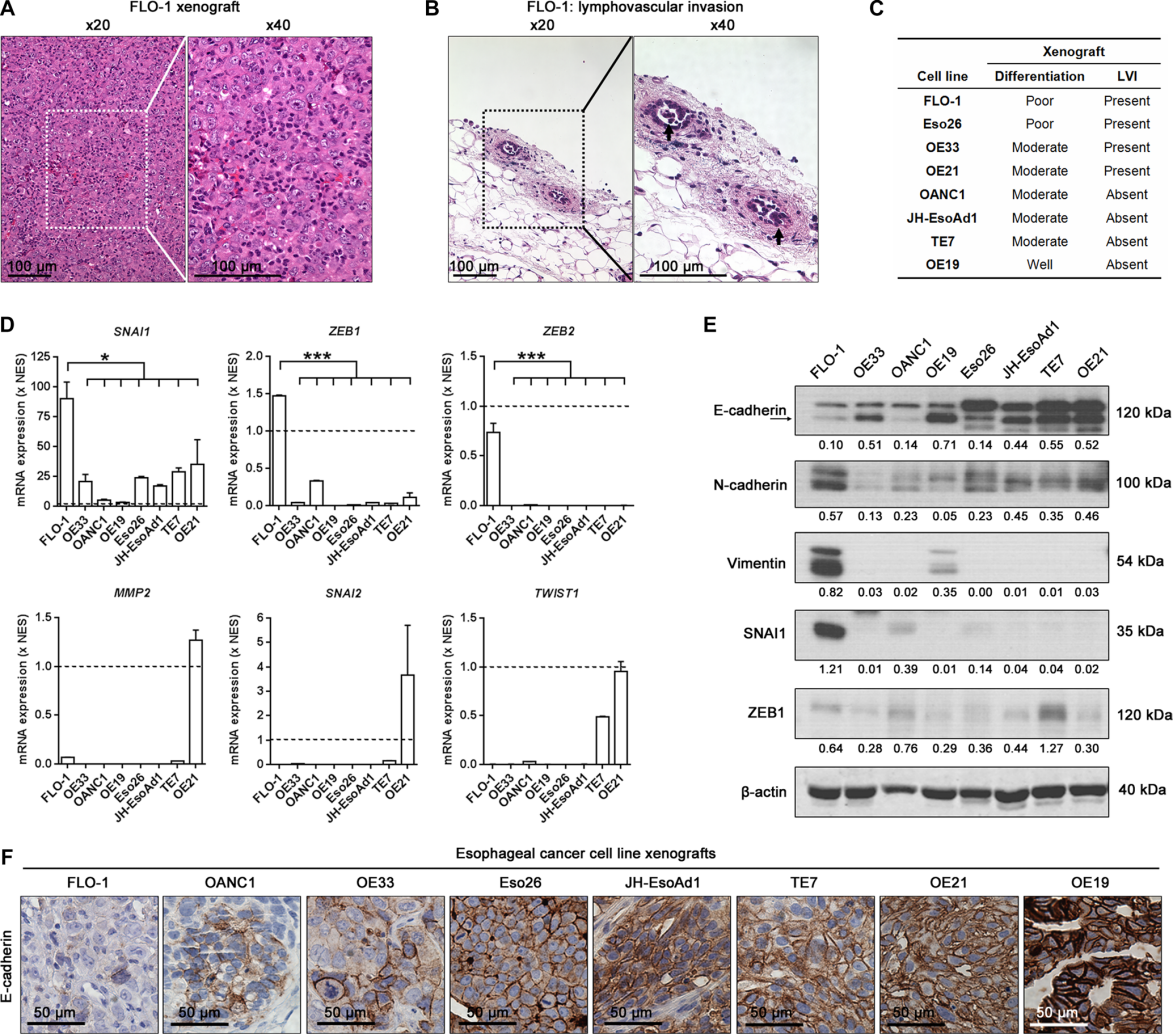
FLO-1 exhibits a mesenchymal phenotype (**A**–**B**) Representative H&E staining of primary FLO-1 xenografts demonstrating (A) poor tumor differentiation and (B) lymphovascular invasion (LVI, arrows). (**C**) Summary of tumor differentiation and LVI status for all cell line xenografts. (**D**) Comparison of endogenous mRNA expression of EMT genes across all cancer cell lines. mRNA expression for each cancer cell line is normalized to NES cells (Broken line), a normal esophageal squamous cell line. One-way ANOVA with Dunnett's multiple comparison posttest. Error bars = SEM, *n* = 2, *p < 0.05, ***p < 0.001. (**E**) Representative western blots of the indicated protein (Above) and their associated densitometric analysis (Below, normalized to β-actin) in all cancer cell lines. (**F**) Representative E-cadherin immunohistochemistry staining in all cancer cell line xenografts. See also Supplementary Figure S2.

To understand why FLO-1 is more metastatic than the other esophageal cancer cell lines, we examined H&E stained sections of all subcutaneous xenografts established in NSG mice (Supplementary Figure S2A). We noted that FLO-1 xenografts were poorly differentiated (Figure 2A) and had obvious evidence of lymphovascular invasion (Figure 2B–2C, Supplementary Figure S2B). Importantly, given that EMT may result in tumor de-differentiation, invasion and metastasis [4, 5], we next compared the mRNA and protein levels of well validated EMT genes across our cell line panel. We found that FLO-1 expressed significantly higher mRNA levels of the mesenchymal markers *SNAI1*, *ZEB1* and *ZEB2* compared to the other cancer cell lines (Figure 2D). Furthermore, the level of the mesenchymal proteins N-cadherin, Vimentin, SNAI1 and ZEB1 were elevated in FLO-1 cells (Figure 2E). In contrast, the expression of the key epithelial marker, E-cadherin, was lowest in FLO-1 cells and xenografts (Figure 2E–2F). Taken together, these findings suggest that FLO-1 cells have undergone EMT, and thus posses an inherent predisposition for metastasis.

### FLO-1^LM^: a highly metastatic and aggressive derivative of parental FLO-1

**Figure 3:**
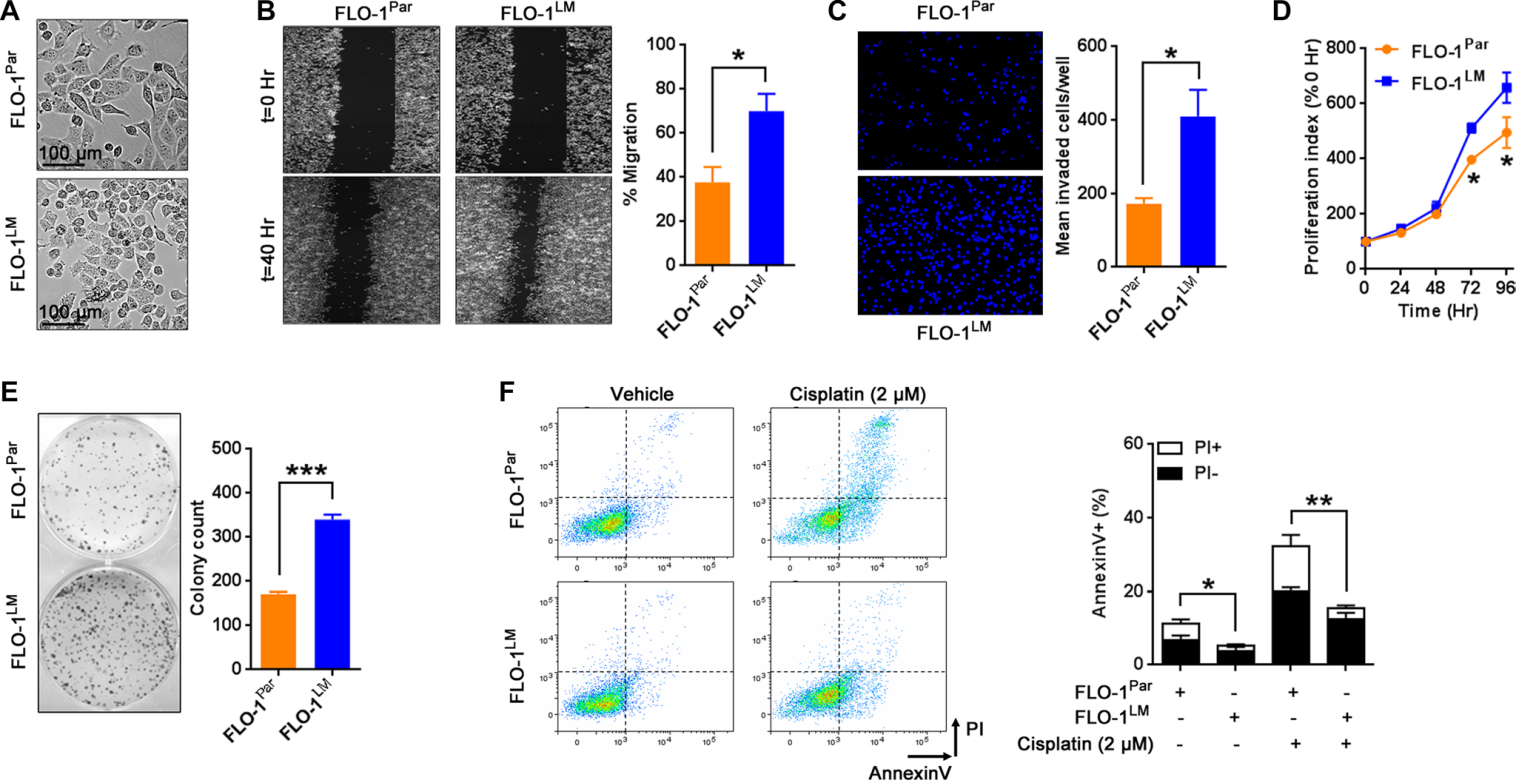
*In vitro* characterization of FLO-1^LM^ (**A**) Representative photomicrographs of parental FLO-1 (FLO-1^Par^) cells and its subline FLO-1^LM^. (**B**) Representative images comparing FLO-1^Par^ and FLO-1^LM^ cells in a monolayer migration assay captured immediately, and 40 hr post wounding (Left). This assay was performed under low serum (1%) conditions to minimize proliferation artifacts. The percentage of wound closure was quantified (Right). (**C)** Transwell invasion assay measured at 24 hr post cell seeding. Invaded cells were stained with DAPI (Left) and quantified (Right). (**D**) Proliferation assay comparing FLO-1^Par^ and FLO-1^LM^ cells. Cells were incubated with AlamarBlue reagent every 24 hr with the resultant fluorescence read and expressed as a percentage of 0 hr. (**E**) Clonogenic assay comparing FLO-1^Par^ and FLO-1^LM^ cells. Representative photograph (Left) and colony count (Right). (**F**) Representative FACS plots (Left) and quantification (Right) of AnnexinV/propidium iodide (PI) labeled FLO-1^Par^ and FLO-1^LM^ cells treated with 2 μM cisplatin. FACS analysis was carried out 48 hr post cisplatin. Unpaired *t*-test. Error bars = SEM, *p < 0.05, **p < 0.01, ***p < 0.001. *n* = 3 for all studies. See also Supplementary Table S1.

To generate a robust and highly metastatic model of EAC, we derived a cell line designated FLO-1^LM^ from a FLO-1 liver macro-metastasis following repeated passages through NSG mice (Figure 1C). Using short tandem repeat (STR) analysis, we firstly confirmed that FLO-1^LM^ is indeed derived from FLO-1 parental cells (Supplementary Table S1). In comparison to parental cells, we found that FLO-1^LM^ were smaller in size, and exhibited greater migratory, invasive, proliferative and clonogenic capacity *in vitro* (Figure 3A–3E). Additionally, we noted that FLO-1^LM^ were more resistant to apoptosis, both under basal growth conditions and following cisplatin treatment, than parental cells (Figure 3F).

**Figure 4:**
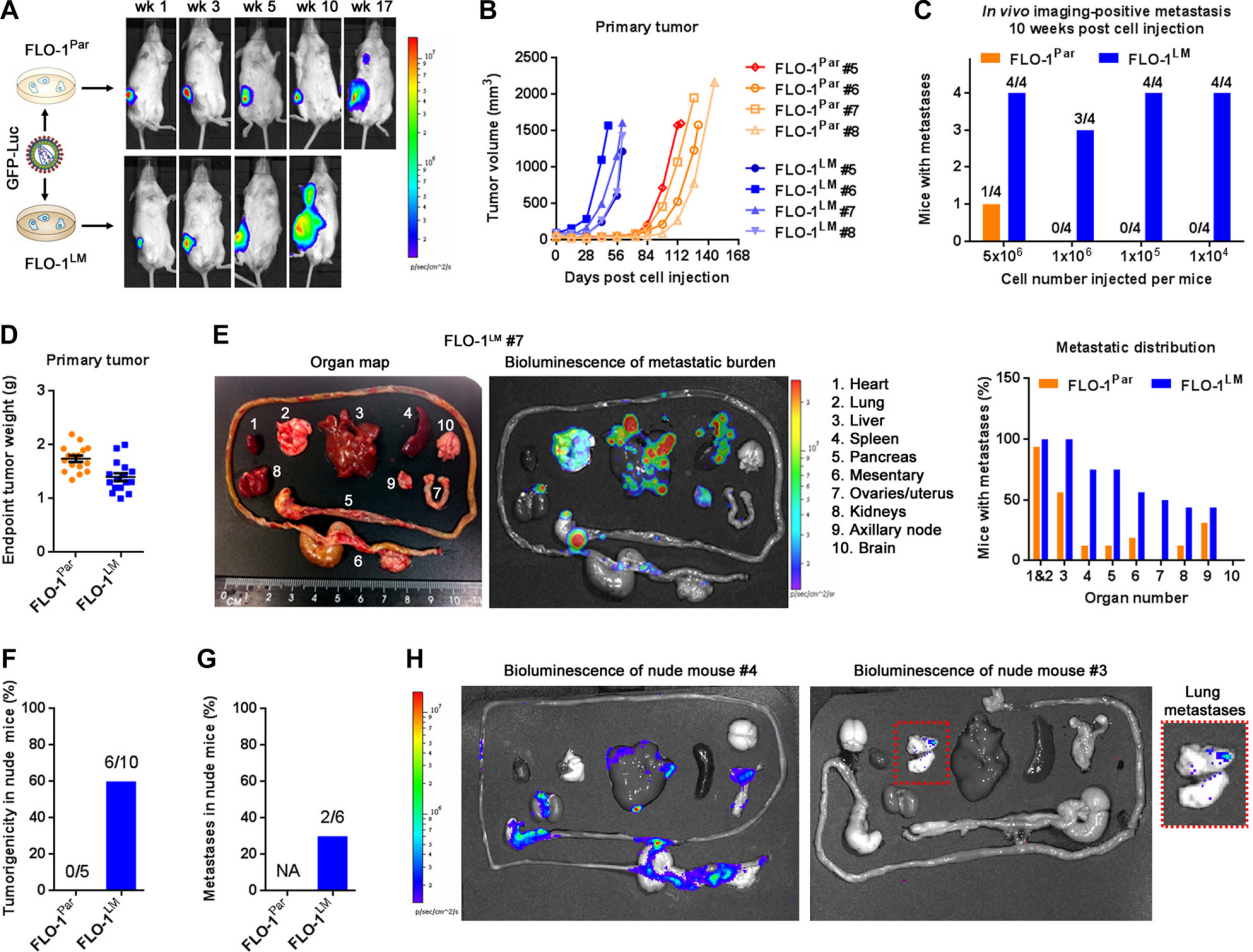
FLO-1^LM^ has increased proliferative and metastatic capacity *in vivo* (**A**) *Luciferase-eGFP* (GFP-Luc) transduced FLO-1^Par^ and FLO-1^LM^ cells were injected subcutaneously into NSG mice and imaged. Representative mice are shown. (**B**) Growth curves of FLO-1^Par^ and FLO-1^LM^ xenografts following subcutaneous injection of 1 million cells into the flank of NSG mice. (**C**) Incidence of image-positive metastasis 10 weeks post subcutaneous injection of different cell numbers into groups of NSG mice. 4 mice per cell line per group. (**D**) Weight of FLO-1^Par^ and FLO-1^LM^ subcutaneous xenografts from all mice in (C) at ethical endpoint. (**E**) Example of an anatomically annotated photograph of mouse organs (Left) paired with its bioluminescent heatmap (Middle). Analysis of metastatic burden at necropsy (Right) was pooled from all mice in (D). (**F**–**G**) Comparison of tumorigenic (F) and metastatic (G) potential between FLO-1^Par^ and FLO-1^LM^ cells in nude mice. (**H**) Bioluminescent heatmaps of organs from the two nude mice in (G) that developed metastases when injected subcutaneously with FLO-1^LM^ cells. Red box highlights multi-focal lung metastases. See also Supplementary Figure S3.

Since assessment of visible macro-metastases at necropsy may underestimate the extent of metastasis [18], we transduced FLO-1 parental and FLO-1^LM^ cells with a *luciferase-eGFP* reporter and compared their metastatic behavior *in vivo* in a limiting dilution assay using bioluminescence imaging (Figure 4A). We found that regardless of cell numbers injected into NSG mice, FLO-1^LM^ tumors grew faster (Figures 4A–4B, Supplementary Figure S3A–S3C), metastasized earlier (Figure 4C) and more widely (Figure 4D–4E, Supplementary Figure S3D) than FLO-1 parental tumors. Interestingly, we found that FLO-1^LM^ cells were also tumorigenic and metastatic in nude mice (Figure 4F–4H), whereas FLO-1 parental cells completely failed to grow in these mice with relatively higher immunocompetency.

### Transcriptomic analysis identifies *CDH1* as the most significantly downregulated gene in FLO-1^LM^

**Figure 5:**
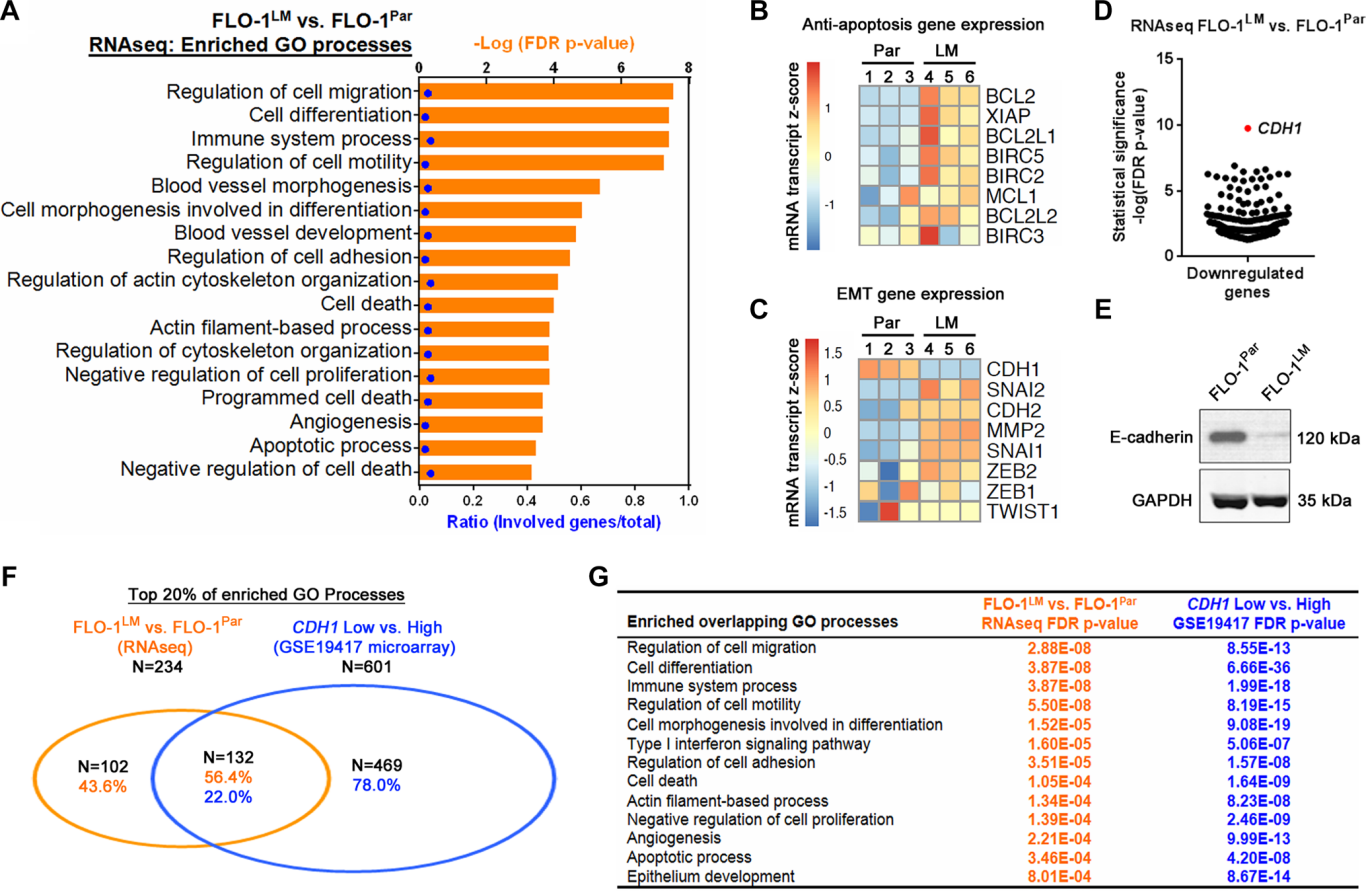
FLO-1^LM^ highlights molecular pathways that are deranged in metastasis (**A**) GO processes enriched from differentially expressed genes in FLO-1^LM^ compared with FLO-1^Par^ cells. Differentially expressed genes were identified using genome-wide RNAseq analysis. See also Supplementary Tables S2–S4. (**B**–**C**) Heatmap of anti-apoptotic (B) and EMT (C) genes expressed in FLO-1^LM^ compared with FLO-1^Par^ cells as determined using qRT-PCR. See also Supplementary Figure S4. (**D**) Scatter plot of all differentially downregulated genes (FDR *p*-value<0.05) in FLO-1^LM^ compared with FLO-1^Par^ cells as determined using RNAseq analysis. (**E**) Western blot comparing E-cadherin protein levels in FLO-1^LM^ vs. FLO-1^Par^ cells. (**F**) Venn diagram demonstrating the number and percentage of GO processes common to FLO-1^LM^ (vs. FLO-1^Par^, RNAseq analysis) and esophageal adenocarcinomas with low levels of *CDH1* expression (vs. *CDH1* high tumors, GSE19417 microarray). GO processes were determined based on differentially expressed genes in each dataset. (**G**) Examples of overlapping GO processes and their associated FDR *p*-values from analysis conducted in (F). See also Supplementary Tables S5–S6.

We next performed genome-wide RNA-sequencing (RNAseq) comparing FLO-1^LM^ and FLO-1 parental cells to gain molecular insights into the phenotypic differences between these two cell lines. Using an adjusted *p*-value < 0.05 as a cut-off, a total of 375 (307 downregulated, 68 upregulated) genes were differentially expressed in FLO-1^LM^ compared with parental cells (Supplementary Tables S2–S3). We found that the gene ontology (GO) terms most strongly associated with differentially expressed genes in FLO-1^LM^ were processes linked to cancer metastasis (Figure 5A, Supplementary Table S4). These included regulation of cell adhesion, migration, differentiation, cytoskeletal components, cellular proliferation, apoptosis, immune system processes and angiogenesis. To extend these findings, we performed qRT-PCR and western blot analysis and identified increased expression of multiple anti-apoptotic and EMT genes in FLO-1^LM^ compared with parental cells (Figure 5B–5C, Supplementary Figure S4A–S4D). Strikingly, on review of our RNAseq data, we found that *CDH1*, which encodes for E-cadherin, was the most significantly downregulated gene in FLO-1^LM^ (Figure 5D). This was subsequently validated using qRT-PCR and western blotting (Figure 5C and 5E).

To determine whether FLO-1^LM^ is a clinically relevant model, we next compared GO processes enriched in patient samples with those found in FLO-1^LM^. The GSE19417 Gene Expression Omnibus (GEO) microarray dataset was chosen for comparison, as it is one of the largest publicly available treatment-naive EAC cohorts with clinical annotation in humans [19]. We stratified this cohort based on *CDH1* expression, with low *CDH1* defined *a priori* as the bottom 25% (and high *CDH1* the top 75%). Significantly differentially expressed genes (Benjamini-Hochberg adjusted *p*-value < 0.05) between *CDH1* high and low tumors were identified (Supplementary Table S5) and the derived list subjected to GO enrichment analysis. Top enriched GO processes found in FLO-1^LM^ (vs. FLO-1 parental) and *CDH1* low tumors (vs. *CDH1* high tumours) were subsequently compared. Of 234 GO processes (top 20%) enriched in FLO-1^LM^, 132 (56.4%) overlapped with those found in the patient derived samples of the GEO dataset (Figure 5F, Supplementary Table S6), and many of these have immediate biological relevance to EMT, cancer invasion and metastasis (Figure 5G). Taken together, these results demonstrate that we have derived a clinically relevant isogenic subline of FLO-1 with heightened mesenchymal characteristics that is more aggressive, less immunogenic, and highly metastatic in nature.

### Low E-cadherin expression is associated with increased FLO-1 metastasis and reduced patient survival

**Figure 6:**
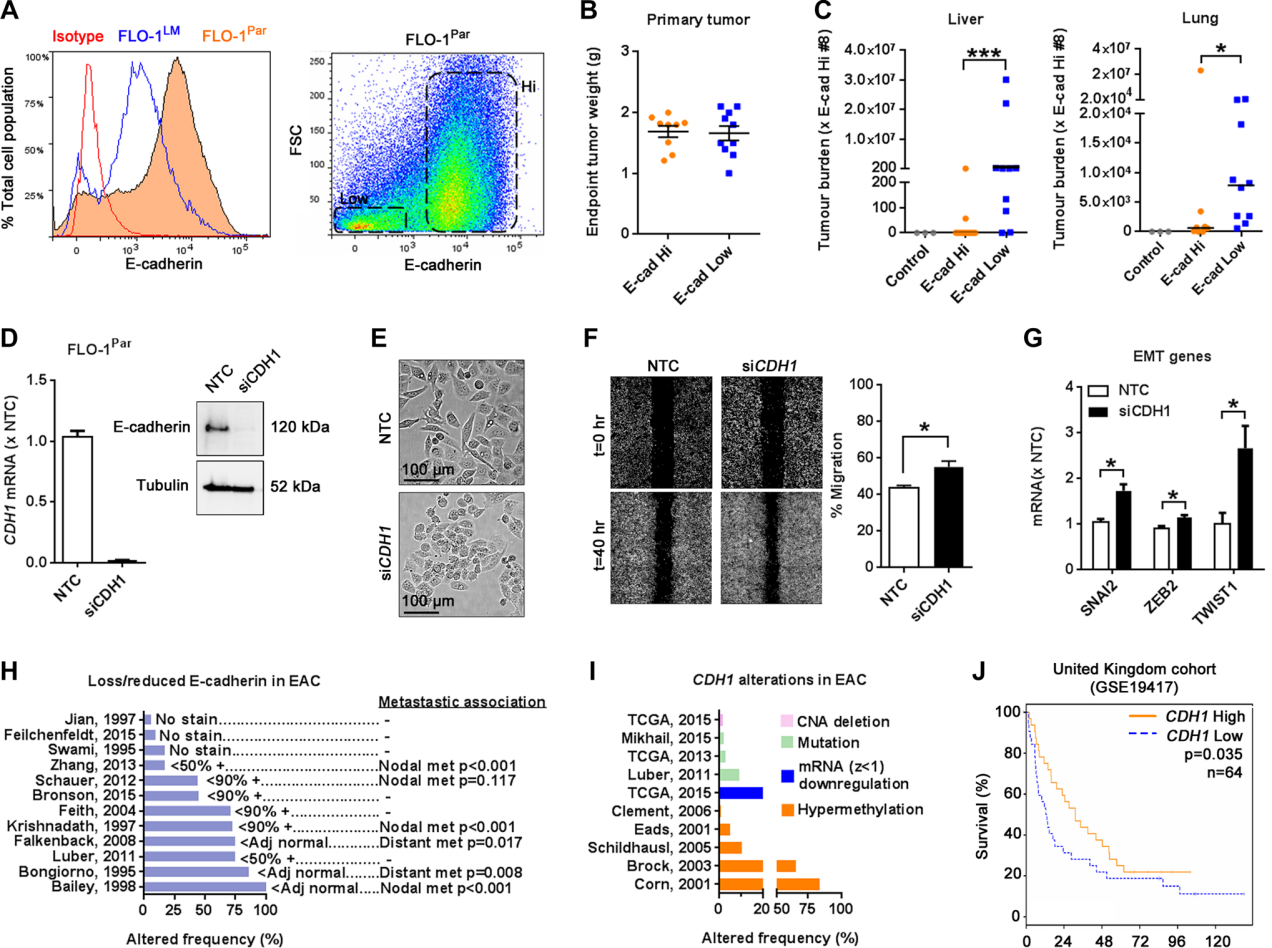
Low E-cadherin expression is associated with increased metastasis in FLO-1 and reduced patient survival (**A**) Histogram comparing E-cadherin expression in FLO-1^Par^ and FLO-1^LM^ cells using FACS analysis (Left). Scatter plot demonstrating subpopulations of FLO-1^Par^ cells with high and low E-cadherin expression (Right). (**B**–**C**) High and low E-cadherin expressing FLO-1^Par^ cells were isolated by FACS and subcutaneously injected in equal numbers into NSG mice. Subcutaneous tumors were weighed at ethical endpoint (B). Metastatic burden in the liver (C, Left) and lungs (C, Right) from these mice was analyzed using a TaqMan qPCR assay. All qPCR results were normalized against mouse #8. E-cadherin Hi: *n* = 9, E-cadherin Low: *n* = 10. Liver and lung tissues from 3 non-tumor bearing mice were used as controls. Bar = median. (**D**) Assessment of *CDH1* knockdown in FLO-1^Par^ cells with qRT-PCR (Left) and western blot (Right). Knockdown was mediated with si*CDH1*. A Non-targeting control (NTC) siRNA was also used. RNA and protein were harvested at 24 and 72 hr post siRNA transfection, respectively. (**E**) Representative photomicrographs of FLO-1^Par^ cells captured at 72 hr post siRNA transfection. (**F**) Representative images comparing FLO-1^Par^ cells transfected with si*CDH1* or NTC siRNA in a monolayer migration assay captured immediately, and 40 hr post wounding (Left). This assay was performed under low serum (1%) conditions to minimize proliferation artifacts. The percentage of wound closure was quantified (Right). (**G**) qRT-PCR analysis of selected EMT genes in FLO-1^Par^ cells 24 hr post siRNA transfection. (**H**) Frequency of lost/reduced E-cadherin expression in esophageal adenocarcinoma (EAC) from published studies, and its association with lymph node and distant organ metastasis. (−) Not reported. The annotations immediately adjacent to each bar indicate the study's definition of loss/reduced E-cadherin expression. (**I**) Frequency of *CDH1* genetic alterations in EAC in TCGA datasets and published studies. (**J**) Survival of patients with EAC stratified by *CDH1* expression as determined from microarray dataset (United Kingdom: GSE19417, low *CDH1* = bottom 25%). Mann-Whitney U test for C. Unpaired *t*-test for F and G. Kaplan-Meier Log-rank and multivariate Cox regression analysis for J. Error bars = SEM. *n* = 3 for all *in vitro* studies. *p < 0.05, ***p < 0.001. See also Supplementary Table S7 and Supplementary Figure S5.

Given that E-cadherin is a key inhibitor of EMT in other tumor types [4], we next examined the role of E-cadherin in metastasis using our FLO-1 model. Based on our finding that E-cadherin was significantly reduced in FLO-1^LM^ relative to parental cells (Figures 5E and 6A), we hypothesized that FLO-1 cells with low E-cadherin expression are intrinsically more metastatic than E-cadherin high expressers. To address this, we isolated subpopulations of FLO-1 parental cells with high and low E-cadherin levels (Figure 6A) and injected them separately into NSG mice. Using a TaqMan qPCR assay which quantifies the proportion of human versus mouse vimentin DNA in an organ, we analyzed the metastatic burden in the liver and lungs from these animals once their subcutaneous primary tumors reached the same endpoint weight (Figure 6B). Strikingly, we found that metastatic burden from cells with low E-cadherin expression was on average 160,000-fold greater in the liver and 12-fold greater in the lungs than high E-cadherin expressing cells (Figure 6C). Extending these findings, we found that E-cadherin knockdown in FLO1-parental cells (Figure 6D) resulted in smaller cells, enhanced migration, and increased the expression of *SNAI2*, *ZEB2* and *TWIST1 in vitro* (Figure 6E–6G, Supplementary Figure S5A), effectively phenocopying FLO-1^LM^ cells.

To investigate the clinical importance of *CDH1*/E-cadherin dysregulation in esophageal cancer, we firstly analyzed TCGA datasets (publicly available at cBioportal for Cancer Genomics: www.cbioportal.org) and reviewed the literature to determine the extent of *CDH1*/E-cadherin aberration in esophageal EAC. Across multiple studies, we found that E-cadherin levels were commonly lost, reduced or heterogeneously expressed in EAC (Figure 6H) [20–31]. This is in part explained by the frequent hyper-methylation of the *CDH1* promoter resulting in impaired gene transcription (Figure 6I) [32–36]. Additionally, genomic abnormalities such as mutations and deletions, albeit uncommon, have also been reported (Figure 6I) [28, 37]. Interestingly, these findings in EAC were also evident in esophageal squamous cell carcinoma (Supplementary Figure S5B–S5C) [38–62]. Finally, we correlated *CDH1* expression with patient survival using the GSE19417 dataset whilst simultaneously controlling for clinicopathological variables (Supplementary Table S7). We found that low *CDH1* expression was independently associated with significantly worse overall survival (Figure 6J). In keeping with this, multiple studies have reported that low E-cadherin expression in esophageal cancer is significantly associated with increased lymph node and distant organ metastasis (Figure 6H, Supplementary Figure S5B). Collectively, these findings illustrate the biological importance of *CDH1* expression in esophageal cancer metastasis, and further validate the utility of FLO-1 parental and FLO-1^LM^ cells as preclinical models of metastasis.

## DISCUSSION

There is a profound need for preclinical metastatic models of esophageal cancer. Here, we report the identification of FLO-1, and derivation of FLO-1^LM^, as two complementary spontaneously metastatic cell line models of human EAC in NSG mice. Using a combination of genomic, transcriptomic, proteomic and histopathological approaches, we have characterized these two cell lines and demonstrated that they recapitulate key aspects of metastatic disease in patients. These include the presence of lymphovascular invasion and high histological grade in primary subcutaneous xenografts. Additionally, the distribution of metastases seen *in vivo*, with a predilection for the liver, lungs and mediastinal lymph nodes, are in line with what is commonly observed in patients [63]. In support of this, metastasis-related pathways active in FLO-1^LM^ were also enriched in EAC samples. Furthermore, since parental FLO-1 cells were originally established from a treatment-naive patient (Table 1), these pathway alterations are likely to reflect innate tumor biology absent of therapy-related selection pressures. Collectively, these findings strongly support the utility of FLO-1 and FLO-1^LM^ as representative preclinical models of metastasis.

From an experimental standpoint, FLO-1 and FLO-1^LM^ overcome several key challenges inherent in existing metastatic models of esophageal cancer that have significantly limited their use. Firstly, FLO-1 and FLO-1^LM^ cells metastasize in a timely manner, approximately 12 and 6 weeks after subcutaneous injection of tumor cells respectively. In comparison, metastases only occur after 40 weeks post surgery in Levrat's surgical reflux model [13] or with EC9706 cells [15]. Secondly, the metastatic frequency of both FLO-1 parental (92%) and FLO-1 ^LM^ (100%) lines are much higher than those reported for PT1590 cells (78%), OE19 cells (70%), EC9706 cells (50%) and Levrat's reflux model (0–40%) [11, 13–16]. Consistent with this, in our hands, OE19 cells failed to metastasize in all animals despite robust primary tumor growth. Thirdly, spontaneous metastasis from a subcutaneous site eliminates the need for invasive surgery to generate such models [10, 13, 64]. This would minimize procedural related morbidity and mortality [13] and increase experimental reproducibility of the model.

Interestingly, parental FLO-1 cells were metastatic in NSG mice but not in NOD-SCID mice. The main difference between these two transgenic strains is the knockout of IL-2 receptor gamma-chain in NSG mice [65], which results in loss of cytokine signalling and impaired T, B, and natural killer cell function [66]. This likely facilitates metastasis of FLO-1 cells in NSG mice. Consistently, other studies have also reported that engraftment rates of human-derived tissues and cells are higher in NSG mice than in any other immuno-deficient mouse models [67, 68].

A key strength of our study is the establishment of FLO-1^LM^, which in contrast to parental FLO-1, is significantly more invasive *in vitro* and more metastatic *in vivo*. Therefore, these two models enable further understanding and investigation of mechanisms involved in cancer invasion and metastasis. To harness this aspect, we conducted a genome-wide comparison of both cell lines using RNAseq and gene ontology enrichment analysis. Consistently, the most differentially altered pathways between FLO-1^LM^ and parental FLO-1 were processes involved in regulating cell adhesion, migration, differentiation, proliferation, cytoskeletal organization, angiogenesis and apoptosis. Importantly, *CDH1* was identified as the most significantly downregulated gene in FLO-1^LM^.

The tumor suppressor gene *CDH1* encodes the cell adhesion molecule E-cadherin [69]. E-cadherin is crucial for the establishment and maintenance of epithelial cell polarity, mediating intercellular adhesion, regulating cytoskeletal organization and preventing EMT [69]. Therefore, E-cadherin expression is a hallmark of epithelial differentiation, and its repression is associated with tumorigenesis, invasion and metastasis in many tumor types [69]. Whilst numerous retrospective clinical studies of EAC have correlated low *CDH1* or E-cadherin expression with metastatic disease and poor survival [70], our study is the first to provide functional *in vivo* evidence of this process. Here, we showed that FLO-1 parental cells with low levels of E-cadherin expression have increased metastatic capacity compared with E-cadherin high expressers. Additionally, E-cadherin knockdown in FLO-1 parental cells induced a mesenchymal-like cell morphology, increased cell migration and upregulated EMT genes.

Reduced E-cadherin expression is common in EAC and typically occurs early during carcinogenesis [71]. The cause of reduced E-cadherin expression is unclear. It is unlikely however, that genomic aberrations alone will explain this phenomenon, as the incidence of *CDH1* mutation is relatively low in EAC. Whilst frequent promoter hypermethylation have been reported [35, 36], these results are not consistent across all studies [32, 33]. Therefore, it is likely that multiple factors are at play to suppress *CDH1* expression. Indeed, SNAI1, ZEB1 and ZEB2, well established transcriptional repressors of *CDH1* [4] are overexpressed in parental FLO-1. Their expression is further elevated in FLO-1^LM^, which concordantly has lower *CDH1* levels. Furthermore, as reported in other tumors types, upregulation of *DAB2* [72], *RND3* [73, 74], *TMEM97* [75], *CSE1L* [76], *MYO1C* [77], and *PTPRK* [78] have been shown to suppress or functionally redistribute E-cadherin expression in cancer cells, resulting in EMT, increased invasion, advanced tumor stage and poor patient survival. Consistently, our RNAseq analysis demonstrates that these genes are all significantly upregulated in FLO-1^LM^. Thus, these findings support our hypothesis that multiple factors conspire to repress E-cadherin expression in EAC.

Although E-cadherin low expressing FLO-1 cells were significantly more metastatic than E-cadherin high expressers, we observed that FLO-1 cells with high E-cadherin levels were still able to metastasize. It is likely that other factors may also promote metastasis. Consistent with this, our RNAseq analysis have also identified significant upregulation of *TRIP6*, *RAP2A* and *CCL2* in FLO-1^LM^. Elevated expression of these genes has been functionally shown to promote key hallmarks of metastasis in different cancers independent of E-cadherin [79–91]. Uniquely, our isogenic FLO-1 and FLO-1^LM^ cell lines thus enable identification and functional interrogation of these genes and other pathways underlying esophageal cancer metastasis.

Beyond highlighting mechanisms of metastasis, our study of FLO-1 and FLO-1^LM^ also suggest that the metastatic process may endow or select for cells with additional attributes. These include increased proliferative, clonogenic, anti-apoptotic and immune-tolerant potential. These findings provide further insights into why metastatic esophageal cancer is often refractory to conventional treatment. Therapeutic strategies designed to inhibit metastatic processes may thus be of important clinical value [92]. In this way, FLO-1 and FLO-1^LM^ are ideal platforms for testing novel anti-metastatic agents.

In summary, we have identified and characterized two spontaneously metastatic cell line models of human esophageal cancer. These models suggest that E-cadherin expression is a key inhibitor of metastasis in this disease, and also implicates a complex interplay of many other factors that may modulate this process. We anticipate that these two cell lines will shed new light into the pathogenesis and treatment of metastatic disease in EAC.

## MATERIALS AND METHODS

### Cell lines and culture

OE19, OE33 and HEK-293T cells were purchased from the American Type Culture Collection. FLO-1, Eso26, TE7 and OE21 cells were gifts from Rebecca Fitzgerald (University of Cambridge, UK). JH-EsoAd1 cells were provided by James Eshleman (John Hopkins University, MD). OANC1 cells were established in our laboratory [93], and immortalized human esophageal epithelial cells (NES) were a gift from Rhonda Souza (University of Texas Southwestern Medical Centre, TX). All cells were maintained at 37°C with 5% CO_2_. Unless otherwise stated, all culture media contained 50 U/ml penicillin, 50 mg/ml streptomycin (Life Technologies) and 10% fetal bovine serum (FBS). HEK-293T, FLO-1 and OANC1 cells were grown in Dulbecco's Modified Eagle Medium (DMEM) containing 2.5 mmol/L L-glutamine and 4.5 g/L D-glucose (Life Technologies). Eso26, OE33, JH-EsoAd1, OE19, TE7 and OE21 cells were cultured in Roswell Park Memorial Institute (RPMI) 1640 medium containing 2.5 mmol/L L-glutamine (Life Technologies). NES cells were maintained in modified MCDB-153 medium as previously reported [94]. All cell lines were authenticated by STR analysis using the PowerPlex^®^ 16 genotyping system (Promega) and confirmed mycoplasma free by PCR (Cerberus Sciences, Australia).

### Mice

All animal experiments were performed in accordance with the National Health and Medical Research Council Australian Code of Practice for the Care and Use of Animals for Scientific Purposes and approved by the Peter MacCallum Cancer Centre (PMCC) Animal Experimentation Ethics Committee. Female BALB/c nu/nu (Nude), SCID and NOD-SCID mice were obtained from the Animal Resource Centre (Western Australia). NOD-SCID IL-2Rγ^KO^ (NSG) mice were bred in-house.

### Tumor xenografts

To assess tumorigenicity and metastatic potential of esophageal cancer cell lines, 5 million cells suspended in 100 μl of 1:1 phosphate buffered saline (PBS) and matrigel (BD Bioscience) were subcutaneously injected into the flank of female Nude, SCID, NOD-SCID or NSG mice. FLO-1 metastatic deposits from the liver, approximately 2 mm in size, were implanted subcutaneously into NSG mice. Subcutaneous tumor volume was determined weekly with calipers and calculated using the formula (length × width^2^)/2. All mice were euthanised when subcutaneous tumors reached ≥ 1500 mm^3^ or at first signs of ill health (labored breathing, bloated abdomen or excessive weight loss: > 10% of baseline body weight).

### Histology and immunohistochemistry

H&E staining and immunohistochemistry was performed on formalin-fixed paraffin embedded tissues as previously described [93]. Antibodies against the following proteins were used: human mitochondrial antigen (MAB1273, Merck Millipore), AE1/AE3 (Leica Biosystems), CD45 (2B11, Dako) and E-cadherin (EP700Y, Abcam). Stained sections were viewed on a BX51 microscope (Olympus).

### Cancer cell line isolation and establishment

Macro-metastatic deposits in the liver were dissected away from the surrounding parenchyma and washed in PBS containing 12 μg/ml penicillin and 50 mg/ml streptomycin. Tumor pieces were finely chopped and incubated in Hank's Balance Salt Solution (Thermo Fisher Scientific) containing 6 mg/ml dispase II (Roche) and 3 mg/ml collagenase A (Roche) at 37°C on an orbital shaker for 2 hr. The resultant cell suspension was pelleted by centrifugation (1400 rpm, 4 min) and cultured in DMEM. Cells were passaged using 0.25% trypsin and verified using STR analysis. Formal experimentation with these cells began after 10 passages in culture.

### RNA-sequencing (RNAseq)

Cells were harvested at 80% confluency, 24 hr after a media change. Total RNA was extracted using the Qiagen RNeasy Kit. RNA quality was confirmed using a Fragment Analyzer^TM^ (Advanced Analytical Technologies). cDNA library preparation was performed using the NEBNext Ultra directional RNA library Prep Kit (Illumina). DNA sequencing was conducted on the Illumina NextSeq 500 system. Image analysis, base calling and quality checks were undertaken with the Illumina data analysis pipeline RTA v2.4.11 and Bcl2fastq v2.17. Read data was mapped to the reference sequence Homo_sapiens.GRCh37.75 using a short read aligner after trimming for adapter sequences with Trimmomatic v0.30. A default mismatch rate of 2% was used. Read counts per gene were imported into R Statistical Package v3.2.3 (www.r-project.org), analyzed using LIMMA package v3.26.9 and voom transformed. Genes differentially expressed between cell lines were identified using linear regression models. *p*-values were adjusted using the Benjamini and Hochberg's method. GO process enrichment was computed using MetaCore^TM^ v6.27 for all differentially expressing genes with a FDR *p*-value < 0.05.

### Gene expression with quantitative real-time PCR (qRT-PCR)

Total cell RNA was isolated using the Qiagen RNeasy Kit and reverse transcribed using the Transcriptor First Strand cDNA Synthesis Kit (Roche). SYBR green qRT-PCR was performed on a Lightcycler^®^ 480 (Roche). Gene expression was normalized against GAPDH and analyzed using the ΔΔC_t_ method. The ΔC_t_ method was used for plotting heatmaps. PCR primers are detailed in Supplementary Table S8.

### Western blot analysis

Cells were lyzed and processed as described previously [94]. Protein densitometric analysis was conducted using Image J software (http://imagej.nih.gov/ij/). The antibodies used are detailed in Supplementary Table S9.

### Cell morphology assessment

Cell morphology was assessed using the AMG EVOS FL (Advanced Microscopy Group) phase-contrast microscope.

### Proliferation assay

5000 cells/well were seeded into 96 well plates and allowed to adhere overnight. Cellular proliferation was assayed with AlamarBlue (Life Technologies) reagent and measured using a FLUOstar OPTIMA microplate reader (BMG Labtech) every 24 hr over a 96 hr period as previously reported [94].

### Clonogenic survival assay

1000 cells/well were seeded into 6 well plates and cultured for 7 days. Cell colonies were fixed and processed as previously described [94]. Discrete colonies (> 50 cells/colony) were counted using MetaMorph software (Molecular Devices).

### Migration assay

25,000 cells/well were seeded into 96 well plates and allowed to adhere overnight to achieve a confluent monolayer. Following wounding with a robot-assisted (Sciclone ALH3000 Workstation, Caliper Life Science) 1.67 mm diameter pin tool (FP3-WP, V&P Scientific), cells were cultured in DMEM containing 1% FBS for the remainder of the assay. At 0 and 40 hr after wounding, cells were fixed with 4% paraformaldehyde (Santa Cruz Biotechnologies), stained with DAPI (Thermo Fisher Scientific), and imaged using a Cellomics ArrayScan VTI HCS reader (ThermoFisher Scientific). The extent of wound closure was quantified using MetaMorph software.

### Invasion assay

Following 24 hr of serum starvation, 400,000 cells suspended in 200 μL of 3:2 serum free DMEM and Matrigel™ were placed into the upper compartment of a Transwell (Corning) chamber. 800 μL of DMEM containing 10% FBS was added to the lower compartment. Cells were allowed to invade across Matrigel™ and an 8 μm pore membrane for 24 hr at 37°C and 5% CO_2_. Cells remaining on the membrane's upper surface were removed with a cotton swab, whilst invaded cells on the membrane's under surface were fixed in 4% paraformaldehyde and stained with DAPI. Membranes were mounted and visualized using a BX51 microscope. Five representative fields per membrane were imaged and invaded cells counted using Image J software.

### Apoptosis assay

25,000 cells/well were seeded into 24 well plates and cultured for 48 hr. Cells were stained with annexin V-APC antibody (BD Pharmigen) and propidium iodide (Molecular Probes) as previously described [94]. The extent of apoptosis was measured by flow cytometry (BD FACSCanto™ II, BD Bioscience) and analyzed using Flowlogic software (Inivai Technologies).

### Luciferase-eGFP ectopic expression

Luciferase cDNA was stably expressed in FLO-1 parental and FLO-1^LM^ cells using the pFUGW lentiviral vector kindly provided by Dr. Mark Shackleton (PMCC, Australia). In this system, luciferase cDNA has been cloned downstream of the ubiquitin-C promoter and contains eGFP as a reporter gene [95]. Lentiviral particles were produced from HEK-293T cells using Lenti-X (Clontech) packaging mix according to manufacturer's instructions. FLO-1 cells were transduced with pFUGW and FACS sorted (BD FACSAria™ Fusion, BD Bioscience) for eGFP^+^ cells.

### Bioluminescence imaging

Luciferase-eGFP^+^ FLO-1 cells suspended in PBS and matrigel were subcutaneously injected into NSG (cohorts of 5 million, 1 million, 100 thousand and 10 thousand cells per mouse) and nude (5 million cells per mouse) mice. Animals were imaged fortnightly on a Xenogen IVIS 100 Imaging System (Caliper Life Science) to detect metastatic onset. 100 μl of 20 mg/ml luciferin (Promega) in PBS was subcutaneously injected into each mouse 5 min before imaging. Imaging was performed under general anesthesia. At experimental endpoint (either tumour volume >1500 mm^3^ or signs of ill health), the whole mouse and its organs were imaged to determine the extent and distribution of metastases. The imaging exposure times were 60 s for whole animal and 5 min for organs. The bioluminescence signal was quantified using the ‘region of interest’ function in Living Image software.

### E-cadherin knockdown

FLO-1 cells were transfected with 40 nM non-targeting control or *CDH1* siRNA (siGenome Smartpool, Dharmacon) using Lipofectamine RNAiMax solution (Life Technologies) according to manufacturer's instructions.

### E-cadherin cell sorting

FLO-1 parental cells were lifted using 0.5 mM EDTA/PBS mixture (30 min), filtered through a 40 μm nylon mesh, pelleted by centrifugation (1400 rpm, 4 min) and resuspended in blocking buffer (2% BSA and 2% FBS in PBS) for 1 hr at 4°C. Cells were then incubated in labeling buffer (2% BSA in PBS) with either anti-IgG (Dako) or anti-E-cadherin (EP700Y, Abcam) antibodies for 1 hr at 4°C. After two washes, cells were stained with Alexa Fluor 700 conjugated goat anti-rabbit IgG (Invitrogen) for 1 hr at 4°C in the dark. Cells were then washed thrice before the addition of 2% FluoroGold™ (Sigma-Aldrich). Viable FLO-1 cells were FACS sorted for E-cadherin high and low expressing populations, and separately injected into NSG mice (500,000 cells/mice). Animals were sacrificed once their subcutaneous tumours reached 1500 mm^3^ and lungs and livers were harvested for quantification of metastatic burden by PCR.

### Assessment of metastatic burden with qPCR

Genomic DNA from whole lungs and livers were extracted using the Qiagen DNA Blood and Tissue Kit. A multiplex TaqMan (Applied Biosystems) qPCR assay was performed on a StepOnePlus RT-PCR system (Applied Biosystems). Metastatic burden was quantified by normalizing the amount of human vimentin against mouse vimentin DNA per organ. PCR primers and probe sequences are detailed in Supplementary Table S10

### Microarray dataset analysis

*CDH1* expression from 64 treatment naive EAC cases were extracted from an Agilent 44K 60-mer oligo-microarray dataset (GSE19417) deposited at the NCBI Gene Expression Omnibus [19]. This cohort consisted of patients recruited from the Bristol Royal Infirmary, Bristol, UK between 1992 and 2000. Clinicopathological and outcome data were correlated with *CDH1* expression. Samples were stratified by *CDH1* high and low expression, and analyzed using the GEO2R platform from NCBI to identify significantly differentially expressed genes (FDR *p*-value < 0.05) for GO process enrichment analysis using MetaCore^TM^.

### Statistical analysis

Data were analyzed with Student's *t*-test or Mann-Whitney *U* test for parametric and non-parametric variables respectively. ANOVA with Dunnett's posttest was performed for multiple comparisons. Categorical variables were analyzed using the Chi-square test. Survival differences were compared using Kaplan-Meier log-rank analysis. A multivariate Cox regression analysis adjusting for clinicopathological variables (GSE19417: gender, nodal status, tumor location and grade) was also performed to interrogate differences in survival outcomes. Statistical analyses were conducted using R Statistical Packages and Prism 6 (GraphPad) with *p* < 0.05 considered statistically significant.

### Supplementary data

Additional supplementary information are available online including 5 figures and 10 tables. RNAseq data for FLO-1 parental and FLO-1^LM^ cell lines are available through the NCBI Gene Expression Omnibus public database (GSE88802).

## SUPPLEMENTARY MATERIALS













## References

[1] Rice TW, Blackstone EH, Rusch VW (2010). 7th edition of the AJCC Cancer Staging Manual: esophagus and esophagogastric junction. Ann Surg Oncol.

[2] Stahl M, Budach W, Meyer HJ, Cervantes A (2010). Esophageal cancer: Clinical Practice Guidelines for diagnosis, treatment and follow-up. Ann Oncol.

[3] Schweigert M, Dubecz A, Stein HJ (2013). Oesophageal cancer--an overview. Nat Rev Gastroenterol Hepatol.

[4] Tsai JH, Yang J (2013). Epithelial-mesenchymal plasticity in carcinoma metastasis. Genes Dev.

[5] Kalluri R, Weinberg RA (2009). The basics of epithelial-mesenchymal transition. J Clin Invest.

[6] Pei Y, Wang P, Liu H, He F, Ming L (2015). FOXQ1 promotes esophageal cancer proliferation and metastasis by negatively modulating CDH1. Biomed Pharmacother.

[7] Wang F, He W, Fanghui P, Wang L, Fan Q (2013). NF-kappaBP65 promotes invasion and metastasis of oesophageal squamous cell cancer by regulating matrix metalloproteinase-9 and epithelial-to-mesenchymal transition. Cell Biol Int.

[8] Kong KL, Kwong DL, Chan TH, Law SY, Chen L, Li Y, Qin YR, Guan XY (2012). MicroRNA-375 inhibits tumour growth and metastasis in oesophageal squamous cell carcinoma through repressing insulin-like growth factor 1 receptor. Gut.

[9] Hu T, Qi H, Li P, Zhao G, Ma Y, Hao Q, Gao C, Zhang Y, Wang C, Yang M, Hoffman RM, Chen P, Dong Z (2015). Comparison of GFP-Expressing Imageable Mouse Models of Human Esophageal Squamous Cell Carcinoma Established in Various Anatomical Sites. Anticancer Res.

[10] Gros SJ, Dohrmann T, Peldschus K, Schurr PG, Kaifi JT, Kalinina T, Reichelt U, Mann O, Strate TG, Adam G, Hoffman RM, Izbicki JR (2010). Complementary use of fluorescence and magnetic resonance imaging of metastatic esophageal cancer in a novel orthotopic mouse model. Int J Cancer.

[11] Gros SJ, Kurschat N, Dohrmann T, Reichelt U, Dancau AM, Peldschus K, Adam G, Hoffman RM, Izbicki JR, Kaifi JT (2010). Effective therapeutic targeting of the overexpressed HER-2 receptor in a highly metastatic orthotopic model of esophageal carcinoma. Mol Cancer Ther.

[12] Li W, Ding F, Zhang L, Liu Z, Wu Y, Luo A, Wu M, Wang M, Zhan Q (2005). Overexpression of stefin A in human esophageal squamous cell carcinoma cells inhibits tumor cell growth, angiogenesis, invasion, and metastasis. Clin Cancer Res.

[13] Zaidi AH, Saldin LT, Kelly LA, Bergal L, Londono R, Kosovec JE, Komatsu Y, Kasi PM, Shetty AA, Keane TJ, Thakkar SJ, Huleihel L, Landreneau RJ (2015). MicroRNA signature characterizes primary tumors that metastasize in an esophageal adenocarcinoma rat model. PloS one.

[14] Lange T, Nentwich MF, Luth M, Yekebas E, Schumacher U (2011). Trastuzumab has anti-metastatic and anti-angiogenic activity in a spontaneous metastasis xenograft model of esophageal adenocarcinoma. Cancer Lett.

[15] Zhou Z, Ran YL, Hu H, Pan J, Li ZF, Chen LZ, Sun LC, Peng L, Zhao XL, Yu L, Sun LX, Yang ZH (2008). TM4SF3 promotes esophageal carcinoma metastasis via upregulating ADAM12m expression. Clin Exp Metastasis.

[16] Raggi M, Langer R, Feith M, Friess H, Schauer M, Theisen J (2010). Successful evaluation of a new animal model using mice for esophageal adenocarcinoma. Langenbecks Arch Surg.

[17] Kato C, Fujii E, Chen YJ, Endaya BB, Matsubara K, Suzuki M, Ohnishi Y, Tamaoki N (2009). Spontaneous thymic lymphomas in the non-obese diabetic/Shi-scid, IL-2R gamma (null) mouse. Lab Anim.

[18] Jenkins DE, Oei Y, Hornig YS, Yu SF, Dusich J, Purchio T, Contag PR (2003). Bioluminescent imaging (BLI) to improve and refine traditional murine models of tumor growth and metastasis. Clin Exp Metastasis.

[19] Peters CJ, Rees JR, Hardwick RH, Hardwick JS, Vowler SL, Ong CA, Zhang C, Save V, O'Donovan M, Rassl D, Alderson D, Caldas C, Fitzgerald RC (2010). A 4-gene signature predicts survival of patients with resected adenocarcinoma of the esophagus, junction, and gastric cardia. Gastroenterology.

[20] Jian WG, Darnton SJ, Jenner K, Billingham LJ, Matthews HR (1997). Expression of E-cadherin in oesophageal carcinomas from the UK, China: disparities in prognostic significance. J Clin Pathol.

[21] Feilchenfeldt J, Varga Z, Siano M, Grabsch HI, Held U, Schuknecht B, Trip A, Hamaguchi T, Gut P, Balague O, Khanfir K, Diebold J, Jochum W (2015). Brain metastases in gastro-oesophageal adenocarcinoma: insights into the role of the human epidermal growth factor receptor 2 (HER2). Br J Cancer.

[22] Swami S, Kumble S, Triadafilopoulos G (1995). E-cadherin expression in gastroesophageal reflux disease, Barrett's esophagus, and esophageal adenocarcinoma: an immunohistochemical and immunoblot study. Am J Gastroenterol.

[23] Zhang LH, Huang Q, Fan XS, Wu HY, Yang J, Feng AN (2013). Clinicopathological significance of SIRT1 and p300/CBP expression in gastroesophageal junction (GEJ) cancer and the correlation with E-cadherin and MLH1. Pathol Res Pract.

[24] Schauer MC, Stoecklein NH, Theisen J, Kropil F, Baldus S, Hoelscher A, Feith M, Bolke E, Matuschek C, Budach W, Knoefel WT (2012). The simultaneous expression of both ephrin B3 receptor and E-cadherin in Barrett's adenocarcinoma is associated with favorable clinical staging. Eur J Med Res.

[25] Bronson NW, Diggs BS, Bakis G, Gatter KM, Sheppard BC, Hunter JG, Dolan JP (2015). Molecular Marker Expression Is Highly Heterogeneous in Esophageal Adenocarcinoma and Does Not Predict a Response to Neoadjuvant Therapy. J Gastrointest Surg.

[26] Feith M, Stein HJ, Mueller J, Siewert JR (2004). Malignant degeneration of Barrett's esophagus: the role of the Ki-67 proliferation fraction, expression of E-cadherin and p53. Dis Esophagus.

[27] Krishnadath KK, Tilanus HW, van Blankenstein M, Hop WC, Kremers ED, Dinjens WN, Bosman FT (1997). Reduced expression of the cadherin-catenin complex in oesophageal adenocarcinoma correlates with poor prognosis. J Pathol.

[28] Luber B, Deplazes J, Keller G, Walch A, Rauser S, Eichmann M, Langer R, Hofler H, Hegewisch-Becker S, Folprecht G, Woll E, Decker T, Endlicher E (2011). Biomarker analysis of cetuximab plus oxaliplatin/leucovorin/5-fluorouracil in first-line metastatic gastric and oesophago-gastric junction cancer: results from a phase II trial of the Arbeitsgemeinschaft Internistische Onkologie (AIO). BMC Cancer.

[29] Bongiorno PF, al-Kasspooles M, Lee SW, Rachwal WJ, Moore JH, Whyte RI, Orringer MB, Beer DG (1995). E-cadherin expression in primary and metastatic thoracic neoplasms and in Barrett's oesophagus. Br J Cancer.

[30] Falkenback D, Nilbert M, Oberg S, Johansson J (2008). Prognostic value of cell adhesion in esophageal adenocarcinomas. Dis Esophagus.

[31] Bailey T, Biddlestone L, Shepherd N, Barr H, Warner P, Jankowski J (1998). Altered cadherin and catenin complexes in the Barrett's esophagus-dysplasia-adenocarcinoma sequence: correlation with disease progression and dedifferentiation. Am J Pathol.

[32] Clement G, Braunschweig R, Pasquier N, Bosman FT, Benhattar J (2006). Methylation of APC, TIMP3, and TERT: a new predictive marker to distinguish Barrett's oesophagus patients at risk for malignant transformation. J Pathol.

[33] Eads CA, Lord RV, Wickramasinghe K, Long TI, Kurumboor SK, Bernstein L, Peters JH, DeMeester SR, DeMeester TR, Skinner KA, Laird PW (2001). Epigenetic patterns in the progression of esophageal adenocarcinoma. Cancer Res.

[34] Schildhaus HU, Krockel I, Lippert H, Malfertheiner P, Roessner A, Schneider-Stock R (2005). Promoter hypermethylation of p16INK4a, E-cadherin, O6-MGMT, DAPK and FHIT in adenocarcinomas of the esophagus, esophagogastric junction and proximal stomach. Int J Oncol.

[35] Brock MV, Gou M, Akiyama Y, Muller A, Wu TT, Montgomery E, Deasel M, Germonpre P, Rubinson L, Heitmiller RF, Yang SC, Forastiere AA, Baylin SB (2003). Prognostic importance of promoter hypermethylation of multiple genes in esophageal adenocarcinoma. Clin Cancer Res.

[36] Corn PG, Heath EI, Heitmiller R, Fogt F, Forastiere AA, Herman JG, Wu TT (2001). Frequent hypermethylation of the 5′ CpG island of E-cadherin in esophageal adenocarcinoma. Clin Cancer Res.

[37] Mikhail S, Ciombor K, Noonan A, Wu C, Goldberg R, Zhao W, Wei L, Mathey K, Yereb M, Timmers C, Bekaii-Saab T (2015). Upfront molecular testing in patients with advanced gastro-esophageal cancer: Is it time yet?. Oncotarget.

[38] Shiozaki H, Doki Y, Yamana H, Isono K (2002). A multi-institutional study of immunohistochemical investigation for the roles of cyclin D1 and E-cadherin in superficial squamous cell carcinoma of the esophagus. J Surg Oncol.

[39] Shiozaki H, Tahara H, Oka H, Miyata M, Kobayashi K, Tamura S, Iihara K, Doki Y, Hirano S, Takeichi M (1991). Expression of immunoreactive E-cadherin adhesion molecules in human cancers. Am J Pathol.

[40] Chung Y, Lam AK, Luk JM, Law S, Chan KW, Lee PY, Wong J (2007). Altered E-cadherin expression and p120 catenin localization in esophageal squamous cell carcinoma. Ann Surg Oncol.

[41] Nakanishi Y, Ochiai A, Akimoto S, Kato H, Watanabe H, Tachimori Y, Yamamoto S, Hirohashi S (1997). Expression of E-cadherin, alpha-catenin, beta-catenin and plakoglobin in esophageal carcinomas and its prognostic significance: immunohistochemical analysis of 96 lesions. Oncology.

[42] Tamura S, Shiozaki H, Miyata M, Kadowaki T, Inoue M, Matsui S, Iwazawa T, Takayama T, Takeichi M, Monden M (1996). Decreased E-cadherin expression is associated with haematogenous recurrence and poor prognosis in patients with squamous cell carcinoma of the oesophagus. Brit J Surg.

[43] Inada S, Koto T, Futami K, Arima S, Iwashita A (1999). Evaluation of malignancy and the prognosis of esophageal cancer based on an immunohistochemical study (p53, E-cadherin, epidermal growth factor receptor). Surg Today.

[44] Sato F, Shimada Y, Watanabe G, Uchida S, Makino T, Imamura M (1999). Expression of vascular endothelial growth factor, matrix metalloproteinase-9 and E-cadherin in the process of lymph node metastasis in oesophageal cancer. Br J Cancer.

[45] Setoyama T, Natsugoe S, Okumura H, Matsumoto M, Uchikado Y, Yokomakura N, Ishigami S, Aikou T (2007). alpha-catenin is a significant prognostic factor than E-cadherin in esophageal squamous cell carcinoma. J Surg Oncol.

[46] Chiba T, Kawachi H, Kawano T, Kumagai J, Kitagaki K, Sekine M, Uchida K, Kobayashi M, Sugihara K, Eishi Y (2010). Independent histological risk factors for lymph node metastasis of superficial esophageal squamous cell carcinoma; implication of claudin-5 immunohistochemistry for expanding the indications of endoscopic resection. Dis Esophagus.

[47] Uchikado Y, Natsugoe S, Okumura H, Setoyama T, Matsumoto M, Ishigami S, Aikou T (2005). Slug Expression in the E-cadherin preserved tumors is related to prognosis in patients with esophageal squamous cell carcinoma. Clin Cancer Res.

[48] Natsugoe S, Uchikado Y, Okumura H, Matsumoto M, Setoyama T, Tamotsu K, Kita Y, Sakamoto A, Owaki T, Ishigami S, Aikou T (2007). Snail plays a key role in E-cadherin-preserved esophageal squamous cell carcinoma. Oncol Rep.

[49] Ling ZQ, Li P, Ge MH, Zhao X, Hu FJ, Fang XH, Dong ZM, Mao WM (2011). Hypermethylation-modulated down-regulation of CDH1 expression contributes to the progression of esophageal cancer. Int J Mol Med.

[50] Takeno S, Noguchi T, Fumoto S, Kimura Y, Shibata T, Kawahara K (2004). E-cadherin expression in patients with esophageal squamous cell carcinoma: promoter hypermethylation, Snail overexpression, and clinicopathologic implications. Am J Clin Pathol.

[51] Sasaki K, Natsugoe S, Ishigami S, Matsumoto M, Okumura H, Setoyama T, Uchikado Y, Kita Y, Tamotsu K, Sakamoto A, Owaki T, Aikou T (2009). Significance of Twist expression and its association with E-cadherin in esophageal squamous cell carcinoma. J Exp Clin Cancer Res.

[52] Shimada Y, Hashimoto Y, Kan T, Kawamura J, Okumura T, Soma T, Kondo K, Teratani N, Watanabe G, Ino Y, Sakamoto M, Hirohashi S, Imamura M (2004). Prognostic significance of dysadherin expression in esophageal squamous cell carcinoma. Oncology.

[53] Zhang G, Zhou X, Xue L, Quan L, Wang Y, Zhou C, Lu N, Wang Q, Zhu H, Xu N (2005). Accumulation of cytoplasmic beta-catenin correlates with reduced expression of E-cadherin, but not with phosphorylated Akt in esophageal squamous cell carcinoma: immunohistochemical study. Pathol Int.

[54] Zhai JW, Yang XG, Yang FS, Hu JG, Hua WX (2010). Expression and clinical significance of Ezrin and E-cadherin in esophageal squamous cell carcinoma. Chin J Cancer.

[55] Shen WD, Ji Y, Liu PF, Xiang B, Chen GQ, Huang B, Wu S (2012). Correlation of E-cadherin and CD44v6 expression with clinical pathology in esophageal carcinoma. Mol Med Rep.

[56] Zhao XJ, Li H, Chen H, Liu YX, Zhang LH, Liu SX, Feng QL (2003). Expression of e-cadherin and beta-catenin in human esophageal squamous cell carcinoma: relationships with prognosis. World J Gastroenterol.

[57] Kadowaki T, Shiozaki H, Inoue M, Tamura S, Oka H, Doki Y, Iihara K, Matsui S, Iwazawa T, Nagafuchi A (1994). E-cadherin and alpha-catenin expression in human esophageal cancer. Cancer Res.

[58] Lin YC, Wu MY, Li DR, Wu XY, Zheng RM (2004). Prognostic and clinicopathological features of E-cadherin, alpha-catenin, beta-catenin, gamma-catenin and cyclin D1 expression in human esophageal squamous cell carcinoma. World J Gastroenterol.

[59] Miyata M, Shiozaki H, Iihara K, Shimaya K, Oka H, Kadowaki T, Inoue M, Tamura S, Takeichi M, Mori T (1994). Relationship between e-cadherin expression and lymph-node metastasis in human esophageal cancer. Int J Oncol.

[60] Li B, Wang B, Niu LJ, Jiang L, Qiu CC (2011). Hypermethylation of multiple tumor-related genes associated with DNMT3b up-regulation served as a biomarker for early diagnosis of esophageal squamous cell carcinoma. Epigenetics.

[61] Lee EJ, Lee BB, Han J, Cho EY, Shim YM, Park J, Kim DH (2008). CpG island hypermethylation of E-cadherin (CDH1) and integrin alpha4 is associated with recurrence of early stage esophageal squamous cell carcinoma. Int J Cancer.

[62] Guo M, Ren J, House MG, Qi Y, Brock MV, Herman JG (2006). Accumulation of promoter methylation suggests epigenetic progression in squamous cell carcinoma of the esophagus. Clin Cancer Res.

[63] Luketich JD, Friedman DM, Weigel TL, Meehan MA, Keenan RJ, Townsend DW, Meltzer CC (1999). Evaluation of distant metastases in esophageal cancer: 100 consecutive positron emission tomography scans. Ann Thorac Surg.

[64] Gros SJ, Dohrmann T, Rawnaq T, Kurschat N, Bouvet M, Wessels J, Hoffmann RM, Izbicki JR, Kaifi JT (2010). Orthotopic fluorescent peritoneal carcinomatosis model of esophageal cancer. Anticancer Res.

[65] Shultz LD, Ishikawa F, Greiner DL (2007). Humanized mice in translational biomedical research. Nat Rev Immunol.

[66] Shultz LD, Lyons BL, Burzenski LM, Gott B, Chen X, Chaleff S, Kotb M, Gillies SD, King M, Mangada J, Greiner DL, Handgretinger R (2005). Human lymphoid and myeloid cell development in NOD/LtSz-scid IL2R gamma null mice engrafted with mobilized human hemopoietic stem cells. J Immunol.

[67] Ito M, Hiramatsu H, Kobayashi K, Suzue K, Kawahata M, Hioki K, Ueyama Y, Koyanagi Y, Sugamura K, Tsuji K, Heike T, Nakahata T (2002). NOD/SCID/gamma(c)(null) mouse: an excellent recipient mouse model for engraftment of human cells. Blood.

[68] Ishikawa F, Yasukawa M, Lyons B, Yoshida S, Miyamoto T, Yoshimoto G, Watanabe T, Akashi K, Shultz LD, Harada M (2005). Development of functional human blood and immune systems in NOD/SCID/IL2 receptor {gamma} chain(null) mice. Blood.

[69] van Roy F, Berx G (2008). The cell-cell adhesion molecule E-cadherin. Cell Mol Life Sci.

[70] Xu XL, Ling ZQ, Chen SZ, Li B, Ji WH, Mao WM (2014). The impact of E-cadherin expression on the prognosis of esophageal cancer: a meta-analysis. Dis Esophagus.

[71] Kalatskaya I (2016). Overview of major molecular alterations during progression from Barrett's esophagus to esophageal adenocarcinoma. Ann N Y Acad Sci.

[72] Chao A, Lin CY, Lee YS, Tsai CL, Wei PC, Hsueh S, Wu TI, Tsai CN, Wang CJ, Chao AS, Wang TH, Lai CH (2012). Regulation of ovarian cancer progression by microRNA-187 through targeting Disabled homolog-2. Oncogene.

[73] Zhou J, Li K, Gu Y, Feng B, Ren G, Zhang L, Wang Y, Nie Y, Fan D (2011). Transcriptional up-regulation of RhoE by hypoxia-inducible factor (HIF)-1 promotes epithelial to mesenchymal transition of gastric cancer cells during hypoxia. Biochem Biophys Res Commun.

[74] Zhou J, Yang J, Li K, Mo P, Feng B, Wang X, Nie Y, Fan D (2013). RhoE is associated with relapse and prognosis of patients with colorectal cancer. Ann Surg Oncol.

[75] Qiu G, Sun W, Zou Y, Cai Z, Wang P, Lin X, Huang J, Jiang L, Ding X, Hu G (2015). RNA interference against TMEM97 inhibits cell proliferation, migration, and invasion in glioma cells. Tumour Biol.

[76] Alnabulsi A, Agouni A, Mitra S, Garcia-Murillas I, Carpenter B, Bird S, Murray GI (2012). Cellular apoptosis susceptibility (chromosome segregation 1-like, CSE1L) gene is a key regulator of apoptosis, migration and invasion in colorectal cancer. J Pathol.

[77] Tokuo H, Coluccio LM (2013). Myosin-1c regulates the dynamic stability of E-cadherin-based cell-cell contacts in polarized Madin-Darby canine kidney cells. Mol Biol Cell.

[78] Novellino L, De Filippo A, Deho P, Perrone F, Pilotti S, Parmiani G, Castelli C (2008). PTPRK negatively regulates transcriptional activity of wild type and mutated oncogenic beta-catenin and affects membrane distribution of beta-catenin/E-cadherin complexes in cancer cells. Cell Signal.

[79] Chastre E, Abdessamad M, Kruglov A, Bruyneel E, Bracke M, Di Gioia Y, Beckerle MC, van Roy F, Kotelevets L (2009). TRIP6, a novel molecular partner of the MAGI-1 scaffolding molecule, promotes invasiveness. FASEB J.

[80] Yamamura M, Noguchi K, Nakano Y, Segawa E, Zushi Y, Takaoka K, Kishimoto H, Hashimoto-Tamaoki T, Urade M (2013). Functional analysis of Zyxin in cell migration and invasive potential of oral squamous cell carcinoma cells. Int J Oncol.

[81] Grunewald TG, Willier S, Janik D, Unland R, Reiss C, Prazeres da Costa O, Buch T, Dirksen U, Richter GH, Neff F, Burdach S, Butt E (2013). The Zyxin-related protein thyroid receptor interacting protein 6 (TRIP6) is overexpressed in Ewing's sarcoma and promotes migration, invasion and cell growth. Biol Cell.

[82] Fei J, Li J, Shen S, Zhou W (2013). Characterization of TRIP6-dependent nasopharyngeal cancer cell migration. Tumour Biol.

[83] Vuchak LA, Tsygankova OM, Meinkoth JL (2011). Rap1GAP impairs cell-matrix adhesion in the absence of effects on cell-cell adhesion. Cell Adh Migr.

[84] Lee YE, He HL, Chen TJ, Lee SW, Chang IW, Hsing CH, Li CF (2015). The prognostic impact of RAP2A expression in patients with early and locoregionally advanced nasopharyngeal carcinoma in an endemic area. Am J Transl Res.

[85] Ji H, Greening DW, Barnes TW, Lim JW, Tauro BJ, Rai A, Xu R, Adda C, Mathivanan S, Zhao W, Xue Y, Xu T, Zhu HJ, Simpson RJ (2013). Proteome profiling of exosomes derived from human primary and metastatic colorectal cancer cells reveal differential expression of key metastatic factors and signal transduction components. Proteomics.

[86] Wu JX, Zhang DG, Zheng JN, Pei DS (2015). Rap2a is a novel target gene of p53 and regulates cancer cell migration and invasion. Cell Signal.

[87] Wang LP, Cao J, Zhang J, Wang BY, Hu XC, Shao ZM, Wang ZH, Ou ZL (2015). The human chemokine receptor CCRL2 suppresses chemotaxis and invasion by blocking CCL2-induced phosphorylation of p38 MAPK in human breast cancer cells. Med Oncol.

[88] Liu R, Li J, Xie K, Zhang T, Lei Y, Chen Y, Zhang L, Huang K, Wang K, Wu H, Wu M, Nice EC, Huang C, Wei Y (2013). FGFR4 promotes stroma-induced epithelial-to-mesenchymal transition in colorectal cancer. Cancer Res.

[89] Roberts TK, Eugenin EA, Lopez L, Romero IA, Weksler BB, Couraud PO, Berman JW (2012). CCL2 disrupts the adherens junction: implications for neuroinflammation. Lab Invest.

[90] Nam JS, Kang MJ, Suchar AM, Shimamura T, Kohn EA, Michalowska AM, Jordan VC, Hirohashi S, Wakefield LM (2006). Chemokine (C-C motif) ligand 2 mediates the prometastatic effect of dysadherin in human breast cancer cells. Cancer Res.

[91] Mestdagt M, Polette M, Buttice G, Noel A, Ueda A, Foidart JM, Gilles C (2006). Transactivation of MCP-1/CCL2 by beta-catenin/TCF-4 in human breast cancer cells. Int J Cancer.

[92] Taylor MD, Liu Y, Nagji AS, Theodosakis N, Jones DR (2010). Combined proteasome and histone deacetylase inhibition attenuates epithelial-mesenchymal transition through E-cadherin in esophageal cancer cells. J Thorac Cardiovasc Surg.

[93] Clemons NJ, Do H, Fennell C, Deb S, Fellowes A, Dobrovic A, Phillips WA (2014). Characterization of a novel tumorigenic esophageal adenocarcinoma cell line: OANC1. Dig Dis Sci.

[94] Liu DS, Read M, Cullinane C, Azar WJ, Fennell CM, Montgomery KG, Haupt S, Haupt Y, Wiman KG, Duong CP, Clemons NJ, Phillips WA (2015). APR-246 potently inhibits tumour growth and overcomes chemoresistance in preclinical models of oesophageal adenocarcinoma. Gut.

[95] Quintana E, Piskounova E, Shackleton M, Weinberg D, Eskiocak U, Fullen DR, Johnson TM, Morrison SJ (2012). Human melanoma metastasis in NSG mice correlates with clinical outcome in patients. Sci Transl Med.

[96] Boonstra JJ, van Marion R, Beer DG, Lin L, Chaves P, Ribeiro C, Pereira AD, Roque L, Darnton SJ, Altorki NK, Schrump DS, Klimstra DS, Tang LH, Eshleman JR, Alvarez H, Shimada Y (2010). Verification and unmasking of widely used human esophageal adenocarcinoma cell lines. J Natl Cancer Inst.

[97] Boonstra JJ, van der Velden AW, Beerens EC, van Marion R, Morita-Fujimura Y, Matsui Y, Nishihira T, Tselepis C, Hainaut P, Lowe AW, Beverloo BH, van Dekken H, Tilanus HW, Dinjens WN (2007). Mistaken identity of widely used esophageal adenocarcinoma cell line TE-7. Cancer Res.

[98] Alvarez H, Koorstra JB, Hong SM, Boonstra JJ, Dinjens WN, Foratiere AA, Wu TT, Montgomery E, Eshleman JR, Maitra A (2008). Establishment and characterization of a bona fide Barrett esophagus-associated adenocarcinoma cell line. Cancer Biol Ther.

