# Supplementary material for: Novel metastatic models of esophageal adenocarcinoma derived from FLO-1 cells highlight the importance of E-cadherin in cancer metastasis
Source: Oncotarget. 2016 Nov 16;7(50):83342–58. doi: 10.18632/oncotarget.13391 (PMC5347774; doi:10.18632/oncotarget.13391)
Supplement: Supplementary file 1 [file oncotarget-07-83342-s001.pdf]

Novel metastatic models of esophageal adenocarcinoma derived from FLO-1 cells highlight the importance of E-cadherin in cancer metastasis

Supplementary Materials

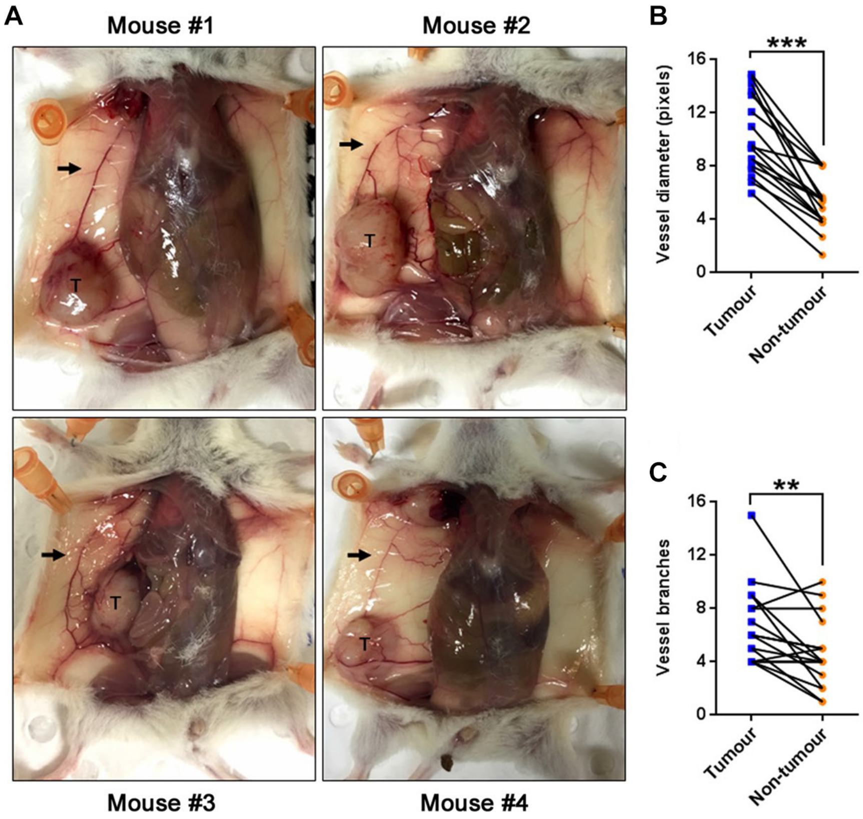

Supplementary Figure S1: Related to Figure 1, FLO-1 spontaneously metastasizes in NOD-SCID IL-2Rγ^{KO} (NSG) mice. (**A–C**) Examples of 4 (Total 17) mice bearing subcutaneous FLO-1 xenografts (T). The mammary artery (arrow) on the ipsilateral side of the tumor was compared to its contralateral counterpart with respect to vessel diameter (B) and number of branching points (C). Vessel diameter was measured at the midpoint from the axilla to the groin. A branching point was considered when a smaller caliber vessel originated directly from the mammary artery.

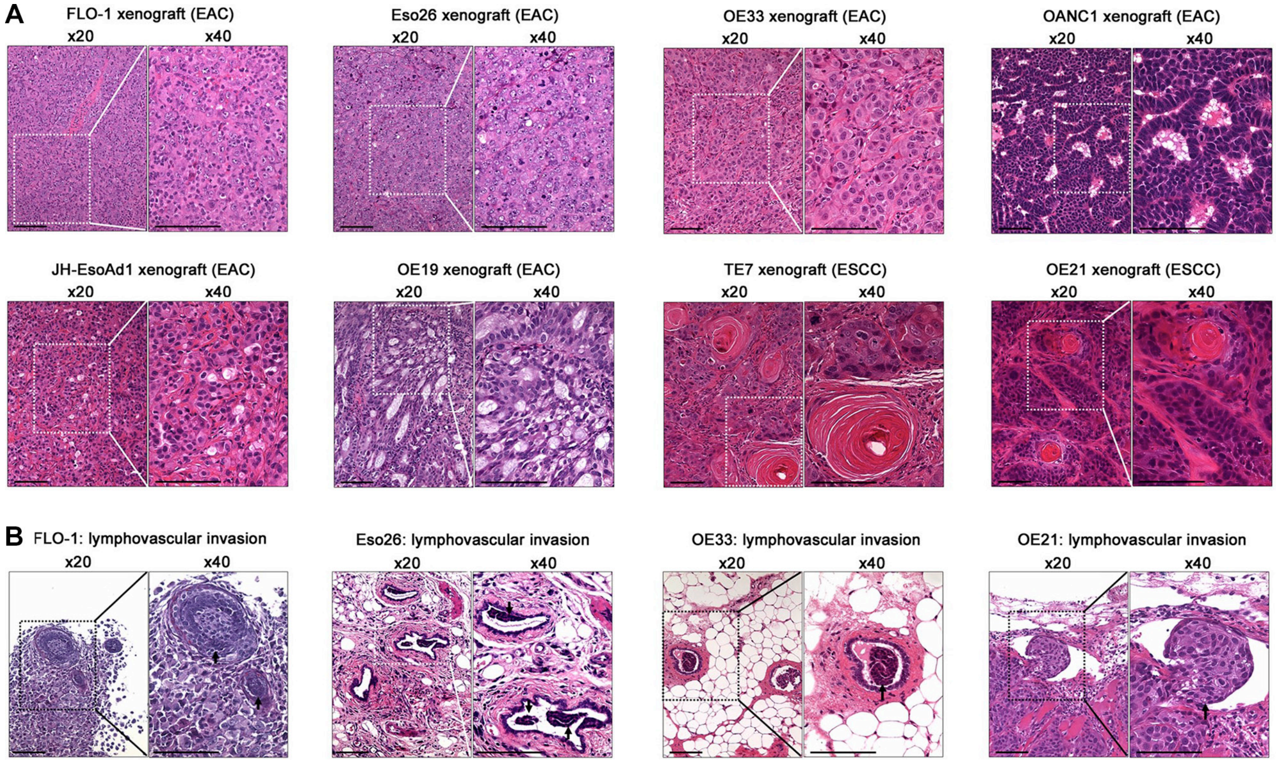

Supplementary Figure S2: Related to Figure 2, FLO-1 exhibits a mesenchymal phenotype. (**A**–**B**) H&E staining of all cell line xenografts demonstrating tumor differentiation (A) and lymphovascular invasion (B, arrows). Esophageal adenocarcinoma (EAC), esophageal squamous cell carcinoma (ESCC). Scale bar = 100 µm.

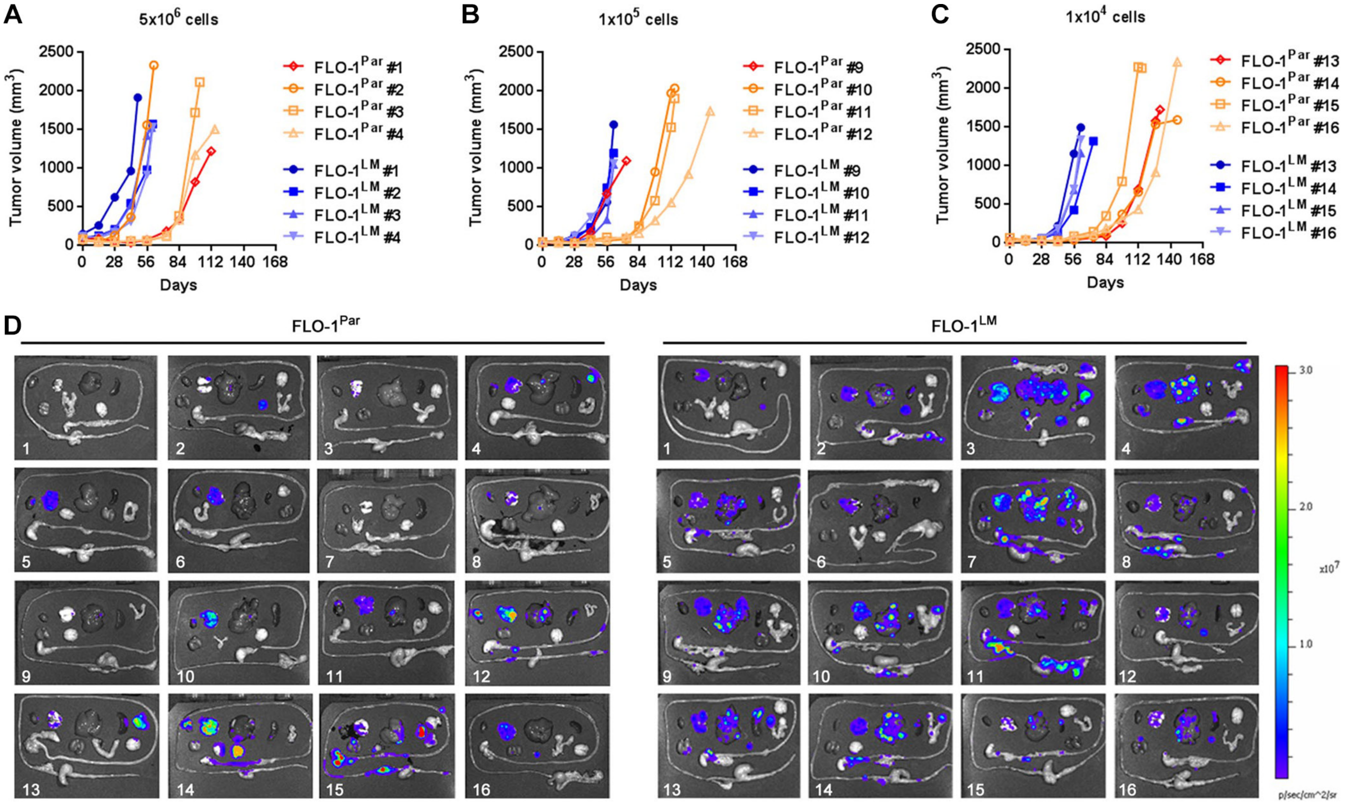

Supplementary Figure S3: Related to Figure 4, FLO-1^{LM} has increased proliferative and metastatic capacity *in vivo*. (**A**–**C**) Growth curves of FLO-1^{Par} and FLO-1^{LM} xenografts following subcutaneous injection of 5 million (A), 100 thousand (B) and 10 thousand (C) cells into the flank of NSG mice. (**D**) Bioluminescent heatmaps of organs from all mice bearing either FLO-1^{Par} and FLO-1^{LM} xenografts.

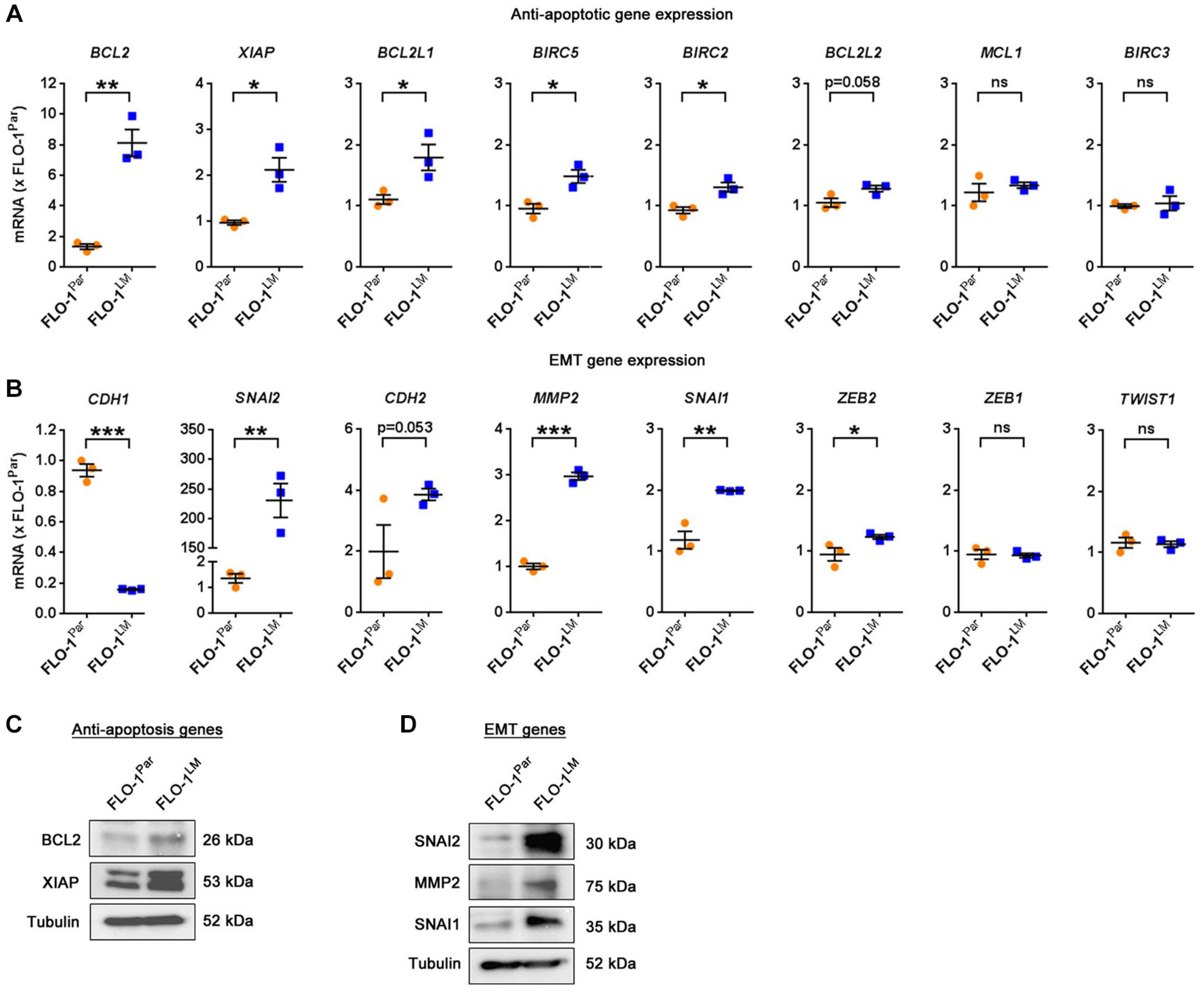

Supplementary Figure S4: Related to Figure 5, FLO-1^{LM} highlights molecular pathways that are deranged in metastasis. (**A**–**B**) qRT-PCR of anti-apoptotic (A) and EMT (B) genes expressed in FLO-1^{LM} compared with FLO-1^{Par} cells. (**C**–**D**) Western blots of anti-apoptotic (C) and EMT (D) genes expressed in FLO-1^{LM} compared with FLO-1^{Par} cells. Unpaired *t*-test. Error bars = SEM, $n = 3$ for A and B, $n = 2$ for C and D, *$p < 0.05$, **$p < 0.01$, ***$p < 0.001$, ns = non-significant.

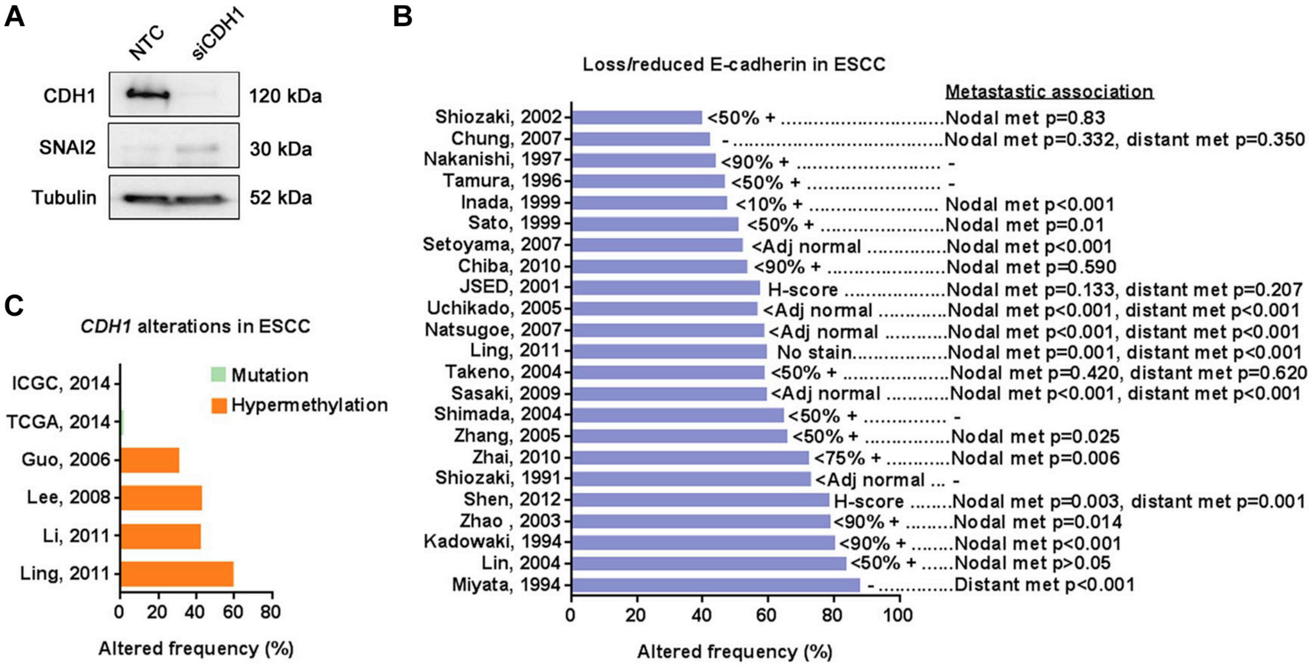

Supplementary Figure S5: Related to Figure 6, Low E-cadherin expression is associated with increased metastasis in FLO-1 and reduced patient survival. (**A**) Western blot of SNAI2 at 72 hr post transfection with *CDH1* and non-targeting control (NTC) siRNA. (**B**) Frequency of lost/reduced E-cadherin expression in esophageal squamous cell carcinoma (ESCC) from published studies, and its association with lymph node and distant organ metastasis. (-) Not reported. The annotations immediately adjacent to each bar indicates the study's definition of loss/reduced E-cadherin expression. (**C**) Frequency of *CDH1* genetic alterations in ESCC in TCGA and ICGC datasets as well as from published studies. $n = 2$ for A.

Supplementary Table S1: STR analysis of FLO-1 parental and FLO-1^{LM} cell lines

	FLO-1 parental		FLO-1LM	
**Marker**	**Genotype**	**Peak sizes**	**Genotype**	**Peak sizes**
AMEL	X	104.1	X	103.96
CSF1PO	11	337.56	11	337.39
D13S317	11	187.98	11	187.62
D16S539	12, 13	290.9, 294.82	12, 13	290.72, 294.73
D18S51	14, 16	306.34, 313.81	14, 16	306.43, 313.84
D21S11	30, 32.2	223.42, 233.26	30, 32.2	223.21, 233.17
D3S1358	15	124.32	15	124.15
D5S818	12, 14	134.75, 143.05	12, 14	134.58, 142.73
D7S820	8	220.36	8	220.11
D8S1179	13	226.33	13	226.14
FGA	21	341.43	21	341.31
Penta D	11, 12	408.16, 412.75	11, 12	408.16, 413.02
Penta E	5, 17	377.29, 438.89	5, 17	377.16, 438.83
TH01	6	161.98	6	161.66
TPOX	9, 11	272.92, 280.83	9, 11	272.89, 280.94
vWA	16	146.26	16	146.14

Supplementary Table S2: Significantly (FDR < 0.05) downregulated genes in FLO-1^{LM} compared with FLO-1^{Par}. See Supplementary_Table_S2.

Supplementary Table S3: Significantly (FDR < 0.05) upregulated genes in FLO-1^{LM} compared with FLO-1^{Par}. See Supplementary_Table_S3.

Supplementary Table S4: Enrichment analysis report: Top 200 GO processes for FLO-1^{LM} vs. FLO-1^{Par}. See Supplementary_Table_S4.

Supplementary Table S5: Differentially (FDR < 0.05) expressed genes in *CDH1* low vs. high tumors in GSE19417. See Supplementary_Table_S5.

Supplementary Table S6: Overlapping GO processes enriched in FLO-1^{LM} and *CDH1* low tumors. See Supplementary_Table_S6.

Supplementary Table S7: Patient demographics, tumor characteristics and survival (GSE19417 dataset)

	Overall (n = 64)	CDH1 High (n = 48)	CDH1 Low (n = 16)	OR/ HR	95% CI	*P* value
**Patient demographics**						
Gender, male (%)	46 (71.9)	36 (75.0)	10 (63.0)	0.55	0.17–1.85	0.34
**Tumour characteristics**						
Location						
Esophagus, *n* (%)	48 (75.0)	36 (75.0)	12 (75.0)	1.00	0.27–3.69	1.00
GEJ, *n* (%)	16 (25.0)	12 (25.0)	4 (25.0)			
Differentiation						
Poor, *n* (%)	31 (48.4)	23 (47.9)	8 (50.0)	1.74	0.18–17.22	1.00
Moderate, *n* (%)	27 (42.2)	20 (41.7)	7 (43.8)	1.75	0.17–17.69	1.00
Well, *n* (%)	6 (9.4)	5 (10.4)	1 (6.3)			
Nodal status						
Positive, *n* (%)	48 (75.0)	36 (75.0)	12 (75.0)	1.00	0.27–3.69	1.00
n positive, median (IQR)	5 (2–8)	4.5 (2.0–8.0)	5.0 (1.8–8.0)	–	–	0.96
**Outcome**						
OS, months, median (IQR)	18.8 (13.6–34.5)	25.9 (15.8–44.2)	6.5 (5.5–51.1)	**2.08***	**1.05 – 4.11**	**0.035**

Totals may not equal 100% due to rounding. Odds ratio (OR), hazard ratio (HR), confidence interval (CI), inter-quartile range (IQR), gastro-esophageal junction (GEJ), overall survival (OS). *CDH1* high and Low groups were compared using the Log-rank test for OS and Chi-square test for all other variables. *The HR presented is from a multivariable Cox regression model adjusting for sex, location, differentiation and nodal status (positive vs. negative).

Supplementary Table S8: RT-PCR primer sequences

Gene	Forward (5′-3′)	Reverse (5′-3′)
CDH1	TGCCACCCTGGCTTTGACGC	ATTCACTCTGCCCAGGACGCGG
CDH2	ATCGCATTATGCAAGACTGGATT	ATGCACATCCTTCGATAAGACTG
SNAI1	TCGGAAGCCTAACTACAGCGA	AGATGAGCATTGGCAGCGAG
SNAI2	AAGCATTTCAACGCCTCCAAA	GGATCTCTGGTTGTGGTATGACA
ZEB1	GATGATGAATGCGAGTCAGATGC	ACAGCAGTGTCTTGTTGTTGT
ZEB2	CAAGAGGCGCAAACAAGCC	GGTTGGCAATACCGTCATCC
MMP2	TACAGGATCATTGGCTACACACC	GGTCACATCGCTCCAGACT
TWIST1	GTCCGCAGTCTTACGAGGAG	GCTTGAGGGTCTGAATCTTGCT
BCL2	ATGTGTGTGGAGAGCGTCAA	TCATCCACAGGGCGATGTT
BCL2L1	TGGAGTCAGTTTAGTGATGTGGA	CCAGGATGGGTTGCCATTG
BCL2L2	GACAAGTGCAGGAGTGGATG	AAGGCCCCTACAGTTACCAG
BIRC2	GTCAAATGCTTCTGTTGTGGC	ACAAGCTACTATGTTCCAAGGTG
BIRC3	TCCACACACTCATTACTTCCG	TGGCCATGTCTGAAAAGTAAGT
BIRC4	TGAGAACTGGGCAGGTTGTA	CTCTTGGGGTTAGGTGAGCA
BIRC5	ACATTCAAGAACTGGCCCTTC	AAGTCTGGCTCGTTCTCAGT
MCL1	GTAAGGAGTCGGGGTCTTCC	CCCCACAGTAGAGGTTGAGT
GAPDH	GGTGTGAACCATGAG	CCAGCAGTTTCCCGGA

Supplementary Table S9: Antibodies for western blotting

Antibody	Origin	Clone	Source
**Anti-E-cadherin**	Rabbit	EP700Y	Abcam
**Anti-N-cadherin**	Mouse	32/N-Cadherin	BD Transduction Laboratories
**Anti-Vimentin**	Rabbit	R28	Cell Signaling Technology
**Anti-Snail**	Rabbit	C15D3	Cell Signaling Technology
**Anti-Slug**	Rabbit	C19G7	Cell Signaling Technology
**Anti-Zeb1**	Rabbit	D80D3	Cell Signaling Technology
**Anti-MMP2**	Mouse	2C1-1D12	ThermoFisher Scientific
**Anti-Bcl2**	Rabbit	50E3	Cell Signaling Technology
**Anti-XIAP**	Mouse	610763	BD Transduction Laboratory
**Anti-β-actin**	Mouse	C4	MP-Biomedical
**Anti-GAPDH**	Mouse	6C5	Abcam
**Anti-Tubulin**	Mouse	B-5-1-2	Sigma-Aldrich
**Swine anti-rabbit**	Swine	P0217	Dako
**Goat anti-mouse**	Goat	P0447	Dako

Supplementary Table S10: Human and mouse vimentin qPCR and probe sequences

Gene	qPCR primers	Probes
**hVimentin**	Forward (5′-3′): AGAGAACTTTGCCGTTGAAGCT	CCTGCAGGATGAGATTCAGAATATGG
	Reverse (5′-3″): GAAGGTGACGAGCCATTTCC	
**mVimentin**	Forward (5′-3′): CGAAGGTGACGAGCCATCTC	CCTTCATGTTTTGGATCTCATCCTGCAGG
	Reverse (5′-3″): AGCTGCTAACTACCAGGACACTATTG	
