# Supplementary material for: Novel metastatic models of esophageal adenocarcinoma derived from FLO-1 cells highlight the importance of E-cadherin in cancer metastasis
Source: Oncotarget. 2016 Nov 16;7(50):83342–58. doi: 10.18632/oncotarget.13391 (PMC5347774; doi:10.18632/oncotarget.13391)
Supplement: Supplementary file 2 [file oncotarget-07-83342-s002.docx]

**Supplementary Table S2.** Significantly (FDR<0.05) downregulated genes in FLO-1^LM^ compared with FLO-1^Par^

| **Rank** | **Ensembl ID** | **Gene name** | **Log_2_(Fold change)** | **FDR p-value** |
| --- | --- | --- | --- | --- |
| 1 | ENSG00000039068 | CDH1 | -2.21 | 1.71E-10 |
| 2 | ENSG00000273149 | RP11-290D2.6 | -2.13 | 1.21E-07 |
| 3 | ENSG00000170421 | KRT8 | -0.70 | 2.39E-07 |
| 4 | ENSG00000234961 | RP11-124N14.3 | -3.43 | 3.49E-07 |
| 5 | ENSG00000258017 | RP11-386G11.10 | -2.10 | 3.49E-07 |
| 6 | ENSG00000272540 | XXbac-BPG252P9.9 | -2.98 | 5.25E-07 |
| 7 | ENSG00000006210 | CX3CL1 | -0.91 | 5.25E-07 |
| 8 | ENSG00000115457 | IGFBP2 | -0.51 | 5.25E-07 |
| 9 | ENSG00000134321 | RSAD2 | -0.86 | 8.48E-07 |
| 10 | ENSG00000269968 | RP5-940J5.9 | -3.70 | 8.88E-07 |
| 11 | ENSG00000111057 | KRT18 | -0.66 | 1.07E-06 |
| 12 | ENSG00000268313 | AC119673.1 | -2.95 | 1.21E-06 |
| 13 | ENSG00000005102 | MEOX1 | -1.18 | 1.28E-06 |
| 14 | ENSG00000100342 | APOL1 | -0.66 | 1.28E-06 |
| 15 | ENSG00000167995 | BEST1 | -2.88 | 1.78E-06 |
| 16 | ENSG00000265401 | RP11-138I1.4 | -4.03 | 4.68E-06 |
| 17 | ENSG00000198695 | MT-ND6 | -0.82 | 5.30E-06 |
| 18 | ENSG00000042062 | FAM65C | -1.25 | 5.50E-06 |
| 19 | ENSG00000229124 | VIM-AS1 | -1.63 | 7.79E-06 |
| 20 | ENSG00000171345 | KRT19 | -1.31 | 1.11E-05 |
| 21 | ENSG00000188290 | HES4 | -0.85 | 1.85E-05 |
| 22 | ENSG00000258232 | RP11-161H23.5 | -3.16 | 1.99E-05 |
| 23 | ENSG00000135114 | OASL | -0.53 | 2.65E-05 |
| 24 | ENSG00000254285 | KRT8P3 | -1.01 | 4.80E-05 |
| 25 | ENSG00000119922 | IFIT2 | -0.49 | 4.91E-05 |
| 26 | ENSG00000136830 | FAM129B | -0.46 | 7.12E-05 |
| 27 | ENSG00000225339 | RP11-513I15.6 | -2.81 | 8.48E-05 |
| 28 | ENSG00000179818 | PCBP1-AS1 | -1.24 | 1.00E-04 |
| 29 | ENSG00000165757 | KIAA1462 | -0.55 | 1.04E-04 |
| 30 | ENSG00000161010 | C5orf45 | -0.93 | 1.56E-04 |
| 31 | ENSG00000172216 | CEBPB | -0.86 | 1.82E-04 |
| 32 | ENSG00000179862 | CITED4 | -1.03 | 2.02E-04 |
| 33 | ENSG00000268702 | AL049829.1 | -2.72 | 2.40E-04 |
| 34 | ENSG00000143882 | ATP6V1C2 | -2.02 | 2.55E-04 |
| 35 | ENSG00000059728 | MXD1 | -0.95 | 3.54E-04 |
| 36 | ENSG00000267469 | AC005944.2 | -5.15 | 3.83E-04 |
| 37 | ENSG00000176463 | SLCO3A1 | -0.63 | 4.26E-04 |
| 38 | ENSG00000124145 | SDC4 | -0.38 | 4.79E-04 |
| 39 | ENSG00000259716 | RP5-977B1.11 | -2.97 | 4.83E-04 |
| 40 | ENSG00000152377 | SPOCK1 | -0.42 | 5.45E-04 |
| 41 | ENSG00000218537 | AP000350.4 | -3.67 | 5.54E-04 |
| 42 | ENSG00000143320 | CRABP2 | -1.18 | 6.70E-04 |
| 43 | ENSG00000240668 | KRT8P36 | -1.38 | 6.75E-04 |
| 44 | ENSG00000074800 | ENO1 | -0.20 | 6.77E-04 |
| 45 | ENSG00000235027 | AC068580.6 | -5.72 | 7.01E-04 |
| 46 | ENSG00000158470 | B4GALT5 | -0.37 | 7.21E-04 |
| 47 | ENSG00000163644 | PPM1K | -0.72 | 7.77E-04 |
| 48 | ENSG00000221792 | MIR1282 | -3.26 | 7.88E-04 |
| 49 | ENSG00000162654 | GBP4 | -0.62 | 8.12E-04 |
| 50 | ENSG00000268310 | AC087645.1 | -4.72 | 8.30E-04 |
| 51 | ENSG00000259661 | AC068831.15 | -5.91 | 8.32E-04 |
| 52 | ENSG00000254422 | RP11-864G5.3 | -2.74 | 9.01E-04 |
| 53 | ENSG00000257342 | RP11-571M6.7 | -4.33 | 1.01E-03 |
| 54 | ENSG00000259357 | RP11-316M1.12 | -2.82 | 1.06E-03 |
| 55 | ENSG00000069702 | TGFBR3 | -0.51 | 1.07E-03 |
| 56 | ENSG00000225951 | ODF2-AS1 | -6.23 | 1.16E-03 |
| 57 | ENSG00000254873 | RP11-770J1.5 | -3.22 | 1.16E-03 |
| 58 | ENSG00000188157 | AGRN | -0.29 | 1.16E-03 |
| 59 | ENSG00000210144 | MT-TY | -2.48 | 1.25E-03 |
| 60 | ENSG00000172803 | SNX32 | -1.53 | 1.34E-03 |
| 61 | ENSG00000269292 | CTB-12A17.3 | -6.07 | 1.42E-03 |
| 62 | ENSG00000262831 | RP11-498C9.2 | -3.08 | 1.48E-03 |
| 63 | ENSG00000228830 | RP4-781K5.2 | -5.69 | 1.54E-03 |
| 64 | ENSG00000187144 | SPATA21 | -3.04 | 1.54E-03 |
| 65 | ENSG00000172183 | ISG20 | -0.56 | 1.54E-03 |
| 66 | ENSG00000227766 | HCG4P5 | -3.52 | 1.67E-03 |
| 67 | ENSG00000261604 | CTD-2636A23.2 | -3.58 | 1.88E-03 |
| 68 | ENSG00000271980 | CTD-2256P15.4 | -2.81 | 1.91E-03 |
| 69 | ENSG00000269871 | AC040977.1 | -2.56 | 1.91E-03 |
| 70 | ENSG00000168016 | TRANK1 | -0.71 | 2.05E-03 |
| 71 | ENSG00000157734 | SNX22 | -2.11 | 2.07E-03 |
| 72 | ENSG00000112096 | SOD2 | -0.30 | 2.29E-03 |
| 73 | ENSG00000167996 | FTH1 | -0.28 | 2.33E-03 |
| 74 | ENSG00000189143 | CLDN4 | -1.08 | 2.39E-03 |
| 75 | ENSG00000119917 | IFIT3 | -0.37 | 2.39E-03 |
| 76 | ENSG00000136305 | CIDEB | -5.71 | 2.43E-03 |
| 77 | ENSG00000059804 | SLC2A3 | -1.48 | 2.55E-03 |
| 78 | ENSG00000079332 | SAR1A | -0.35 | 2.57E-03 |
| 79 | ENSG00000042493 | CAPG | -1.00 | 2.77E-03 |
| 80 | ENSG00000129951 | LPPR3 | -0.54 | 2.78E-03 |
| 81 | ENSG00000005884 | ITGA3 | -0.32 | 2.83E-03 |
| 82 | ENSG00000161570 | CCL5 | -0.36 | 2.99E-03 |
| 83 | ENSG00000160683 | CXCR5 | -5.36 | 3.13E-03 |
| 84 | ENSG00000006534 | ALDH3B1 | -0.58 | 3.21E-03 |
| 85 | ENSG00000161714 | PLCD3 | -0.50 | 3.24E-03 |
| 86 | ENSG00000259342 | RP11-519G16.5 | -4.49 | 3.37E-03 |
| 87 | ENSG00000087245 | MMP2 | -0.65 | 3.45E-03 |
| 88 | ENSG00000186919 | ZACN | -2.74 | 4.05E-03 |
| 89 | ENSG00000267987 | AL161784.1 | -2.57 | 4.05E-03 |
| 90 | ENSG00000148180 | GSN | -0.29 | 4.17E-03 |
| 91 | ENSG00000187942 | LDLRAD2 | -4.13 | 4.41E-03 |
| 92 | ENSG00000028137 | TNFRSF1B | -0.55 | 5.69E-03 |
| 93 | ENSG00000127528 | KLF2 | -0.60 | 5.71E-03 |
| 94 | ENSG00000184584 | TMEM173 | -0.58 | 5.71E-03 |
| 95 | ENSG00000257550 | RP11-793H13.3 | -5.20 | 5.75E-03 |
| 96 | ENSG00000267458 | CTC-425F1.4 | -4.56 | 6.40E-03 |
| 97 | ENSG00000251143 | RP11-849H4.4 | -2.74 | 7.05E-03 |
| 98 | ENSG00000254236 | KB-1639H6.2 | -5.43 | 7.34E-03 |
| 99 | ENSG00000172232 | AZU1 | -0.56 | 7.35E-03 |
| 100 | ENSG00000267769 | CTB-50L17.9 | -5.42 | 7.47E-03 |
| 101 | ENSG00000128567 | PODXL | -0.37 | 7.49E-03 |
| 102 | ENSG00000260005 | AC027601.1 | -5.35 | 7.53E-03 |
| 103 | ENSG00000112309 | B3GAT2 | -3.94 | 7.55E-03 |
| 104 | ENSG00000244509 | APOBEC3C | -1.04 | 7.64E-03 |
| 105 | ENSG00000162783 | IER5 | -0.39 | 7.74E-03 |
| 106 | ENSG00000086300 | SNX10 | -0.48 | 7.74E-03 |
| 107 | ENSG00000227617 | CERS6-AS1 | -1.82 | 7.83E-03 |
| 108 | ENSG00000258959 | RP11-1017G21.4 | -5.56 | 7.94E-03 |
| 109 | ENSG00000227409 | ZMYM4-AS1 | -5.30 | 8.34E-03 |
| 110 | ENSG00000254230 | RP11-582J16.3 | -4.94 | 8.53E-03 |
| 111 | ENSG00000254741 | RP11-661A12.7 | -3.76 | 8.53E-03 |
| 112 | ENSG00000138646 | HERC5 | -0.32 | 8.71E-03 |
| 113 | ENSG00000262112 | RP11-670E13.5 | -5.08 | 8.83E-03 |
| 114 | ENSG00000270820 | RP11-355B11.2 | -2.00 | 8.84E-03 |
| 115 | ENSG00000122861 | PLAU | -0.25 | 8.93E-03 |
| 116 | ENSG00000258749 | RP11-688G15.3 | -4.91 | 9.39E-03 |
| 117 | ENSG00000164442 | CITED2 | -0.43 | 9.59E-03 |
| 118 | ENSG00000236326 | RP3-486I3.5 | -5.21 | 9.94E-03 |
| 119 | ENSG00000258908 | RP11-203M5.8 | -5.21 | 9.94E-03 |
| 120 | ENSG00000213002 | AC120194.1 | -3.62 | 9.94E-03 |
| 121 | ENSG00000117266 | CDK18 | -0.51 | 9.94E-03 |
| 122 | ENSG00000271581 | XXbac-BPG248L24.12 | -4.93 | 1.01E-02 |
| 123 | ENSG00000245322 | RP11-15B17.1 | -2.68 | 1.01E-02 |
| 124 | ENSG00000258388 | PPT2-EGFL8 | -2.67 | 1.01E-02 |
| 125 | ENSG00000260668 | RP11-744D14.1 | -1.48 | 1.01E-02 |
| 126 | ENSG00000062038 | CDH3 | -1.27 | 1.01E-02 |
| 127 | ENSG00000255050 | RP11-661A12.9 | -5.11 | 1.01E-02 |
| 128 | ENSG00000255857 | PXN-AS1 | -1.05 | 1.01E-02 |
| 129 | ENSG00000100345 | MYH9 | -0.17 | 1.01E-02 |
| 130 | ENSG00000238045 | AC009133.14 | -1.29 | 1.03E-02 |
| 131 | ENSG00000262413 | RP11-498C9.3 | -3.49 | 1.06E-02 |
| 132 | ENSG00000247473 | CARS-AS1 | -4.98 | 1.10E-02 |
| 133 | ENSG00000245748 | RP11-367J11.2 | -4.05 | 1.10E-02 |
| 134 | ENSG00000237676 | RPL30P4 | -0.62 | 1.11E-02 |
| 135 | ENSG00000230733 | AC092171.4 | -5.12 | 1.14E-02 |
| 136 | ENSG00000267607 | CTD-2369P2.8 | -2.13 | 1.14E-02 |
| 137 | ENSG00000095739 | BAMBI | -0.49 | 1.14E-02 |
| 138 | ENSG00000267165 | RP11-78A19.3 | -4.78 | 1.27E-02 |
| 139 | ENSG00000271970 | RP3-337H4.10 | -5.08 | 1.28E-02 |
| 140 | ENSG00000258430 | RP11-982M15.2 | -3.04 | 1.33E-02 |
| 141 | ENSG00000250539 | KRT8P33 | -0.63 | 1.36E-02 |
| 142 | ENSG00000136826 | KLF4 | -0.54 | 1.36E-02 |
| 143 | ENSG00000120949 | TNFRSF8 | -0.82 | 1.37E-02 |
| 144 | ENSG00000236540 | AC006547.13 | -5.06 | 1.37E-02 |
| 145 | ENSG00000008294 | SPAG9 | -0.26 | 1.42E-02 |
| 146 | ENSG00000267532 | MIR497HG | -3.90 | 1.42E-02 |
| 147 | ENSG00000124151 | NCOA3 | -0.37 | 1.45E-02 |
| 148 | ENSG00000235298 | RP11-575L7.8 | -4.67 | 1.48E-02 |
| 149 | ENSG00000167767 | KRT80 | -0.60 | 1.48E-02 |
| 150 | ENSG00000128284 | APOL3 | -0.53 | 1.49E-02 |
| 151 | ENSG00000266341 | RP5-890E16.4 | -2.82 | 1.49E-02 |
| 152 | ENSG00000227543 | SPAG5-AS1 | -1.94 | 1.49E-02 |
| 153 | ENSG00000025708 | TYMP | -0.36 | 1.50E-02 |
| 154 | ENSG00000265618 | CTB-96E2.7 | -5.01 | 1.52E-02 |
| 155 | ENSG00000232729 | AC083884.8 | -3.84 | 1.52E-02 |
| 156 | ENSG00000261460 | RP11-106M3.3 | -1.90 | 1.52E-02 |
| 157 | ENSG00000259298 | RP11-562A8.4 | -4.97 | 1.54E-02 |
| 158 | ENSG00000158828 | PINK1 | -0.42 | 1.54E-02 |
| 159 | ENSG00000171992 | SYNPO | -0.40 | 1.54E-02 |
| 160 | ENSG00000116691 | MIIP | -0.37 | 1.54E-02 |
| 161 | ENSG00000269131 | AC004447.2 | -3.75 | 1.54E-02 |
| 162 | ENSG00000273090 | RP11-78I14.1 | -3.62 | 1.55E-02 |
| 163 | ENSG00000260465 | RP11-63M22.2 | -3.25 | 1.55E-02 |
| 164 | ENSG00000185033 | SEMA4B | -0.31 | 1.62E-02 |
| 165 | ENSG00000236051 | MYCBP2-AS1 | -4.98 | 1.64E-02 |
| 166 | ENSG00000197753 | LHFPL5 | -4.81 | 1.69E-02 |
| 167 | ENSG00000167970 | AC009065.1 | -4.45 | 1.69E-02 |
| 168 | ENSG00000156427 | FGF18 | -1.31 | 1.71E-02 |
| 169 | ENSG00000263165 | RP11-810M2.2 | -4.76 | 1.71E-02 |
| 170 | ENSG00000258745 | RP11-218E20.5 | -4.92 | 1.73E-02 |
| 171 | ENSG00000164916 | FOXK1 | -0.32 | 1.73E-02 |
| 172 | ENSG00000178685 | PARP10 | -0.44 | 1.73E-02 |
| 173 | ENSG00000262160 | RP11-96D1.11 | -2.26 | 1.73E-02 |
| 174 | ENSG00000236338 | AC015987.2 | -4.60 | 1.74E-02 |
| 175 | ENSG00000170271 | FAXDC2 | -5.01 | 1.79E-02 |
| 176 | ENSG00000185689 | C6orf201 | -4.52 | 1.79E-02 |
| 177 | ENSG00000130787 | HIP1R | -0.40 | 1.79E-02 |
| 178 | ENSG00000203394 | RP5-930J4.4 | -3.20 | 1.81E-02 |
| 179 | ENSG00000258377 | RP11-649E7.5 | -1.86 | 1.84E-02 |
| 180 | ENSG00000134326 | CMPK2 | -0.64 | 1.85E-02 |
| 181 | ENSG00000160813 | PPP1R35 | -0.56 | 1.85E-02 |
| 182 | ENSG00000229036 | RP1-20N2.6 | -2.70 | 1.87E-02 |
| 183 | ENSG00000003137 | CYP26B1 | -0.60 | 1.87E-02 |
| 184 | ENSG00000155657 | TTN | -3.68 | 1.88E-02 |
| 185 | ENSG00000258504 | RP11-638I2.6 | -4.85 | 1.93E-02 |
| 186 | ENSG00000272742 | CTB-43P18.1 | -4.50 | 1.93E-02 |
| 187 | ENSG00000259952 | AC009133.15 | -2.87 | 1.93E-02 |
| 188 | ENSG00000137834 | SMAD6 | -0.64 | 2.00E-02 |
| 189 | ENSG00000142599 | RERE | -0.31 | 2.00E-02 |
| 190 | ENSG00000125968 | ID1 | -0.47 | 2.03E-02 |
| 191 | ENSG00000167550 | RHEBL1 | -0.52 | 2.07E-02 |
| 192 | ENSG00000261614 | YBX3P1 | -1.12 | 2.19E-02 |
| 193 | ENSG00000249180 | CTC-506B8.1 | -3.53 | 2.22E-02 |
| 194 | ENSG00000197943 | PLCG2 | -0.40 | 2.26E-02 |
| 195 | ENSG00000269094 | AC006449.1 | -4.84 | 2.35E-02 |
| 196 | ENSG00000256152 | RP11-463O12.3 | -4.54 | 2.35E-02 |
| 197 | ENSG00000167476 | JSRP1 | -3.09 | 2.39E-02 |
| 198 | ENSG00000224985 | RP11-297K8.2 | -4.43 | 2.41E-02 |
| 199 | ENSG00000236782 | RP11-96L14.7 | -3.84 | 2.42E-02 |
| 200 | ENSG00000261641 | LA16c-390E6.5 | -4.47 | 2.43E-02 |
| 201 | ENSG00000267633 | CTB-5E10.3 | -4.56 | 2.46E-02 |
| 202 | ENSG00000111335 | OAS2 | -0.50 | 2.46E-02 |
| 203 | ENSG00000173227 | SYT12 | -0.58 | 2.46E-02 |
| 204 | ENSG00000175920 | DOK7 | -4.26 | 2.47E-02 |
| 205 | ENSG00000260466 | RP4-536B24.2 | -4.87 | 2.50E-02 |
| 206 | ENSG00000115602 | IL1RL1 | -4.76 | 2.50E-02 |
| 207 | ENSG00000245904 | RP11-796E2.4 | -4.76 | 2.50E-02 |
| 208 | ENSG00000215910 | C1orf167 | -4.44 | 2.50E-02 |
| 209 | ENSG00000228748 | RP13-39P12.3 | -4.26 | 2.50E-02 |
| 210 | ENSG00000262211 | CTD-2031P19.5 | -2.90 | 2.50E-02 |
| 211 | ENSG00000188549 | C15orf52 | -0.69 | 2.50E-02 |
| 212 | ENSG00000107731 | UNC5B | -0.55 | 2.50E-02 |
| 213 | ENSG00000228925 | AC016722.4 | -4.73 | 2.50E-02 |
| 214 | ENSG00000164855 | TMEM184A | -2.84 | 2.51E-02 |
| 215 | ENSG00000226047 | RP11-196I18.4 | -4.75 | 2.53E-02 |
| 216 | ENSG00000117461 | PIK3R3 | -0.42 | 2.55E-02 |
| 217 | ENSG00000255959 | RP11-804A23.2 | -4.79 | 2.66E-02 |
| 218 | ENSG00000266993 | RP4-657D16.3 | -3.92 | 2.66E-02 |
| 219 | ENSG00000269604 | AC005523.2 | -3.44 | 2.66E-02 |
| 220 | ENSG00000268015 | CTD-2525I3.3 | -4.68 | 2.67E-02 |
| 221 | ENSG00000197561 | ELANE | -0.61 | 2.76E-02 |
| 222 | ENSG00000230565 | ZNF32-AS2 | -4.67 | 2.78E-02 |
| 223 | ENSG00000273132 | RP11-350J20.12 | -4.48 | 2.78E-02 |
| 224 | ENSG00000267121 | CTD-2020K17.1 | -1.06 | 2.78E-02 |
| 225 | ENSG00000258666 | RP11-638I2.8 | -4.69 | 2.80E-02 |
| 226 | ENSG00000269243 | CTD-2231E14.8 | -2.97 | 2.80E-02 |
| 227 | ENSG00000120738 | EGR1 | -0.63 | 2.83E-02 |
| 228 | ENSG00000007237 | GAS7 | -0.54 | 2.85E-02 |
| 229 | ENSG00000184058 | TBX1 | -0.31 | 2.85E-02 |
| 230 | ENSG00000259081 | RP11-488C13.6 | -4.65 | 2.86E-02 |
| 231 | ENSG00000267601 | RP11-323N12.5 | -4.27 | 2.86E-02 |
| 232 | ENSG00000254452 | RP11-867G23.4 | -3.69 | 2.86E-02 |
| 233 | ENSG00000269926 | RP11-442H21.2 | -1.95 | 2.86E-02 |
| 234 | ENSG00000105559 | PLEKHA4 | -0.68 | 2.86E-02 |
| 235 | ENSG00000118898 | PPL | -0.36 | 2.86E-02 |
| 236 | ENSG00000156875 | HIAT1 | -0.34 | 2.86E-02 |
| 237 | ENSG00000107798 | LIPA | -0.26 | 2.86E-02 |
| 238 | ENSG00000166825 | ANPEP | -0.25 | 2.86E-02 |
| 239 | ENSG00000229431 | RP1-92O14.6 | -4.67 | 2.89E-02 |
| 240 | ENSG00000087253 | LPCAT2 | -0.46 | 2.94E-02 |
| 241 | ENSG00000253174 | RP11-360L9.7 | -4.61 | 2.96E-02 |
| 242 | ENSG00000268047 | AC018766.6 | -4.61 | 2.96E-02 |
| 243 | ENSG00000076356 | PLXNA2 | -0.82 | 2.96E-02 |
| 244 | ENSG00000255467 | RP11-144G7.2 | -4.42 | 3.03E-02 |
| 245 | ENSG00000257553 | RP11-603J24.17 | -2.81 | 3.09E-02 |
| 246 | ENSG00000224888 | RP5-1142A6.2 | -4.27 | 3.10E-02 |
| 247 | ENSG00000176293 | ZNF135 | -4.39 | 3.12E-02 |
| 248 | ENSG00000237080 | EHMT2-AS1 | -4.24 | 3.30E-02 |
| 249 | ENSG00000256364 | RP11-173P15.3 | -2.08 | 3.35E-02 |
| 250 | ENSG00000273489 | RP11-180C16.1 | -3.18 | 3.37E-02 |
| 251 | ENSG00000246889 | AP000487.5 | -2.35 | 3.37E-02 |
| 252 | ENSG00000261684 | RP11-265N6.1 | -4.32 | 3.38E-02 |
| 253 | ENSG00000227198 | C6orf47-AS1 | -4.23 | 3.38E-02 |
| 254 | ENSG00000262967 | RP11-294J22.6 | -4.21 | 3.38E-02 |
| 255 | ENSG00000228677 | TTC3-AS1 | -3.06 | 3.38E-02 |
| 256 | ENSG00000197077 | KIAA1671 | -0.41 | 3.43E-02 |
| 257 | ENSG00000188269 | OR7A5 | -4.53 | 3.46E-02 |
| 258 | ENSG00000233144 | RP11-537A6.9 | -4.17 | 3.46E-02 |
| 259 | ENSG00000260946 | RP11-407G23.3 | -3.21 | 3.46E-02 |
| 260 | ENSG00000223374 | AC005104.3 | -2.37 | 3.46E-02 |
| 261 | ENSG00000184897 | H1FX | -0.34 | 3.46E-02 |
| 262 | ENSG00000173706 | HEG1 | -0.33 | 3.46E-02 |
| 263 | ENSG00000235237 | RP1-151B14.6 | -4.20 | 3.46E-02 |
| 264 | ENSG00000234945 | AC109828.1 | -3.54 | 3.48E-02 |
| 265 | ENSG00000266658 | RNA28S5 | -0.31 | 3.67E-02 |
| 266 | ENSG00000100285 | NEFH | -0.26 | 3.67E-02 |
| 267 | ENSG00000251224 | CNOT10-AS1 | -4.26 | 3.67E-02 |
| 268 | ENSG00000154642 | C21orf91 | -0.47 | 3.75E-02 |
| 269 | ENSG00000164764 | SBSPON | -1.24 | 3.80E-02 |
| 270 | ENSG00000245385 | RP11-334E6.10 | -3.93 | 3.82E-02 |
| 271 | ENSG00000177989 | ODF3B | -0.44 | 3.86E-02 |
| 272 | ENSG00000145901 | TNIP1 | -0.28 | 3.86E-02 |
| 273 | ENSG00000224645 | RP11-126K1.8 | -4.29 | 3.89E-02 |
| 274 | ENSG00000177764 | ZCCHC3 | -2.28 | 3.89E-02 |
| 275 | ENSG00000268059 | AL441883.1 | -2.21 | 3.89E-02 |
| 276 | ENSG00000170262 | MRAP | -4.64 | 3.95E-02 |
| 277 | ENSG00000185338 | SOCS1 | -1.16 | 3.95E-02 |
| 278 | ENSG00000130600 | H19 | -0.52 | 4.13E-02 |
| 279 | ENSG00000228509 | AC006460.2 | -4.45 | 4.14E-02 |
| 280 | ENSG00000254662 | RP11-872D17.4 | -2.93 | 4.14E-02 |
| 281 | ENSG00000204055 | RP11-247A12.2 | -2.61 | 4.14E-02 |
| 282 | ENSG00000263766 | RP11-580I16.2 | -1.95 | 4.14E-02 |
| 283 | ENSG00000133321 | RARRES3 | -0.80 | 4.14E-02 |
| 284 | ENSG00000164626 | KCNK5 | -0.43 | 4.14E-02 |
| 285 | ENSG00000196526 | AFAP1 | -0.33 | 4.14E-02 |
| 286 | ENSG00000197694 | SPTAN1 | -0.19 | 4.14E-02 |
| 287 | ENSG00000234377 | RNF219-AS1 | -3.80 | 4.30E-02 |
| 288 | ENSG00000272345 | RP1-30M3.5 | -1.17 | 4.33E-02 |
| 289 | ENSG00000167106 | FAM102A | -0.36 | 4.35E-02 |
| 290 | ENSG00000124171 | PARD6B | -0.45 | 4.40E-02 |
| 291 | ENSG00000229893 | AC004549.6 | -2.44 | 4.48E-02 |
| 292 | ENSG00000133401 | PDZD2 | -0.53 | 4.65E-02 |
| 293 | ENSG00000203469 | RP5-1113E3.3 | -4.19 | 4.68E-02 |
| 294 | ENSG00000268030 | AC005253.2 | -4.41 | 4.70E-02 |
| 295 | ENSG00000266677 | RP11-258F1.1 | -4.38 | 4.70E-02 |
| 296 | ENSG00000232581 | AC079742.4 | -4.38 | 4.70E-02 |
| 297 | ENSG00000235351 | AC114730.11 | -4.38 | 4.70E-02 |
| 298 | ENSG00000258714 | RP11-998D10.7 | -4.38 | 4.70E-02 |
| 299 | ENSG00000212768 | AC114546.1 | -3.15 | 4.70E-02 |
| 300 | ENSG00000264558 | RP11-138C9.1 | -2.96 | 4.70E-02 |
| 301 | ENSG00000265496 | MIR1539 | -3.91 | 4.75E-02 |
| 302 | ENSG00000267598 | CTC-250I14.6 | -2.84 | 4.75E-02 |
| 303 | ENSG00000135253 | KCP | -2.03 | 4.75E-02 |
| 304 | ENSG00000135002 | RFK | -0.39 | 4.75E-02 |
| 305 | ENSG00000188501 | LCTL | -4.15 | 4.89E-02 |
| 306 | ENSG00000184922 | FMNL1 | -0.42 | 4.99E-02 |
| 307 | ENSG00000141298 | SSH2 | -0.61 | 5.00E-02 |
