# Supplementary material for: Novel metastatic models of esophageal adenocarcinoma derived from FLO-1 cells highlight the importance of E-cadherin in cancer metastasis
Source: Oncotarget. 2016 Nov 16;7(50):83342–58. doi: 10.18632/oncotarget.13391 (PMC5347774; doi:10.18632/oncotarget.13391)
Supplement: Supplementary file 3 [file oncotarget-07-83342-s003.docx]

**Supplementary Table S3.** Significantly (FDR<0.05) upregulated genes in FLO-1^LM^ compared with FLO-1^Par^

| **Rank** | **Ensembl ID** | **Gene name** | **Log_2_(Fold change)** | **FDR p-value** |
| --- | --- | --- | --- | --- |
| 1 | ENSG00000210082 | MT-RNR2 | 0.35 | 5.98E-11 |
| 2 | ENSG00000198763 | MT-ND2 | 0.37 | 5.31E-08 |
| 3 | ENSG00000198786 | MT-ND5 | 0.31 | 5.14E-07 |
| 4 | ENSG00000228253 | MT-ATP8 | 0.93 | 5.55E-07 |
| 5 | ENSG00000198886 | MT-ND4 | 0.31 | 6.86E-07 |
| 6 | ENSG00000198888 | MT-ND1 | 0.32 | 8.07E-07 |
| 7 | ENSG00000198712 | MT-CO2 | 0.28 | 2.22E-06 |
| 8 | ENSG00000137501 | SYTL2 | 0.85 | 6.04E-06 |
| 9 | ENSG00000198938 | MT-CO3 | 0.31 | 1.13E-05 |
| 10 | ENSG00000087086 | FTL | 0.48 | 1.34E-05 |
| 11 | ENSG00000211459 | MT-RNR1 | 0.30 | 1.89E-05 |
| 12 | ENSG00000169604 | ANTXR1 | 0.65 | 2.76E-05 |
| 13 | ENSG00000080824 | HSP90AA1 | 0.27 | 1.00E-04 |
| 14 | ENSG00000161970 | RPL26 | 0.53 | 1.27E-04 |
| 15 | ENSG00000117114 | LPHN2 | 1.53 | 1.46E-04 |
| 16 | ENSG00000087077 | TRIP6 | 1.32 | 1.75E-04 |
| 17 | ENSG00000175745 | NR2F1 | 1.11 | 2.02E-04 |
| 18 | ENSG00000198727 | MT-CYB | 0.20 | 2.06E-04 |
| 19 | ENSG00000198804 | MT-CO1 | 0.29 | 3.15E-04 |
| 20 | ENSG00000125249 | RAP2A | 0.47 | 5.21E-04 |
| 21 | ENSG00000100526 | CDKN3 | 0.73 | 6.48E-04 |
| 22 | ENSG00000197879 | MYO1C | 0.29 | 1.50E-03 |
| 23 | ENSG00000124614 | RPS10 | 0.64 | 1.95E-03 |
| 24 | ENSG00000078053 | AMPH | 2.31 | 2.20E-03 |
| 25 | ENSG00000159200 | RCAN1 | 0.43 | 4.83E-03 |
| 26 | ENSG00000219451 | RPL23P8 | 1.24 | 7.56E-03 |
| 27 | ENSG00000198899 | MT-ATP6 | 0.13 | 9.21E-03 |
| 28 | ENSG00000145569 | FAM105A | 0.97 | 1.01E-02 |
| 29 | ENSG00000111640 | GAPDH | 0.18 | 1.19E-02 |
| 30 | ENSG00000111885 | MAN1A1 | 0.79 | 1.37E-02 |
| 31 | ENSG00000152558 | TMEM123 | 0.24 | 1.56E-02 |
| 32 | ENSG00000141756 | FKBP10 | 0.27 | 1.73E-02 |
| 33 | ENSG00000196230 | TUBB | 0.17 | 1.88E-02 |
| 34 | ENSG00000131018 | SYNE1 | 1.13 | 2.00E-02 |
| 35 | ENSG00000137500 | CCDC90B | 0.53 | 2.03E-02 |
| 36 | ENSG00000128513 | POT1 | 0.53 | 2.38E-02 |
| 37 | ENSG00000138326 | RPS24 | 0.29 | 2.39E-02 |
| 38 | ENSG00000225912 | RP13-258O15.1 | 0.54 | 2.50E-02 |
| 39 | ENSG00000153071 | DAB2 | 2.03 | 2.50E-02 |
| 40 | ENSG00000152894 | PTPRK | 0.82 | 2.51E-02 |
| 41 | ENSG00000105223 | PLD3 | 0.27 | 2.54E-02 |
| 42 | ENSG00000145730 | PAM | 0.34 | 2.57E-02 |
| 43 | ENSG00000226608 | FTLP3 | 0.34 | 2.62E-02 |
| 44 | ENSG00000124207 | CSE1L | 0.27 | 2.78E-02 |
| 45 | ENSG00000216624 | GAPDHP72 | 0.80 | 2.78E-02 |
| 46 | ENSG00000174748 | RPL15 | 0.24 | 2.80E-02 |
| 47 | ENSG00000134440 | NARS | 0.28 | 2.83E-02 |
| 48 | ENSG00000167460 | TPM4 | 0.32 | 2.85E-02 |
| 49 | ENSG00000138778 | CENPE | 0.36 | 2.86E-02 |
| 50 | ENSG00000101773 | RBBP8 | 0.45 | 3.08E-02 |
| 51 | ENSG00000104408 | EIF3E | 0.26 | 3.31E-02 |
| 52 | ENSG00000108691 | CCL2 | 0.29 | 3.33E-02 |
| 53 | ENSG00000263834 | MIR4635 | 4.49 | 3.38E-02 |
| 54 | ENSG00000232555 | AC104088.1 | 4.45 | 3.46E-02 |
| 55 | ENSG00000232070 | TMEM253 | 4.29 | 3.51E-02 |
| 56 | ENSG00000109084 | TMEM97 | 0.44 | 3.59E-02 |
| 57 | ENSG00000241494 | RP11-796G6.1 | 0.81 | 3.59E-02 |
| 58 | ENSG00000167658 | EEF2 | 0.16 | 3.66E-02 |
| 59 | ENSG00000133112 | TPT1 | 0.18 | 3.67E-02 |
| 60 | ENSG00000115963 | RND3 | 0.84 | 3.72E-02 |
| 61 | ENSG00000196549 | MME | 0.68 | 3.89E-02 |
| 62 | ENSG00000158290 | CUL4B | 0.28 | 4.15E-02 |
| 63 | ENSG00000117122 | MFAP2 | 4.40 | 4.22E-02 |
| 64 | ENSG00000196205 | EEF1A1P5 | 0.29 | 4.70E-02 |
| 65 | ENSG00000133302 | ANKRD32 | 0.46 | 4.70E-02 |
| 66 | ENSG00000161016 | RPL8 | 0.20 | 4.70E-02 |
| 67 | ENSG00000074201 | CLNS1A | 0.30 | 4.70E-02 |
| 68 | ENSG00000156414 | TDRD9 | 0.41 | 4.99E-02 |
