# Supplementary material for: Novel metastatic models of esophageal adenocarcinoma derived from FLO-1 cells highlight the importance of E-cadherin in cancer metastasis
Source: Oncotarget. 2016 Nov 16;7(50):83342–58. doi: 10.18632/oncotarget.13391 (PMC5347774; doi:10.18632/oncotarget.13391)
Supplement: Supplementary file 4 [file oncotarget-07-83342-s004.docx]

**Supplementary Table S4.** Enrichment analysis report: Top 200 GO processes for FLO-1^LM^ vs. FLO-1^Par^

| **Rank** | **Networks** | **FDR** | **In data** | **Total** | **Ratio** |
| --- | --- | --- | --- | --- | --- |
| 1 | single-organism cellular process | 7.82E-10 | 192 | 15176 | 0.01 |
| 2 | response to organic substance | 7.62E-09 | 85 | 4290 | 0.02 |
| 3 | response to chemical | 1.93E-08 | 107 | 6278 | 0.02 |
| 4 | in utero embryonic development | 2.21E-08 | 28 | 633 | 0.04 |
| 5 | multicellular organism development | 2.88E-08 | 110 | 6641 | 0.02 |
| 6 | regulation of cell migration | 2.88E-08 | 34 | 951 | 0.04 |
| 7 | response to stress | 3.57E-08 | 91 | 5016 | 0.02 |
| 8 | regulation of cellular component movement | 3.87E-08 | 36 | 1082 | 0.03 |
| 9 | localization | 3.87E-08 | 104 | 6201 | 0.02 |
| 10 | regulation of locomotion | 3.87E-08 | 36 | 1095 | 0.03 |
| 11 | cell differentiation | 3.87E-08 | 88 | 4817 | 0.02 |
| 12 | immune system process | 3.87E-08 | 67 | 3154 | 0.02 |
| 13 | system development | 3.87E-08 | 100 | 5860 | 0.02 |
| 14 | regulation of cell motility | 5.50E-08 | 34 | 1006 | 0.03 |
| 15 | cellular developmental process | 6.93E-08 | 90 | 5060 | 0.02 |
| 16 | developmental process | 1.15E-07 | 117 | 7560 | 0.02 |
| 17 | embryo development | 1.24E-07 | 42 | 1519 | 0.03 |
| 18 | cellular response to chemical stimulus | 1.45E-07 | 74 | 3823 | 0.02 |
| 19 | regulation of localization | 1.90E-07 | 67 | 3303 | 0.02 |
| 20 | regulation of cellular component organization | 2.02E-07 | 64 | 3088 | 0.02 |
| 21 | anatomical structure development | 2.02E-07 | 112 | 7180 | 0.02 |
| 22 | single-organism developmental process | 2.02E-07 | 115 | 7467 | 0.02 |
| 23 | anatomical structure morphogenesis | 2.10E-07 | 66 | 3251 | 0.02 |
| 24 | chordate embryonic development | 2.10E-07 | 32 | 969 | 0.03 |
| 25 | response to hyperoxia | 2.40E-07 | 10 | 70 | 0.14 |
| 26 | response to increased oxygen levels | 2.40E-07 | 10 | 70 | 0.14 |
| 27 | embryo development ending in birth or egg hatching | 2.40E-07 | 32 | 979 | 0.03 |
| 28 | response to external stimulus | 2.70E-07 | 66 | 3284 | 0.02 |
| 29 | response to lipid | 3.06E-07 | 43 | 1658 | 0.03 |
| 30 | response to oxygen-containing compound | 3.08E-07 | 57 | 2625 | 0.02 |
| 31 | single-multicellular organism process | 3.09E-07 | 116 | 7650 | 0.02 |
| 32 | response to organic cyclic compound | 3.26E-07 | 44 | 1732 | 0.03 |
| 33 | response to estrogen | 4.58E-07 | 21 | 453 | 0.05 |
| 34 | animal organ development | 4.67E-07 | 78 | 4305 | 0.02 |
| 35 | immune effector process | 4.70E-07 | 27 | 745 | 0.04 |
| 36 | cell migration | 4.93E-07 | 33 | 1078 | 0.03 |
| 37 | response to inorganic substance | 5.38E-07 | 30 | 913 | 0.03 |
| 38 | response to endogenous stimulus | 5.59E-07 | 57 | 2685 | 0.02 |
| 39 | cellular response to organic substance | 6.45E-07 | 64 | 3226 | 0.02 |
| 40 | negative regulation of biological process | 7.54E-07 | 100 | 6283 | 0.02 |
| 41 | respiratory electron transport chain | 1.11E-06 | 13 | 168 | 0.08 |
| 42 | electron transport chain | 1.25E-06 | 13 | 170 | 0.08 |
| 43 | cellular process | 1.31E-06 | 206 | 18572 | 0.01 |
| 44 | single-organism process | 1.35E-06 | 194 | 16751 | 0.01 |
| 45 | localization of cell | 1.59E-06 | 34 | 1203 | 0.03 |
| 46 | cell motility | 1.59E-06 | 34 | 1203 | 0.03 |
| 47 | cellular component organization | 1.94E-06 | 105 | 6873 | 0.02 |
| 48 | negative regulation of cellular process | 1.94E-06 | 93 | 5776 | 0.02 |
| 49 | endocytosis | 2.34E-06 | 26 | 763 | 0.03 |
| 50 | cellular response to cytokine stimulus | 2.41E-06 | 31 | 1048 | 0.03 |
| 51 | response to stimulus | 2.41E-06 | 144 | 10819 | 0.01 |
| 52 | cellular component organization or biogenesis | 3.18E-06 | 106 | 7037 | 0.02 |
| 53 | defense response to virus | 4.12E-06 | 14 | 227 | 0.06 |
| 54 | blood vessel morphogenesis | 4.45E-06 | 22 | 581 | 0.04 |
| 55 | response to steroid hormone | 5.04E-06 | 29 | 968 | 0.03 |
| 56 | response to acid chemical | 5.65E-06 | 25 | 748 | 0.03 |
| 57 | locomotion | 6.42E-06 | 43 | 1883 | 0.02 |
| 58 | response to abiotic stimulus | 7.11E-06 | 43 | 1891 | 0.02 |
| 59 | regulation of biological quality | 7.90E-06 | 80 | 4811 | 0.02 |
| 60 | response to virus | 7.94E-06 | 17 | 365 | 0.05 |
| 61 | cellular localization | 9.77E-06 | 53 | 2646 | 0.02 |
| 62 | immune response | 9.77E-06 | 45 | 2059 | 0.02 |
| 63 | cellular component morphogenesis | 9.77E-06 | 39 | 1643 | 0.02 |
| 64 | negative regulation of viral genome replication | 1.02E-05 | 8 | 62 | 0.13 |
| 65 | multi-organism process | 1.02E-05 | 64 | 3512 | 0.02 |
| 66 | muscle structure development | 1.30E-05 | 24 | 733 | 0.03 |
| 67 | hematopoietic or lymphoid organ development | 1.38E-05 | 28 | 965 | 0.03 |
| 68 | small molecule metabolic process | 1.45E-05 | 58 | 3072 | 0.02 |
| 69 | cell morphogenesis involved in differentiation | 1.52E-05 | 31 | 1154 | 0.03 |
| 70 | type I interferon signaling pathway | 1.60E-05 | 9 | 91 | 0.10 |
| 71 | response to alcohol | 1.63E-05 | 25 | 801 | 0.03 |
| 72 | cellular response to type I interferon | 1.71E-05 | 9 | 92 | 0.10 |
| 73 | movement of cell or subcellular component | 1.93E-05 | 44 | 2048 | 0.02 |
| 74 | cell development | 2.07E-05 | 50 | 2496 | 0.02 |
| 75 | phagocytosis | 2.07E-05 | 15 | 308 | 0.05 |
| 76 | organophosphate metabolic process | 2.07E-05 | 30 | 1113 | 0.03 |
| 77 | cytokine-mediated signaling pathway | 2.13E-05 | 23 | 704 | 0.03 |
| 78 | nervous system development | 2.29E-05 | 61 | 3366 | 0.02 |
| 79 | blood vessel development | 2.29E-05 | 23 | 708 | 0.03 |
| 80 | response to type I interferon | 2.43E-05 | 9 | 97 | 0.09 |
| 81 | hemopoiesis | 2.79E-05 | 26 | 889 | 0.03 |
| 82 | ATP metabolic process | 2.98E-05 | 13 | 236 | 0.06 |
| 83 | cell morphogenesis | 3.02E-05 | 36 | 1530 | 0.02 |
| 84 | immune system development | 3.29E-05 | 28 | 1019 | 0.03 |
| 85 | regulation of cell adhesion | 3.51E-05 | 27 | 962 | 0.03 |
| 86 | neurogenesis | 3.61E-05 | 49 | 2479 | 0.02 |
| 87 | cellular respiration | 4.49E-05 | 13 | 246 | 0.05 |
| 88 | ribonucleoside monophosphate metabolic process | 5.31E-05 | 14 | 292 | 0.05 |
| 89 | embryonic placenta development | 5.31E-05 | 10 | 139 | 0.07 |
| 90 | response to other organism | 5.52E-05 | 34 | 1439 | 0.02 |
| 91 | vasculature development | 5.60E-05 | 23 | 752 | 0.03 |
| 92 | response to cytokine | 5.60E-05 | 31 | 1243 | 0.02 |
| 93 | response to external biotic stimulus | 5.60E-05 | 34 | 1442 | 0.02 |
| 94 | nucleoside triphosphate metabolic process | 5.64E-05 | 14 | 295 | 0.05 |
| 95 | regulation of anatomical structure size | 5.86E-05 | 22 | 699 | 0.03 |
| 96 | regeneration | 5.88E-05 | 15 | 341 | 0.04 |
| 97 | purine ribonucleoside triphosphate metabolic process | 6.58E-05 | 13 | 257 | 0.05 |
| 98 | macrophage chemotaxis | 6.80E-05 | 5 | 21 | 0.24 |
| 99 | response to estradiol | 6.80E-05 | 14 | 301 | 0.05 |
| 100 | anatomical structure formation involved in morphogenesis | 6.81E-05 | 33 | 1392 | 0.02 |
| 101 | positive regulation of cellular component organization | 7.00E-05 | 36 | 1599 | 0.02 |
| 102 | cellular protein localization | 7.54E-05 | 35 | 1536 | 0.02 |
| 103 | regulation of actin cytoskeleton organization | 7.54E-05 | 16 | 397 | 0.04 |
| 104 | nucleoside monophosphate metabolic process | 8.16E-05 | 14 | 307 | 0.05 |
| 105 | ribonucleoside triphosphate metabolic process | 8.19E-05 | 13 | 264 | 0.05 |
| 106 | purine nucleoside triphosphate metabolic process | 8.46E-05 | 13 | 265 | 0.05 |
| 107 | actin cytoskeleton organization | 8.60E-05 | 19 | 555 | 0.03 |
| 108 | cellular macromolecule localization | 8.64E-05 | 35 | 1549 | 0.02 |
| 109 | organonitrogen compound metabolic process | 9.84E-05 | 47 | 2434 | 0.02 |
| 110 | single-organism cellular localization | 9.99E-05 | 30 | 1223 | 0.02 |
| 111 | response to biotic stimulus | 1.03E-04 | 34 | 1494 | 0.02 |
| 112 | negative regulation of protein complex assembly | 1.05E-04 | 10 | 154 | 0.06 |
| 113 | cell death | 1.05E-04 | 35 | 1567 | 0.02 |
| 114 | regulation of protein targeting | 1.05E-04 | 16 | 411 | 0.04 |
| 115 | innate immune response | 1.05E-04 | 33 | 1430 | 0.02 |
| 116 | establishment of localization | 1.05E-04 | 79 | 5105 | 0.02 |
| 117 | positive regulation of intracellular transport | 1.05E-04 | 18 | 513 | 0.04 |
| 118 | establishment of T cell polarity | 1.05E-04 | 4 | 11 | 0.36 |
| 119 | cell killing | 1.20E-04 | 8 | 92 | 0.09 |
| 120 | regulation of cellular component size | 1.20E-04 | 17 | 467 | 0.04 |
| 121 | leukocyte migration | 1.27E-04 | 15 | 370 | 0.04 |
| 122 | negative regulation of viral life cycle | 1.27E-04 | 9 | 124 | 0.07 |
| 123 | positive regulation of locomotion | 1.28E-04 | 20 | 631 | 0.03 |
| 124 | negative regulation of fibroblast proliferation | 1.28E-04 | 6 | 43 | 0.14 |
| 125 | purine ribonucleoside monophosphate metabolic process | 1.31E-04 | 13 | 280 | 0.05 |
| 126 | regulation of protein processing in phagocytic vesicle | 1.31E-04 | 3 | 4 | 0.75 |
| 127 | positive regulation of protein processing in phagocytic vesicle | 1.31E-04 | 3 | 4 | 0.75 |
| 128 | purine nucleoside monophosphate metabolic process | 1.34E-04 | 13 | 281 | 0.05 |
| 129 | actin filament-based process | 1.34E-04 | 20 | 635 | 0.03 |
| 130 | negative regulation of cell proliferation | 1.39E-04 | 26 | 995 | 0.03 |
| 131 | establishment of lymphocyte polarity | 1.41E-04 | 4 | 12 | 0.33 |
| 132 | negative regulation of locomotion | 1.41E-04 | 15 | 376 | 0.04 |
| 133 | response to metal ion | 1.41E-04 | 19 | 583 | 0.03 |
| 134 | regulation of cytoskeleton organization | 1.42E-04 | 21 | 696 | 0.03 |
| 135 | cellular protein complex assembly | 1.46E-04 | 17 | 478 | 0.04 |
| 136 | cellular response to endogenous stimulus | 1.50E-04 | 40 | 1964 | 0.02 |
| 137 | generation of precursor metabolites and energy | 1.51E-04 | 19 | 587 | 0.03 |
| 138 | regulation of viral genome replication | 1.53E-04 | 8 | 97 | 0.08 |
| 139 | positive regulation of cell migration | 1.53E-04 | 19 | 588 | 0.03 |
| 140 | negative regulation of viral process | 1.73E-04 | 9 | 131 | 0.07 |
| 141 | positive regulation of cytoplasmic transport | 1.82E-04 | 15 | 386 | 0.04 |
| 142 | nucleoside metabolic process | 1.86E-04 | 16 | 437 | 0.04 |
| 143 | neuron migration | 1.92E-04 | 10 | 169 | 0.06 |
| 144 | positive regulation of endocytosis | 1.92E-04 | 11 | 208 | 0.05 |
| 145 | vesicle-mediated transport | 1.93E-04 | 35 | 1628 | 0.02 |
| 146 | regulation of anatomical structure morphogenesis | 1.93E-04 | 31 | 1350 | 0.02 |
| 147 | response to hormone | 1.97E-04 | 38 | 1846 | 0.02 |
| 148 | positive regulation of intracellular protein transport | 2.06E-04 | 14 | 343 | 0.04 |
| 149 | regulation of cellular component biogenesis | 2.06E-04 | 26 | 1025 | 0.03 |
| 150 | cytoskeleton organization | 2.06E-04 | 28 | 1155 | 0.02 |
| 151 | positive regulation of cellular process | 2.21E-04 | 90 | 6241 | 0.01 |
| 152 | angiogenesis | 2.21E-04 | 16 | 446 | 0.04 |
| 153 | positive regulation of cell motility | 2.21E-04 | 19 | 608 | 0.03 |
| 154 | regulation of actin filament depolymerization | 2.21E-04 | 7 | 74 | 0.09 |
| 155 | placenta development | 2.21E-04 | 12 | 255 | 0.05 |
| 156 | programmed cell death | 2.21E-04 | 33 | 1502 | 0.02 |
| 157 | membrane organization | 2.24E-04 | 31 | 1365 | 0.02 |
| 158 | cell surface receptor signaling pathway | 2.37E-04 | 55 | 3188 | 0.02 |
| 159 | regulation of actin filament-based process | 2.40E-04 | 16 | 450 | 0.04 |
| 160 | actin filament capping | 2.45E-04 | 6 | 50 | 0.12 |
| 161 | aging | 2.49E-04 | 18 | 559 | 0.03 |
| 162 | actin cytoskeleton reorganization | 2.50E-04 | 7 | 76 | 0.09 |
| 163 | regulation of immune complex clearance by monocytes and macrophages | 2.50E-04 | 3 | 5 | 0.60 |
| 164 | positive regulation of immune complex clearance by monocytes and macrophages | 2.50E-04 | 3 | 5 | 0.60 |
| 165 | response to ammonium ion | 2.50E-04 | 11 | 217 | 0.05 |
| 166 | protein localization | 2.52E-04 | 44 | 2327 | 0.02 |
| 167 | response to oxygen levels | 2.52E-04 | 19 | 617 | 0.03 |
| 168 | regulation of intracellular transport | 2.70E-04 | 23 | 858 | 0.03 |
| 169 | regulation of cell morphogenesis | 2.76E-04 | 21 | 738 | 0.03 |
| 170 | positive regulation of cellular component movement | 2.77E-04 | 19 | 622 | 0.03 |
| 171 | phosphate-containing compound metabolic process | 2.87E-04 | 47 | 2575 | 0.02 |
| 172 | hepatocyte apoptotic process | 2.90E-04 | 4 | 15 | 0.27 |
| 173 | glycosyl compound metabolic process | 2.90E-04 | 16 | 460 | 0.03 |
| 174 | regulation of cellular localization | 3.02E-04 | 37 | 1821 | 0.02 |
| 175 | ribonucleoside metabolic process | 3.10E-04 | 15 | 411 | 0.04 |
| 176 | defense response | 3.25E-04 | 44 | 2356 | 0.02 |
| 177 | generation of neurons | 3.41E-04 | 44 | 2361 | 0.02 |
| 178 | response to oxidative stress | 3.44E-04 | 18 | 577 | 0.03 |
| 179 | macromolecule localization | 3.44E-04 | 49 | 2756 | 0.02 |
| 180 | apoptotic process | 3.46E-04 | 32 | 1475 | 0.02 |
| 181 | protein localization to membrane | 3.61E-04 | 17 | 524 | 0.03 |
| 182 | negative regulation of multi-organism process | 3.61E-04 | 11 | 228 | 0.05 |
| 183 | interspecies interaction between organisms | 3.66E-04 | 26 | 1072 | 0.02 |
| 184 | symbiosis, encompassing mutualism through parasitism | 3.66E-04 | 26 | 1072 | 0.02 |
| 185 | positive regulation of nitric oxide biosynthetic process | 3.66E-04 | 7 | 82 | 0.09 |
| 186 | negative regulation of actin filament depolymerization | 3.70E-04 | 6 | 55 | 0.11 |
| 187 | positive regulation of nitric oxide metabolic process | 3.93E-04 | 7 | 83 | 0.08 |
| 188 | response to fluid shear stress | 4.05E-04 | 6 | 56 | 0.11 |
| 189 | striated muscle contraction | 4.05E-04 | 9 | 151 | 0.06 |
| 190 | cellular component biogenesis | 4.05E-04 | 51 | 2940 | 0.02 |
| 191 | circulatory system process | 4.05E-04 | 18 | 587 | 0.03 |
| 192 | receptor-mediated endocytosis | 4.34E-04 | 14 | 375 | 0.04 |
| 193 | cellular macromolecular complex assembly | 4.36E-04 | 23 | 892 | 0.03 |
| 194 | establishment of protein localization to organelle | 4.37E-04 | 16 | 480 | 0.03 |
| 195 | cellular component assembly | 4.58E-04 | 48 | 2715 | 0.02 |
| 196 | cellular response to acid chemical | 4.58E-04 | 15 | 429 | 0.03 |
| 197 | negative regulation of cellular component organization | 4.61E-04 | 25 | 1025 | 0.02 |
| 198 | negative regulation of cell death | 4.61E-04 | 31 | 1433 | 0.02 |
| 199 | nucleobase-containing small molecule metabolic process | 5.10E-04 | 20 | 717 | 0.03 |
| 200 | regulation of developmental process | 5.10E-04 | 53 | 3134 | 0.02 |
