# Supplementary material for: Novel metastatic models of esophageal adenocarcinoma derived from FLO-1 cells highlight the importance of E-cadherin in cancer metastasis
Source: Oncotarget. 2016 Nov 16;7(50):83342–58. doi: 10.18632/oncotarget.13391 (PMC5347774; doi:10.18632/oncotarget.13391)
Supplement: Supplementary file 5 [file oncotarget-07-83342-s005.docx]

**Supplementary Table S5.** Differentially (FDR<0.05) expressed genes in *CDH1* low vs. high tumors in GSE19417

| **Rank** | **Gene name** | **Log2(Fold change)** | **FDR p-value** |
| --- | --- | --- | --- |
| 1 | CDH1 | -0.56 | 1.42E-03 |
| 2 | MTO1 | -0.27 | 9.72E-03 |
| 3 | CHST12 | -0.44 | 9.72E-03 |
| 4 | DPY19L1 | -0.46 | 9.72E-03 |
| 5 | CBX4 | -0.42 | 9.72E-03 |
| 6 | TADA3L | 0.31 | 9.72E-03 |
| 7 | TTC7A | -0.25 | 9.72E-03 |
| 8 | TNFRSF21 | -0.32 | 9.72E-03 |
| 9 | ASXL1 | -0.26 | 9.72E-03 |
| 10 | MTF1 | -0.32 | 9.72E-03 |
| 11 | CUX1 | -0.48 | 9.72E-03 |
| 12 | TMEM41A | -0.31 | 9.72E-03 |
| 13 | ZADH2 | -0.36 | 9.72E-03 |
| 14 | PIGG | -0.47 | 9.72E-03 |
| 15 | ITFG1 | -0.36 | 9.72E-03 |
| 16 | FAM108B1 | 0.25 | 9.72E-03 |
| 17 | MBNL1 | -0.96 | 9.72E-03 |
| 18 | KPNA6 | -0.23 | 9.72E-03 |
| 19 | GPR1 | 0.30 | 9.72E-03 |
| 20 | NAGLU | -0.33 | 9.72E-03 |
| 21 | NOMO1 | -0.31 | 9.72E-03 |
| 22 | SDF4 | -0.34 | 9.72E-03 |
| 23 | KRT81 | -0.30 | 9.72E-03 |
| 24 | TTC23 | -0.30 | 9.72E-03 |
| 25 | GPHB5 | 0.33 | 9.72E-03 |
| 26 | TBC1D21 | 0.24 | 9.72E-03 |
| 27 | TMEM127 | -0.39 | 9.72E-03 |
| 28 | SNTB2 | -0.36 | 9.72E-03 |
| 29 | IL11 | 0.22 | 9.72E-03 |
| 30 | SDF4 | -0.32 | 9.72E-03 |
| 31 | C7orf44 | -0.39 | 9.72E-03 |
| 32 | GSTO2 | 0.36 | 9.72E-03 |
| 33 | LRRC15 | 0.19 | 9.72E-03 |
| 34 | ZNF313 | -0.42 | 9.72E-03 |
| 35 | DYNC1H1 | -0.30 | 9.72E-03 |
| 36 | SAFB | -0.24 | 9.72E-03 |
| 37 | TETRAN | -0.45 | 9.72E-03 |
| 38 | NLRP6 | 0.24 | 9.72E-03 |
| 39 | C20orf26 | 0.33 | 9.72E-03 |
| 40 | KRT73 | 0.30 | 9.72E-03 |
| 41 | FLJ43505 | 0.40 | 9.72E-03 |
| 42 | FNBP1 | -0.58 | 9.72E-03 |
| 43 | hCG_26523 | 0.39 | 9.72E-03 |
| 44 | SIRPD | 0.20 | 9.72E-03 |
| 45 | UNKL | -0.37 | 9.72E-03 |
| 46 | SP3 | -0.35 | 9.72E-03 |
| 47 | MRPL28 | -0.30 | 9.72E-03 |
| 48 | ATM | -0.32 | 9.72E-03 |
| 49 | LAMB2 | -0.31 | 9.72E-03 |
| 50 | GPR115 | 0.27 | 9.72E-03 |
| 51 | ANP32B | 0.32 | 9.72E-03 |
| 52 | BSDC1 | -0.25 | 9.72E-03 |
| 53 | SLC12A9 | -0.43 | 9.72E-03 |
| 54 | TNFRSF21 | -0.42 | 9.72E-03 |
| 55 | SCRN1 | -0.30 | 9.72E-03 |
| 56 | GTF2H4 | -0.25 | 9.72E-03 |
| 57 | PDPR | -0.36 | 9.72E-03 |
| 58 | EDARADD | -0.21 | 9.72E-03 |
| 59 | TCF12 | -0.71 | 9.72E-03 |
| 60 | XPO1 | -0.34 | 9.72E-03 |
| 61 | CRIPAK | -0.34 | 9.72E-03 |
| 62 | MYLIP | -0.30 | 9.72E-03 |
| 63 | PPIC | -0.39 | 9.72E-03 |
| 64 | CYBA | -0.34 | 9.72E-03 |
| 65 | LOC728782 | 0.35 | 9.72E-03 |
| 66 | GPRC5B | -0.55 | 9.72E-03 |
| 67 | RPL26 | 0.37 | 9.72E-03 |
| 68 | ARHGEF10 | -0.25 | 9.72E-03 |
| 69 | NAT1 | 0.21 | 9.72E-03 |
| 70 | SRGAP2 | -0.28 | 9.72E-03 |
| 71 | ADIPOR1 | -0.47 | 9.72E-03 |
| 72 | RMND1 | 0.28 | 9.72E-03 |
| 73 | ZNF346 | -0.39 | 9.72E-03 |
| 74 | PRKCZ | -0.41 | 9.72E-03 |
| 75 | LAMP2 | -0.59 | 9.72E-03 |
| 76 | CIAPIN1 | -0.21 | 9.72E-03 |
| 77 | TXNDC4 | -0.39 | 9.72E-03 |
| 78 | TCTN2 | -0.43 | 9.72E-03 |
| 79 | SCG2 | 0.42 | 9.72E-03 |
| 80 | MAN1B1 | -0.31 | 9.72E-03 |
| 81 | MANBA | -0.36 | 9.72E-03 |
| 82 | DTNA | 0.24 | 9.72E-03 |
| 83 | DHX8 | -0.20 | 9.72E-03 |
| 84 | CDY2B | 0.28 | 9.72E-03 |
| 85 | MAN1B1 | -0.31 | 9.72E-03 |
| 86 | ZNF629 | -0.25 | 9.72E-03 |
| 87 | ABCC4 | -0.31 | 9.72E-03 |
| 88 | KIF1B | -0.50 | 9.72E-03 |
| 89 | LGALS3BP | -0.30 | 9.72E-03 |
| 90 | EPB41L3 | -0.33 | 9.72E-03 |
| 91 | PLP2 | -0.20 | 9.72E-03 |
| 92 | LOC149643 | 0.24 | 9.72E-03 |
| 93 | CD151 | -0.35 | 9.72E-03 |
| 94 | ZHX1 | 0.25 | 9.72E-03 |
| 95 | C7orf54 | -0.47 | 9.72E-03 |
| 96 | CC2D1B | -0.21 | 9.72E-03 |
| 97 | ZP1 | 0.39 | 9.72E-03 |
| 98 | RNF12 | -0.32 | 9.74E-03 |
| 99 | LAYN | 0.32 | 9.74E-03 |
| 100 | C20orf3 | -0.41 | 9.74E-03 |
| 101 | OR4A15 | 0.26 | 9.86E-03 |
| 102 | TBC1D25 | -0.27 | 9.86E-03 |
| 103 | ABCG4 | 0.30 | 9.86E-03 |
| 104 | LOC440956 | 0.31 | 9.86E-03 |
| 105 | JMJD4 | -0.20 | 9.86E-03 |
| 106 | LOC387753///RPL21 | 0.31 | 9.86E-03 |
| 107 | ABCC10 | -0.36 | 9.86E-03 |
| 108 | CLSTN1 | -0.38 | 9.86E-03 |
| 109 | GJC1 | -0.31 | 9.86E-03 |
| 110 | OR6Y1 | 0.22 | 9.86E-03 |
| 111 | ENO2 | -0.35 | 9.86E-03 |
| 112 | HIST1H2BK | -0.32 | 9.86E-03 |
| 113 | PNLIPRP3 | 0.26 | 9.86E-03 |
| 114 | LOC202051 | 0.24 | 9.86E-03 |
| 115 | KCNH7 | 0.23 | 9.86E-03 |
| 116 | CUGBP2 | 0.35 | 9.86E-03 |
| 117 | LOC728190 | -0.42 | 9.86E-03 |
| 118 | DRAP1 | -0.21 | 9.86E-03 |
| 119 | PTPRC | -0.28 | 9.86E-03 |
| 120 | MMP2 | -0.42 | 9.86E-03 |
| 121 | USMG5 | 0.33 | 9.86E-03 |
| 122 | KCNQ2 | -0.27 | 9.86E-03 |
| 123 | GALNT6 | -0.29 | 9.86E-03 |
| 124 | MSTP9 | -0.42 | 9.86E-03 |
| 125 | PKM2 | -0.21 | 9.86E-03 |
| 126 | STARD13 | 0.25 | 9.86E-03 |
| 127 | RAD1 | -0.30 | 9.86E-03 |
| 128 | LAMB3 | -0.56 | 9.86E-03 |
| 129 | OR2D2 | 0.34 | 9.86E-03 |
| 130 | KIAA0319L | -0.40 | 9.86E-03 |
| 131 | KRT40 | 0.30 | 9.86E-03 |
| 132 | RAD1 | -0.30 | 9.86E-03 |
| 133 | C4orf21 | 0.26 | 9.86E-03 |
| 134 | PDLIM7 | -0.32 | 9.86E-03 |
| 135 | BRD2 | -0.19 | 9.86E-03 |
| 136 | RBM44///LRRFIP1 | -0.78 | 9.86E-03 |
| 137 | C1orf94 | 0.32 | 9.86E-03 |
| 138 | CADM1 | -0.39 | 9.86E-03 |
| 139 | TXNDC11 | -0.37 | 9.86E-03 |
| 140 | HYOU1 | -0.32 | 9.86E-03 |
| 141 | LRRC8A | -0.41 | 9.86E-03 |
| 142 | FAM46A | -0.34 | 9.86E-03 |
| 143 | HMX3 | 0.66 | 9.86E-03 |
| 144 | HIST1H2BJ | 0.38 | 9.86E-03 |
| 145 | BPTF | -0.52 | 9.86E-03 |
| 146 | RPL21 | 0.33 | 9.86E-03 |
| 147 | HLA-B | -0.45 | 9.86E-03 |
| 148 | HD | -0.31 | 9.86E-03 |
| 149 | FIGLA | 0.36 | 9.86E-03 |
| 150 | TMEM184B | -0.34 | 9.86E-03 |
| 151 | OR3A1 | 0.38 | 9.86E-03 |
| 152 | USP11 | -0.46 | 9.86E-03 |
| 153 | NOTO | 0.25 | 9.86E-03 |
| 154 | DENND2C | 0.75 | 9.86E-03 |
| 155 | NKX2-1 | 0.33 | 9.86E-03 |
| 156 | SSR1 | -0.32 | 9.86E-03 |
| 157 | HSPA1A | -0.37 | 9.86E-03 |
| 158 | STXBP5L | 0.32 | 9.86E-03 |
| 159 | UBIAD1 | -0.31 | 9.86E-03 |
| 160 | MYEOV2 | 0.69 | 9.86E-03 |
| 161 | USP50 | 0.34 | 9.86E-03 |
| 162 | HSD17B6 | -0.30 | 9.86E-03 |
| 163 | NOMO3 | -0.33 | 9.86E-03 |
| 164 | NUBP1 | -0.25 | 9.86E-03 |
| 165 | C1orf27 | -0.37 | 9.86E-03 |
| 166 | PDIA6 | -0.29 | 9.86E-03 |
| 167 | OR4C1P | 0.25 | 9.86E-03 |
| 168 | CDK5R1 | 0.68 | 9.86E-03 |
| 169 | HTR3C | 0.26 | 9.86E-03 |
| 170 | RPS17 | 0.44 | 9.86E-03 |
| 171 | HEPACAM | 0.34 | 9.86E-03 |
| 172 | BRD8 | 0.57 | 9.86E-03 |
| 173 | UQCRB | 0.36 | 9.86E-03 |
| 174 | TACR1 | 0.69 | 9.86E-03 |
| 175 | ARHGEF7 | -0.36 | 9.86E-03 |
| 176 | SEPT14 | 0.77 | 9.86E-03 |
| 177 | ARSD | -0.35 | 9.86E-03 |
| 178 | PRMT5 | -0.36 | 9.86E-03 |
| 179 | ZNF66 | -0.34 | 9.86E-03 |
| 180 | ARL4C | 0.58 | 9.86E-03 |
| 181 | CDY2A | 0.28 | 9.86E-03 |
| 182 | LOC479559 | 0.30 | 9.86E-03 |
| 183 | HP | -0.26 | 9.86E-03 |
| 184 | TAC1 | 0.21 | 9.86E-03 |
| 185 | CHD9 | -0.48 | 9.86E-03 |
| 186 | DDR1 | -0.31 | 9.86E-03 |
| 187 | SNAPC1 | 0.76 | 9.86E-03 |
| 188 | FKBP9 | -0.26 | 9.86E-03 |
| 189 | RPS11 | 0.32 | 9.86E-03 |
| 190 | OR5M10 | 0.34 | 9.87E-03 |
| 191 | TUBB4 | -0.39 | 9.89E-03 |
| 192 | SURF4 | -0.25 | 9.90E-03 |
| 193 | ERAF | 0.26 | 9.90E-03 |
| 194 | EPB41L4B | 0.36 | 9.91E-03 |
| 195 | NOC2L | -0.25 | 9.91E-03 |
| 196 | HTR2C | 0.43 | 9.91E-03 |
| 197 | KCNQ1OT1 | -0.29 | 9.91E-03 |
| 198 | RAB18 | -0.48 | 9.91E-03 |
| 199 | ANKDD1A | -0.38 | 9.91E-03 |
| 200 | UGCGL1 | -0.37 | 9.91E-03 |
| 201 | AADACL2 | 0.36 | 9.91E-03 |
| 202 | CACNA1A | 0.34 | 9.91E-03 |
| 203 | KIAA0090 | -0.33 | 9.91E-03 |
| 204 | CTSD | -0.34 | 9.91E-03 |
| 205 | RHOJ | 0.20 | 9.91E-03 |
| 206 | SLC30A7 | -0.51 | 9.95E-03 |
| 207 | ABCG4 | 0.27 | 9.98E-03 |
| 208 | P4HB | -0.27 | 9.98E-03 |
| 209 | TNNT3 | 0.77 | 9.98E-03 |
| 210 | GAST | 0.81 | 9.98E-03 |
| 211 | TMEM164 | -0.42 | 9.98E-03 |
| 212 | PLEKHH2 | -0.30 | 9.98E-03 |
| 213 | BPY2 | 0.32 | 9.98E-03 |
| 214 | C2orf32 | 0.27 | 9.98E-03 |
| 215 | C1orf166 | -0.41 | 9.98E-03 |
| 216 | SNORD117 | -0.16 | 9.98E-03 |
| 217 | EPB41L4A | 0.43 | 9.98E-03 |
| 218 | SLC4A3 | -0.33 | 9.98E-03 |
| 219 | HIST1H2BB | 0.25 | 9.98E-03 |
| 220 | LAMC2 | -0.56 | 1.01E-02 |
| 221 | DFFA | -0.25 | 1.01E-02 |
| 222 | CCR4 | 0.24 | 1.01E-02 |
| 223 | TMEM109 | -0.27 | 1.01E-02 |
| 224 | LOC641293 | 0.32 | 1.01E-02 |
| 225 | LOC729165 | 0.24 | 1.01E-02 |
| 226 | CLCN3 | -0.31 | 1.01E-02 |
| 227 | METTL9 | -0.48 | 1.01E-02 |
| 228 | SMN1 | -0.33 | 1.01E-02 |
| 229 | FBXO17 | -0.24 | 1.01E-02 |
| 230 | RS1 | 0.25 | 1.02E-02 |
| 231 | 7A5 | 0.30 | 1.02E-02 |
| 232 | GLT25D1 | -0.31 | 1.02E-02 |
| 233 | SPTBN4 | 0.23 | 1.02E-02 |
| 234 | KRTAP19-5 | -0.42 | 1.02E-02 |
| 235 | KIAA1655 | 0.37 | 1.02E-02 |
| 236 | PINX1 | 0.72 | 1.02E-02 |
| 237 | DGCR8 | 0.41 | 1.02E-02 |
| 238 | CCNB1IP1 | 0.27 | 1.02E-02 |
| 239 | GPR56 | -0.38 | 1.02E-02 |
| 240 | RAD9A | -0.34 | 1.02E-02 |
| 241 | CENPL | -0.44 | 1.02E-02 |
| 242 | HLA-B | -0.50 | 1.02E-02 |
| 243 | TMEM104 | -0.25 | 1.02E-02 |
| 244 | KRTAP9-9 | 0.27 | 1.02E-02 |
| 245 | CNOT1 | -0.20 | 1.02E-02 |
| 246 | LOC401463 | 0.30 | 1.02E-02 |
| 247 | LOC728535///LOC653391///LOC653080 | -0.46 | 1.02E-02 |
| 248 | RPL31 | 0.43 | 1.02E-02 |
| 249 | ZNF708 | -0.31 | 1.02E-02 |
| 250 | DDR1 | -0.41 | 1.02E-02 |
| 251 | NOMO2 | -0.32 | 1.02E-02 |
| 252 | TMEM43 | -0.41 | 1.02E-02 |
| 253 | LOC387761 | 0.31 | 1.02E-02 |
| 254 | ZNF549 | -0.26 | 1.02E-02 |
| 255 | C8orf31 | 0.23 | 1.02E-02 |
| 256 | IL1F9 | 0.63 | 1.02E-02 |
| 257 | SDCCAG1 | 0.36 | 1.02E-02 |
| 258 | CSGlcA-T | -0.39 | 1.02E-02 |
| 259 | TRAV20 | 0.31 | 1.02E-02 |
| 260 | INTS10 | 0.23 | 1.02E-02 |
| 261 | LOC729993 | 0.32 | 1.02E-02 |
| 262 | C19orf45 | 0.27 | 1.02E-02 |
| 263 | LOC642924 | 0.23 | 1.02E-02 |
| 264 | SLC30A7 | -0.51 | 1.02E-02 |
| 265 | POLR3A | -0.25 | 1.02E-02 |
| 266 | P2RY13 | 0.26 | 1.02E-02 |
| 267 | PCSK6 | 0.49 | 1.02E-02 |
| 268 | EMX1 | 0.63 | 1.02E-02 |
| 269 | FLJ10357 | -0.60 | 1.02E-02 |
| 270 | CALU | -0.30 | 1.02E-02 |
| 271 | ABCC4 | -0.48 | 1.02E-02 |
| 272 | ASB12 | 0.31 | 1.02E-02 |
| 273 | SYT2 | 0.21 | 1.02E-02 |
| 274 | NDNL2 | -0.47 | 1.02E-02 |
| 275 | LSAMP | 0.55 | 1.02E-02 |
| 276 | RPL34 | 0.36 | 1.02E-02 |
| 277 | C4orf26 | 0.53 | 1.02E-02 |
| 278 | LOC390876 | 0.38 | 1.02E-02 |
| 279 | AMY2A | -0.42 | 1.02E-02 |
| 280 | ARID2 | -0.42 | 1.02E-02 |
| 281 | URG4 | -0.21 | 1.02E-02 |
| 282 | LCE5A | -0.23 | 1.02E-02 |
| 283 | RGS12 | -0.22 | 1.02E-02 |
| 284 | CBX3 | -0.42 | 1.02E-02 |
| 285 | LOC727820 | -0.72 | 1.02E-02 |
| 286 | MYST3 | -0.33 | 1.02E-02 |
| 287 | ORM2 | -0.45 | 1.02E-02 |
| 288 | PRDM5 | 0.35 | 1.02E-02 |
| 289 | LOC151658 | 0.32 | 1.02E-02 |
| 290 | SLC29A3 | 0.23 | 1.02E-02 |
| 291 | IYD | 0.28 | 1.02E-02 |
| 292 | AKT2 | 0.29 | 1.02E-02 |
| 293 | CCDC98 | 0.25 | 1.02E-02 |
| 294 | DKFZp779O175 | 0.22 | 1.02E-02 |
| 295 | MGC40069///TRAV8-3 | 0.33 | 1.03E-02 |
| 296 | VTI1B | 0.22 | 1.03E-02 |
| 297 | ELF4 | -0.28 | 1.03E-02 |
| 298 | LOC400590 | -0.40 | 1.03E-02 |
| 299 | LOC284167///POLRMT | -0.24 | 1.03E-02 |
| 300 | ZNF207 | -0.66 | 1.03E-02 |
| 301 | GRIN2D | 0.49 | 1.03E-02 |
| 302 | CD3D | -0.25 | 1.03E-02 |
| 303 | POR | -0.33 | 1.03E-02 |
| 304 | ZFYVE27 | -0.40 | 1.03E-02 |
| 305 | ERBB3 | -0.38 | 1.03E-02 |
| 306 | SMN1 | -0.32 | 1.03E-02 |
| 307 | TNFRSF10B | -0.29 | 1.03E-02 |
| 308 | MRPL43 | 0.85 | 1.03E-02 |
| 309 | ZNF282 | -0.21 | 1.03E-02 |
| 310 | CSNK2A1P | -0.26 | 1.03E-02 |
| 311 | MIF | -0.26 | 1.03E-02 |
| 312 | GPR148 | 0.25 | 1.03E-02 |
| 313 | GH2 | 0.29 | 1.03E-02 |
| 314 | RAG2 | 0.40 | 1.03E-02 |
| 315 | SYT7 | 0.23 | 1.03E-02 |
| 316 | HCN2 | -0.25 | 1.03E-02 |
| 317 | PTPRF | -0.43 | 1.03E-02 |
| 318 | RIT2 | 0.79 | 1.03E-02 |
| 319 | SMN2 | -0.42 | 1.03E-02 |
| 320 | MED6 | -0.51 | 1.04E-02 |
| 321 | CACNG8 | 0.36 | 1.04E-02 |
| 322 | UHRF1 | -0.26 | 1.04E-02 |
| 323 | ZNF317 | -0.26 | 1.04E-02 |
| 324 | DPM2 | -0.24 | 1.04E-02 |
| 325 | SLC3A1 | 0.47 | 1.04E-02 |
| 326 | OR6N2 | 0.37 | 1.04E-02 |
| 327 | LOC285778 | 0.51 | 1.04E-02 |
| 328 | SLC35E1 | -0.32 | 1.04E-02 |
| 329 | ORM1 | -0.34 | 1.04E-02 |
| 330 | CYP11A1 | 0.29 | 1.04E-02 |
| 331 | C6orf221 | 0.41 | 1.04E-02 |
| 332 | DKFZP434O047 | 0.25 | 1.04E-02 |
| 333 | MGAT1 | -0.34 | 1.04E-02 |
| 334 | RPS17 | 0.31 | 1.04E-02 |
| 335 | IGF2R | -0.39 | 1.04E-02 |
| 336 | TNFRSF10B | -0.29 | 1.04E-02 |
| 337 | PANK4 | -0.27 | 1.04E-02 |
| 338 | CD200R2 | 0.15 | 1.04E-02 |
| 339 | APOC4 | 0.25 | 1.04E-02 |
| 340 | CEP250 | -0.26 | 1.04E-02 |
| 341 | C14orf156 | 0.23 | 1.04E-02 |
| 342 | C18orf33 | 0.30 | 1.04E-02 |
| 343 | DKFZp761E198 | -0.16 | 1.04E-02 |
| 344 | HMGA2 | 0.22 | 1.04E-02 |
| 345 | LOC285957 | 0.44 | 1.04E-02 |
| 346 | OR3A4 | 0.49 | 1.04E-02 |
| 347 | TMED10 | -0.41 | 1.04E-02 |
| 348 | FLJ20674 | 0.17 | 1.04E-02 |
| 349 | DENND4B | -0.27 | 1.04E-02 |
| 350 | DNAH11 | 0.32 | 1.04E-02 |
| 351 | IQCF3 | 0.36 | 1.04E-02 |
| 352 | RPS20 | 0.41 | 1.04E-02 |
| 353 | RIPK5 | -0.39 | 1.04E-02 |
| 354 | OR4K2 | 0.37 | 1.04E-02 |
| 355 | LOC402057 | 0.36 | 1.04E-02 |
| 356 | HIST1H2AA | 0.21 | 1.04E-02 |
| 357 | NDUFC1 | 0.20 | 1.04E-02 |
| 358 | C20orf66 | 0.17 | 1.04E-02 |
| 359 | RPL35 | 0.38 | 1.04E-02 |
| 360 | HDAC4 | -0.31 | 1.04E-02 |
| 361 | ST7L | -0.38 | 1.04E-02 |
| 362 | GPR39 | 0.75 | 1.04E-02 |
| 363 | RP11-679B17.1 | 0.35 | 1.04E-02 |
| 364 | ADCK1 | 0.55 | 1.04E-02 |
| 365 | GLMN | -0.26 | 1.04E-02 |
| 366 | FAM62A | -0.39 | 1.04E-02 |
| 367 | LHFPL2 | -0.18 | 1.04E-02 |
| 368 | TXNDC5 | -0.27 | 1.04E-02 |
| 369 | NTRK1 | 0.30 | 1.04E-02 |
| 370 | OR1L3 | 0.22 | 1.04E-02 |
| 371 | PTCHD3 | 0.27 | 1.04E-02 |
| 372 | DSG2 | -0.30 | 1.04E-02 |
| 373 | LOC348808 | 0.46 | 1.04E-02 |
| 374 | CLPTM1L | -0.20 | 1.04E-02 |
| 375 | C16orf55 | -0.25 | 1.04E-02 |
| 376 | SLC17A1 | 0.33 | 1.04E-02 |
| 377 | TAPBP | -0.35 | 1.04E-02 |
| 378 | DNAH14 | 0.22 | 1.04E-02 |
| 379 | CNTN4 | 0.25 | 1.04E-02 |
| 380 | PGS1 | -0.40 | 1.04E-02 |
| 381 | ARPC1B | -0.34 | 1.04E-02 |
| 382 | MGC14376 | 0.67 | 1.04E-02 |
| 383 | RPS19 | 0.40 | 1.04E-02 |
| 384 | RAET1G | 0.25 | 1.04E-02 |
| 385 | ACTB | -0.34 | 1.04E-02 |
| 386 | LOC154907 | 0.57 | 1.04E-02 |
| 387 | GSTA5 | 0.34 | 1.04E-02 |
| 388 | POU5F1P3 | -0.30 | 1.04E-02 |
| 389 | DMRT2 | 0.32 | 1.04E-02 |
| 390 | PPIB | -0.23 | 1.04E-02 |
| 391 | SERPINA6 | -0.31 | 1.04E-02 |
| 392 | PLXNB1 | -0.29 | 1.04E-02 |
| 393 | EPHB4 | -0.28 | 1.04E-02 |
| 394 | ESAM | -0.49 | 1.04E-02 |
| 395 | IGSF8 | -0.29 | 1.04E-02 |
| 396 | UBE1 | -0.26 | 1.04E-02 |
| 397 | CCDC83 | 0.32 | 1.04E-02 |
| 398 | HRASLS5 | 0.55 | 1.04E-02 |
| 399 | RP11-218C14.6 | 0.74 | 1.04E-02 |
| 400 | LOC285626 | 0.24 | 1.04E-02 |
| 401 | PCDHA2 | 0.24 | 1.04E-02 |
| 402 | BPY2C | 0.36 | 1.04E-02 |
| 403 | SFRS17A | -0.26 | 1.04E-02 |
| 404 | POU5F1P1 | -0.47 | 1.04E-02 |
| 405 | TEP1 | 0.35 | 1.04E-02 |
| 406 | RPL15 | 0.35 | 1.04E-02 |
| 407 | SLC35B2 | -0.38 | 1.04E-02 |
| 408 | B3GAT3 | -0.27 | 1.04E-02 |
| 409 | PDSS1 | 0.45 | 1.04E-02 |
| 410 | PCDHA3 | -0.62 | 1.04E-02 |
| 411 | DDX51 | -0.20 | 1.04E-02 |
| 412 | ICAM2 | -0.26 | 1.04E-02 |
| 413 | GAMT | -0.25 | 1.04E-02 |
| 414 | FOXR1 | 0.71 | 1.04E-02 |
| 415 | TBL2 | -0.26 | 1.04E-02 |
| 416 | LOC51233 | 0.60 | 1.04E-02 |
| 417 | PYY | 0.31 | 1.04E-02 |
| 418 | RPL4 | 0.39 | 1.04E-02 |
| 419 | DHRS4L2 | 0.33 | 1.04E-02 |
| 420 | KCNB2 | 0.32 | 1.04E-02 |
| 421 | SMN2 | -0.40 | 1.04E-02 |
| 422 | FGF13 | 0.28 | 1.04E-02 |
| 423 | GRAMD1A | -0.23 | 1.04E-02 |
| 424 | TPP1 | -0.37 | 1.04E-02 |
| 425 | PKM2 | -0.19 | 1.05E-02 |
| 426 | C9orf5 | -0.42 | 1.05E-02 |
| 427 | ADAMTS12 | 0.29 | 1.05E-02 |
| 428 | VSTM2L | 0.27 | 1.05E-02 |
| 429 | OR2AE1 | 0.28 | 1.05E-02 |
| 430 | MFSD8 | -0.41 | 1.05E-02 |
| 431 | CPNE5 | -0.32 | 1.05E-02 |
| 432 | PAPLN | -0.56 | 1.05E-02 |
| 433 | KIAA0460 | -0.27 | 1.05E-02 |
| 434 | TG | 0.46 | 1.05E-02 |
| 435 | MARS | -0.28 | 1.05E-02 |
| 436 | ISX | 0.46 | 1.05E-02 |
| 437 | CEP350 | -0.44 | 1.05E-02 |
| 438 | LOC285577 | 0.26 | 1.05E-02 |
| 439 | SLC12A7 | -0.41 | 1.05E-02 |
| 440 | ENPP5 | 0.31 | 1.05E-02 |
| 441 | RP1-127L4.6 | 0.60 | 1.05E-02 |
| 442 | SRY | 0.62 | 1.05E-02 |
| 443 | C16orf58 | -0.34 | 1.05E-02 |
| 444 | SMN2 | -0.39 | 1.05E-02 |
| 445 | PBX4 | -0.44 | 1.05E-02 |
| 446 | PPARA | -0.35 | 1.05E-02 |
| 447 | C19orf63 | -0.30 | 1.05E-02 |
| 448 | PLAU | -0.30 | 1.05E-02 |
| 449 | LOC196913 | 0.44 | 1.05E-02 |
| 450 | C19orf10 | -0.26 | 1.05E-02 |
| 451 | CCT8L2 | 0.36 | 1.05E-02 |
| 452 | R3HDML | 0.41 | 1.05E-02 |
| 453 | ATP11A | 0.51 | 1.05E-02 |
| 454 | IPO4 | -0.22 | 1.05E-02 |
| 455 | ATP13A2 | -0.25 | 1.06E-02 |
| 456 | LMAN2 | -0.24 | 1.06E-02 |
| 457 | LOC286059 | 0.36 | 1.06E-02 |
| 458 | F11R | -0.46 | 1.06E-02 |
| 459 | SMN1 | -0.35 | 1.06E-02 |
| 460 | PTTG1IP | -0.26 | 1.06E-02 |
| 461 | HLA-A | -0.54 | 1.06E-02 |
| 462 | SEC61A1 | -0.36 | 1.06E-02 |
| 463 | DIRC2 | -0.57 | 1.06E-02 |
| 464 | LOC285286 | 0.33 | 1.06E-02 |
| 465 | TNFRSF21 | -0.37 | 1.06E-02 |
| 466 | TTLL2 | 0.27 | 1.06E-02 |
| 467 | ASCC3L1 | -0.21 | 1.06E-02 |
| 468 | TMEM2 | -0.46 | 1.06E-02 |
| 469 | ADAM22 | 0.14 | 1.06E-02 |
| 470 | TMEM9 | -0.34 | 1.06E-02 |
| 471 | OR7G3 | 0.43 | 1.06E-02 |
| 472 | CSPG4 | -0.24 | 1.06E-02 |
| 473 | C4orf31 | 0.26 | 1.06E-02 |
| 474 | BPY2B | 0.37 | 1.06E-02 |
| 475 | RGS5 | 0.91 | 1.06E-02 |
| 476 | IPO7 | -0.33 | 1.06E-02 |
| 477 | EMP3 | -0.38 | 1.06E-02 |
| 478 | C22orf15 | 0.38 | 1.06E-02 |
| 479 | PHYHIP | 0.37 | 1.06E-02 |
| 480 | SLC24A4 | 0.21 | 1.06E-02 |
| 481 | SEMA6D | -0.46 | 1.06E-02 |
| 482 | GSG1L | 0.18 | 1.06E-02 |
| 483 | PDE1A | 0.29 | 1.06E-02 |
| 484 | NNAT | 0.56 | 1.06E-02 |
| 485 | SIKE | -0.39 | 1.06E-02 |
| 486 | HIST3H2A | -0.21 | 1.06E-02 |
| 487 | KIAA1328 | 0.21 | 1.06E-02 |
| 488 | KIR3DL2 | 0.29 | 1.06E-02 |
| 489 | PDK1 | -0.22 | 1.06E-02 |
| 490 | DBF4B | -0.36 | 1.06E-02 |
| 491 | TBC1D3C | -0.34 | 1.06E-02 |
| 492 | PPIB | -0.26 | 1.07E-02 |
| 493 | RP11-431O22.2 | 0.70 | 1.07E-02 |
| 494 | LOC440995 | 0.82 | 1.07E-02 |
| 495 | CSN1S1 | 0.30 | 1.07E-02 |
| 496 | ZNF783 | -0.19 | 1.07E-02 |
| 497 | LOC283953 | 0.40 | 1.07E-02 |
| 498 | SLIT1 | 0.28 | 1.07E-02 |
| 499 | BMP10 | 0.52 | 1.07E-02 |
| 500 | GLT8D1 | -0.31 | 1.07E-02 |
| 501 | UGCG | -0.29 | 1.07E-02 |
| 502 | HLA-C | -0.28 | 1.07E-02 |
| 503 | OLFML2A | -0.62 | 1.07E-02 |
| 504 | TNFRSF12A | -0.26 | 1.07E-02 |
| 505 | C14orf122 | -0.18 | 1.07E-02 |
| 506 | QSOX2 | -0.18 | 1.07E-02 |
| 507 | SLC9A5 | -0.27 | 1.07E-02 |
| 508 | MIA3 | 0.22 | 1.07E-02 |
| 509 | GABRG1 | 0.18 | 1.07E-02 |
| 510 | PPTC7 | -0.27 | 1.07E-02 |
| 511 | FLJ32679 | -0.34 | 1.07E-02 |
| 512 | UNC45A | -0.18 | 1.07E-02 |
| 513 | STRAP | 0.47 | 1.07E-02 |
| 514 | PCDHB1 | -0.30 | 1.07E-02 |
| 515 | UNQ1887 | -0.22 | 1.07E-02 |
| 516 | KRTAP9-9 | 0.25 | 1.07E-02 |
| 517 | PTGER4 | 0.18 | 1.07E-02 |
| 518 | CCDC23 | 0.16 | 1.07E-02 |
| 519 | VARS | -0.19 | 1.07E-02 |
| 520 | MAFG | -0.29 | 1.07E-02 |
| 521 | ANKRD13D | -0.31 | 1.07E-02 |
| 522 | SLC9A8 | -0.58 | 1.07E-02 |
| 523 | BLOC1S3 | -0.20 | 1.07E-02 |
| 524 | LMLN | -0.56 | 1.07E-02 |
| 525 | LRRC50 | 0.20 | 1.07E-02 |
| 526 | SMAD5 | -0.35 | 1.07E-02 |
| 527 | SLC25A1 | -0.20 | 1.07E-02 |
| 528 | SPI1 | 0.57 | 1.07E-02 |
| 529 | PRSS2 | -0.27 | 1.07E-02 |
| 530 | ERP29 | -0.32 | 1.07E-02 |
| 531 | CASP14 | 0.51 | 1.07E-02 |
| 532 | CADPS2 | 0.67 | 1.07E-02 |
| 533 | XCL1 | 0.58 | 1.07E-02 |
| 534 | GRIA4 | 0.40 | 1.07E-02 |
| 535 | OR8J3 | 0.62 | 1.07E-02 |
| 536 | PLOD1 | -0.29 | 1.07E-02 |
| 537 | TMEM16K | -0.23 | 1.07E-02 |
| 538 | PGAP1 | -0.67 | 1.07E-02 |
| 539 | ATG2B | 0.37 | 1.07E-02 |
| 540 | WDR91 | -0.27 | 1.07E-02 |
| 541 | LOC645716 | 0.34 | 1.07E-02 |
| 542 | KRTAP4-2 | 0.66 | 1.07E-02 |
| 543 | SMN2 | -0.39 | 1.07E-02 |
| 544 | ZNF747 | 0.44 | 1.07E-02 |
| 545 | NUP160 | 0.37 | 1.07E-02 |
| 546 | PCDHA6 | -0.35 | 1.07E-02 |
| 547 | PKM2 | -0.18 | 1.07E-02 |
| 548 | PACS2 | -0.31 | 1.07E-02 |
| 549 | SLC5A2 | 0.26 | 1.07E-02 |
| 550 | LOC202181 | -0.25 | 1.07E-02 |
| 551 | C3 | -0.43 | 1.07E-02 |
| 552 | ZNF573 | -0.44 | 1.07E-02 |
| 553 | PRDM1 | -0.43 | 1.07E-02 |
| 554 | RPL36AL | 0.25 | 1.07E-02 |
| 555 | DCXR | -0.36 | 1.08E-02 |
| 556 | MAS1 | 0.26 | 1.08E-02 |
| 557 | CD44 | -0.55 | 1.08E-02 |
| 558 | PAR4 | 0.28 | 1.08E-02 |
| 559 | EIF2C2 | -0.28 | 1.08E-02 |
| 560 | DPY19L2P4 | 0.36 | 1.08E-02 |
| 561 | TMEM38A | -0.21 | 1.08E-02 |
| 562 | EBF1 | 0.67 | 1.08E-02 |
| 563 | RAI16 | -0.21 | 1.08E-02 |
| 564 | AOX2 | 0.23 | 1.08E-02 |
| 565 | SMG1 | -0.45 | 1.08E-02 |
| 566 | DOLPP1 | 0.20 | 1.08E-02 |
| 567 | LTA | 0.22 | 1.08E-02 |
| 568 | UNC45B | 0.17 | 1.08E-02 |
| 569 | LOC285762 | 0.44 | 1.08E-02 |
| 570 | KCNN4 | -0.40 | 1.08E-02 |
| 571 | RPL35A | 0.34 | 1.08E-02 |
| 572 | HOXA6 | 0.29 | 1.08E-02 |
| 573 | ACTR1B | -0.42 | 1.08E-02 |
| 574 | RPS12 | 0.48 | 1.08E-02 |
| 575 | LOC730168 | 0.22 | 1.08E-02 |
| 576 | PTOV1 | -0.23 | 1.08E-02 |
| 577 | DIP2B | 0.23 | 1.08E-02 |
| 578 | RBP4 | -0.46 | 1.08E-02 |
| 579 | LYK5 | -0.22 | 1.08E-02 |
| 580 | CD59 | -0.39 | 1.08E-02 |
| 581 | NPEPL1 | -0.23 | 1.08E-02 |
| 582 | RHBDL2 | 0.64 | 1.08E-02 |
| 583 | TLE1 | -0.26 | 1.08E-02 |
| 584 | PPFIBP1 | -0.48 | 1.08E-02 |
| 585 | GAPDH | -0.34 | 1.08E-02 |
| 586 | RPL36A | 0.31 | 1.08E-02 |
| 587 | DNAJB9 | -0.34 | 1.09E-02 |
| 588 | SLC5A5 | 0.34 | 1.09E-02 |
| 589 | AGK | -0.33 | 1.09E-02 |
| 590 | PHKG1 | -0.45 | 1.09E-02 |
| 591 | SEC24C | -0.20 | 1.09E-02 |
| 592 | RPS20 | 0.35 | 1.09E-02 |
| 593 | AP3D1 | -0.20 | 1.09E-02 |
| 594 | DEGS1 | -0.30 | 1.09E-02 |
| 595 | RPS27 | 0.29 | 1.09E-02 |
| 596 | LOC441495 | 0.40 | 1.09E-02 |
| 597 | LOC390998 | 0.27 | 1.09E-02 |
| 598 | C12orf51 | 0.26 | 1.09E-02 |
| 599 | MARCH7 | -0.32 | 1.09E-02 |
| 600 | BYSL | -0.25 | 1.09E-02 |
| 601 | C8orf56 | 0.27 | 1.09E-02 |
| 602 | BRPF3 | -0.19 | 1.09E-02 |
| 603 | P4HB | -0.25 | 1.09E-02 |
| 604 | C10orf124 | 0.38 | 1.09E-02 |
| 605 | TPM3 | 0.33 | 1.10E-02 |
| 606 | LIME1 | -0.28 | 1.10E-02 |
| 607 | C19orf28 | -0.22 | 1.10E-02 |
| 608 | NCOA3 | -0.27 | 1.10E-02 |
| 609 | ABCC10 | -0.31 | 1.10E-02 |
| 610 | LHX4 | 0.26 | 1.10E-02 |
| 611 | NAALADL1 | 0.36 | 1.10E-02 |
| 612 | BCAR3 | 0.27 | 1.10E-02 |
| 613 | MTPN | 0.20 | 1.10E-02 |
| 614 | YY2 | 0.34 | 1.10E-02 |
| 615 | GOSR1 | -0.33 | 1.10E-02 |
| 616 | ERGIC3 | -0.26 | 1.10E-02 |
| 617 | SLC9A10 | 0.28 | 1.10E-02 |
| 618 | ZNF610 | -0.36 | 1.10E-02 |
| 619 | HRNR | 0.41 | 1.10E-02 |
| 620 | DPP7 | -0.24 | 1.11E-02 |
| 621 | ATP6AP2 | -0.28 | 1.11E-02 |
| 622 | WNT5A | -0.35 | 1.11E-02 |
| 623 | COBLL1 | 0.47 | 1.11E-02 |
| 624 | CTRB1 | 0.37 | 1.11E-02 |
| 625 | SLC1A5 | -0.27 | 1.11E-02 |
| 626 | PCYOX1L | -0.30 | 1.11E-02 |
| 627 | LOC151760 | 0.35 | 1.11E-02 |
| 628 | JMJD2C | -0.23 | 1.11E-02 |
| 629 | TEGT | -0.26 | 1.11E-02 |
| 630 | C6orf85 | -0.43 | 1.11E-02 |
| 631 | EN2 | 0.55 | 1.11E-02 |
| 632 | LOC729409 | -0.40 | 1.11E-02 |
| 633 | C14orf148 | 0.25 | 1.11E-02 |
| 634 | SMARCC2 | -0.17 | 1.11E-02 |
| 635 | ZNF630 | 0.57 | 1.11E-02 |
| 636 | LOC339874 | 0.21 | 1.11E-02 |
| 637 | FBXO6 | -0.34 | 1.11E-02 |
| 638 | ARSF | 0.30 | 1.11E-02 |
| 639 | TPD52L3 | 0.52 | 1.11E-02 |
| 640 | B3GAT3 | -0.19 | 1.11E-02 |
| 641 | FLJ41757///ATP6V1E2 | 0.28 | 1.11E-02 |
| 642 | RBBP6 | -0.42 | 1.11E-02 |
| 643 | C14orf173 | 0.28 | 1.11E-02 |
| 644 | LEMD2 | -0.36 | 1.11E-02 |
| 645 | C2orf50 | 0.39 | 1.11E-02 |
| 646 | GGCX | -0.44 | 1.11E-02 |
| 647 | ALPL | 0.34 | 1.11E-02 |
| 648 | SNORD56 | -0.30 | 1.11E-02 |
| 649 | LIN28B | -0.22 | 1.11E-02 |
| 650 | DGKI | 0.38 | 1.11E-02 |
| 651 | HIF1AN | -0.39 | 1.11E-02 |
| 652 | DAGLB | -0.36 | 1.11E-02 |
| 653 | SLC16A5 | -0.41 | 1.11E-02 |
| 654 | LOC55565 | 0.16 | 1.11E-02 |
| 655 | PFKFB2 | 0.24 | 1.11E-02 |
| 656 | PTPN14 | -0.32 | 1.11E-02 |
| 657 | ADCY8 | 0.25 | 1.11E-02 |
| 658 | CCDC42 | 0.48 | 1.11E-02 |
| 659 | CCL19 | 0.42 | 1.11E-02 |
| 660 | OR51B5 | 0.28 | 1.11E-02 |
| 661 | BCAR1 | -0.21 | 1.11E-02 |
| 662 | KDELC2 | 0.57 | 1.11E-02 |
| 663 | SETD8 | -0.37 | 1.11E-02 |
| 664 | ART1 | 0.29 | 1.11E-02 |
| 665 | H1FX | -0.19 | 1.12E-02 |
| 666 | TRIM25 | -0.22 | 1.12E-02 |
| 667 | FLJ20294 | -0.25 | 1.12E-02 |
| 668 | DPP8 | -0.20 | 1.12E-02 |
| 669 | C14orf176 | 0.42 | 1.12E-02 |
| 670 | HIPK4 | 0.27 | 1.12E-02 |
| 671 | GHITM | -0.31 | 1.12E-02 |
| 672 | GRID2 | 0.54 | 1.12E-02 |
| 673 | LOC284422 | -0.36 | 1.12E-02 |
| 674 | OR51S1 | 0.27 | 1.12E-02 |
| 675 | RGPD5 | -0.18 | 1.12E-02 |
| 676 | TNKS1BP1 | -0.29 | 1.12E-02 |
| 677 | UBAP2L | -0.24 | 1.12E-02 |
| 678 | PLAUR | -0.31 | 1.12E-02 |
| 679 | C1orf108 | -0.16 | 1.12E-02 |
| 680 | KCNC2 | 0.36 | 1.12E-02 |
| 681 | TEX12 | 0.17 | 1.12E-02 |
| 682 | FAM92B | 0.34 | 1.12E-02 |
| 683 | PSMD9 | -0.38 | 1.12E-02 |
| 684 | OR13A1 | 0.22 | 1.12E-02 |
| 685 | C2orf7 | 0.52 | 1.12E-02 |
| 686 | SQSTM1 | -0.25 | 1.13E-02 |
| 687 | DACT1 | 0.59 | 1.13E-02 |
| 688 | KCNK17 | 0.26 | 1.13E-02 |
| 689 | PRAMEF1 | 0.25 | 1.13E-02 |
| 690 | FBXL14 | 0.26 | 1.13E-02 |
| 691 | SUPV3L1 | 0.52 | 1.13E-02 |
| 692 | CHST9 | 0.34 | 1.14E-02 |
| 693 | NPIP | -0.30 | 1.14E-02 |
| 694 | ANKRD31 | 0.46 | 1.14E-02 |
| 695 | GJB2 | 0.56 | 1.14E-02 |
| 696 | ANKFY1 | -0.47 | 1.14E-02 |
| 697 | MAD2L2 | -0.26 | 1.14E-02 |
| 698 | GAPVD1 | -0.31 | 1.15E-02 |
| 699 | DOLK | -0.45 | 1.15E-02 |
| 700 | C10orf4 | 0.37 | 1.15E-02 |
| 701 | C8orf45 | 0.55 | 1.15E-02 |
| 702 | GGA2 | -0.29 | 1.15E-02 |
| 703 | ADD2 | 0.23 | 1.15E-02 |
| 704 | ALPP | 0.43 | 1.15E-02 |
| 705 | CAMK1G | 0.36 | 1.15E-02 |
| 706 | SOX14 | 0.70 | 1.15E-02 |
| 707 | CPSF1 | -0.23 | 1.15E-02 |
| 708 | GNAS | -0.46 | 1.15E-02 |
| 709 | NRG1 | 0.45 | 1.15E-02 |
| 710 | SRD5A2L2 | -0.50 | 1.15E-02 |
| 711 | OR13F1 | 0.23 | 1.15E-02 |
| 712 | ZMYM3 | -0.17 | 1.15E-02 |
| 713 | VIT | 0.44 | 1.15E-02 |
| 714 | UNC13D | 0.28 | 1.15E-02 |
| 715 | FAM134A | -0.37 | 1.15E-02 |
| 716 | PERQ1 | -0.20 | 1.15E-02 |
| 717 | UBQLN3 | 0.45 | 1.16E-02 |
| 718 | GLDC | -0.23 | 1.16E-02 |
| 719 | PIAS4 | -0.16 | 1.16E-02 |
| 720 | STUB1 | -0.30 | 1.16E-02 |
| 721 | CNGA2 | 0.33 | 1.16E-02 |
| 722 | CDK6 | 0.31 | 1.16E-02 |
| 723 | OR11A1 | 0.29 | 1.16E-02 |
| 724 | CLONE795723 | 0.24 | 1.16E-02 |
| 725 | NCSTN | -0.21 | 1.16E-02 |
| 726 | LASS5 | -0.33 | 1.16E-02 |
| 727 | FAM39E | -0.22 | 1.16E-02 |
| 728 | PPFIA3 | 0.23 | 1.16E-02 |
| 729 | REL | -0.38 | 1.16E-02 |
| 730 | PLGLB1 | 0.26 | 1.16E-02 |
| 731 | ZNF382 | 0.50 | 1.16E-02 |
| 732 | EIF4G1 | -0.32 | 1.16E-02 |
| 733 | WDFY3 | -0.34 | 1.16E-02 |
| 734 | FLJ39632///DUXAP10 | -0.44 | 1.16E-02 |
| 735 | NRG1 | 0.34 | 1.16E-02 |
| 736 | HMX2 | 0.39 | 1.16E-02 |
| 737 | KDELR2 | -0.38 | 1.16E-02 |
| 738 | LOC284788 | 0.46 | 1.16E-02 |
| 739 | TRIM56 | -0.26 | 1.16E-02 |
| 740 | ZNF345 | 0.25 | 1.16E-02 |
| 741 | KCNE1 | 0.64 | 1.16E-02 |
| 742 | MBNL1 | -0.49 | 1.17E-02 |
| 743 | CYB561 | -0.29 | 1.17E-02 |
| 744 | FLNB | -0.35 | 1.17E-02 |
| 745 | RPLP1 | 0.45 | 1.17E-02 |
| 746 | C11orf9 | -0.30 | 1.17E-02 |
| 747 | HOMEZ | 0.15 | 1.17E-02 |
| 748 | WDR40C | 0.62 | 1.17E-02 |
| 749 | DRP2 | 0.33 | 1.17E-02 |
| 750 | AURKC | -0.21 | 1.17E-02 |
| 751 | CIB4 | 0.20 | 1.17E-02 |
| 752 | C6orf89 | -0.40 | 1.17E-02 |
| 753 | SEMA5A | -0.28 | 1.17E-02 |
| 754 | BEST3 | -0.22 | 1.17E-02 |
| 755 | C17orf61 | 0.16 | 1.17E-02 |
| 756 | HCFC1 | -0.32 | 1.17E-02 |
| 757 | NALCN | 0.19 | 1.17E-02 |
| 758 | IGSF1 | 0.66 | 1.17E-02 |
| 759 | HSP90B1 | -0.38 | 1.17E-02 |
| 760 | FAT | -0.35 | 1.17E-02 |
| 761 | RIMS2 | -0.28 | 1.17E-02 |
| 762 | MGC16275 | 0.17 | 1.17E-02 |
| 763 | PTPN9 | -0.43 | 1.17E-02 |
| 764 | ZNF280A | -0.20 | 1.17E-02 |
| 765 | LOC572558 | 0.28 | 1.17E-02 |
| 766 | MSR1 | 0.15 | 1.17E-02 |
| 767 | CEBPA | 0.24 | 1.18E-02 |
| 768 | KCTD8 | 0.34 | 1.18E-02 |
| 769 | SLC26A2 | -0.43 | 1.18E-02 |
| 770 | LOC127841 | 0.37 | 1.18E-02 |
| 771 | C19orf44 | 0.25 | 1.18E-02 |
| 772 | LOC401431 | 0.20 | 1.18E-02 |
| 773 | POPDC2 | 0.59 | 1.18E-02 |
| 774 | ING5 | -0.28 | 1.18E-02 |
| 775 | OR2B2 | 0.27 | 1.18E-02 |
| 776 | RBMY2FP | 0.33 | 1.18E-02 |
| 777 | LOC730272///FLJ40330 | -0.47 | 1.18E-02 |
| 778 | ROCK1 | 0.28 | 1.18E-02 |
| 779 | ARHGDIA | -0.20 | 1.18E-02 |
| 780 | TSPY1 | 0.31 | 1.18E-02 |
| 781 | SERPINA5 | -0.22 | 1.19E-02 |
| 782 | SCUBE3 | -0.25 | 1.19E-02 |
| 783 | LOC388339 | 0.39 | 1.19E-02 |
| 784 | IFRD2 | -0.21 | 1.19E-02 |
| 785 | EGFL8 | 0.36 | 1.19E-02 |
| 786 | SRGAP1 | -0.26 | 1.19E-02 |
| 787 | GBAP | 0.37 | 1.19E-02 |
| 788 | ST7OT2 | 0.18 | 1.19E-02 |
| 789 | GON4L | -0.36 | 1.19E-02 |
| 790 | WDR6 | -0.23 | 1.19E-02 |
| 791 | SLC44A2 | -0.30 | 1.19E-02 |
| 792 | TRIM10 | 0.35 | 1.19E-02 |
| 793 | SLC35F3 | 0.26 | 1.19E-02 |
| 794 | TSPAN3 | -0.48 | 1.19E-02 |
| 795 | MUM1 | -0.38 | 1.19E-02 |
| 796 | F13B | 0.44 | 1.19E-02 |
| 797 | ABCC6 | 0.29 | 1.19E-02 |
| 798 | PABPC4 | -0.15 | 1.19E-02 |
| 799 | IRF4 | -0.30 | 1.19E-02 |
| 800 | GRIP2 | -0.34 | 1.19E-02 |
| 801 | LOC100101115///TTTY21 | 0.50 | 1.19E-02 |
| 802 | C1orf102 | -0.26 | 1.19E-02 |
| 803 | RANBP9 | -0.38 | 1.19E-02 |
| 804 | hCG_22804///RPL17 | 0.27 | 1.19E-02 |
| 805 | COL12A1 | -0.63 | 1.19E-02 |
| 806 | INHBA | -0.42 | 1.19E-02 |
| 807 | PSAP | -0.26 | 1.19E-02 |
| 808 | OR6A2 | 0.31 | 1.19E-02 |
| 809 | ZMYND8 | -0.31 | 1.19E-02 |
| 810 | CASP8 | 0.37 | 1.19E-02 |
| 811 | XRCC5 | -0.57 | 1.19E-02 |
| 812 | HLA-DQA2 | 0.35 | 1.19E-02 |
| 813 | ASCL2 | 0.26 | 1.19E-02 |
| 814 | RPS24 | 0.35 | 1.19E-02 |
| 815 | TMPRSS11F | 0.37 | 1.19E-02 |
| 816 | ATP9B | 0.29 | 1.19E-02 |
| 817 | BRWD1 | -0.29 | 1.19E-02 |
| 818 | CCKAR | 0.26 | 1.19E-02 |
| 819 | UTP14C | -0.20 | 1.19E-02 |
| 820 | SLC5A2 | 0.64 | 1.19E-02 |
| 821 | OR8B8 | 0.31 | 1.19E-02 |
| 822 | YARS | -0.31 | 1.19E-02 |
| 823 | NKX6-2 | 0.37 | 1.19E-02 |
| 824 | TGM6///TGM5 | 0.53 | 1.19E-02 |
| 825 | TMIE | 0.24 | 1.19E-02 |
| 826 | SERPINA3 | -0.41 | 1.19E-02 |
| 827 | ZIM3 | 0.40 | 1.19E-02 |
| 828 | ARF3 | -0.19 | 1.19E-02 |
| 829 | PRPF18 | -0.21 | 1.19E-02 |
| 830 | UBE2M | -0.21 | 1.19E-02 |
| 831 | PLAT | -0.49 | 1.19E-02 |
| 832 | TGOLN2 | -0.35 | 1.19E-02 |
| 833 | MTMR10 | 0.35 | 1.19E-02 |
| 834 | TGOLN2 | -0.33 | 1.20E-02 |
| 835 | HIST1H3I | 0.59 | 1.20E-02 |
| 836 | MTAP | 0.36 | 1.20E-02 |
| 837 | SLC6A16 | -0.37 | 1.20E-02 |
| 838 | ATRN | -0.40 | 1.20E-02 |
| 839 | C11orf1 | 0.20 | 1.20E-02 |
| 840 | HLA-F | 0.29 | 1.20E-02 |
| 841 | SMAD3 | -0.23 | 1.20E-02 |
| 842 | SEMA4C | -0.24 | 1.20E-02 |
| 843 | PYGB | -0.24 | 1.20E-02 |
| 844 | CROP | -0.38 | 1.20E-02 |
| 845 | MYL7 | 0.21 | 1.20E-02 |
| 846 | KLHL28 | -0.47 | 1.20E-02 |
| 847 | FOXK2 | -0.22 | 1.20E-02 |
| 848 | BIRC8 | 0.31 | 1.20E-02 |
| 849 | RP13-347D8.3 | 0.21 | 1.20E-02 |
| 850 | HERC2P7 | 0.25 | 1.20E-02 |
| 851 | YTHDC2 | 0.22 | 1.20E-02 |
| 852 | TSPY2 | 0.29 | 1.20E-02 |
| 853 | SPO11 | 0.32 | 1.20E-02 |
| 854 | HOXA1 | 0.25 | 1.20E-02 |
| 855 | NOTCH1 | -0.45 | 1.20E-02 |
| 856 | SLC6A17 | 0.33 | 1.20E-02 |
| 857 | TULP2 | 0.16 | 1.20E-02 |
| 858 | CYP2C19 | 0.26 | 1.20E-02 |
| 859 | NANOGP8 | -0.36 | 1.20E-02 |
| 860 | C16orf65 | 0.25 | 1.20E-02 |
| 861 | GLB1 | -0.29 | 1.20E-02 |
| 862 | ZNF192 | 0.63 | 1.20E-02 |
| 863 | TFEC | 0.36 | 1.20E-02 |
| 864 | RP13-102H20.1 | 0.22 | 1.20E-02 |
| 865 | RHOBTB1 | -0.48 | 1.20E-02 |
| 866 | DGAT2L6 | 0.26 | 1.21E-02 |
| 867 | CDCP1 | -0.45 | 1.21E-02 |
| 868 | CSRP3 | 0.28 | 1.21E-02 |
| 869 | CRYBA2 | 0.21 | 1.21E-02 |
| 870 | RPS29 | 0.23 | 1.21E-02 |
| 871 | MAP3K2 | -0.25 | 1.21E-02 |
| 872 | ANKRD28 | -0.33 | 1.21E-02 |
| 873 | PAK3 | -0.21 | 1.21E-02 |
| 874 | KCNJ14 | 0.25 | 1.21E-02 |
| 875 | SIX2 | 0.32 | 1.21E-02 |
| 876 | PRND | 0.14 | 1.21E-02 |
| 877 | CSNK1D | -0.18 | 1.21E-02 |
| 878 | NDUFA10 | -0.38 | 1.21E-02 |
| 879 | MLXIPL | 0.26 | 1.21E-02 |
| 880 | KIAA1704 | -0.32 | 1.21E-02 |
| 881 | KIAA1276 | -0.35 | 1.21E-02 |
| 882 | OR10G8 | 0.44 | 1.21E-02 |
| 883 | KRTAP13-4 | 0.27 | 1.21E-02 |
| 884 | CCDC132 | -0.38 | 1.21E-02 |
| 885 | OR4K17 | 0.30 | 1.21E-02 |
| 886 | PCSK1 | 0.51 | 1.21E-02 |
| 887 | UBE2Q1 | -0.28 | 1.21E-02 |
| 888 | ZNF264 | -0.35 | 1.21E-02 |
| 889 | RP11-114H20.1 | 0.59 | 1.21E-02 |
| 890 | CADM3 | 0.40 | 1.21E-02 |
| 891 | LENG6 | 0.29 | 1.21E-02 |
| 892 | C11orf56 | -0.25 | 1.21E-02 |
| 893 | INTS6 | -0.39 | 1.21E-02 |
| 894 | TMEM24 | -0.33 | 1.21E-02 |
| 895 | FKSG30 | -0.30 | 1.21E-02 |
| 896 | KLHL23 | -0.34 | 1.21E-02 |
| 897 | ABCB9 | -0.31 | 1.21E-02 |
| 898 | LOC390595 | 0.36 | 1.21E-02 |
| 899 | MRS2L | 0.30 | 1.22E-02 |
| 900 | KRTAP1-5 | 0.46 | 1.22E-02 |
| 901 | NXF1 | -0.19 | 1.22E-02 |
| 902 | SNORD47 | -0.36 | 1.22E-02 |
| 903 | KIR2DL3 | 0.57 | 1.23E-02 |
| 904 | PROK1 | 0.16 | 1.23E-02 |
| 905 | RGS7 | 0.34 | 1.23E-02 |
| 906 | LUZP2 | 0.42 | 1.23E-02 |
| 907 | NOL6 | -0.33 | 1.23E-02 |
| 908 | UNC45B | 0.33 | 1.23E-02 |
| 909 | C1orf58 | -0.23 | 1.23E-02 |
| 910 | SDK1 | 0.26 | 1.23E-02 |
| 911 | RFX4 | 0.35 | 1.23E-02 |
| 912 | ANKRD30A | 0.22 | 1.23E-02 |
| 913 | CALCRL | -0.65 | 1.23E-02 |
| 914 | HYDIN | 0.21 | 1.23E-02 |
| 915 | C2orf57 | 0.26 | 1.23E-02 |
| 916 | OPRS1 | -0.19 | 1.23E-02 |
| 917 | PLVAP | -0.55 | 1.23E-02 |
| 918 | PIGU | -0.25 | 1.23E-02 |
| 919 | SERPINA1 | -0.54 | 1.23E-02 |
| 920 | HOXA10 | 0.23 | 1.23E-02 |
| 921 | ZNF697 | -0.36 | 1.23E-02 |
| 922 | MESDC2 | -0.24 | 1.23E-02 |
| 923 | LOC151878 | -0.59 | 1.23E-02 |
| 924 | KCNQ3 | 0.32 | 1.23E-02 |
| 925 | SYNGR2 | -0.24 | 1.24E-02 |
| 926 | LAMA5 | -0.32 | 1.24E-02 |
| 927 | PLXNC1 | -0.38 | 1.24E-02 |
| 928 | NCDN | 0.20 | 1.24E-02 |
| 929 | NUP214 | -0.14 | 1.24E-02 |
| 930 | ZNF7 | -0.24 | 1.24E-02 |
| 931 | MDC1 | -0.22 | 1.24E-02 |
| 932 | SPPL2A | -0.35 | 1.24E-02 |
| 933 | C8orf74 | 0.45 | 1.24E-02 |
| 934 | RBMY1A3P | 0.55 | 1.24E-02 |
| 935 | ECOP | -0.24 | 1.24E-02 |
| 936 | LRRC37B | -0.30 | 1.24E-02 |
| 937 | TPMT | 0.29 | 1.24E-02 |
| 938 | PHOX2A | 0.36 | 1.24E-02 |
| 939 | DEFB126 | 0.54 | 1.24E-02 |
| 940 | CCBE1 | 0.18 | 1.24E-02 |
| 941 | HIST1H2BF | 0.38 | 1.24E-02 |
| 942 | HTN3 | 0.34 | 1.25E-02 |
| 943 | OSTN | 0.41 | 1.25E-02 |
| 944 | FLJ34651 | 0.16 | 1.25E-02 |
| 945 | ELA2 | 0.36 | 1.25E-02 |
| 946 | C9orf14 | 0.34 | 1.25E-02 |
| 947 | OPA3 | -0.20 | 1.25E-02 |
| 948 | PILRB | -0.28 | 1.25E-02 |
| 949 | SKIP | 0.32 | 1.25E-02 |
| 950 | GEMIN5 | -0.17 | 1.25E-02 |
| 951 | ADAM3A | -0.23 | 1.25E-02 |
| 952 | MAG | -0.16 | 1.25E-02 |
| 953 | YEATS2 | -0.56 | 1.25E-02 |
| 954 | MCF2L2 | 0.29 | 1.25E-02 |
| 955 | PRPS1L1 | -0.17 | 1.25E-02 |
| 956 | UBQLNL | 0.32 | 1.25E-02 |
| 957 | RPS6KL1 | -0.17 | 1.25E-02 |
| 958 | LOC145786 | -0.43 | 1.25E-02 |
| 959 | MAN1A1 | -0.38 | 1.25E-02 |
| 960 | SLC35E3 | -0.31 | 1.25E-02 |
| 961 | CDC42EP4 | -0.17 | 1.25E-02 |
| 962 | FLJ45513 | -0.37 | 1.25E-02 |
| 963 | IGFL4 | 0.27 | 1.25E-02 |
| 964 | ABCC3 | -0.49 | 1.25E-02 |
| 965 | ATP6AP1 | -0.28 | 1.25E-02 |
| 966 | BATF3 | -0.37 | 1.25E-02 |
| 967 | KSR1 | 0.33 | 1.25E-02 |
| 968 | CENPL///GAS5 | 0.28 | 1.25E-02 |
| 969 | CDRT1 | 0.27 | 1.25E-02 |
| 970 | MNT | -0.34 | 1.25E-02 |
| 971 | YIF1A | -0.24 | 1.25E-02 |
| 972 | OR4C45 | 0.43 | 1.25E-02 |
| 973 | TARS2 | -0.24 | 1.25E-02 |
| 974 | C11orf55 | 0.59 | 1.25E-02 |
| 975 | SLC2A11 | -0.43 | 1.25E-02 |
| 976 | MOBKL2B | -0.24 | 1.25E-02 |
| 977 | OLA1 | 0.32 | 1.25E-02 |
| 978 | ATAD4 | -0.19 | 1.25E-02 |
| 979 | PRR14 | -0.19 | 1.25E-02 |
| 980 | OR7C2 | 0.31 | 1.25E-02 |
| 981 | SPTBN1 | 0.25 | 1.25E-02 |
| 982 | PSG11 | 0.24 | 1.25E-02 |
| 983 | GNPTG | -0.24 | 1.25E-02 |
| 984 | INSL5 | -0.39 | 1.25E-02 |
| 985 | CYP4A11 | 0.19 | 1.25E-02 |
| 986 | LOC389901 | -0.15 | 1.25E-02 |
| 987 | VPS18 | -0.20 | 1.25E-02 |
| 988 | SF3B3 | -0.28 | 1.25E-02 |
| 989 | BTAF1 | -0.46 | 1.25E-02 |
| 990 | GAS8 | 0.27 | 1.25E-02 |
| 991 | QPCT | -0.35 | 1.25E-02 |
| 992 | ATP13A1 | -0.22 | 1.25E-02 |
| 993 | SSBP2 | 0.23 | 1.25E-02 |
| 994 | HOXB13 | 0.28 | 1.25E-02 |
| 995 | ARL11 | 0.29 | 1.25E-02 |
| 996 | FAM134C | -0.34 | 1.25E-02 |
| 997 | PCDHA12 | -0.56 | 1.25E-02 |
| 998 | SCAMP4 | -0.29 | 1.25E-02 |
| 999 | RP11-144G6.7 | -0.53 | 1.25E-02 |
| 1000 | HNF4G | 0.26 | 1.25E-02 |
| 1001 | SNX22 | -0.19 | 1.25E-02 |
| 1002 | LRRTM1 | 0.20 | 1.25E-02 |
| 1003 | KIAA1394 | 0.23 | 1.25E-02 |
| 1004 | C9orf100 | 0.29 | 1.25E-02 |
| 1005 | CPA6 | 0.24 | 1.25E-02 |
| 1006 | RUFY4 | 0.38 | 1.25E-02 |
| 1007 | TMEM179 | 0.33 | 1.26E-02 |
| 1008 | FGF6 | 0.22 | 1.26E-02 |
| 1009 | KCNT2 | 0.36 | 1.26E-02 |
| 1010 | ATM | -0.27 | 1.26E-02 |
| 1011 | CELSR1 | -0.45 | 1.26E-02 |
| 1012 | BRD8 | -0.23 | 1.26E-02 |
| 1013 | TAS2R1 | 0.22 | 1.26E-02 |
| 1014 | ITGB5 | -0.22 | 1.26E-02 |
| 1015 | APOB48R | 0.27 | 1.26E-02 |
| 1016 | JAG2 | -0.20 | 1.26E-02 |
| 1017 | RPL41 | 0.27 | 1.26E-02 |
| 1018 | F10 | -0.25 | 1.26E-02 |
| 1019 | KIAA0644 | -0.23 | 1.26E-02 |
| 1020 | SELI | -0.30 | 1.26E-02 |
| 1021 | KIAA1211 | 0.30 | 1.26E-02 |
| 1022 | HDC | 0.21 | 1.26E-02 |
| 1023 | LOC284648 | 0.20 | 1.26E-02 |
| 1024 | HSPB3 | 0.20 | 1.26E-02 |
| 1025 | LOC152663 | 0.19 | 1.26E-02 |
| 1026 | NR4A2 | -0.34 | 1.26E-02 |
| 1027 | PRSS12 | 0.27 | 1.27E-02 |
| 1028 | LOC93349 | -0.36 | 1.27E-02 |
| 1029 | CYLC1 | 0.29 | 1.27E-02 |
| 1030 | TM9SF1 | -0.29 | 1.27E-02 |
| 1031 | OPRS1 | -0.28 | 1.27E-02 |
| 1032 | LOC388813 | 0.30 | 1.27E-02 |
| 1033 | RPL17 | 0.36 | 1.27E-02 |
| 1034 | HDGF | -0.22 | 1.27E-02 |
| 1035 | SLC6A11 | 0.28 | 1.27E-02 |
| 1036 | RBPJ | -0.29 | 1.27E-02 |
| 1037 | DNAJC16 | 0.68 | 1.27E-02 |
| 1038 | C11orf16 | 0.25 | 1.27E-02 |
| 1039 | LOC203411 | -0.37 | 1.27E-02 |
| 1040 | MAPK8IP2 | 0.30 | 1.27E-02 |
| 1041 | AQP2 | 0.24 | 1.27E-02 |
| 1042 | JARID1A | -0.27 | 1.28E-02 |
| 1043 | AHSA2 | -0.25 | 1.28E-02 |
| 1044 | LOC253573 | 0.24 | 1.28E-02 |
| 1045 | HUWE1 | -0.27 | 1.28E-02 |
| 1046 | WIT1 | -0.31 | 1.28E-02 |
| 1047 | LOC284454 | -0.43 | 1.28E-02 |
| 1048 | ZNF281 | -0.27 | 1.28E-02 |
| 1049 | RAET1L | -0.22 | 1.28E-02 |
| 1050 | PSMAL | 0.18 | 1.28E-02 |
| 1051 | MIB1 | -0.42 | 1.28E-02 |
| 1052 | ADAMTSL5 | 0.26 | 1.28E-02 |
| 1053 | ANGPTL2 | 0.23 | 1.28E-02 |
| 1054 | RPL41 | 0.35 | 1.28E-02 |
| 1055 | FAM131A | -0.26 | 1.28E-02 |
| 1056 | BMP8A | 0.29 | 1.28E-02 |
| 1057 | KTELC1 | -0.42 | 1.28E-02 |
| 1058 | RFXANK | -0.18 | 1.28E-02 |
| 1059 | SERPINB12 | 0.23 | 1.28E-02 |
| 1060 | SUMF2 | -0.31 | 1.28E-02 |
| 1061 | TMEM70 | 0.25 | 1.28E-02 |
| 1062 | NFATC3 | -0.50 | 1.28E-02 |
| 1063 | C10orf10 | -0.37 | 1.28E-02 |
| 1064 | LEPRE1 | -0.31 | 1.28E-02 |
| 1065 | POR | -0.30 | 1.28E-02 |
| 1066 | hCG_1817208 | 0.25 | 1.28E-02 |
| 1067 | LAMC1 | -0.22 | 1.28E-02 |
| 1068 | CHKB | -0.22 | 1.29E-02 |
| 1069 | CSH2 | 0.34 | 1.29E-02 |
| 1070 | RPS15A | 0.42 | 1.29E-02 |
| 1071 | NCR3 | 0.39 | 1.29E-02 |
| 1072 | PTPLAD1 | -0.39 | 1.29E-02 |
| 1073 | OR51G2 | 0.29 | 1.29E-02 |
| 1074 | KCNK1 | -0.46 | 1.29E-02 |
| 1075 | CDY1 | 0.37 | 1.29E-02 |
| 1076 | SLC39A10 | -0.32 | 1.29E-02 |
| 1077 | RCOR3 | -0.35 | 1.29E-02 |
| 1078 | LOC144481 | 0.29 | 1.29E-02 |
| 1079 | CSF2RA | -0.28 | 1.29E-02 |
| 1080 | AEBP1 | -0.26 | 1.29E-02 |
| 1081 | FRMPD4 | 0.26 | 1.29E-02 |
| 1082 | EIF2C4 | -0.30 | 1.29E-02 |
| 1083 | CD81 | -0.26 | 1.29E-02 |
| 1084 | FKRP | -0.11 | 1.30E-02 |
| 1085 | TTN | 0.54 | 1.30E-02 |
| 1086 | GCNT1 | -0.44 | 1.30E-02 |
| 1087 | EIF3E | 0.28 | 1.30E-02 |
| 1088 | LOC91149 | 0.19 | 1.30E-02 |
| 1089 | ZBED1 | -0.25 | 1.30E-02 |
| 1090 | HYAL2 | -0.26 | 1.30E-02 |
| 1091 | OPN1SW | 0.16 | 1.30E-02 |
| 1092 | ING1 | 0.22 | 1.30E-02 |
| 1093 | FBLN1 | -0.19 | 1.30E-02 |
| 1094 | GRM5 | 0.33 | 1.30E-02 |
| 1095 | LOC619207 | 0.23 | 1.30E-02 |
| 1096 | B4GALT1 | -0.30 | 1.30E-02 |
| 1097 | DFFB | -0.30 | 1.30E-02 |
| 1098 | LRIT1 | 0.38 | 1.30E-02 |
| 1099 | FLJ35816 | 0.49 | 1.30E-02 |
| 1100 | LOC285697 | 0.56 | 1.30E-02 |
| 1101 | KRTAP4-14 | 0.20 | 1.30E-02 |
| 1102 | KRT7 | -0.20 | 1.30E-02 |
| 1103 | PRLR | -0.30 | 1.30E-02 |
| 1104 | GBP6 | 0.55 | 1.30E-02 |
| 1105 | C3orf35 | 0.43 | 1.30E-02 |
| 1106 | LRRC46 | 0.32 | 1.30E-02 |
| 1107 | TMCC1 | -0.23 | 1.30E-02 |
| 1108 | ZFHX3 | -0.45 | 1.30E-02 |
| 1109 | SLC12A7 | -0.38 | 1.30E-02 |
| 1110 | OS9 | -0.34 | 1.30E-02 |
| 1111 | RPS13 | 0.35 | 1.30E-02 |
| 1112 | C10orf57 | 0.40 | 1.30E-02 |
| 1113 | C16orf80 | -0.21 | 1.30E-02 |
| 1114 | SNORA70 | -0.37 | 1.30E-02 |
| 1115 | VPRBP | -0.18 | 1.30E-02 |
| 1116 | PSMD7 | -0.32 | 1.30E-02 |
| 1117 | CPAMD8 | 0.23 | 1.30E-02 |
| 1118 | HRBL | -0.29 | 1.30E-02 |
| 1119 | ZNF324B | 0.17 | 1.30E-02 |
| 1120 | RCAN2 | 0.24 | 1.30E-02 |
| 1121 | BMPR2 | -0.42 | 1.30E-02 |
| 1122 | SLC1A4 | -0.33 | 1.30E-02 |
| 1123 | OR10H4 | 0.20 | 1.30E-02 |
| 1124 | PRRC1 | -0.47 | 1.31E-02 |
| 1125 | HRC | 0.36 | 1.31E-02 |
| 1126 | KRTAP5-2 | 0.24 | 1.31E-02 |
| 1127 | TDRD6 | 0.31 | 1.31E-02 |
| 1128 | TMED10P | -0.42 | 1.31E-02 |
| 1129 | PDGFA | -0.28 | 1.31E-02 |
| 1130 | PLXDC2 | 0.41 | 1.31E-02 |
| 1131 | SELT | -0.39 | 1.31E-02 |
| 1132 | GTF3C1 | -0.14 | 1.31E-02 |
| 1133 | TNRC17 | 0.36 | 1.32E-02 |
| 1134 | C5orf15 | -0.23 | 1.32E-02 |
| 1135 | SYNGR1 | 0.53 | 1.32E-02 |
| 1136 | ABCC4 | -0.29 | 1.32E-02 |
| 1137 | PPP1R16B | -0.30 | 1.32E-02 |
| 1138 | RNF5 | 0.24 | 1.33E-02 |
| 1139 | GP9 | 0.41 | 1.33E-02 |
| 1140 | CD3E | 0.48 | 1.33E-02 |
| 1141 | SFRP1 | -0.35 | 1.33E-02 |
| 1142 | MAST4 | 0.42 | 1.33E-02 |
| 1143 | PCDH1 | -0.33 | 1.33E-02 |
| 1144 | KIAA0194 | -0.20 | 1.33E-02 |
| 1145 | RGS16 | 0.43 | 1.33E-02 |
| 1146 | KRTAP5-11 | 0.21 | 1.33E-02 |
| 1147 | ZMYND8 | -0.27 | 1.33E-02 |
| 1148 | INSRR | 0.55 | 1.33E-02 |
| 1149 | SMYD5 | 0.24 | 1.33E-02 |
| 1150 | LOC645620 | 0.55 | 1.33E-02 |
| 1151 | RPL3 | 0.35 | 1.33E-02 |
| 1152 | BAZ2A | -0.29 | 1.33E-02 |
| 1153 | SLC6A9 | 0.31 | 1.33E-02 |
| 1154 | LOC474358 | 0.58 | 1.33E-02 |
| 1155 | OR56A3 | 0.19 | 1.33E-02 |
| 1156 | HK2 | 0.24 | 1.33E-02 |
| 1157 | MAPT | 0.15 | 1.33E-02 |
| 1158 | HIP1 | -0.20 | 1.33E-02 |
| 1159 | INHBB | -0.47 | 1.33E-02 |
| 1160 | SCAMP3 | -0.26 | 1.33E-02 |
| 1161 | PLA2G4F | 0.27 | 1.33E-02 |
| 1162 | VPS13D | -0.51 | 1.33E-02 |
| 1163 | ADAM2 | 0.27 | 1.33E-02 |
| 1164 | CRHR2 | 0.29 | 1.33E-02 |
| 1165 | EPB42 | 0.19 | 1.33E-02 |
| 1166 | OR2T2 | 0.17 | 1.33E-02 |
| 1167 | TNFRSF19 | -0.35 | 1.33E-02 |
| 1168 | OR10K1 | 0.51 | 1.33E-02 |
| 1169 | PRKG1 | 0.36 | 1.33E-02 |
| 1170 | C1orf55 | -0.41 | 1.33E-02 |
| 1171 | PI4KAP2 | -0.17 | 1.33E-02 |
| 1172 | SORCS3 | 0.35 | 1.33E-02 |
| 1173 | LOC148696 | 0.50 | 1.33E-02 |
| 1174 | C20orf29 | 0.32 | 1.34E-02 |
| 1175 | HCG3 | 0.31 | 1.34E-02 |
| 1176 | CADM1 | 0.18 | 1.34E-02 |
| 1177 | SRPRB | -0.34 | 1.34E-02 |
| 1178 | ONECUT2 | -0.31 | 1.34E-02 |
| 1179 | RP5-1054A22.3 | 0.35 | 1.34E-02 |
| 1180 | ITGB4 | 0.58 | 1.34E-02 |
| 1181 | WWC2 | 0.33 | 1.34E-02 |
| 1182 | GLCE | -0.45 | 1.34E-02 |
| 1183 | NUCB1 | -0.34 | 1.34E-02 |
| 1184 | HIST1H2BA | 0.41 | 1.34E-02 |
| 1185 | UCHL1 | -0.31 | 1.34E-02 |
| 1186 | SYVN1 | -0.32 | 1.34E-02 |
| 1187 | RRAGC | -0.18 | 1.34E-02 |
| 1188 | NTSR2 | 0.26 | 1.34E-02 |
| 1189 | ALS2CR16 | -0.33 | 1.34E-02 |
| 1190 | AQP8 | 0.23 | 1.34E-02 |
| 1191 | RP11-217H1.1 | -0.36 | 1.34E-02 |
| 1192 | LOC729658 | 0.27 | 1.35E-02 |
| 1193 | WDR6 | -0.27 | 1.35E-02 |
| 1194 | C6orf89 | -0.35 | 1.35E-02 |
| 1195 | CACNA1D | 0.26 | 1.35E-02 |
| 1196 | SPTB | 0.40 | 1.35E-02 |
| 1197 | GPR88 | 0.30 | 1.35E-02 |
| 1198 | NRM | -0.24 | 1.35E-02 |
| 1199 | FAM9A | 0.21 | 1.35E-02 |
| 1200 | LATS2 | 0.35 | 1.35E-02 |
| 1201 | RPS25 | 0.32 | 1.35E-02 |
| 1202 | ZMAT3 | -0.28 | 1.35E-02 |
| 1203 | FEV | 0.17 | 1.35E-02 |
| 1204 | OR4M2 | 0.29 | 1.35E-02 |
| 1205 | KPNA1 | 0.28 | 1.35E-02 |
| 1206 | DTNA | 0.53 | 1.35E-02 |
| 1207 | TNFRSF1B | -0.37 | 1.35E-02 |
| 1208 | OR10A6 | 0.25 | 1.35E-02 |
| 1209 | CCDC93 | -0.29 | 1.36E-02 |
| 1210 | PAR5 | 0.15 | 1.36E-02 |
| 1211 | ORAI2 | -0.16 | 1.36E-02 |
| 1212 | PRSS27 | 0.18 | 1.36E-02 |
| 1213 | SLC22A16 | 0.36 | 1.36E-02 |
| 1214 | TNFRSF1B | -0.48 | 1.37E-02 |
| 1215 | RORB | -0.22 | 1.37E-02 |
| 1216 | CDH16 | 0.52 | 1.37E-02 |
| 1217 | EFNA1 | -0.34 | 1.37E-02 |
| 1218 | ADAM29 | 0.20 | 1.37E-02 |
| 1219 | TTC22 | 0.45 | 1.37E-02 |
| 1220 | CCRN4L | 0.14 | 1.37E-02 |
| 1221 | MUC15 | -0.19 | 1.37E-02 |
| 1222 | C2orf57 | 0.21 | 1.37E-02 |
| 1223 | CXCR4 | -0.30 | 1.37E-02 |
| 1224 | CCDC84 | -0.20 | 1.37E-02 |
| 1225 | DUSP14 | -0.22 | 1.37E-02 |
| 1226 | COX15 | -0.29 | 1.37E-02 |
| 1227 | GALNTL2 | -0.21 | 1.37E-02 |
| 1228 | SPATA2 | 0.27 | 1.37E-02 |
| 1229 | MYADM | -0.35 | 1.38E-02 |
| 1230 | ZCCHC11 | -0.47 | 1.38E-02 |
| 1231 | WDR49 | 0.53 | 1.38E-02 |
| 1232 | FNTA | -0.41 | 1.38E-02 |
| 1233 | ZIC4 | 0.45 | 1.38E-02 |
| 1234 | SMPD4 | -0.28 | 1.38E-02 |
| 1235 | ZNF697 | -0.20 | 1.38E-02 |
| 1236 | LFNG | -0.43 | 1.38E-02 |
| 1237 | POLR3H | -0.25 | 1.38E-02 |
| 1238 | PSENEN | -0.18 | 1.38E-02 |
| 1239 | LOC440117 | 0.22 | 1.38E-02 |
| 1240 | LOC400566 | -0.39 | 1.38E-02 |
| 1241 | CTSA | -0.29 | 1.38E-02 |
| 1242 | PAPD4 | -0.38 | 1.38E-02 |
| 1243 | USP9Y | -0.23 | 1.39E-02 |
| 1244 | HIST1H2BL | 0.33 | 1.39E-02 |
| 1245 | WDR6 | -0.30 | 1.39E-02 |
| 1246 | LINGO4 | 0.26 | 1.39E-02 |
| 1247 | OSBPL11 | 0.13 | 1.39E-02 |
| 1248 | POMT1 | -0.23 | 1.39E-02 |
| 1249 | ANK1 | 0.43 | 1.39E-02 |
| 1250 | TSPAN9 | -0.28 | 1.39E-02 |
| 1251 | ASTE1 | 0.31 | 1.39E-02 |
| 1252 | QRICH1 | -0.22 | 1.39E-02 |
| 1253 | PHTF2 | -0.37 | 1.39E-02 |
| 1254 | C20orf116 | -0.23 | 1.39E-02 |
| 1255 | B3GALNT1 | 0.30 | 1.39E-02 |
| 1256 | CYP26A1 | 0.34 | 1.39E-02 |
| 1257 | PHACTR1 | 0.44 | 1.39E-02 |
| 1258 | HP | -0.30 | 1.39E-02 |
| 1259 | OR6T1 | 0.22 | 1.39E-02 |
| 1260 | UNC13B | 0.18 | 1.39E-02 |
| 1261 | KIAA0247 | -0.37 | 1.39E-02 |
| 1262 | VPS13A | 0.34 | 1.39E-02 |
| 1263 | CACNG5 | 0.29 | 1.39E-02 |
| 1264 | CRYZL1 | 0.20 | 1.39E-02 |
| 1265 | FAM78A | -0.29 | 1.39E-02 |
| 1266 | IKIP | -0.38 | 1.39E-02 |
| 1267 | SLC18A2 | 0.17 | 1.39E-02 |
| 1268 | VCPIP1 | 0.24 | 1.39E-02 |
| 1269 | PDIA4 | -0.33 | 1.39E-02 |
| 1270 | KC6 | 0.37 | 1.39E-02 |
| 1271 | LOC339240 | 0.41 | 1.39E-02 |
| 1272 | MESTIT1 | 0.19 | 1.39E-02 |
| 1273 | SRPR | -0.27 | 1.39E-02 |
| 1274 | NEUROD4 | 0.43 | 1.40E-02 |
| 1275 | OR6K3 | 0.33 | 1.40E-02 |
| 1276 | FGF16 | 0.19 | 1.40E-02 |
| 1277 | UCN | -0.20 | 1.40E-02 |
| 1278 | LOC90784 | -0.34 | 1.40E-02 |
| 1279 | SLC9A4 | 0.28 | 1.40E-02 |
| 1280 | SST | 0.42 | 1.40E-02 |
| 1281 | CANT1 | -0.21 | 1.40E-02 |
| 1282 | EFNA1 | -0.37 | 1.40E-02 |
| 1283 | MMP14 | -0.43 | 1.40E-02 |
| 1284 | TMEM30A | -0.34 | 1.40E-02 |
| 1285 | FEM1B | -0.39 | 1.40E-02 |
| 1286 | MANEA | -0.47 | 1.40E-02 |
| 1287 | CRELD2 | -0.21 | 1.40E-02 |
| 1288 | DUX5 | 0.22 | 1.40E-02 |
| 1289 | DLX5 | 0.25 | 1.40E-02 |
| 1290 | RAB18 | -0.43 | 1.40E-02 |
| 1291 | PPP1R15B | -0.24 | 1.41E-02 |
| 1292 | SLCO1C1 | 0.24 | 1.41E-02 |
| 1293 | CTSH | -0.38 | 1.41E-02 |
| 1294 | KLK8 | 0.20 | 1.41E-02 |
| 1295 | RPS27A | 0.31 | 1.41E-02 |
| 1296 | LOC286150 | 0.14 | 1.41E-02 |
| 1297 | LOC286260 | -0.20 | 1.41E-02 |
| 1298 | LRRC8B | -0.24 | 1.41E-02 |
| 1299 | RPL7 | 0.35 | 1.41E-02 |
| 1300 | PON2 | -0.27 | 1.41E-02 |
| 1301 | LENG10 | -0.26 | 1.41E-02 |
| 1302 | DOM3Z | -0.26 | 1.41E-02 |
| 1303 | SLMAP | -0.42 | 1.41E-02 |
| 1304 | FLJ10213 | -0.38 | 1.41E-02 |
| 1305 | SPACA4 | 0.40 | 1.41E-02 |
| 1306 | SHC4 | -0.26 | 1.41E-02 |
| 1307 | FLJ35409 | 0.32 | 1.41E-02 |
| 1308 | ENO1P | -0.18 | 1.42E-02 |
| 1309 | TOR3A | -0.25 | 1.42E-02 |
| 1310 | MORC1 | 0.26 | 1.42E-02 |
| 1311 | TRY1 | 0.24 | 1.42E-02 |
| 1312 | FLJ42957 | 0.26 | 1.42E-02 |
| 1313 | FST | -0.23 | 1.42E-02 |
| 1314 | CARD8 | -0.31 | 1.43E-02 |
| 1315 | LPP | -0.44 | 1.43E-02 |
| 1316 | SLC22A8 | 0.49 | 1.43E-02 |
| 1317 | ADAMTS17 | 0.36 | 1.43E-02 |
| 1318 | C15orf2 | 0.50 | 1.43E-02 |
| 1319 | UCA1 | 0.32 | 1.43E-02 |
| 1320 | GORASP2 | -0.23 | 1.43E-02 |
| 1321 | TMEM87A | -0.26 | 1.43E-02 |
| 1322 | TACR3 | 0.29 | 1.43E-02 |
| 1323 | LOC729026 | 0.16 | 1.43E-02 |
| 1324 | UNC84B | -0.32 | 1.43E-02 |
| 1325 | TNRC6B | -0.36 | 1.44E-02 |
| 1326 | DEF6 | -0.23 | 1.44E-02 |
| 1327 | ADAM12 | -0.43 | 1.44E-02 |
| 1328 | NPY | 0.37 | 1.44E-02 |
| 1329 | GAD1 | -0.39 | 1.44E-02 |
| 1330 | KRTCAP2 | -0.22 | 1.44E-02 |
| 1331 | RPL12 | 0.35 | 1.44E-02 |
| 1332 | CCRL1 | 0.21 | 1.44E-02 |
| 1333 | CYP4F11 | 0.41 | 1.44E-02 |
| 1334 | LOC388553 | 0.33 | 1.44E-02 |
| 1335 | CTAGEP | -0.26 | 1.44E-02 |
| 1336 | C21orf29 | 0.23 | 1.44E-02 |
| 1337 | TIMP1 | -0.37 | 1.44E-02 |
| 1338 | VDR | 0.25 | 1.44E-02 |
| 1339 | LOC152667 | -0.18 | 1.44E-02 |
| 1340 | LOC340074 | 0.29 | 1.44E-02 |
| 1341 | ABCF2 | -0.22 | 1.44E-02 |
| 1342 | ITPR2 | 0.35 | 1.44E-02 |
| 1343 | RP3-402G11.5 | -0.17 | 1.44E-02 |
| 1344 | POM121 | -0.19 | 1.44E-02 |
| 1345 | UBR4 | 0.21 | 1.44E-02 |
| 1346 | NCOR2 | -0.22 | 1.44E-02 |
| 1347 | ECSM2 | 0.32 | 1.44E-02 |
| 1348 | DNASE2B | 0.37 | 1.44E-02 |
| 1349 | MMRN1 | 0.23 | 1.44E-02 |
| 1350 | ALKBH1 | -0.16 | 1.44E-02 |
| 1351 | PCGF5 | 0.37 | 1.44E-02 |
| 1352 | ODZ1 | 0.15 | 1.44E-02 |
| 1353 | ADAM10 | 0.30 | 1.44E-02 |
| 1354 | OR5B21 | 0.21 | 1.44E-02 |
| 1355 | SNORD52 | -0.40 | 1.44E-02 |
| 1356 | AMICA1 | 0.15 | 1.45E-02 |
| 1357 | NOL9 | -0.25 | 1.45E-02 |
| 1358 | SLC5A11 | 0.38 | 1.45E-02 |
| 1359 | AMHR2 | 0.43 | 1.46E-02 |
| 1360 | TNNC1 | 0.14 | 1.46E-02 |
| 1361 | MKLN1 | 0.34 | 1.46E-02 |
| 1362 | DCHS2 | -0.38 | 1.46E-02 |
| 1363 | SLC35E2 | -0.24 | 1.46E-02 |
| 1364 | TUBA1A | -0.20 | 1.47E-02 |
| 1365 | CREBBP | -0.25 | 1.47E-02 |
| 1366 | PIGT | -0.31 | 1.47E-02 |
| 1367 | LOC728769 | -0.28 | 1.47E-02 |
| 1368 | SLC5A5 | 0.29 | 1.47E-02 |
| 1369 | SIL1 | -0.27 | 1.47E-02 |
| 1370 | MPHOSPH1 | -0.34 | 1.47E-02 |
| 1371 | SOS1 | -0.56 | 1.47E-02 |
| 1372 | C20orf123 | 0.26 | 1.47E-02 |
| 1373 | SCGB1C1 | 0.15 | 1.47E-02 |
| 1374 | ZNF346 | 0.16 | 1.47E-02 |
| 1375 | ACSBG1 | 0.14 | 1.47E-02 |
| 1376 | PSG1 | 0.57 | 1.47E-02 |
| 1377 | SUCLA2 | 0.19 | 1.47E-02 |
| 1378 | EGF | 0.29 | 1.48E-02 |
| 1379 | ATP1B4 | 0.36 | 1.48E-02 |
| 1380 | TBR1 | 0.32 | 1.48E-02 |
| 1381 | DAG1 | -0.27 | 1.48E-02 |
| 1382 | HAVCR2 | 0.46 | 1.48E-02 |
| 1383 | RPLP2 | 0.27 | 1.48E-02 |
| 1384 | STARD5 | 0.18 | 1.48E-02 |
| 1385 | ZNF579 | 0.30 | 1.48E-02 |
| 1386 | NCAPG2 | -0.30 | 1.48E-02 |
| 1387 | PON2 | -0.30 | 1.48E-02 |
| 1388 | IRF2BP2 | -0.47 | 1.48E-02 |
| 1389 | CLCN6 | -0.22 | 1.48E-02 |
| 1390 | tcag7.1017 | -0.21 | 1.48E-02 |
| 1391 | PCDHGC5 | 0.26 | 1.48E-02 |
| 1392 | KCNK6 | -0.15 | 1.49E-02 |
| 1393 | AMACR | 0.23 | 1.49E-02 |
| 1394 | hCG_2015956 | 0.33 | 1.49E-02 |
| 1395 | WDR77 | 0.21 | 1.49E-02 |
| 1396 | LOC729738 | 0.30 | 1.49E-02 |
| 1397 | RELA | -0.16 | 1.49E-02 |
| 1398 | C1QTNF4 | -0.26 | 1.50E-02 |
| 1399 | SEMA4C | 0.54 | 1.50E-02 |
| 1400 | SLCO3A1 | -0.38 | 1.50E-02 |
| 1401 | C20orf70 | 0.40 | 1.50E-02 |
| 1402 | FLJ20254 | -0.32 | 1.50E-02 |
| 1403 | AHNAK | -0.30 | 1.50E-02 |
| 1404 | C14orf101 | -0.28 | 1.50E-02 |
| 1405 | LRRC51 | -0.31 | 1.50E-02 |
| 1406 | TEX264 | -0.32 | 1.50E-02 |
| 1407 | LSS | -0.19 | 1.50E-02 |
| 1408 | AKR1A1 | 0.25 | 1.50E-02 |
| 1409 | SYT5 | 0.33 | 1.50E-02 |
| 1410 | C2CD2 | -0.27 | 1.50E-02 |
| 1411 | LOC401561 | 0.62 | 1.50E-02 |
| 1412 | BAT5 | -0.25 | 1.50E-02 |
| 1413 | C14orf121 | 0.33 | 1.50E-02 |
| 1414 | TOP1MT | 0.15 | 1.50E-02 |
| 1415 | EMB | 0.19 | 1.50E-02 |
| 1416 | JMJD2C | -0.52 | 1.50E-02 |
| 1417 | VPS8 | -0.26 | 1.51E-02 |
| 1418 | SLC13A5 | -0.17 | 1.51E-02 |
| 1419 | C9orf84 | 0.41 | 1.51E-02 |
| 1420 | LOC285431 | -0.25 | 1.51E-02 |
| 1421 | ZNF718///ZNF595 | 0.19 | 1.51E-02 |
| 1422 | PROL1 | 0.22 | 1.51E-02 |
| 1423 | MRPL37 | 0.32 | 1.51E-02 |
| 1424 | SMAD1 | -0.38 | 1.51E-02 |
| 1425 | LOC150739 | -0.37 | 1.51E-02 |
| 1426 | SPAG17 | 0.26 | 1.51E-02 |
| 1427 | LOC116236 | -0.38 | 1.51E-02 |
| 1428 | PRSS21 | -0.18 | 1.51E-02 |
| 1429 | ATG2A | -0.26 | 1.51E-02 |
| 1430 | CALR | -0.29 | 1.51E-02 |
| 1431 | PHACS | -0.30 | 1.51E-02 |
| 1432 | ATG16L1 | -0.36 | 1.51E-02 |
| 1433 | C7orf53 | -0.42 | 1.51E-02 |
| 1434 | SIN3B | -0.14 | 1.51E-02 |
| 1435 | OTUD3 | -0.29 | 1.51E-02 |
| 1436 | FBXO9 | 0.26 | 1.51E-02 |
| 1437 | HYAL4 | 0.25 | 1.51E-02 |
| 1438 | PDIA5 | -0.38 | 1.52E-02 |
| 1439 | DHX30 | -0.21 | 1.52E-02 |
| 1440 | LOC729358///LOC440416///LOC339260 | 0.33 | 1.52E-02 |
| 1441 | KRT223P | 0.35 | 1.52E-02 |
| 1442 | EFHD2 | -0.29 | 1.52E-02 |
| 1443 | FLJ43752 | 0.42 | 1.52E-02 |
| 1444 | CASK | -0.46 | 1.52E-02 |
| 1445 | VPS41 | -0.31 | 1.52E-02 |
| 1446 | ANKMY1 | 0.26 | 1.52E-02 |
| 1447 | SEMA3B | -0.25 | 1.52E-02 |
| 1448 | SNORD43 | -0.31 | 1.52E-02 |
| 1449 | TMEM63A | -0.39 | 1.52E-02 |
| 1450 | MPZ | 0.28 | 1.52E-02 |
| 1451 | EDG3 | 0.27 | 1.52E-02 |
| 1452 | RXRB | -0.23 | 1.52E-02 |
| 1453 | CYP20A1 | -0.33 | 1.52E-02 |
| 1454 | IL10RB | -0.36 | 1.52E-02 |
| 1455 | CSMD3 | 0.22 | 1.52E-02 |
| 1456 | ASCL3 | 0.28 | 1.52E-02 |
| 1457 | CALU | -0.57 | 1.52E-02 |
| 1458 | CDH13 | -0.34 | 1.52E-02 |
| 1459 | CD37 | -0.19 | 1.52E-02 |
| 1460 | USP44 | -0.14 | 1.52E-02 |
| 1461 | OR56B4 | 0.15 | 1.52E-02 |
| 1462 | ACTN1 | -0.32 | 1.52E-02 |
| 1463 | RAB7A | -0.19 | 1.52E-02 |
| 1464 | SLC12A4 | -0.31 | 1.52E-02 |
| 1465 | KRT34 | 0.26 | 1.52E-02 |
| 1466 | MAST1 | 0.22 | 1.52E-02 |
| 1467 | ECOP | -0.21 | 1.52E-02 |
| 1468 | OR10G9 | 0.20 | 1.52E-02 |
| 1469 | RPL19 | 0.32 | 1.53E-02 |
| 1470 | AGA | -0.28 | 1.53E-02 |
| 1471 | MLNR | 0.42 | 1.53E-02 |
| 1472 | H2AFV | -0.28 | 1.53E-02 |
| 1473 | C20orf121 | -0.30 | 1.53E-02 |
| 1474 | HAPLN3 | -0.38 | 1.53E-02 |
| 1475 | PMS2L11 | -0.21 | 1.53E-02 |
| 1476 | AGPAT2 | 0.36 | 1.53E-02 |
| 1477 | MACF1 | -0.25 | 1.53E-02 |
| 1478 | MAGEA10 | -0.17 | 1.53E-02 |
| 1479 | TPCN1 | -0.21 | 1.53E-02 |
| 1480 | MTERFD2 | 0.18 | 1.53E-02 |
| 1481 | OR1B1 | 0.29 | 1.53E-02 |
| 1482 | VPS72 | -0.18 | 1.53E-02 |
| 1483 | MYO9A | -0.33 | 1.53E-02 |
| 1484 | C10orf26 | -0.37 | 1.53E-02 |
| 1485 | CSF2RA | -0.18 | 1.53E-02 |
| 1486 | RGS7BP | 0.24 | 1.53E-02 |
| 1487 | MCCC1 | -0.24 | 1.53E-02 |
| 1488 | KBTBD2 | 0.31 | 1.53E-02 |
| 1489 | GPR171 | 0.30 | 1.53E-02 |
| 1490 | LOC255275 | 0.15 | 1.53E-02 |
| 1491 | QKI | -0.42 | 1.53E-02 |
| 1492 | KCTD9 | -0.27 | 1.53E-02 |
| 1493 | SLC8A1 | 0.29 | 1.53E-02 |
| 1494 | CHRNB1 | 0.29 | 1.53E-02 |
| 1495 | RPS8 | 0.34 | 1.53E-02 |
| 1496 | TMED9 | -0.27 | 1.53E-02 |
| 1497 | WNT10B | 0.43 | 1.53E-02 |
| 1498 | MAN1A2 | -0.45 | 1.53E-02 |
| 1499 | TLR7 | 0.21 | 1.53E-02 |
| 1500 | GIP | 0.35 | 1.53E-02 |
| 1501 | PDX1 | 0.20 | 1.53E-02 |
| 1502 | LMAN2L | -0.23 | 1.53E-02 |
| 1503 | ANKRD21 | 0.33 | 1.53E-02 |
| 1504 | IL9R | 0.27 | 1.53E-02 |
| 1505 | IGSF2 | 0.29 | 1.53E-02 |
| 1506 | GPR172B | 0.31 | 1.53E-02 |
| 1507 | FLJ22222 | -0.21 | 1.53E-02 |
| 1508 | CDCP1 | -0.25 | 1.53E-02 |
| 1509 | PAPPA2 | 0.33 | 1.53E-02 |
| 1510 | THY1 | -0.27 | 1.53E-02 |
| 1511 | HOXA11 | 0.35 | 1.53E-02 |
| 1512 | B9D1 | 0.25 | 1.53E-02 |
| 1513 | IGSF3 | 0.16 | 1.53E-02 |
| 1514 | PKHD1 | 0.24 | 1.53E-02 |
| 1515 | WFS1 | -0.28 | 1.53E-02 |
| 1516 | NEU1 | -0.28 | 1.53E-02 |
| 1517 | ADAMTS8 | 0.23 | 1.53E-02 |
| 1518 | BCKDHB | 0.29 | 1.53E-02 |
| 1519 | IFNA6 | 0.62 | 1.53E-02 |
| 1520 | IRAK1BP1 | 0.43 | 1.53E-02 |
| 1521 | ATXN3 | 0.25 | 1.53E-02 |
| 1522 | GALNT2 | -0.28 | 1.53E-02 |
| 1523 | MAN1A2 | -0.27 | 1.53E-02 |
| 1524 | TOM1L2 | -0.18 | 1.53E-02 |
| 1525 | SH3GLP1 | 0.40 | 1.53E-02 |
| 1526 | CDK5RAP2 | -0.21 | 1.53E-02 |
| 1527 | DMTF1 | 0.25 | 1.53E-02 |
| 1528 | LOC729338 | 0.27 | 1.53E-02 |
| 1529 | DGCR7 | 0.28 | 1.53E-02 |
| 1530 | LOC642361 | -0.20 | 1.53E-02 |
| 1531 | CRIM1 | 0.36 | 1.54E-02 |
| 1532 | C6orf21 | 0.30 | 1.54E-02 |
| 1533 | NAG | -0.30 | 1.54E-02 |
| 1534 | C5orf30 | 0.32 | 1.54E-02 |
| 1535 | CCDC14 | -0.25 | 1.54E-02 |
| 1536 | ANKRD47 | 0.21 | 1.54E-02 |
| 1537 | ZNF595 | 0.38 | 1.54E-02 |
| 1538 | F2RL1 | 0.28 | 1.54E-02 |
| 1539 | CCDC94 | 0.21 | 1.54E-02 |
| 1540 | PFN1 | -0.26 | 1.54E-02 |
| 1541 | ZRANB3 | 0.64 | 1.54E-02 |
| 1542 | SYT11 | -0.20 | 1.54E-02 |
| 1543 | TREML3 | 0.16 | 1.54E-02 |
| 1544 | KIF24 | -0.48 | 1.54E-02 |
| 1545 | JAG1 | -0.35 | 1.54E-02 |
| 1546 | HELZ | -0.30 | 1.54E-02 |
| 1547 | TMEM29 | -0.28 | 1.54E-02 |
| 1548 | BNIP3 | -0.22 | 1.54E-02 |
| 1549 | DOCK4 | 0.17 | 1.54E-02 |
| 1550 | AFF4 | -0.17 | 1.54E-02 |
| 1551 | CNOT2 | -0.17 | 1.54E-02 |
| 1552 | RAB11B | 0.34 | 1.54E-02 |
| 1553 | FREM1 | 0.30 | 1.54E-02 |
| 1554 | OR1J2 | 0.26 | 1.54E-02 |
| 1555 | ACTA1 | -0.26 | 1.54E-02 |
| 1556 | HCG8///C6orf12 | 0.20 | 1.54E-02 |
| 1557 | RPS16 | 0.34 | 1.54E-02 |
| 1558 | BHLHB5 | 0.29 | 1.54E-02 |
| 1559 | ZNRF3 | -0.52 | 1.54E-02 |
| 1560 | COX6A2 | 0.39 | 1.54E-02 |
| 1561 | ZNF560 | 0.31 | 1.54E-02 |
| 1562 | ADAMTS9 | -0.56 | 1.54E-02 |
| 1563 | OR10H3 | 0.20 | 1.54E-02 |
| 1564 | HIPK4 | 0.38 | 1.54E-02 |
| 1565 | RPL26 | 0.27 | 1.55E-02 |
| 1566 | CIDEB | 0.36 | 1.55E-02 |
| 1567 | ATP5J | 0.23 | 1.55E-02 |
| 1568 | TMEM161A | -0.18 | 1.55E-02 |
| 1569 | CDR2 | -0.38 | 1.55E-02 |
| 1570 | ORAI1 | -0.17 | 1.55E-02 |
| 1571 | CCNA1 | 0.18 | 1.55E-02 |
| 1572 | SLC13A2 | 0.28 | 1.55E-02 |
| 1573 | ATG4A | 0.23 | 1.55E-02 |
| 1574 | IHPK2 | -0.26 | 1.55E-02 |
| 1575 | NT5C1B | 0.37 | 1.55E-02 |
| 1576 | ABCF3 | -0.19 | 1.55E-02 |
| 1577 | TRPM7 | -0.50 | 1.55E-02 |
| 1578 | THSD3 | 0.21 | 1.55E-02 |
| 1579 | SPOP | -0.23 | 1.55E-02 |
| 1580 | IPO9 | -0.29 | 1.55E-02 |
| 1581 | HCN4 | 0.26 | 1.55E-02 |
| 1582 | SCN11A | 0.41 | 1.55E-02 |
| 1583 | PLEKHM2 | -0.15 | 1.55E-02 |
| 1584 | PRPF38B | -0.45 | 1.55E-02 |
| 1585 | SLC23A3 | -0.29 | 1.55E-02 |
| 1586 | DENND2C | 0.48 | 1.55E-02 |
| 1587 | CCDC38 | 0.28 | 1.55E-02 |
| 1588 | MEIS1 | -0.26 | 1.55E-02 |
| 1589 | CSNK2B | -0.16 | 1.56E-02 |
| 1590 | VGF | 0.31 | 1.56E-02 |
| 1591 | FRMD4A | -0.27 | 1.56E-02 |
| 1592 | ATAD4 | 0.26 | 1.56E-02 |
| 1593 | SENP1 | 0.26 | 1.56E-02 |
| 1594 | OBSL1 | 0.27 | 1.56E-02 |
| 1595 | HLA-G | -0.31 | 1.56E-02 |
| 1596 | COL2A1 | -0.20 | 1.56E-02 |
| 1597 | HERC1 | -0.31 | 1.56E-02 |
| 1598 | LOC440900 | 0.22 | 1.56E-02 |
| 1599 | EHBP1L1 | -0.22 | 1.56E-02 |
| 1600 | APBA3 | -0.20 | 1.56E-02 |
| 1601 | UBXD6 | 0.25 | 1.56E-02 |
| 1602 | FPGS | -0.13 | 1.56E-02 |
| 1603 | LRRC24 | 0.16 | 1.56E-02 |
| 1604 | UPK1B | 0.38 | 1.57E-02 |
| 1605 | RRAGD | 0.39 | 1.57E-02 |
| 1606 | LOC729507 | 0.54 | 1.57E-02 |
| 1607 | RIMBP2 | 0.26 | 1.57E-02 |
| 1608 | CCPG1 | -0.35 | 1.57E-02 |
| 1609 | MC5R | 0.24 | 1.57E-02 |
| 1610 | ZBTB40 | -0.29 | 1.57E-02 |
| 1611 | IKZF2 | 0.22 | 1.57E-02 |
| 1612 | HHEX | -0.19 | 1.57E-02 |
| 1613 | TBL3 | -0.19 | 1.57E-02 |
| 1614 | COX7B2 | 0.43 | 1.57E-02 |
| 1615 | ATP2B2 | 0.16 | 1.58E-02 |
| 1616 | SHB | -0.22 | 1.58E-02 |
| 1617 | IRAK4 | -0.28 | 1.58E-02 |
| 1618 | FGFR1 | -0.27 | 1.58E-02 |
| 1619 | RCE1 | -0.25 | 1.58E-02 |
| 1620 | SSX2 | -0.37 | 1.58E-02 |
| 1621 | AMY1B///AMY1A | -0.36 | 1.58E-02 |
| 1622 | SLC39A13 | -0.36 | 1.58E-02 |
| 1623 | C11orf35 | 0.17 | 1.58E-02 |
| 1624 | SLC4A2 | 0.40 | 1.59E-02 |
| 1625 | SH2D2A | -0.30 | 1.59E-02 |
| 1626 | COG4 | -0.24 | 1.59E-02 |
| 1627 | ZNF714 | 0.20 | 1.59E-02 |
| 1628 | C6orf12 | 0.29 | 1.59E-02 |
| 1629 | DEFB123 | 0.21 | 1.59E-02 |
| 1630 | CNTN6 | 0.28 | 1.59E-02 |
| 1631 | ARFGAP1 | -0.19 | 1.59E-02 |
| 1632 | CCL1 | -0.24 | 1.59E-02 |
| 1633 | ZNF536 | 0.16 | 1.59E-02 |
| 1634 | WDR42C | 0.33 | 1.59E-02 |
| 1635 | HFE | -0.16 | 1.59E-02 |
| 1636 | PDGFA | -0.42 | 1.59E-02 |
| 1637 | LASP1 | -0.23 | 1.59E-02 |
| 1638 | ACSM4 | 0.13 | 1.59E-02 |
| 1639 | CLK2 | -0.21 | 1.59E-02 |
| 1640 | MLL2 | -0.30 | 1.59E-02 |
| 1641 | NUP85 | -0.22 | 1.59E-02 |
| 1642 | COX7C | 0.27 | 1.59E-02 |
| 1643 | NFAM1 | 0.20 | 1.59E-02 |
| 1644 | TIAF1 | -0.21 | 1.59E-02 |
| 1645 | CNGA3 | 0.23 | 1.60E-02 |
| 1646 | SMURF1 | -0.41 | 1.60E-02 |
| 1647 | LOC283378 | -0.35 | 1.60E-02 |
| 1648 | SLC28A2 | 0.25 | 1.60E-02 |
| 1649 | TTC16 | 0.17 | 1.60E-02 |
| 1650 | CDX2 | 0.24 | 1.60E-02 |
| 1651 | CCDC45 | -0.21 | 1.60E-02 |
| 1652 | LCN9 | 0.29 | 1.60E-02 |
| 1653 | NCAN | 0.23 | 1.60E-02 |
| 1654 | INTU | -0.43 | 1.60E-02 |
| 1655 | NUDT15 | -0.26 | 1.60E-02 |
| 1656 | CLCA4 | 0.32 | 1.60E-02 |
| 1657 | SERPINB4 | 0.29 | 1.61E-02 |
| 1658 | FLJ21075 | 0.26 | 1.61E-02 |
| 1659 | FLJ25778 | 0.28 | 1.61E-02 |
| 1660 | LOC387895 | 0.45 | 1.61E-02 |
| 1661 | TRIM65 | -0.17 | 1.61E-02 |
| 1662 | CCKBR | 0.41 | 1.61E-02 |
| 1663 | EVI2B | 0.19 | 1.61E-02 |
| 1664 | STAT2 | 0.36 | 1.61E-02 |
| 1665 | ZNF266 | -0.21 | 1.61E-02 |
| 1666 | ZNF652 | -0.34 | 1.61E-02 |
| 1667 | C2orf37 | -0.30 | 1.61E-02 |
| 1668 | VPS13C | -0.36 | 1.61E-02 |
| 1669 | FLJ10154 | -0.24 | 1.61E-02 |
| 1670 | CAND1 | -0.23 | 1.61E-02 |
| 1671 | NISCH | -0.21 | 1.61E-02 |
| 1672 | CITED1 | 0.31 | 1.61E-02 |
| 1673 | WBSCR22 | -0.18 | 1.61E-02 |
| 1674 | GPSN2 | -0.28 | 1.61E-02 |
| 1675 | SLC9A1 | -0.32 | 1.61E-02 |
| 1676 | CCDC35 | 0.72 | 1.62E-02 |
| 1677 | FBXO9 | -0.38 | 1.62E-02 |
| 1678 | ZNF8 | -0.22 | 1.62E-02 |
| 1679 | GH2 | 0.26 | 1.62E-02 |
| 1680 | NPBWR2 | 0.23 | 1.62E-02 |
| 1681 | PKN1 | -0.12 | 1.62E-02 |
| 1682 | ADAMTS20 | 0.29 | 1.62E-02 |
| 1683 | EPHB2 | 0.45 | 1.62E-02 |
| 1684 | C14orf39 | 0.29 | 1.62E-02 |
| 1685 | MICAL3 | -0.26 | 1.62E-02 |
| 1686 | RPL7A | 0.32 | 1.62E-02 |
| 1687 | C20orf174 | 0.20 | 1.62E-02 |
| 1688 | ICAM4 | -0.20 | 1.62E-02 |
| 1689 | HTR1A | 0.20 | 1.62E-02 |
| 1690 | LOC728208 | 0.61 | 1.62E-02 |
| 1691 | RPS6KC1 | -0.20 | 1.62E-02 |
| 1692 | KRTAP9-8 | 0.30 | 1.63E-02 |
| 1693 | SLC16A7 | -0.32 | 1.63E-02 |
| 1694 | WFDC9 | 0.25 | 1.63E-02 |
| 1695 | OR51T1 | 0.21 | 1.63E-02 |
| 1696 | APOA1 | -0.20 | 1.63E-02 |
| 1697 | SC4MOL | -0.40 | 1.63E-02 |
| 1698 | WNK3 | 0.29 | 1.63E-02 |
| 1699 | RPS6 | 0.27 | 1.63E-02 |
| 1700 | FGF17 | 0.20 | 1.63E-02 |
| 1701 | USP10 | -0.24 | 1.63E-02 |
| 1702 | CUZD1 | 0.27 | 1.63E-02 |
| 1703 | BAGE4 | -0.22 | 1.63E-02 |
| 1704 | SGK269 | -0.19 | 1.63E-02 |
| 1705 | RPL38 | 0.16 | 1.63E-02 |
| 1706 | BCAS4 | -0.20 | 1.64E-02 |
| 1707 | MRM1 | -0.20 | 1.64E-02 |
| 1708 | MALAT1 | -0.48 | 1.64E-02 |
| 1709 | RNF17 | 0.12 | 1.64E-02 |
| 1710 | MATN1 | 0.20 | 1.64E-02 |
| 1711 | TNNI2 | -0.31 | 1.64E-02 |
| 1712 | LOC114227 | 0.23 | 1.64E-02 |
| 1713 | MBOAT1 | -0.43 | 1.64E-02 |
| 1714 | CTLA4 | -0.47 | 1.64E-02 |
| 1715 | psiTPTE22 | 0.20 | 1.64E-02 |
| 1716 | LOC375748 | 0.33 | 1.64E-02 |
| 1717 | MAGEH1 | -0.20 | 1.64E-02 |
| 1718 | C9orf96 | 0.26 | 1.64E-02 |
| 1719 | SLC8A3 | 0.29 | 1.64E-02 |
| 1720 | SNORD49A | -0.32 | 1.64E-02 |
| 1721 | COL9A2 | 0.63 | 1.64E-02 |
| 1722 | RPL7A | 0.25 | 1.64E-02 |
| 1723 | APLP2 | -0.33 | 1.65E-02 |
| 1724 | ARL6IP2 | -0.37 | 1.65E-02 |
| 1725 | ANKRD46 | 0.22 | 1.65E-02 |
| 1726 | UXS1 | -0.36 | 1.65E-02 |
| 1727 | RPL37A | 0.27 | 1.65E-02 |
| 1728 | RAP1A | 0.35 | 1.65E-02 |
| 1729 | PLCXD3 | 0.51 | 1.65E-02 |
| 1730 | FAM134C | -0.31 | 1.65E-02 |
| 1731 | MSL2L1 | -0.21 | 1.65E-02 |
| 1732 | AMPD1 | 0.34 | 1.66E-02 |
| 1733 | FZD6 | -0.32 | 1.66E-02 |
| 1734 | SNAP25 | 0.27 | 1.66E-02 |
| 1735 | ABCF2 | -0.21 | 1.66E-02 |
| 1736 | LOC93444 | 0.27 | 1.66E-02 |
| 1737 | BAGE | 0.18 | 1.66E-02 |
| 1738 | MON2 | -0.34 | 1.66E-02 |
| 1739 | LYPLA3 | -0.21 | 1.66E-02 |
| 1740 | ANGEL1 | -0.23 | 1.66E-02 |
| 1741 | C16orf45 | -0.29 | 1.66E-02 |
| 1742 | SNRPG | 0.22 | 1.66E-02 |
| 1743 | DPYSL4 | -0.17 | 1.66E-02 |
| 1744 | RASGEF1B | 0.20 | 1.66E-02 |
| 1745 | TRAPPC1 | -0.19 | 1.66E-02 |
| 1746 | RSC1A1 | 0.23 | 1.67E-02 |
| 1747 | RBM7 | 0.42 | 1.67E-02 |
| 1748 | COX4I2 | 0.14 | 1.67E-02 |
| 1749 | HOXB1 | 0.35 | 1.67E-02 |
| 1750 | SLC6A1 | -0.53 | 1.67E-02 |
| 1751 | NLRP7 | -0.26 | 1.67E-02 |
| 1752 | RBMY1J | 0.34 | 1.67E-02 |
| 1753 | GOT1 | -0.29 | 1.67E-02 |
| 1754 | ATP6V0A1 | -0.19 | 1.67E-02 |
| 1755 | EDEM3 | -0.40 | 1.67E-02 |
| 1756 | NTS | -0.26 | 1.67E-02 |
| 1757 | PLXND1 | -0.26 | 1.67E-02 |
| 1758 | EMILIN1 | -0.36 | 1.67E-02 |
| 1759 | ADRBK2 | -0.36 | 1.67E-02 |
| 1760 | BSDC1 | 0.46 | 1.67E-02 |
| 1761 | LOC645700 | 0.32 | 1.67E-02 |
| 1762 | PCDHGA6 | 0.21 | 1.68E-02 |
| 1763 | SLC26A11 | -0.29 | 1.69E-02 |
| 1764 | MGC21881 | -0.29 | 1.69E-02 |
| 1765 | MAOB | 0.44 | 1.69E-02 |
| 1766 | DRD1IP | -0.19 | 1.69E-02 |
| 1767 | BPTF | -0.20 | 1.70E-02 |
| 1768 | SPATA8 | 0.23 | 1.70E-02 |
| 1769 | TNR | 0.32 | 1.70E-02 |
| 1770 | LOC388210 | 0.33 | 1.70E-02 |
| 1771 | CDC123 | 0.28 | 1.70E-02 |
| 1772 | ABCG1 | -0.32 | 1.70E-02 |
| 1773 | RBMY1A1 | 0.31 | 1.70E-02 |
| 1774 | APOA1 | 0.47 | 1.71E-02 |
| 1775 | YAP1 | -0.23 | 1.71E-02 |
| 1776 | CENTD3 | -0.30 | 1.71E-02 |
| 1777 | FAM75C1 | 0.22 | 1.71E-02 |
| 1778 | ZNF614 | -0.39 | 1.71E-02 |
| 1779 | SCD5 | 0.35 | 1.71E-02 |
| 1780 | ANGPTL3 | 0.22 | 1.71E-02 |
| 1781 | KIF5C | -0.21 | 1.71E-02 |
| 1782 | FAM55D | 0.32 | 1.71E-02 |
| 1783 | SNCA | 0.35 | 1.71E-02 |
| 1784 | ZNF696 | -0.30 | 1.71E-02 |
| 1785 | SETDB1 | -0.21 | 1.71E-02 |
| 1786 | A2ML1 | 0.55 | 1.71E-02 |
| 1787 | ANAPC7 | -0.17 | 1.71E-02 |
| 1788 | LOC285033 | 0.29 | 1.71E-02 |
| 1789 | CD63 | -0.18 | 1.71E-02 |
| 1790 | C1orf34 | 0.35 | 1.71E-02 |
| 1791 | GALNT10 | 0.20 | 1.71E-02 |
| 1792 | LOC727819 | 0.41 | 1.72E-02 |
| 1793 | AP4M1 | 0.19 | 1.72E-02 |
| 1794 | CYP11B1 | 0.19 | 1.72E-02 |
| 1795 | PPM1G | -0.25 | 1.72E-02 |
| 1796 | RBM25 | -0.32 | 1.72E-02 |
| 1797 | C10orf12 | -0.16 | 1.72E-02 |
| 1798 | ZNF623 | -0.27 | 1.72E-02 |
| 1799 | TMEM44 | -0.25 | 1.72E-02 |
| 1800 | PXDN | -0.24 | 1.72E-02 |
| 1801 | PABPC1 | 0.36 | 1.73E-02 |
| 1802 | PSMC3IP | -0.28 | 1.73E-02 |
| 1803 | SCML4 | 0.23 | 1.73E-02 |
| 1804 | SEMA6A | -0.27 | 1.73E-02 |
| 1805 | PLA2G4D | 0.16 | 1.73E-02 |
| 1806 | BGLAP | -0.19 | 1.73E-02 |
| 1807 | LOC284632 | 0.27 | 1.73E-02 |
| 1808 | KIAA0492 | -0.43 | 1.73E-02 |
| 1809 | ZFP2 | 0.49 | 1.73E-02 |
| 1810 | KIAA0232 | -0.38 | 1.73E-02 |
| 1811 | DHX16 | -0.13 | 1.73E-02 |
| 1812 | GCK | 0.37 | 1.73E-02 |
| 1813 | LOC143381 | -0.23 | 1.73E-02 |
| 1814 | FAM12B | 0.23 | 1.74E-02 |
| 1815 | CHRNA1 | 0.23 | 1.74E-02 |
| 1816 | HPCA | 0.18 | 1.74E-02 |
| 1817 | RPS3 | 0.42 | 1.74E-02 |
| 1818 | PELO | -0.35 | 1.74E-02 |
| 1819 | SF1 | -0.13 | 1.74E-02 |
| 1820 | LARP1 | -0.27 | 1.74E-02 |
| 1821 | SERPINH1 | -0.25 | 1.74E-02 |
| 1822 | LOC128977 | -0.27 | 1.74E-02 |
| 1823 | PRPF4B | 0.50 | 1.74E-02 |
| 1824 | LOC283403 | 0.30 | 1.74E-02 |
| 1825 | EFEMP2 | -0.19 | 1.74E-02 |
| 1826 | SCOTIN | -0.20 | 1.74E-02 |
| 1827 | DHRS13 | -0.18 | 1.74E-02 |
| 1828 | LOC388946 | 0.38 | 1.74E-02 |
| 1829 | NPC1 | -0.30 | 1.74E-02 |
| 1830 | PPARD | -0.22 | 1.74E-02 |
| 1831 | HIST1H2BM | -0.21 | 1.74E-02 |
| 1832 | GALNAC4S-6ST | -0.41 | 1.74E-02 |
| 1833 | GMPR | 0.16 | 1.74E-02 |
| 1834 | TUT1 | -0.22 | 1.74E-02 |
| 1835 | TXN | 0.33 | 1.74E-02 |
| 1836 | CD44 | -0.29 | 1.75E-02 |
| 1837 | OXER1 | 0.36 | 1.75E-02 |
| 1838 | FAM78B | -0.18 | 1.75E-02 |
| 1839 | DGKQ | -0.32 | 1.75E-02 |
| 1840 | ASB5 | 0.22 | 1.75E-02 |
| 1841 | RASGRF1 | 0.27 | 1.75E-02 |
| 1842 | ERBB4 | 0.25 | 1.75E-02 |
| 1843 | TFAP2C | 0.18 | 1.75E-02 |
| 1844 | DDX23 | -0.14 | 1.75E-02 |
| 1845 | CPS1 | -0.24 | 1.75E-02 |
| 1846 | CCDC110 | 0.24 | 1.75E-02 |
| 1847 | UNQ2963 | -0.23 | 1.75E-02 |
| 1848 | PTPRM | -0.37 | 1.75E-02 |
| 1849 | PCDHGA3 | 0.18 | 1.75E-02 |
| 1850 | SLC27A3 | -0.33 | 1.75E-02 |
| 1851 | EXOC3L2 | 0.30 | 1.75E-02 |
| 1852 | KRTAP10-11 | 0.24 | 1.76E-02 |
| 1853 | TGFB3 | 0.23 | 1.76E-02 |
| 1854 | EMP2 | -0.29 | 1.76E-02 |
| 1855 | SEPT9 | -0.21 | 1.76E-02 |
| 1856 | CCL20 | -0.41 | 1.76E-02 |
| 1857 | NUFIP2 | -0.23 | 1.76E-02 |
| 1858 | ORMDL3 | -0.32 | 1.76E-02 |
| 1859 | AKR1D1 | 0.28 | 1.76E-02 |
| 1860 | SMR3B | 0.17 | 1.76E-02 |
| 1861 | DRP2 | 0.26 | 1.76E-02 |
| 1862 | ITIH3 | -0.18 | 1.77E-02 |
| 1863 | ZKSCAN1 | -0.20 | 1.77E-02 |
| 1864 | TMEM19 | -0.21 | 1.77E-02 |
| 1865 | OCLM | 0.12 | 1.77E-02 |
| 1866 | AMIGO1 | -0.27 | 1.77E-02 |
| 1867 | WNT10A | 0.30 | 1.77E-02 |
| 1868 | HIP1R | -0.29 | 1.77E-02 |
| 1869 | GPI | -0.29 | 1.77E-02 |
| 1870 | OR10Q1 | 0.13 | 1.77E-02 |
| 1871 | C4orf35 | 0.25 | 1.77E-02 |
| 1872 | RFESD | 0.21 | 1.77E-02 |
| 1873 | HSPA12A | 0.26 | 1.77E-02 |
| 1874 | RABGAP1L | -0.19 | 1.77E-02 |
| 1875 | MPL | 0.21 | 1.78E-02 |
| 1876 | LOC253805 | 0.32 | 1.78E-02 |
| 1877 | RPUSD1 | -0.19 | 1.78E-02 |
| 1878 | NOTCH4 | -0.19 | 1.78E-02 |
| 1879 | HPSE2 | 0.27 | 1.78E-02 |
| 1880 | SNORD46 | -0.20 | 1.78E-02 |
| 1881 | LOC284542 | 0.24 | 1.78E-02 |
| 1882 | TPR | -0.17 | 1.78E-02 |
| 1883 | CCR9 | 0.29 | 1.78E-02 |
| 1884 | CALCA | 0.45 | 1.78E-02 |
| 1885 | TAAR5 | -0.19 | 1.78E-02 |
| 1886 | GH2 | 0.25 | 1.78E-02 |
| 1887 | KRTAP2-4 | 0.44 | 1.78E-02 |
| 1888 | GPX7 | -0.25 | 1.78E-02 |
| 1889 | ZNF207 | -0.28 | 1.78E-02 |
| 1890 | ZNF623 | 0.23 | 1.78E-02 |
| 1891 | EDEM3 | -0.40 | 1.78E-02 |
| 1892 | TWSG1 | -0.28 | 1.78E-02 |
| 1893 | FBXO42 | -0.36 | 1.78E-02 |
| 1894 | VPS16 | -0.20 | 1.78E-02 |
| 1895 | DHX37 | -0.20 | 1.78E-02 |
| 1896 | NPLOC4 | -0.30 | 1.78E-02 |
| 1897 | ADAM29 | 0.43 | 1.78E-02 |
| 1898 | OTX2 | 0.50 | 1.79E-02 |
| 1899 | RPL8 | 0.33 | 1.79E-02 |
| 1900 | GPR114 | 0.28 | 1.79E-02 |
| 1901 | TRAF2 | -0.28 | 1.79E-02 |
| 1902 | SIGLEC14 | 0.19 | 1.79E-02 |
| 1903 | LOC283692 | 0.15 | 1.79E-02 |
| 1904 | OCIAD2 | 0.23 | 1.79E-02 |
| 1905 | LOC728612 | 0.23 | 1.79E-02 |
| 1906 | SERPINI1 | -0.27 | 1.79E-02 |
| 1907 | TI-227H | -0.30 | 1.80E-02 |
| 1908 | ASPHD2 | -0.30 | 1.80E-02 |
| 1909 | FSCN3 | 0.33 | 1.80E-02 |
| 1910 | FLJ38122 | 0.27 | 1.80E-02 |
| 1911 | SERINC2 | -0.29 | 1.80E-02 |
| 1912 | CCDC142 | -0.28 | 1.80E-02 |
| 1913 | VCAN | -0.35 | 1.80E-02 |
| 1914 | RETNLB | 0.32 | 1.80E-02 |
| 1915 | FLJ45557 | 0.17 | 1.80E-02 |
| 1916 | OR51A2 | 0.30 | 1.80E-02 |
| 1917 | LOC731986 | 0.43 | 1.80E-02 |
| 1918 | OR2L1P | 0.34 | 1.80E-02 |
| 1919 | TAF6 | -0.27 | 1.80E-02 |
| 1920 | JMJD5 | 0.22 | 1.80E-02 |
| 1921 | FAM148C | 0.20 | 1.80E-02 |
| 1922 | C6orf192 | -0.26 | 1.80E-02 |
| 1923 | ZNF449 | -0.36 | 1.81E-02 |
| 1924 | OR5V1 | 0.47 | 1.81E-02 |
| 1925 | MFAP3 | -0.20 | 1.81E-02 |
| 1926 | BAGE2 | -0.22 | 1.81E-02 |
| 1927 | MBTPS1 | -0.21 | 1.81E-02 |
| 1928 | C10orf4 | -0.31 | 1.81E-02 |
| 1929 | LEPROT | -0.36 | 1.81E-02 |
| 1930 | TTC12 | -0.29 | 1.81E-02 |
| 1931 | LOC285548 | 0.31 | 1.81E-02 |
| 1932 | CSF2RA | -0.30 | 1.81E-02 |
| 1933 | PTPRT | 0.38 | 1.81E-02 |
| 1934 | IL1RAPL1 | 0.27 | 1.81E-02 |
| 1935 | DPEP1 | 0.27 | 1.81E-02 |
| 1936 | AGBL2 | 0.22 | 1.81E-02 |
| 1937 | METTL3 | 0.23 | 1.82E-02 |
| 1938 | LOC645553 | 0.18 | 1.82E-02 |
| 1939 | LOC285389 | 0.21 | 1.82E-02 |
| 1940 | TXNL1 | -0.18 | 1.82E-02 |
| 1941 | ULBP1 | 0.35 | 1.82E-02 |
| 1942 | FAM137B | 0.24 | 1.82E-02 |
| 1943 | OR10A2 | 0.27 | 1.82E-02 |
| 1944 | GPR19 | 0.39 | 1.82E-02 |
| 1945 | RBM12 | -0.30 | 1.82E-02 |
| 1946 | KCNMB2 | 0.23 | 1.82E-02 |
| 1947 | P2RX2 | 0.24 | 1.82E-02 |
| 1948 | PRIC285 | -0.38 | 1.82E-02 |
| 1949 | LOC349160 | 0.22 | 1.82E-02 |
| 1950 | LOC728676 | 0.22 | 1.82E-02 |
| 1951 | GRIK1 | 0.21 | 1.83E-02 |
| 1952 | CACNA1F | 0.33 | 1.83E-02 |
| 1953 | CDON | -0.23 | 1.83E-02 |
| 1954 | TXNDC12 | -0.26 | 1.83E-02 |
| 1955 | TUBD1 | -0.21 | 1.83E-02 |
| 1956 | ASAH3 | 0.25 | 1.83E-02 |
| 1957 | SMA4 | -0.55 | 1.83E-02 |
| 1958 | MORN3 | 0.31 | 1.83E-02 |
| 1959 | LOC391742 | 0.53 | 1.83E-02 |
| 1960 | TRIM26 | -0.27 | 1.83E-02 |
| 1961 | KIAA0508 | -0.18 | 1.83E-02 |
| 1962 | LOC284417 | -0.15 | 1.83E-02 |
| 1963 | EDG6 | -0.38 | 1.83E-02 |
| 1964 | SEMA3D | 0.22 | 1.83E-02 |
| 1965 | GRP | 0.18 | 1.83E-02 |
| 1966 | SLC35E2 | -0.33 | 1.83E-02 |
| 1967 | ZNF579 | -0.16 | 1.83E-02 |
| 1968 | LOC256374 | -0.26 | 1.84E-02 |
| 1969 | RAPGEF2 | -0.47 | 1.84E-02 |
| 1970 | BTRC | -0.22 | 1.84E-02 |
| 1971 | BLMH | 0.29 | 1.84E-02 |
| 1972 | LRRC16 | 0.15 | 1.84E-02 |
| 1973 | IL12RB1 | 0.29 | 1.84E-02 |
| 1974 | KRTAP12-2 | 0.34 | 1.84E-02 |
| 1975 | TDGF3 | 0.29 | 1.84E-02 |
| 1976 | ZNF384 | -0.22 | 1.84E-02 |
| 1977 | RLBP1L1 | 0.21 | 1.84E-02 |
| 1978 | CXorf6 | 0.59 | 1.84E-02 |
| 1979 | THSD4 | 0.37 | 1.84E-02 |
| 1980 | C17orf45 | 0.23 | 1.84E-02 |
| 1981 | LOC283508 | -0.38 | 1.84E-02 |
| 1982 | HIST2H2AA3 | -0.25 | 1.84E-02 |
| 1983 | ABCC5 | -0.45 | 1.84E-02 |
| 1984 | SERPINA12 | 0.13 | 1.84E-02 |
| 1985 | DMXL2 | -0.38 | 1.84E-02 |
| 1986 | SERPINB10 | 0.33 | 1.84E-02 |
| 1987 | SERPIND1 | -0.32 | 1.84E-02 |
| 1988 | SOCS4 | 0.21 | 1.84E-02 |
| 1989 | C1orf107 | -0.41 | 1.84E-02 |
| 1990 | ANKRD16 | 0.29 | 1.84E-02 |
| 1991 | CADM1 | -0.14 | 1.84E-02 |
| 1992 | ARL5A | 0.34 | 1.84E-02 |
| 1993 | PM20D1 | 0.14 | 1.84E-02 |
| 1994 | SLCO3A1 | -0.38 | 1.84E-02 |
| 1995 | LOC440799 | 0.17 | 1.84E-02 |
| 1996 | DKFZp434G179 | 0.28 | 1.84E-02 |
| 1997 | TAF1L | 0.22 | 1.84E-02 |
| 1998 | ZBED4 | 0.23 | 1.84E-02 |
| 1999 | CDRT4 | -0.21 | 1.84E-02 |
| 2000 | DEC1 | 0.18 | 1.84E-02 |
| 2001 | BTBD12 | -0.22 | 1.84E-02 |
| 2002 | CRYGC | 0.28 | 1.85E-02 |
| 2003 | PTTG1IP | 0.27 | 1.85E-02 |
| 2004 | ALG3 | -0.25 | 1.85E-02 |
| 2005 | ZNF782 | -0.47 | 1.85E-02 |
| 2006 | SLCO2A1 | -0.32 | 1.85E-02 |
| 2007 | MGC42090 | 0.17 | 1.85E-02 |
| 2008 | CLDN12 | 0.29 | 1.85E-02 |
| 2009 | ZNF599 | -0.31 | 1.85E-02 |
| 2010 | PXT1 | 0.21 | 1.85E-02 |
| 2011 | KRTAP10-3 | 0.18 | 1.85E-02 |
| 2012 | ZNF710 | -0.40 | 1.85E-02 |
| 2013 | RBMXL2 | 0.15 | 1.85E-02 |
| 2014 | AKAP10 | -0.22 | 1.85E-02 |
| 2015 | CLPTM1 | -0.20 | 1.85E-02 |
| 2016 | SLC23A2 | 0.20 | 1.85E-02 |
| 2017 | LETM2 | 0.23 | 1.85E-02 |
| 2018 | OTUB1 | -0.09 | 1.85E-02 |
| 2019 | PFN4 | 0.46 | 1.85E-02 |
| 2020 | KLHL1 | 0.29 | 1.86E-02 |
| 2021 | LAIR1 | -0.19 | 1.86E-02 |
| 2022 | ABCA7 | -0.18 | 1.86E-02 |
| 2023 | OR4N4 | 0.26 | 1.86E-02 |
| 2024 | C10orf18 | -0.58 | 1.86E-02 |
| 2025 | WNT7B | 0.19 | 1.86E-02 |
| 2026 | LOC286382 | 0.44 | 1.87E-02 |
| 2027 | SFRS3 | 0.27 | 1.87E-02 |
| 2028 | CAPSL | 0.23 | 1.87E-02 |
| 2029 | GRK5 | -0.22 | 1.87E-02 |
| 2030 | IMPDH1 | -0.21 | 1.87E-02 |
| 2031 | CYP4Z2P | 0.26 | 1.87E-02 |
| 2032 | SGPP1 | -0.26 | 1.87E-02 |
| 2033 | PPP1R1A | 0.27 | 1.87E-02 |
| 2034 | MYNN | 0.23 | 1.87E-02 |
| 2035 | PAX3 | 0.40 | 1.87E-02 |
| 2036 | GRIP1 | 0.25 | 1.87E-02 |
| 2037 | TRIML1 | 0.23 | 1.87E-02 |
| 2038 | IGLV@ | -0.12 | 1.87E-02 |
| 2039 | OR3A2 | 0.27 | 1.87E-02 |
| 2040 | C20orf142 | -0.39 | 1.87E-02 |
| 2041 | APLP2 | -0.33 | 1.87E-02 |
| 2042 | BANF1 | -0.25 | 1.87E-02 |
| 2043 | CPN1 | -0.31 | 1.87E-02 |
| 2044 | CD40 | 0.22 | 1.87E-02 |
| 2045 | PARS2 | -0.23 | 1.87E-02 |
| 2046 | FAM70B | -0.27 | 1.87E-02 |
| 2047 | HTR3E | 0.32 | 1.87E-02 |
| 2048 | KPNA3 | 0.25 | 1.87E-02 |
| 2049 | WBSCR17 | 0.31 | 1.87E-02 |
| 2050 | COQ2 | -0.20 | 1.87E-02 |
| 2051 | DHFRL1 | -0.35 | 1.87E-02 |
| 2052 | CYP7B1 | 0.36 | 1.88E-02 |
| 2053 | PRKY | -0.22 | 1.88E-02 |
| 2054 | ANKRD28 | -0.41 | 1.88E-02 |
| 2055 | CDH23 | 0.27 | 1.88E-02 |
| 2056 | NEIL1 | -0.26 | 1.88E-02 |
| 2057 | SMEK2 | 0.26 | 1.89E-02 |
| 2058 | MAL | 0.17 | 1.89E-02 |
| 2059 | PCSK5 | -0.46 | 1.89E-02 |
| 2060 | MDH1 | -0.23 | 1.89E-02 |
| 2061 | HRH3 | 0.42 | 1.89E-02 |
| 2062 | GPR23 | -0.20 | 1.89E-02 |
| 2063 | SCARB1 | -0.24 | 1.89E-02 |
| 2064 | LCAT | 0.31 | 1.89E-02 |
| 2065 | AK3///AK3L1 | -0.24 | 1.89E-02 |
| 2066 | RPL5 | 0.37 | 1.89E-02 |
| 2067 | MRPL2 | -0.20 | 1.89E-02 |
| 2068 | C16orf70 | -0.24 | 1.89E-02 |
| 2069 | PRL | 0.28 | 1.89E-02 |
| 2070 | C16orf73 | 0.46 | 1.89E-02 |
| 2071 | SBNO2 | -0.22 | 1.89E-02 |
| 2072 | PRAMEF8 | 0.20 | 1.89E-02 |
| 2073 | PPP2R1A | -0.23 | 1.90E-02 |
| 2074 | LYVE1 | 0.22 | 1.90E-02 |
| 2075 | MIA2 | 0.22 | 1.90E-02 |
| 2076 | ZNF546 | 0.20 | 1.90E-02 |
| 2077 | EFCAB6 | 0.23 | 1.90E-02 |
| 2078 | C1orf122 | 0.18 | 1.90E-02 |
| 2079 | DAP | -0.16 | 1.90E-02 |
| 2080 | CEACAM16 | 0.29 | 1.90E-02 |
| 2081 | CLIC5 | 0.43 | 1.90E-02 |
| 2082 | SERINC3 | -0.26 | 1.90E-02 |
| 2083 | ADAR | -0.16 | 1.90E-02 |
| 2084 | GATA2 | 0.29 | 1.91E-02 |
| 2085 | CSN1S2B | 0.31 | 1.91E-02 |
| 2086 | GDF3 | 0.38 | 1.91E-02 |
| 2087 | EDNRB | -0.44 | 1.91E-02 |
| 2088 | C12orf35 | -0.33 | 1.91E-02 |
| 2089 | EYA4 | 0.25 | 1.91E-02 |
| 2090 | ZNF558 | -0.33 | 1.91E-02 |
| 2091 | CLRN1 | 0.25 | 1.91E-02 |
| 2092 | AKAP5 | 0.21 | 1.91E-02 |
| 2093 | APLP2 | -0.33 | 1.91E-02 |
| 2094 | ATP2B1 | -0.29 | 1.91E-02 |
| 2095 | HSPB1 | -0.24 | 1.91E-02 |
| 2096 | YIPF3 | -0.20 | 1.92E-02 |
| 2097 | MCCC2 | -0.13 | 1.92E-02 |
| 2098 | TUBB2C | -0.34 | 1.92E-02 |
| 2099 | SNHG5 | -0.35 | 1.92E-02 |
| 2100 | LRRC20 | 0.38 | 1.92E-02 |
| 2101 | SLC8A3 | 0.47 | 1.92E-02 |
| 2102 | CYP26A1 | 0.32 | 1.92E-02 |
| 2103 | HCRT | 0.25 | 1.92E-02 |
| 2104 | FCER1G | 0.36 | 1.92E-02 |
| 2105 | FKBP4 | -0.15 | 1.92E-02 |
| 2106 | SLC2A6 | 0.25 | 1.92E-02 |
| 2107 | ANKRD19 | -0.31 | 1.92E-02 |
| 2108 | ITGB1 | -0.36 | 1.92E-02 |
| 2109 | RAB3A | 0.31 | 1.92E-02 |
| 2110 | STAMBP | -0.26 | 1.92E-02 |
| 2111 | OTUB2 | 0.37 | 1.92E-02 |
| 2112 | CDK5 | -0.21 | 1.92E-02 |
| 2113 | LOC652478///LOC646813 | 0.18 | 1.92E-02 |
| 2114 | LOC286002 | 0.14 | 1.93E-02 |
| 2115 | LOC440587 | 0.25 | 1.93E-02 |
| 2116 | AP1B1 | -0.19 | 1.93E-02 |
| 2117 | MPV17 | -0.20 | 1.93E-02 |
| 2118 | CRB2 | 0.17 | 1.93E-02 |
| 2119 | TMEM87B | -0.43 | 1.93E-02 |
| 2120 | BBS4 | -0.25 | 1.93E-02 |
| 2121 | UBXD8 | -0.31 | 1.93E-02 |
| 2122 | DCTN2 | 0.27 | 1.93E-02 |
| 2123 | RPS19 | 0.21 | 1.93E-02 |
| 2124 | ZNF428 | -0.26 | 1.94E-02 |
| 2125 | CD9 | -0.32 | 1.94E-02 |
| 2126 | OR52A4 | 0.17 | 1.94E-02 |
| 2127 | SEPT11 | 0.18 | 1.94E-02 |
| 2128 | LOC647166 | 0.27 | 1.94E-02 |
| 2129 | S100A12 | 0.38 | 1.94E-02 |
| 2130 | LAMA4 | -0.34 | 1.94E-02 |
| 2131 | BCOR | -0.48 | 1.94E-02 |
| 2132 | C10orf93 | 0.33 | 1.94E-02 |
| 2133 | TBL1Y | 0.20 | 1.94E-02 |
| 2134 | PTPRF | -0.23 | 1.95E-02 |
| 2135 | C19orf22 | -0.21 | 1.95E-02 |
| 2136 | TYRO3 | -0.21 | 1.95E-02 |
| 2137 | PPP1R14D | 0.26 | 1.95E-02 |
| 2138 | SPCS3 | 0.23 | 1.95E-02 |
| 2139 | TYR | -0.32 | 1.95E-02 |
| 2140 | UCK2 | -0.24 | 1.95E-02 |
| 2141 | LOC644100 | 0.34 | 1.95E-02 |
| 2142 | ARID5A | 0.38 | 1.95E-02 |
| 2143 | GATA1 | 0.47 | 1.95E-02 |
| 2144 | IPMK | -0.11 | 1.95E-02 |
| 2145 | B4GALT6 | 0.23 | 1.96E-02 |
| 2146 | RASSF5 | 0.14 | 1.96E-02 |
| 2147 | PYGM | 0.14 | 1.96E-02 |
| 2148 | SRRM2 | -0.23 | 1.96E-02 |
| 2149 | NUP62CL | 0.38 | 1.96E-02 |
| 2150 | CYP46A1 | 0.22 | 1.96E-02 |
| 2151 | DOHH | -0.18 | 1.96E-02 |
| 2152 | F13A1 | 0.51 | 1.96E-02 |
| 2153 | C21orf71 | 0.27 | 1.96E-02 |
| 2154 | CD40 | 0.22 | 1.96E-02 |
| 2155 | ITGB1 | -0.34 | 1.96E-02 |
| 2156 | LOC389662 | 0.26 | 1.96E-02 |
| 2157 | IL2 | 0.39 | 1.96E-02 |
| 2158 | ARHGEF15 | -0.31 | 1.96E-02 |
| 2159 | FOXP4 | -0.21 | 1.96E-02 |
| 2160 | CLDND2 | 0.36 | 1.96E-02 |
| 2161 | BTNL9 | 0.25 | 1.96E-02 |
| 2162 | IGFBP4 | -0.29 | 1.96E-02 |
| 2163 | FURIN | -0.30 | 1.96E-02 |
| 2164 | KAP2.1B | 0.31 | 1.96E-02 |
| 2165 | C10orf40 | 0.15 | 1.96E-02 |
| 2166 | CCS | -0.14 | 1.96E-02 |
| 2167 | TMEM132D | -0.17 | 1.96E-02 |
| 2168 | BPIL2 | 0.18 | 1.97E-02 |
| 2169 | SEC23A | -0.42 | 1.97E-02 |
| 2170 | CPLX4 | 0.21 | 1.97E-02 |
| 2171 | ZNF673 | -0.29 | 1.97E-02 |
| 2172 | C11orf30 | -0.23 | 1.97E-02 |
| 2173 | CLN3 | -0.25 | 1.97E-02 |
| 2174 | TAS2R38 | 0.13 | 1.97E-02 |
| 2175 | SOHLH2 | 0.29 | 1.97E-02 |
| 2176 | TLX1 | 0.30 | 1.97E-02 |
| 2177 | TMTC2 | -0.40 | 1.97E-02 |
| 2178 | CCL2 | 0.21 | 1.98E-02 |
| 2179 | HECTD3 | -0.31 | 1.98E-02 |
| 2180 | KIAA0141 | -0.15 | 1.98E-02 |
| 2181 | LRPAP1 | -0.27 | 1.98E-02 |
| 2182 | GPR6 | 0.25 | 1.98E-02 |
| 2183 | LOC479559 | 0.27 | 1.98E-02 |
| 2184 | CHKA | -0.19 | 1.98E-02 |
| 2185 | RBMY2EP | 0.22 | 1.98E-02 |
| 2186 | SMG5 | -0.15 | 1.98E-02 |
| 2187 | C3orf27 | 0.20 | 1.98E-02 |
| 2188 | TRAM1L1 | 0.33 | 1.98E-02 |
| 2189 | BCAP29 | -0.28 | 1.98E-02 |
| 2190 | FLJ90709 | -0.30 | 1.98E-02 |
| 2191 | LOC730495 | 0.33 | 1.98E-02 |
| 2192 | PRPF6 | 0.37 | 1.98E-02 |
| 2193 | BAT2 | -0.19 | 1.98E-02 |
| 2194 | ACO2 | -0.14 | 1.98E-02 |
| 2195 | LOC643449 | 0.30 | 1.98E-02 |
| 2196 | COTL1 | 0.25 | 1.99E-02 |
| 2197 | RPS15A | 0.29 | 1.99E-02 |
| 2198 | TIRAP | -0.12 | 1.99E-02 |
| 2199 | CYLD | -0.38 | 1.99E-02 |
| 2200 | LOC374395 | -0.13 | 1.99E-02 |
| 2201 | C19orf54 | -0.17 | 1.99E-02 |
| 2202 | IL17B | 0.25 | 1.99E-02 |
| 2203 | PSCD1 | -0.21 | 1.99E-02 |
| 2204 | XRCC6 | -0.15 | 1.99E-02 |
| 2205 | ZNF253 | -0.22 | 1.99E-02 |
| 2206 | GRIPAP1 | -0.18 | 2.00E-02 |
| 2207 | PRR6 | 0.24 | 2.00E-02 |
| 2208 | SLC4A4 | -0.39 | 2.00E-02 |
| 2209 | SLC31A1 | -0.24 | 2.00E-02 |
| 2210 | FGF11 | 0.18 | 2.00E-02 |
| 2211 | TMEM131 | -0.32 | 2.00E-02 |
| 2212 | C11orf24 | -0.22 | 2.00E-02 |
| 2213 | EDNRA | -0.42 | 2.00E-02 |
| 2214 | TRBV3-1 | 0.12 | 2.00E-02 |
| 2215 | C1orf173 | 0.27 | 2.00E-02 |
| 2216 | YTHDC1 | -0.41 | 2.00E-02 |
| 2217 | ZMYM1 | 0.24 | 2.00E-02 |
| 2218 | PDE5A | -0.25 | 2.00E-02 |
| 2219 | LOC441228 | 0.14 | 2.00E-02 |
| 2220 | LOC158376 | 0.16 | 2.00E-02 |
| 2221 | MAP3K8 | -0.34 | 2.00E-02 |
| 2222 | HEG1 | -0.20 | 2.00E-02 |
| 2223 | IDI1 | 0.28 | 2.00E-02 |
| 2224 | UMOD | 0.31 | 2.00E-02 |
| 2225 | CPB2 | -0.23 | 2.00E-02 |
| 2226 | RRBP1 | -0.40 | 2.00E-02 |
| 2227 | KIF26A | -0.41 | 2.00E-02 |
| 2228 | ZNF577 | -0.48 | 2.00E-02 |
| 2229 | QRICH2 | 0.27 | 2.01E-02 |
| 2230 | OR6F1 | 0.34 | 2.01E-02 |
| 2231 | RNGTT | -0.23 | 2.01E-02 |
| 2232 | SSX3 | -0.15 | 2.01E-02 |
| 2233 | PVRL3 | -0.32 | 2.01E-02 |
| 2234 | LMAN1 | 0.18 | 2.01E-02 |
| 2235 | PCDHA3 | 0.21 | 2.01E-02 |
| 2236 | HIST1H3C | 0.16 | 2.01E-02 |
| 2237 | FCRL6 | 0.25 | 2.01E-02 |
| 2238 | LOC645688 | 0.31 | 2.01E-02 |
| 2239 | MNDA | 0.33 | 2.01E-02 |
| 2240 | ZNFX1 | -0.25 | 2.01E-02 |
| 2241 | MAP4 | -0.22 | 2.01E-02 |
| 2242 | KRTAP10-2 | 0.40 | 2.01E-02 |
| 2243 | AFM | 0.23 | 2.01E-02 |
| 2244 | GABRR1 | 0.21 | 2.01E-02 |
| 2245 | TAZ | -0.25 | 2.02E-02 |
| 2246 | ZNF787 | 0.17 | 2.02E-02 |
| 2247 | FLJ25778 | 0.21 | 2.02E-02 |
| 2248 | STAT3 | -0.26 | 2.02E-02 |
| 2249 | FOXP3 | 0.17 | 2.02E-02 |
| 2250 | C20orf179 | -0.13 | 2.02E-02 |
| 2251 | CNN2 | -0.19 | 2.02E-02 |
| 2252 | RPL36AL | 0.19 | 2.02E-02 |
| 2253 | TM7SF4 | 0.31 | 2.02E-02 |
| 2254 | PLOD3 | -0.20 | 2.02E-02 |
| 2255 | ESRRB | 0.25 | 2.02E-02 |
| 2256 | PLCXD2 | 0.64 | 2.02E-02 |
| 2257 | hCG_1645727 | 0.19 | 2.02E-02 |
| 2258 | PSEN2 | -0.27 | 2.02E-02 |
| 2259 | DLGAP4 | -0.37 | 2.02E-02 |
| 2260 | LOC151171 | 0.24 | 2.02E-02 |
| 2261 | FPRL2 | 0.32 | 2.02E-02 |
| 2262 | SLC4A5 | 0.26 | 2.02E-02 |
| 2263 | C3orf21 | -0.26 | 2.02E-02 |
| 2264 | YLPM1 | -0.57 | 2.03E-02 |
| 2265 | CLEC4E | 0.29 | 2.03E-02 |
| 2266 | SPNS1 | -0.21 | 2.03E-02 |
| 2267 | STX16 | -0.22 | 2.03E-02 |
| 2268 | FOXP2 | 0.21 | 2.03E-02 |
| 2269 | RPS29 | 0.23 | 2.03E-02 |
| 2270 | TMEM86A | 0.23 | 2.03E-02 |
| 2271 | WDR33 | -0.26 | 2.03E-02 |
| 2272 | ZNF630 | 0.24 | 2.03E-02 |
| 2273 | DGKQ | 0.15 | 2.03E-02 |
| 2274 | USP13 | -0.33 | 2.03E-02 |
| 2275 | SERPINA3 | -0.29 | 2.03E-02 |
| 2276 | NBPF14 | -0.34 | 2.03E-02 |
| 2277 | THBS3 | -0.28 | 2.03E-02 |
| 2278 | ELAVL2 | -0.34 | 2.03E-02 |
| 2279 | SRPX2 | -0.33 | 2.03E-02 |
| 2280 | SGMS2 | 0.25 | 2.03E-02 |
| 2281 | CCNL2 | -0.21 | 2.03E-02 |
| 2282 | PLXNA2 | -0.43 | 2.03E-02 |
| 2283 | PIK3C2A | -0.34 | 2.03E-02 |
| 2284 | XYLT2 | -0.24 | 2.04E-02 |
| 2285 | CEP350 | -0.23 | 2.04E-02 |
| 2286 | MEGF6 | -0.19 | 2.04E-02 |
| 2287 | ALKBH5 | 0.40 | 2.04E-02 |
| 2288 | KIAA0319L | -0.15 | 2.04E-02 |
| 2289 | C6orf204 | -0.34 | 2.04E-02 |
| 2290 | CHML | -0.37 | 2.04E-02 |
| 2291 | OR10D3P | 0.30 | 2.04E-02 |
| 2292 | HOXD10 | 0.59 | 2.04E-02 |
| 2293 | EPAS1 | 0.23 | 2.04E-02 |
| 2294 | SRM | -0.26 | 2.04E-02 |
| 2295 | PLA2G2F | 0.19 | 2.04E-02 |
| 2296 | SEC16A | -0.28 | 2.04E-02 |
| 2297 | OR6C68 | 0.16 | 2.04E-02 |
| 2298 | NEFL | 0.16 | 2.05E-02 |
| 2299 | AFF2 | 0.17 | 2.05E-02 |
| 2300 | KRT84 | 0.15 | 2.05E-02 |
| 2301 | PCDHGB1 | 0.18 | 2.05E-02 |
| 2302 | MARCH7 | -0.22 | 2.05E-02 |
| 2303 | CAPZB | -0.17 | 2.05E-02 |
| 2304 | TRIM31 | 0.63 | 2.05E-02 |
| 2305 | MASP2 | 0.33 | 2.05E-02 |
| 2306 | RPS7 | 0.31 | 2.05E-02 |
| 2307 | MGAT5B | 0.40 | 2.05E-02 |
| 2308 | MOCS3 | 0.14 | 2.05E-02 |
| 2309 | SPAM1 | 0.15 | 2.05E-02 |
| 2310 | LEPROTL1 | -0.39 | 2.05E-02 |
| 2311 | HDAC6 | -0.19 | 2.05E-02 |
| 2312 | HYDIN | 0.28 | 2.06E-02 |
| 2313 | KRTAP12-3 | 0.26 | 2.06E-02 |
| 2314 | ZNF81 | 0.32 | 2.06E-02 |
| 2315 | CHRNB4 | 0.28 | 2.06E-02 |
| 2316 | STK38L | 0.21 | 2.06E-02 |
| 2317 | LOC151146 | -0.12 | 2.06E-02 |
| 2318 | GPR172A | -0.24 | 2.06E-02 |
| 2319 | HSFY1 | 0.26 | 2.06E-02 |
| 2320 | DNAH6 | 0.14 | 2.06E-02 |
| 2321 | FLJ34048 | 0.53 | 2.06E-02 |
| 2322 | C18orf30 | 0.35 | 2.06E-02 |
| 2323 | MPO | 0.15 | 2.06E-02 |
| 2324 | NOTCH3 | -0.29 | 2.06E-02 |
| 2325 | CYP2B6 | 0.21 | 2.07E-02 |
| 2326 | RAET1E | 0.17 | 2.07E-02 |
| 2327 | SOX3 | 0.33 | 2.07E-02 |
| 2328 | CACNG5 | 0.14 | 2.07E-02 |
| 2329 | FBXL21 | -0.20 | 2.07E-02 |
| 2330 | SLC38A2 | -0.28 | 2.07E-02 |
| 2331 | FAM46C | -0.34 | 2.07E-02 |
| 2332 | APOC3 | -0.31 | 2.07E-02 |
| 2333 | PHC2 | -0.11 | 2.07E-02 |
| 2334 | PRM3 | 0.34 | 2.07E-02 |
| 2335 | PCYOX1 | -0.26 | 2.07E-02 |
| 2336 | C20orf179 | 0.17 | 2.08E-02 |
| 2337 | MAPK14 | 0.14 | 2.08E-02 |
| 2338 | SOX30 | 0.22 | 2.08E-02 |
| 2339 | PSMB7 | -0.28 | 2.08E-02 |
| 2340 | C1QTNF9 | 0.27 | 2.08E-02 |
| 2341 | MYL6B | 0.20 | 2.08E-02 |
| 2342 | CYP4A11 | 0.43 | 2.08E-02 |
| 2343 | COL4A2 | -0.29 | 2.08E-02 |
| 2344 | DEFB129 | 0.29 | 2.09E-02 |
| 2345 | SDC4P | 0.23 | 2.09E-02 |
| 2346 | LOC340094 | 0.22 | 2.09E-02 |
| 2347 | PBK | 0.70 | 2.09E-02 |
| 2348 | SCAND1 | -0.21 | 2.09E-02 |
| 2349 | DYSF | 0.14 | 2.09E-02 |
| 2350 | SUPT6H | -0.15 | 2.10E-02 |
| 2351 | RXFP3 | 0.36 | 2.10E-02 |
| 2352 | DENND3 | -0.34 | 2.10E-02 |
| 2353 | IGLL1 | -0.30 | 2.10E-02 |
| 2354 | KCNH4 | 0.15 | 2.10E-02 |
| 2355 | SPINT1 | 0.13 | 2.10E-02 |
| 2356 | ENDOG | -0.15 | 2.10E-02 |
| 2357 | UBE1L2 | -0.27 | 2.10E-02 |
| 2358 | PTDSS1 | -0.18 | 2.10E-02 |
| 2359 | PPAPDC1B | -0.24 | 2.10E-02 |
| 2360 | RPS27L | 0.26 | 2.10E-02 |
| 2361 | AQP6 | 0.24 | 2.11E-02 |
| 2362 | EVX1 | 0.28 | 2.11E-02 |
| 2363 | CEP76 | -0.31 | 2.11E-02 |
| 2364 | TNFRSF9 | 0.36 | 2.11E-02 |
| 2365 | LOC654350 | 0.26 | 2.11E-02 |
| 2366 | OR51B4 | 0.28 | 2.11E-02 |
| 2367 | KRTAP9-8 | 0.25 | 2.11E-02 |
| 2368 | GPR52 | 0.26 | 2.11E-02 |
| 2369 | C21orf116 | 0.22 | 2.11E-02 |
| 2370 | OPHN1 | 0.25 | 2.11E-02 |
| 2371 | CD84 | 0.21 | 2.11E-02 |
| 2372 | FHOD1 | -0.16 | 2.11E-02 |
| 2373 | ACSL6 | 0.16 | 2.11E-02 |
| 2374 | ALPK1 | -0.37 | 2.11E-02 |
| 2375 | CRHR1 | 0.18 | 2.11E-02 |
| 2376 | LARP5 | -0.34 | 2.11E-02 |
| 2377 | LOC144766 | 0.24 | 2.11E-02 |
| 2378 | GDAP1L1 | 0.15 | 2.11E-02 |
| 2379 | PDE6C | 0.16 | 2.11E-02 |
| 2380 | FAM134B | -0.43 | 2.11E-02 |
| 2381 | ZMIZ1 | -0.24 | 2.11E-02 |
| 2382 | TAC1 | 0.23 | 2.11E-02 |
| 2383 | TMEM129 | -0.27 | 2.11E-02 |
| 2384 | PCYT1B | -0.32 | 2.11E-02 |
| 2385 | UCP3 | -0.38 | 2.11E-02 |
| 2386 | ELL2 | -0.32 | 2.11E-02 |
| 2387 | CTBP1 | -0.17 | 2.11E-02 |
| 2388 | AIPL1 | 0.25 | 2.12E-02 |
| 2389 | PQBP1 | -0.15 | 2.12E-02 |
| 2390 | HEATR6 | -0.16 | 2.12E-02 |
| 2391 | CTNNA1 | -0.18 | 2.12E-02 |
| 2392 | ZCCHC3 | 0.17 | 2.12E-02 |
| 2393 | SLC39A1 | -0.23 | 2.12E-02 |
| 2394 | GDF2 | 0.35 | 2.12E-02 |
| 2395 | PRDX4 | -0.19 | 2.12E-02 |
| 2396 | ATG9A | -0.25 | 2.12E-02 |
| 2397 | SNORA75 | 0.46 | 2.12E-02 |
| 2398 | CCDC121 | 0.34 | 2.12E-02 |
| 2399 | TNRC6B | -0.19 | 2.12E-02 |
| 2400 | ZDHHC15 | -0.20 | 2.12E-02 |
| 2401 | OR10A5 | 0.35 | 2.12E-02 |
| 2402 | CD93 | -0.47 | 2.12E-02 |
| 2403 | SULT1C3 | 0.38 | 2.12E-02 |
| 2404 | SPIRE1 | 0.42 | 2.12E-02 |
| 2405 | LOC729994 | 0.32 | 2.12E-02 |
| 2406 | LST1 | 0.26 | 2.12E-02 |
| 2407 | CHMP7 | -0.22 | 2.12E-02 |
| 2408 | ESD | 0.24 | 2.12E-02 |
| 2409 | CYP2C19 | 0.38 | 2.12E-02 |
| 2410 | BBX | -0.26 | 2.12E-02 |
| 2411 | VEPH1 | -0.32 | 2.12E-02 |
| 2412 | MAN2A2 | -0.18 | 2.12E-02 |
| 2413 | LOC149134 | 0.23 | 2.12E-02 |
| 2414 | PTPRCAP | 0.36 | 2.12E-02 |
| 2415 | POLR3A | -0.24 | 2.12E-02 |
| 2416 | C1orf168 | -0.18 | 2.12E-02 |
| 2417 | AGPAT4 | -0.34 | 2.12E-02 |
| 2418 | CD177 | 0.27 | 2.13E-02 |
| 2419 | DTNA | 0.14 | 2.13E-02 |
| 2420 | YIF1B | 0.27 | 2.13E-02 |
| 2421 | RCCD1 | 0.26 | 2.13E-02 |
| 2422 | ATF6 | 0.39 | 2.13E-02 |
| 2423 | SCN10A | 0.27 | 2.13E-02 |
| 2424 | TMEM30B | 0.17 | 2.13E-02 |
| 2425 | MAGED2 | -0.16 | 2.13E-02 |
| 2426 | SLC27A6 | -0.22 | 2.13E-02 |
| 2427 | TM9SF2 | -0.25 | 2.14E-02 |
| 2428 | C14orf166B | 0.27 | 2.14E-02 |
| 2429 | DKFZP564C196 | 0.36 | 2.14E-02 |
| 2430 | MLL5 | -0.23 | 2.14E-02 |
| 2431 | FLJ23569 | 0.15 | 2.14E-02 |
| 2432 | PIP5K1C | -0.19 | 2.14E-02 |
| 2433 | SCN2B | 0.47 | 2.14E-02 |
| 2434 | PCDHGA2 | 0.50 | 2.14E-02 |
| 2435 | LOC149837 | 0.25 | 2.14E-02 |
| 2436 | USP29 | 0.22 | 2.14E-02 |
| 2437 | FAM120A | -0.31 | 2.14E-02 |
| 2438 | PPME1 | -0.23 | 2.14E-02 |
| 2439 | USP34 | -0.53 | 2.14E-02 |
| 2440 | PLXNA2 | -0.57 | 2.14E-02 |
| 2441 | DAZ2 | 0.26 | 2.14E-02 |
| 2442 | OR10H2 | 0.22 | 2.14E-02 |
| 2443 | DKFZp667E0512 | -0.29 | 2.15E-02 |
| 2444 | SC5DL | 0.19 | 2.15E-02 |
| 2445 | ZFYVE1 | -0.19 | 2.15E-02 |
| 2446 | OMD | 0.33 | 2.15E-02 |
| 2447 | C6orf118 | 0.25 | 2.15E-02 |
| 2448 | EPM2AIP1 | -0.34 | 2.15E-02 |
| 2449 | ST7 | 0.36 | 2.15E-02 |
| 2450 | PLXNA4 | -0.35 | 2.15E-02 |
| 2451 | OR52M1 | 0.22 | 2.15E-02 |
| 2452 | GSTM2 | 0.20 | 2.15E-02 |
| 2453 | CAMK2A | 0.19 | 2.16E-02 |
| 2454 | KLC1 | -0.26 | 2.16E-02 |
| 2455 | ANAPC1 | -0.21 | 2.16E-02 |
| 2456 | C9 | 0.29 | 2.16E-02 |
| 2457 | OR1G1 | 0.21 | 2.16E-02 |
| 2458 | ZP3 | -0.20 | 2.16E-02 |
| 2459 | OR13C5 | 0.25 | 2.16E-02 |
| 2460 | NPM1 | 0.38 | 2.17E-02 |
| 2461 | LOC643276 | 0.63 | 2.17E-02 |
| 2462 | TMCO3 | -0.22 | 2.17E-02 |
| 2463 | PROZ | 0.30 | 2.17E-02 |
| 2464 | SESN2 | -0.20 | 2.17E-02 |
| 2465 | CTDSPL2 | -0.25 | 2.17E-02 |
| 2466 | UNC93B1 | -0.33 | 2.17E-02 |
| 2467 | TMC4 | -0.32 | 2.17E-02 |
| 2468 | TYROBP | 0.37 | 2.17E-02 |
| 2469 | TMEM117 | -0.38 | 2.17E-02 |
| 2470 | GAL3ST3 | 0.22 | 2.17E-02 |
| 2471 | MPPE1 | -0.31 | 2.18E-02 |
| 2472 | UCP3 | 0.44 | 2.18E-02 |
| 2473 | ZNF711 | 0.24 | 2.18E-02 |
| 2474 | OR7A5 | 0.28 | 2.18E-02 |
| 2475 | DDOST | -0.28 | 2.18E-02 |
| 2476 | CDC37L1 | 0.15 | 2.18E-02 |
| 2477 | CSF3 | 0.37 | 2.18E-02 |
| 2478 | SFT2D2 | -0.34 | 2.18E-02 |
| 2479 | SNORD54 | -0.35 | 2.18E-02 |
| 2480 | OAS2 | 0.42 | 2.18E-02 |
| 2481 | FOXE1 | 0.28 | 2.19E-02 |
| 2482 | SNF1LK | -0.30 | 2.19E-02 |
| 2483 | CRISPLD2 | -0.47 | 2.19E-02 |
| 2484 | CDC42EP3 | -0.31 | 2.19E-02 |
| 2485 | RPS12 | 0.27 | 2.19E-02 |
| 2486 | HELB | -0.62 | 2.19E-02 |
| 2487 | KLHL7 | 0.33 | 2.19E-02 |
| 2488 | ANGPTL5 | 0.15 | 2.19E-02 |
| 2489 | MDK | -0.28 | 2.19E-02 |
| 2490 | CTSB | -0.28 | 2.19E-02 |
| 2491 | KIAA1303 | -0.25 | 2.19E-02 |
| 2492 | ZNF770 | 0.39 | 2.19E-02 |
| 2493 | NMT1 | -0.19 | 2.19E-02 |
| 2494 | LIPG | -0.52 | 2.19E-02 |
| 2495 | PQLC2 | -0.22 | 2.19E-02 |
| 2496 | NAT10 | -0.18 | 2.19E-02 |
| 2497 | LOC145837 | 0.28 | 2.19E-02 |
| 2498 | RBP2 | 0.36 | 2.19E-02 |
| 2499 | ZNF625 | 0.21 | 2.19E-02 |
| 2500 | PCDHA12 | 0.17 | 2.19E-02 |
| 2501 | ZNF608 | -0.25 | 2.19E-02 |
| 2502 | COLEC10 | 0.24 | 2.19E-02 |
| 2503 | TMEM185A | -0.24 | 2.19E-02 |
| 2504 | MCTP2 | -0.59 | 2.19E-02 |
| 2505 | PSKH1 | -0.19 | 2.19E-02 |
| 2506 | EPM2AIP1 | -0.28 | 2.19E-02 |
| 2507 | RPL27A | 0.19 | 2.19E-02 |
| 2508 | SEZ6L2 | -0.20 | 2.19E-02 |
| 2509 | CAP2 | -0.31 | 2.19E-02 |
| 2510 | C10orf44 | 0.22 | 2.19E-02 |
| 2511 | C6orf150 | 0.22 | 2.19E-02 |
| 2512 | WDR65 | 0.33 | 2.19E-02 |
| 2513 | TMEM175 | -0.24 | 2.19E-02 |
| 2514 | MAPK8IP1 | -0.19 | 2.19E-02 |
| 2515 | LOC284930 | 0.18 | 2.19E-02 |
| 2516 | HOXA4 | 0.20 | 2.19E-02 |
| 2517 | AK1 | 0.36 | 2.19E-02 |
| 2518 | KRT2 | 0.37 | 2.19E-02 |
| 2519 | DCLRE1C | -0.18 | 2.19E-02 |
| 2520 | C3orf65 | 0.59 | 2.19E-02 |
| 2521 | MAFK | -0.20 | 2.19E-02 |
| 2522 | HELZ | -0.21 | 2.20E-02 |
| 2523 | SHMT2 | -0.19 | 2.20E-02 |
| 2524 | DST | 0.17 | 2.20E-02 |
| 2525 | FLJ36032 | 0.28 | 2.20E-02 |
| 2526 | DKFZP564M1462 | -0.34 | 2.20E-02 |
| 2527 | HERPUD1 | -0.20 | 2.20E-02 |
| 2528 | CASP2 | -0.14 | 2.20E-02 |
| 2529 | TRPV2 | -0.24 | 2.20E-02 |
| 2530 | SLC5A3 | -0.33 | 2.20E-02 |
| 2531 | SLC43A3 | -0.38 | 2.20E-02 |
| 2532 | POGZ | -0.23 | 2.21E-02 |
| 2533 | RBMY1D | 0.32 | 2.21E-02 |
| 2534 | ZNF419 | -0.29 | 2.21E-02 |
| 2535 | HDAC6 | -0.26 | 2.21E-02 |
| 2536 | FKBP2 | -0.17 | 2.21E-02 |
| 2537 | C20orf74 | 0.23 | 2.21E-02 |
| 2538 | DLL4 | -0.44 | 2.21E-02 |
| 2539 | RHCE | 0.38 | 2.21E-02 |
| 2540 | MED16 | -0.14 | 2.21E-02 |
| 2541 | TNFRSF10D | -0.48 | 2.21E-02 |
| 2542 | PDIA3 | -0.31 | 2.21E-02 |
| 2543 | GNB4 | -0.31 | 2.21E-02 |
| 2544 | HIST1H2BD | -0.40 | 2.21E-02 |
| 2545 | SLC24A5 | -0.21 | 2.21E-02 |
| 2546 | HKR1 | 0.18 | 2.21E-02 |
| 2547 | TPSB2 | -0.38 | 2.21E-02 |
| 2548 | IDH1 | 0.20 | 2.21E-02 |
| 2549 | ABCF1 | -0.15 | 2.21E-02 |
| 2550 | RUVBL1 | -0.23 | 2.21E-02 |
| 2551 | RPL30 | 0.26 | 2.22E-02 |
| 2552 | PCA3 | 0.28 | 2.22E-02 |
| 2553 | SETD6 | -0.39 | 2.22E-02 |
| 2554 | OR2W3 | 0.53 | 2.22E-02 |
| 2555 | PTPRE | -0.28 | 2.22E-02 |
| 2556 | SLC7A11 | -0.22 | 2.22E-02 |
| 2557 | CRP | 0.32 | 2.22E-02 |
| 2558 | KIAA0265 | -0.19 | 2.22E-02 |
| 2559 | NIPBL | -0.22 | 2.22E-02 |
| 2560 | OR5D18 | 0.30 | 2.22E-02 |
| 2561 | KRT86 | 0.36 | 2.22E-02 |
| 2562 | GPR89B | -0.31 | 2.22E-02 |
| 2563 | HK1 | -0.20 | 2.22E-02 |
| 2564 | WDR63 | 0.36 | 2.22E-02 |
| 2565 | RRAS2 | -0.35 | 2.22E-02 |
| 2566 | RAB13 | 0.22 | 2.22E-02 |
| 2567 | C6orf25 | 0.18 | 2.23E-02 |
| 2568 | C15orf23 | -0.20 | 2.23E-02 |
| 2569 | CATSPER2 | -0.29 | 2.23E-02 |
| 2570 | SSX2 | -0.32 | 2.23E-02 |
| 2571 | RPL13A | 0.18 | 2.23E-02 |
| 2572 | NR6A1 | 0.12 | 2.23E-02 |
| 2573 | SP4 | -0.35 | 2.23E-02 |
| 2574 | RAB21 | -0.31 | 2.23E-02 |
| 2575 | ACADVL | -0.27 | 2.23E-02 |
| 2576 | EPN1 | -0.14 | 2.23E-02 |
| 2577 | RAB3C | 0.32 | 2.23E-02 |
| 2578 | DLEU2 | 0.20 | 2.24E-02 |
| 2579 | MCTP2 | -0.25 | 2.24E-02 |
| 2580 | C2orf53 | 0.23 | 2.24E-02 |
| 2581 | PRDM2 | 0.15 | 2.24E-02 |
| 2582 | MARK1 | -0.40 | 2.24E-02 |
| 2583 | SYS1 | 0.18 | 2.24E-02 |
| 2584 | RETSAT | -0.25 | 2.24E-02 |
| 2585 | HUS1 | 0.20 | 2.24E-02 |
| 2586 | LARS2 | -0.16 | 2.24E-02 |
| 2587 | ABHD2 | -0.34 | 2.24E-02 |
| 2588 | ZXDC | -0.27 | 2.24E-02 |
| 2589 | TNRC6A | -0.29 | 2.24E-02 |
| 2590 | OR2J3 | 0.39 | 2.25E-02 |
| 2591 | C14orf23 | 0.22 | 2.25E-02 |
| 2592 | OR51M1 | 0.38 | 2.25E-02 |
| 2593 | TRIM24 | -0.22 | 2.25E-02 |
| 2594 | BAIAP3 | 0.22 | 2.25E-02 |
| 2595 | CXYorf8 | -0.15 | 2.25E-02 |
| 2596 | DEFB119 | 0.14 | 2.25E-02 |
| 2597 | ORM2 | -0.23 | 2.25E-02 |
| 2598 | BRS3 | 0.39 | 2.25E-02 |
| 2599 | ENG | -0.20 | 2.25E-02 |
| 2600 | EXDL1 | -0.14 | 2.25E-02 |
| 2601 | PTPN6 | -0.12 | 2.25E-02 |
| 2602 | FAM21C | 0.38 | 2.25E-02 |
| 2603 | DLK1 | 0.40 | 2.25E-02 |
| 2604 | hCG_1814486 | -0.21 | 2.25E-02 |
| 2605 | LRRC33 | -0.41 | 2.25E-02 |
| 2606 | KRT19P2 | -0.29 | 2.25E-02 |
| 2607 | DPF2 | -0.19 | 2.25E-02 |
| 2608 | PIK3IP1 | -0.36 | 2.25E-02 |
| 2609 | CPB1 | 0.35 | 2.25E-02 |
| 2610 | OR2C1 | 0.17 | 2.25E-02 |
| 2611 | H2AFX | -0.18 | 2.25E-02 |
| 2612 | TCEA2 | -0.17 | 2.26E-02 |
| 2613 | CNGB3 | 0.27 | 2.26E-02 |
| 2614 | UBAP1 | -0.20 | 2.26E-02 |
| 2615 | OR4E2 | 0.25 | 2.26E-02 |
| 2616 | PRDM11 | 0.23 | 2.26E-02 |
| 2617 | hCG_1983332 | 0.24 | 2.26E-02 |
| 2618 | NDST2 | -0.25 | 2.26E-02 |
| 2619 | FJX1 | 0.35 | 2.26E-02 |
| 2620 | DUX3 | 0.23 | 2.26E-02 |
| 2621 | ST3GAL5 | -0.41 | 2.26E-02 |
| 2622 | TMEM34 | 0.21 | 2.26E-02 |
| 2623 | RBM4B | 0.14 | 2.26E-02 |
| 2624 | EPYC | 0.21 | 2.26E-02 |
| 2625 | C22orf26 | 0.23 | 2.26E-02 |
| 2626 | CLEC11A | -0.28 | 2.26E-02 |
| 2627 | HDAC2 | 0.21 | 2.26E-02 |
| 2628 | GLT6D1 | 0.32 | 2.26E-02 |
| 2629 | RNF26 | -0.18 | 2.26E-02 |
| 2630 | COL25A1 | 0.20 | 2.26E-02 |
| 2631 | OR5H1 | 0.32 | 2.26E-02 |
| 2632 | LOC146346 | -0.34 | 2.27E-02 |
| 2633 | S100G | 0.26 | 2.27E-02 |
| 2634 | LOC399959 | 0.33 | 2.27E-02 |
| 2635 | PNMT | 0.21 | 2.27E-02 |
| 2636 | ELMO2 | -0.26 | 2.27E-02 |
| 2637 | MGC50559 | 0.34 | 2.27E-02 |
| 2638 | STT3A | -0.20 | 2.27E-02 |
| 2639 | WDR1 | -0.30 | 2.27E-02 |
| 2640 | NDUFA1 | 0.17 | 2.27E-02 |
| 2641 | CHRNA6 | 0.33 | 2.27E-02 |
| 2642 | MAS1L | 0.60 | 2.27E-02 |
| 2643 | SLC34A1 | 0.27 | 2.27E-02 |
| 2644 | PPRC1 | -0.16 | 2.27E-02 |
| 2645 | CPD | -0.33 | 2.27E-02 |
| 2646 | BAT2D1 | -0.26 | 2.27E-02 |
| 2647 | ADAMTSL2 | -0.18 | 2.27E-02 |
| 2648 | CREB3L2 | -0.23 | 2.27E-02 |
| 2649 | FAM105B | -0.36 | 2.28E-02 |
| 2650 | CHDH | -0.19 | 2.28E-02 |
| 2651 | LOC645676 | -0.26 | 2.28E-02 |
| 2652 | NPHS1 | 0.29 | 2.28E-02 |
| 2653 | APOOL | -0.22 | 2.28E-02 |
| 2654 | TAC1 | 0.31 | 2.28E-02 |
| 2655 | DTNB | 0.27 | 2.28E-02 |
| 2656 | FAM23B | 0.16 | 2.28E-02 |
| 2657 | MC1R | 0.35 | 2.28E-02 |
| 2658 | IQCF2 | -0.17 | 2.28E-02 |
| 2659 | SERPINE2 | -0.24 | 2.28E-02 |
| 2660 | LOC728442 | 0.17 | 2.28E-02 |
| 2661 | DLL3 | 0.16 | 2.28E-02 |
| 2662 | DNAH3 | 0.40 | 2.29E-02 |
| 2663 | MFHAS1 | -0.12 | 2.29E-02 |
| 2664 | C20orf94 | 0.40 | 2.29E-02 |
| 2665 | LPAL2 | 0.13 | 2.29E-02 |
| 2666 | RGS3 | -0.18 | 2.29E-02 |
| 2667 | CD97 | -0.30 | 2.29E-02 |
| 2668 | SLC9A6 | -0.27 | 2.29E-02 |
| 2669 | KIRREL3 | 0.27 | 2.29E-02 |
| 2670 | HYAL3 | -0.24 | 2.29E-02 |
| 2671 | OAS3 | 0.15 | 2.30E-02 |
| 2672 | C10orf49 | 0.23 | 2.30E-02 |
| 2673 | B3GALT6 | -0.18 | 2.30E-02 |
| 2674 | STK36 | -0.27 | 2.30E-02 |
| 2675 | CRP | 0.33 | 2.30E-02 |
| 2676 | C1orf218 | -0.41 | 2.30E-02 |
| 2677 | HLF | 0.16 | 2.30E-02 |
| 2678 | NBEAL2 | 0.21 | 2.30E-02 |
| 2679 | SMCR6 | -0.17 | 2.30E-02 |
| 2680 | ALG9 | -0.27 | 2.30E-02 |
| 2681 | FAM83C | 0.22 | 2.31E-02 |
| 2682 | TTYH2 | -0.27 | 2.31E-02 |
| 2683 | C17orf81 | -0.20 | 2.31E-02 |
| 2684 | COX6C | 0.20 | 2.31E-02 |
| 2685 | ARGFX | 0.37 | 2.31E-02 |
| 2686 | KRTAP12-4 | 0.19 | 2.31E-02 |
| 2687 | CD4 | 0.23 | 2.31E-02 |
| 2688 | PDE4C | 0.28 | 2.31E-02 |
| 2689 | KLHDC6 | 0.25 | 2.31E-02 |
| 2690 | SNX19 | 0.29 | 2.31E-02 |
| 2691 | ACVR1B | -0.37 | 2.31E-02 |
| 2692 | FRMPD3 | 0.36 | 2.31E-02 |
| 2693 | TINAGL1 | -0.42 | 2.31E-02 |
| 2694 | FIS1 | -0.35 | 2.31E-02 |
| 2695 | MCM4 | -0.17 | 2.31E-02 |
| 2696 | GPR19 | -0.13 | 2.32E-02 |
| 2697 | MICALL2 | -0.30 | 2.32E-02 |
| 2698 | FAT3 | -0.37 | 2.32E-02 |
| 2699 | BAGE5 | -0.21 | 2.32E-02 |
| 2700 | UROC1 | 0.20 | 2.32E-02 |
| 2701 | ALG12 | -0.22 | 2.32E-02 |
| 2702 | CYP27B1 | 0.17 | 2.32E-02 |
| 2703 | YIPF7 | 0.14 | 2.32E-02 |
| 2704 | TNFRSF9 | 0.36 | 2.32E-02 |
| 2705 | GIT2 | 0.34 | 2.33E-02 |
| 2706 | NCF1 | -0.14 | 2.33E-02 |
| 2707 | TCP11 | 0.30 | 2.33E-02 |
| 2708 | AQR | -0.35 | 2.33E-02 |
| 2709 | PDPK1 | -0.28 | 2.33E-02 |
| 2710 | TMC6 | -0.16 | 2.33E-02 |
| 2711 | SETD1A | -0.18 | 2.33E-02 |
| 2712 | TNFRSF1A | -0.16 | 2.33E-02 |
| 2713 | TJAP1 | -0.25 | 2.33E-02 |
| 2714 | NRXN3 | -0.43 | 2.33E-02 |
| 2715 | AHCYL1 | -0.30 | 2.33E-02 |
| 2716 | ADH4 | 0.40 | 2.33E-02 |
| 2717 | RAD51L1 | 0.46 | 2.33E-02 |
| 2718 | P4HA2 | -0.19 | 2.33E-02 |
| 2719 | P2RX7 | 0.19 | 2.33E-02 |
| 2720 | SUOX | -0.21 | 2.33E-02 |
| 2721 | ASB2 | 0.17 | 2.33E-02 |
| 2722 | KIAA0329 | -0.27 | 2.33E-02 |
| 2723 | ATP2C1 | -0.32 | 2.33E-02 |
| 2724 | ADAMTS14 | 0.36 | 2.33E-02 |
| 2725 | PSG6 | -0.22 | 2.33E-02 |
| 2726 | ZNF342 | 0.38 | 2.34E-02 |
| 2727 | TBCD | -0.15 | 2.34E-02 |
| 2728 | LTB4R2 | 0.24 | 2.34E-02 |
| 2729 | KIAA0152 | -0.27 | 2.34E-02 |
| 2730 | BZRPL1 | 0.31 | 2.34E-02 |
| 2731 | MRPL43 | -0.16 | 2.34E-02 |
| 2732 | FKBP11 | -0.24 | 2.34E-02 |
| 2733 | P76 | -0.11 | 2.34E-02 |
| 2734 | TAGLN3 | 0.34 | 2.34E-02 |
| 2735 | ATP6V1A | -0.20 | 2.34E-02 |
| 2736 | MBOAT5 | -0.24 | 2.34E-02 |
| 2737 | C6orf120 | -0.22 | 2.34E-02 |
| 2738 | GCET2 | 0.20 | 2.34E-02 |
| 2739 | PRKAG2 | -0.17 | 2.34E-02 |
| 2740 | STAB2 | 0.27 | 2.35E-02 |
| 2741 | KRTAP3-1 | -0.26 | 2.35E-02 |
| 2742 | UNQ6411 | 0.20 | 2.35E-02 |
| 2743 | EGLN3 | 0.22 | 2.35E-02 |
| 2744 | OR4D10 | 0.27 | 2.35E-02 |
| 2745 | SHANK2 | 0.14 | 2.35E-02 |
| 2746 | MALAT1///PRO1073 | 0.18 | 2.35E-02 |
| 2747 | SNORA68 | -0.47 | 2.35E-02 |
| 2748 | OFCC1 | 0.20 | 2.36E-02 |
| 2749 | SLC39A4 | -0.18 | 2.36E-02 |
| 2750 | CUX2 | -0.26 | 2.36E-02 |
| 2751 | TMEM118 | -0.22 | 2.36E-02 |
| 2752 | SYK | -0.51 | 2.36E-02 |
| 2753 | CNR1 | -0.26 | 2.36E-02 |
| 2754 | POMGNT1 | -0.19 | 2.36E-02 |
| 2755 | GLIPR1L1 | 0.22 | 2.36E-02 |
| 2756 | RSL1D1 | -0.32 | 2.36E-02 |
| 2757 | LOC143678 | 0.21 | 2.36E-02 |
| 2758 | TRIM38 | -0.27 | 2.36E-02 |
| 2759 | UPK1A | 0.35 | 2.36E-02 |
| 2760 | TP53RK | 0.24 | 2.36E-02 |
| 2761 | KIAA1267 | -0.19 | 2.36E-02 |
| 2762 | ATF4 | -0.23 | 2.36E-02 |
| 2763 | KIAA1804 | -0.22 | 2.36E-02 |
| 2764 | SLC22A6 | 0.20 | 2.36E-02 |
| 2765 | BACE2 | -0.32 | 2.36E-02 |
| 2766 | UBC///SCARB1 | -0.20 | 2.37E-02 |
| 2767 | LHX6 | -0.20 | 2.37E-02 |
| 2768 | INS | 0.27 | 2.37E-02 |
| 2769 | PSG5 | 0.23 | 2.37E-02 |
| 2770 | SAV1 | 0.63 | 2.37E-02 |
| 2771 | MYST2 | -0.16 | 2.37E-02 |
| 2772 | A3GALT2 | 0.13 | 2.37E-02 |
| 2773 | IKBKG | -0.14 | 2.37E-02 |
| 2774 | KRTAP4-9 | 0.23 | 2.37E-02 |
| 2775 | OR1D2 | 0.29 | 2.37E-02 |
| 2776 | ZNF432 | -0.49 | 2.37E-02 |
| 2777 | COX11 | 0.21 | 2.37E-02 |
| 2778 | CD320 | -0.14 | 2.37E-02 |
| 2779 | ZNF385 | 0.20 | 2.38E-02 |
| 2780 | OR51F1 | 0.19 | 2.38E-02 |
| 2781 | C10orf88 | -0.36 | 2.38E-02 |
| 2782 | CCND3 | -0.28 | 2.38E-02 |
| 2783 | SEMA3A | -0.27 | 2.39E-02 |
| 2784 | CTSF | -0.21 | 2.39E-02 |
| 2785 | SPATA12 | -0.15 | 2.39E-02 |
| 2786 | DTNA | 0.27 | 2.39E-02 |
| 2787 | KCNJ2 | -0.45 | 2.39E-02 |
| 2788 | C18orf57 | 0.21 | 2.39E-02 |
| 2789 | TMEM41A | -0.19 | 2.39E-02 |
| 2790 | HS2ST1 | -0.18 | 2.39E-02 |
| 2791 | SATB1 | 0.31 | 2.39E-02 |
| 2792 | FAM148A | -0.31 | 2.39E-02 |
| 2793 | CAMKK2 | 0.34 | 2.39E-02 |
| 2794 | C6orf15 | 0.22 | 2.39E-02 |
| 2795 | PANK2 | -0.17 | 2.39E-02 |
| 2796 | EFR3B | 0.41 | 2.39E-02 |
| 2797 | FGFR1OP | 0.40 | 2.39E-02 |
| 2798 | ATP5L2 | 0.17 | 2.40E-02 |
| 2799 | MAPK10 | 0.39 | 2.40E-02 |
| 2800 | OR2M1P | 0.31 | 2.40E-02 |
| 2801 | ZNF773 | -0.30 | 2.40E-02 |
| 2802 | ARID3C | 0.25 | 2.40E-02 |
| 2803 | APBA2BP | -0.19 | 2.40E-02 |
| 2804 | SPINT2 | -0.33 | 2.40E-02 |
| 2805 | SEMA4B | 0.48 | 2.40E-02 |
| 2806 | ETV2 | 0.19 | 2.40E-02 |
| 2807 | DCP1A | -0.28 | 2.40E-02 |
| 2808 | SPAG16 | 0.18 | 2.40E-02 |
| 2809 | SLC16A7 | -0.25 | 2.40E-02 |
| 2810 | TNS3 | 0.24 | 2.40E-02 |
| 2811 | CAMKK2 | 0.33 | 2.40E-02 |
| 2812 | HIST2H2BE | -0.23 | 2.40E-02 |
| 2813 | SPEN | -0.18 | 2.40E-02 |
| 2814 | HDDC2 | 0.40 | 2.40E-02 |
| 2815 | VAMP1 | -0.18 | 2.40E-02 |
| 2816 | ADAMTS1 | -0.28 | 2.40E-02 |
| 2817 | PRDM14 | 0.36 | 2.41E-02 |
| 2818 | LZTR1 | -0.19 | 2.41E-02 |
| 2819 | FZD2 | -0.30 | 2.41E-02 |
| 2820 | NUDT10 | 0.38 | 2.41E-02 |
| 2821 | MMAB | -0.21 | 2.41E-02 |
| 2822 | OR9Q1 | 0.57 | 2.41E-02 |
| 2823 | VPS39 | -0.20 | 2.41E-02 |
| 2824 | SLC14A2 | 0.19 | 2.41E-02 |
| 2825 | FNBP4 | -0.18 | 2.42E-02 |
| 2826 | PSME4 | -0.25 | 2.42E-02 |
| 2827 | CHRM2 | 0.31 | 2.42E-02 |
| 2828 | SNORA66 | -0.23 | 2.42E-02 |
| 2829 | PIGO | -0.21 | 2.42E-02 |
| 2830 | LDOC1L | 0.16 | 2.42E-02 |
| 2831 | CRK | -0.26 | 2.42E-02 |
| 2832 | DTX3L | 0.51 | 2.42E-02 |
| 2833 | KLK6 | 0.37 | 2.42E-02 |
| 2834 | ABCB4 | -0.35 | 2.42E-02 |
| 2835 | UBR5 | -0.24 | 2.42E-02 |
| 2836 | UGCGL2 | -0.21 | 2.42E-02 |
| 2837 | MMP16 | 0.34 | 2.42E-02 |
| 2838 | SLC22A1 | 0.22 | 2.42E-02 |
| 2839 | TNPO1 | -0.29 | 2.43E-02 |
| 2840 | LETM1 | -0.16 | 2.43E-02 |
| 2841 | TRAF3IP3 | 0.37 | 2.43E-02 |
| 2842 | PTPN1 | -0.22 | 2.43E-02 |
| 2843 | LOC147093 | 0.26 | 2.43E-02 |
| 2844 | TCIRG1 | -0.28 | 2.43E-02 |
| 2845 | OR51V1 | 0.37 | 2.43E-02 |
| 2846 | DEFA1 | 0.34 | 2.43E-02 |
| 2847 | LOC648210///LOC644037 | 0.25 | 2.43E-02 |
| 2848 | NACA | 0.25 | 2.43E-02 |
| 2849 | C21orf62 | 0.13 | 2.43E-02 |
| 2850 | XPNPEP2 | 0.19 | 2.44E-02 |
| 2851 | EIF5A | -0.42 | 2.44E-02 |
| 2852 | LONP2///SIAH1 | -0.24 | 2.44E-02 |
| 2853 | STIL | -0.15 | 2.44E-02 |
| 2854 | STEAP4 | -0.31 | 2.44E-02 |
| 2855 | LOC648343 | -0.12 | 2.44E-02 |
| 2856 | OR3A3 | 0.29 | 2.44E-02 |
| 2857 | FOXA1 | -0.36 | 2.44E-02 |
| 2858 | BHMT2 | 0.46 | 2.44E-02 |
| 2859 | OR5T2 | -0.12 | 2.44E-02 |
| 2860 | TSHZ1 | -0.22 | 2.44E-02 |
| 2861 | LASS6 | -0.38 | 2.44E-02 |
| 2862 | FLJ34077 | 0.27 | 2.44E-02 |
| 2863 | RP13-15M17.2 | -0.26 | 2.44E-02 |
| 2864 | ATP10B | -0.43 | 2.44E-02 |
| 2865 | C3orf39 | -0.25 | 2.44E-02 |
| 2866 | STK19 | -0.22 | 2.45E-02 |
| 2867 | APBA1 | 0.27 | 2.45E-02 |
| 2868 | LOC283089 | 0.15 | 2.45E-02 |
| 2869 | C16orf71 | 0.26 | 2.46E-02 |
| 2870 | OR2G3 | 0.29 | 2.46E-02 |
| 2871 | HSD11B1L | -0.20 | 2.46E-02 |
| 2872 | NUDCD3 | -0.21 | 2.46E-02 |
| 2873 | DDI2 | -0.12 | 2.46E-02 |
| 2874 | PARD3B | 0.27 | 2.46E-02 |
| 2875 | EPO | 0.18 | 2.46E-02 |
| 2876 | PURG | 0.16 | 2.47E-02 |
| 2877 | CYBASC3 | -0.22 | 2.47E-02 |
| 2878 | PPP2R1B | -0.38 | 2.47E-02 |
| 2879 | KLHL4 | 0.14 | 2.47E-02 |
| 2880 | LOC341112 | 0.17 | 2.47E-02 |
| 2881 | COL4A1 | -0.50 | 2.47E-02 |
| 2882 | DUOX1 | 0.21 | 2.47E-02 |
| 2883 | KAAG1 | 0.26 | 2.47E-02 |
| 2884 | WFDC5 | 0.25 | 2.47E-02 |
| 2885 | TRPM3 | 0.12 | 2.47E-02 |
| 2886 | C1S | -0.42 | 2.48E-02 |
| 2887 | NPM1 | 0.29 | 2.48E-02 |
| 2888 | POLE | -0.11 | 2.48E-02 |
| 2889 | KIR2DL2 | 0.22 | 2.48E-02 |
| 2890 | CDC14B | -0.33 | 2.48E-02 |
| 2891 | DNAJC5G | 0.21 | 2.48E-02 |
| 2892 | ZMYM6 | -0.17 | 2.48E-02 |
| 2893 | RPS27 | 0.20 | 2.48E-02 |
| 2894 | RNASE11 | 0.20 | 2.48E-02 |
| 2895 | PIK3CD | -0.35 | 2.48E-02 |
| 2896 | FUT5 | 0.15 | 2.48E-02 |
| 2897 | CDC42EP3 | 0.26 | 2.48E-02 |
| 2898 | LOC728171 | 0.28 | 2.48E-02 |
| 2899 | HS3ST5 | 0.24 | 2.48E-02 |
| 2900 | CCDC75 | 0.28 | 2.49E-02 |
| 2901 | LOC283174 | -0.12 | 2.49E-02 |
| 2902 | RPL31 | 0.24 | 2.49E-02 |
| 2903 | CNTN1 | 0.11 | 2.49E-02 |
| 2904 | ERMP1 | -0.35 | 2.49E-02 |
| 2905 | NXNL2 | -0.16 | 2.49E-02 |
| 2906 | OR4F5 | 0.26 | 2.49E-02 |
| 2907 | TBC1D13 | -0.29 | 2.49E-02 |
| 2908 | SERPINA6 | -0.27 | 2.49E-02 |
| 2909 | NQO2 | -0.27 | 2.49E-02 |
| 2910 | EFCAB5 | 0.16 | 2.49E-02 |
| 2911 | MRPS12 | 0.21 | 2.49E-02 |
| 2912 | SLC7A5 | -0.22 | 2.49E-02 |
| 2913 | YAP1 | -0.16 | 2.49E-02 |
| 2914 | SLC4A8 | -0.28 | 2.49E-02 |
| 2915 | ANKRD34 | 0.31 | 2.49E-02 |
| 2916 | DBNDD1 | -0.28 | 2.49E-02 |
| 2917 | GAA | -0.19 | 2.49E-02 |
| 2918 | HUS1B | 0.22 | 2.50E-02 |
| 2919 | ATXN2 | -0.28 | 2.50E-02 |
| 2920 | F5 | -0.43 | 2.50E-02 |
| 2921 | HSPB7 | -0.16 | 2.50E-02 |
| 2922 | LOC284100 | -0.32 | 2.50E-02 |
| 2923 | MRPL39 | 0.23 | 2.50E-02 |
| 2924 | ZNF791 | -0.38 | 2.50E-02 |
| 2925 | CSF2RA | -0.27 | 2.50E-02 |
| 2926 | NAP1L6 | 0.20 | 2.50E-02 |
| 2927 | SLC39A5 | -0.30 | 2.50E-02 |
| 2928 | TAPBPL | -0.29 | 2.50E-02 |
| 2929 | COL5A3 | -0.47 | 2.50E-02 |
| 2930 | GPR173 | 0.26 | 2.50E-02 |
| 2931 | VIPR1 | 0.18 | 2.50E-02 |
| 2932 | GPR21 | 0.28 | 2.50E-02 |
| 2933 | LOC645478 | 0.35 | 2.50E-02 |
| 2934 | AMIGO3 | 0.14 | 2.50E-02 |
| 2935 | NCOA3 | -0.37 | 2.50E-02 |
| 2936 | OPN1MW | 0.26 | 2.51E-02 |
| 2937 | LCORL | -0.36 | 2.51E-02 |
| 2938 | NIPA1 | -0.20 | 2.51E-02 |
| 2939 | ZNF460 | -0.30 | 2.52E-02 |
| 2940 | ATP1A1 | -0.26 | 2.52E-02 |
| 2941 | PNPLA7 | -0.19 | 2.52E-02 |
| 2942 | WBSCR19 | 0.34 | 2.52E-02 |
| 2943 | GPR135 | 0.23 | 2.52E-02 |
| 2944 | CYP27B1 | 0.17 | 2.52E-02 |
| 2945 | MRPL1 | 0.26 | 2.52E-02 |
| 2946 | GSTM4 | 0.28 | 2.52E-02 |
| 2947 | PHC2 | 0.28 | 2.52E-02 |
| 2948 | KCNJ16 | 0.18 | 2.52E-02 |
| 2949 | C14orf82 | -0.33 | 2.52E-02 |
| 2950 | ARHGDIA | -0.16 | 2.52E-02 |
| 2951 | SEC61A1 | -0.23 | 2.52E-02 |
| 2952 | IL4 | 0.65 | 2.52E-02 |
| 2953 | DDX46 | 0.21 | 2.53E-02 |
| 2954 | FAM129C | 0.20 | 2.53E-02 |
| 2955 | GC | 0.29 | 2.53E-02 |
| 2956 | SLCO2A1 | -0.37 | 2.53E-02 |
| 2957 | KIAA1409 | 0.20 | 2.53E-02 |
| 2958 | FLJ12993 | -0.24 | 2.54E-02 |
| 2959 | IGF2BP2 | -0.25 | 2.54E-02 |
| 2960 | ALG10 | -0.42 | 2.54E-02 |
| 2961 | METT11D1 | -0.18 | 2.54E-02 |
| 2962 | IL28RA | -0.41 | 2.54E-02 |
| 2963 | SGSM1 | 0.30 | 2.54E-02 |
| 2964 | LOC644192 | -0.36 | 2.54E-02 |
| 2965 | GTF3C4 | -0.23 | 2.54E-02 |
| 2966 | HSPC171 | -0.23 | 2.54E-02 |
| 2967 | HERC2 | -0.31 | 2.54E-02 |
| 2968 | C21orf34 | -0.36 | 2.54E-02 |
| 2969 | N4BP1 | -0.16 | 2.54E-02 |
| 2970 | DEFB127 | 0.33 | 2.54E-02 |
| 2971 | KIF1C | 0.16 | 2.54E-02 |
| 2972 | HIST2H2AB | 0.33 | 2.54E-02 |
| 2973 | MARCH1 | 0.25 | 2.55E-02 |
| 2974 | SP140 | 0.16 | 2.55E-02 |
| 2975 | OR51B2 | 0.26 | 2.55E-02 |
| 2976 | PKD2 | -0.32 | 2.55E-02 |
| 2977 | ITGB1 | -0.36 | 2.55E-02 |
| 2978 | CHST11 | -0.15 | 2.56E-02 |
| 2979 | SREBF2 | -0.21 | 2.56E-02 |
| 2980 | WISP1 | 0.21 | 2.56E-02 |
| 2981 | LOC283129 | -0.20 | 2.56E-02 |
| 2982 | USP20 | -0.12 | 2.57E-02 |
| 2983 | ZNF24 | 0.31 | 2.57E-02 |
| 2984 | FAM3A | -0.15 | 2.57E-02 |
| 2985 | PAX2 | 0.17 | 2.57E-02 |
| 2986 | PMS2L3 | 0.35 | 2.57E-02 |
| 2987 | ABCF3 | -0.16 | 2.57E-02 |
| 2988 | LOC642817 | 0.24 | 2.57E-02 |
| 2989 | NETO1 | 0.19 | 2.57E-02 |
| 2990 | ADAMTS13 | 0.36 | 2.57E-02 |
| 2991 | SLC38A3 | 0.24 | 2.57E-02 |
| 2992 | MKNK1 | -0.25 | 2.57E-02 |
| 2993 | SNORD51 | -0.19 | 2.57E-02 |
| 2994 | CACNA1B | 0.16 | 2.58E-02 |
| 2995 | PDIA2 | -0.28 | 2.58E-02 |
| 2996 | PARVA | -0.33 | 2.58E-02 |
| 2997 | POMP | 0.16 | 2.58E-02 |
| 2998 | LDHC | 0.34 | 2.58E-02 |
| 2999 | OR51D1 | 0.20 | 2.58E-02 |
| 3000 | CYP1B1 | 0.20 | 2.58E-02 |
| 3001 | CYP24A1 | -0.33 | 2.58E-02 |
| 3002 | SAP130 | -0.23 | 2.58E-02 |
| 3003 | SIRPB2 | 0.13 | 2.58E-02 |
| 3004 | PCDH7 | 0.32 | 2.58E-02 |
| 3005 | CDSN | 0.25 | 2.58E-02 |
| 3006 | TM9SF4 | -0.30 | 2.58E-02 |
| 3007 | PDGFB | -0.28 | 2.59E-02 |
| 3008 | RPSAP15 | 0.27 | 2.59E-02 |
| 3009 | CCNJL | 0.25 | 2.59E-02 |
| 3010 | LOC619208 | 0.25 | 2.59E-02 |
| 3011 | PXMP4 | 0.56 | 2.59E-02 |
| 3012 | ARX | 0.29 | 2.59E-02 |
| 3013 | NAT2 | 0.40 | 2.59E-02 |
| 3014 | C2CD3 | 0.23 | 2.59E-02 |
| 3015 | LOC145474 | -0.37 | 2.59E-02 |
| 3016 | LOC729198 | 0.43 | 2.59E-02 |
| 3017 | INSR | -0.38 | 2.59E-02 |
| 3018 | SLC4A2 | -0.19 | 2.59E-02 |
| 3019 | PEX14 | -0.26 | 2.59E-02 |
| 3020 | CD5L | 0.22 | 2.59E-02 |
| 3021 | CCNK | 0.16 | 2.59E-02 |
| 3022 | KIAA0101 | 0.28 | 2.60E-02 |
| 3023 | RXFP1 | 0.18 | 2.60E-02 |
| 3024 | FLJ45032 | -0.20 | 2.60E-02 |
| 3025 | TYK2 | -0.14 | 2.60E-02 |
| 3026 | LOC284861 | 0.25 | 2.60E-02 |
| 3027 | MEF2D | -0.18 | 2.60E-02 |
| 3028 | CABYR | -0.37 | 2.61E-02 |
| 3029 | ZNF229 | 0.22 | 2.61E-02 |
| 3030 | KIAA1045 | 0.56 | 2.61E-02 |
| 3031 | NME6 | 0.23 | 2.61E-02 |
| 3032 | CEACAM7 | 0.20 | 2.61E-02 |
| 3033 | WDR73 | -0.22 | 2.61E-02 |
| 3034 | LOC130940 | 0.18 | 2.61E-02 |
| 3035 | SLA2 | -0.39 | 2.61E-02 |
| 3036 | C1S | -0.45 | 2.61E-02 |
| 3037 | TPT1 | 0.28 | 2.61E-02 |
| 3038 | ZMYND17 | 0.22 | 2.61E-02 |
| 3039 | EBF3 | 0.13 | 2.63E-02 |
| 3040 | USP32 | 0.15 | 2.63E-02 |
| 3041 | ULK1 | 0.24 | 2.63E-02 |
| 3042 | IDUA | 0.28 | 2.63E-02 |
| 3043 | ELFN2 | 0.26 | 2.63E-02 |
| 3044 | GSTCD | -0.39 | 2.63E-02 |
| 3045 | MRPL45 | 0.17 | 2.63E-02 |
| 3046 | INADL | -0.11 | 2.63E-02 |
| 3047 | NAT11 | 0.18 | 2.63E-02 |
| 3048 | SLC35E1 | -0.29 | 2.63E-02 |
| 3049 | ZIK1 | -0.18 | 2.63E-02 |
| 3050 | PMPCB | -0.32 | 2.63E-02 |
| 3051 | HSFX1 | -0.36 | 2.63E-02 |
| 3052 | MLN | 0.12 | 2.63E-02 |
| 3053 | FAM19A4 | 0.66 | 2.63E-02 |
| 3054 | USP2 | 0.18 | 2.63E-02 |
| 3055 | JAK1 | -0.17 | 2.63E-02 |
| 3056 | IFNA13 | 0.46 | 2.63E-02 |
| 3057 | THSD4 | -0.41 | 2.63E-02 |
| 3058 | NAG | 0.55 | 2.63E-02 |
| 3059 | SLC12A4 | -0.21 | 2.63E-02 |
| 3060 | XPOT | -0.19 | 2.63E-02 |
| 3061 | WDR61 | 0.18 | 2.63E-02 |
| 3062 | ICEBERG | 0.28 | 2.64E-02 |
| 3063 | LOC497256 | 0.19 | 2.64E-02 |
| 3064 | WRN | 0.20 | 2.64E-02 |
| 3065 | ZNF514 | -0.37 | 2.64E-02 |
| 3066 | WDR24 | -0.13 | 2.64E-02 |
| 3067 | LOC92270 | -0.17 | 2.64E-02 |
| 3068 | RPS28 | 0.18 | 2.64E-02 |
| 3069 | MTO1 | -0.31 | 2.64E-02 |
| 3070 | BMF | -0.26 | 2.64E-02 |
| 3071 | KIF1A | 0.19 | 2.64E-02 |
| 3072 | SPOP | 0.27 | 2.64E-02 |
| 3073 | PALM2 | 0.34 | 2.64E-02 |
| 3074 | TRIM32 | -0.22 | 2.65E-02 |
| 3075 | PER1 | -0.24 | 2.65E-02 |
| 3076 | NLRP12 | -0.13 | 2.65E-02 |
| 3077 | LOC388248 | -0.12 | 2.65E-02 |
| 3078 | PGLYRP2 | 0.23 | 2.65E-02 |
| 3079 | C11orf66 | 0.22 | 2.65E-02 |
| 3080 | RCN1 | -0.31 | 2.65E-02 |
| 3081 | STRN | 0.24 | 2.65E-02 |
| 3082 | LOC339803 | -0.13 | 2.65E-02 |
| 3083 | CATSPER4 | 0.21 | 2.65E-02 |
| 3084 | PTPRG | -0.22 | 2.65E-02 |
| 3085 | DUSP27 | 0.20 | 2.65E-02 |
| 3086 | MPZL1 | -0.33 | 2.65E-02 |
| 3087 | SLC22A15 | -0.39 | 2.65E-02 |
| 3088 | ABHD4 | -0.17 | 2.65E-02 |
| 3089 | OTUD6A | 0.36 | 2.65E-02 |
| 3090 | ENTPD4 | -0.12 | 2.65E-02 |
| 3091 | SARM1 | 0.14 | 2.65E-02 |
| 3092 | PDZD7 | 0.27 | 2.65E-02 |
| 3093 | OR5K3 | 0.45 | 2.65E-02 |
| 3094 | SLC25A36 | -0.22 | 2.65E-02 |
| 3095 | THPO | 0.37 | 2.65E-02 |
| 3096 | FGF10 | 0.25 | 2.65E-02 |
| 3097 | LALBA | 0.13 | 2.66E-02 |
| 3098 | GGA3 | -0.20 | 2.66E-02 |
| 3099 | C21orf118 | 0.29 | 2.66E-02 |
| 3100 | SHB | -0.12 | 2.66E-02 |
| 3101 | GOLGA2L1 | -0.27 | 2.66E-02 |
| 3102 | MMAA | 0.34 | 2.66E-02 |
| 3103 | FGR | 0.32 | 2.66E-02 |
| 3104 | IGKV1D-8 | 0.21 | 2.66E-02 |
| 3105 | DHCR24 | -0.26 | 2.66E-02 |
| 3106 | DAAM1 | -0.51 | 2.66E-02 |
| 3107 | ZNF333 | 0.31 | 2.66E-02 |
| 3108 | ZNHIT4 | -0.14 | 2.66E-02 |
| 3109 | RICH2 | 0.41 | 2.66E-02 |
| 3110 | EXT2 | -0.41 | 2.66E-02 |
| 3111 | C1orf95 | -0.10 | 2.67E-02 |
| 3112 | LOC728953 | 0.20 | 2.67E-02 |
| 3113 | MASTL | -0.34 | 2.67E-02 |
| 3114 | PRAP1 | 0.22 | 2.67E-02 |
| 3115 | LMAN1L | 0.17 | 2.67E-02 |
| 3116 | ZBTB34 | -0.37 | 2.67E-02 |
| 3117 | CYP2S1 | -0.38 | 2.67E-02 |
| 3118 | KLK13 | 0.24 | 2.67E-02 |
| 3119 | RP11-216N14.7 | 0.20 | 2.67E-02 |
| 3120 | KIAA1009 | 0.54 | 2.67E-02 |
| 3121 | PODXL | -0.30 | 2.67E-02 |
| 3122 | OR51G1 | 0.28 | 2.67E-02 |
| 3123 | OFD1 | 0.53 | 2.67E-02 |
| 3124 | TNNI3 | -0.18 | 2.67E-02 |
| 3125 | CXCL12 | 0.16 | 2.67E-02 |
| 3126 | AIPL1 | 0.22 | 2.67E-02 |
| 3127 | UGT2A1 | 0.26 | 2.67E-02 |
| 3128 | GALT | -0.24 | 2.68E-02 |
| 3129 | hCG_2041321 | 0.12 | 2.68E-02 |
| 3130 | REXO1 | 0.33 | 2.68E-02 |
| 3131 | CST3 | -0.25 | 2.68E-02 |
| 3132 | XCR1 | 0.15 | 2.68E-02 |
| 3133 | CORIN | -0.41 | 2.68E-02 |
| 3134 | SMG6 | 0.37 | 2.68E-02 |
| 3135 | PIAS1 | -0.20 | 2.68E-02 |
| 3136 | CLCN7 | -0.20 | 2.68E-02 |
| 3137 | IKZF5 | 0.29 | 2.68E-02 |
| 3138 | SLC39A1 | -0.23 | 2.68E-02 |
| 3139 | TRIM40 | 0.13 | 2.68E-02 |
| 3140 | SLC37A4 | -0.24 | 2.68E-02 |
| 3141 | VAMP1 | -0.21 | 2.68E-02 |
| 3142 | KRTAP19-4 | 0.23 | 2.68E-02 |
| 3143 | C5orf25 | 0.21 | 2.68E-02 |
| 3144 | SCARA3 | 0.18 | 2.68E-02 |
| 3145 | C1orf85 | -0.30 | 2.68E-02 |
| 3146 | THSD7A | -0.31 | 2.68E-02 |
| 3147 | ASAHL | -0.20 | 2.68E-02 |
| 3148 | PBX1 | -0.26 | 2.69E-02 |
| 3149 | ATRIP | 0.15 | 2.69E-02 |
| 3150 | CIB3 | -0.09 | 2.69E-02 |
| 3151 | NR0B1 | 0.17 | 2.69E-02 |
| 3152 | TIMP2 | -0.21 | 2.69E-02 |
| 3153 | PFN3 | 0.13 | 2.69E-02 |
| 3154 | PLCB1 | -0.22 | 2.69E-02 |
| 3155 | PDE4A | 0.23 | 2.69E-02 |
| 3156 | PXK | 0.24 | 2.69E-02 |
| 3157 | ZNF208 | 0.30 | 2.69E-02 |
| 3158 | TRPM3 | 0.13 | 2.69E-02 |
| 3159 | ST8SIA2 | 0.17 | 2.69E-02 |
| 3160 | UGT2A1 | 0.27 | 2.69E-02 |
| 3161 | LOC284395 | 0.21 | 2.69E-02 |
| 3162 | IMPG2 | 0.15 | 2.69E-02 |
| 3163 | HIST1H2BN | -0.20 | 2.70E-02 |
| 3164 | YIPF6 | -0.22 | 2.70E-02 |
| 3165 | TCTN1 | 0.54 | 2.70E-02 |
| 3166 | C9orf107 | 0.32 | 2.70E-02 |
| 3167 | RALGPS1 | 0.19 | 2.70E-02 |
| 3168 | LOC286123 | -0.24 | 2.70E-02 |
| 3169 | RAPGEF6 | -0.38 | 2.70E-02 |
| 3170 | SPRYD4 | -0.10 | 2.70E-02 |
| 3171 | SEMA6C | 0.21 | 2.70E-02 |
| 3172 | FDFT1 | -0.40 | 2.71E-02 |
| 3173 | KIF3C | 0.32 | 2.71E-02 |
| 3174 | PDCD6IP | -0.20 | 2.71E-02 |
| 3175 | SLC7A4 | 0.25 | 2.71E-02 |
| 3176 | C1orf50 | 0.31 | 2.71E-02 |
| 3177 | SPC24 | -0.17 | 2.71E-02 |
| 3178 | CXorf23 | 0.21 | 2.71E-02 |
| 3179 | ZDHHC5 | -0.20 | 2.71E-02 |
| 3180 | RCHY1 | 0.19 | 2.72E-02 |
| 3181 | TPM4 | -0.27 | 2.72E-02 |
| 3182 | LUZP2 | 0.27 | 2.72E-02 |
| 3183 | SDR-O | 0.22 | 2.72E-02 |
| 3184 | CDC2L5 | -0.26 | 2.72E-02 |
| 3185 | PCF11 | -0.27 | 2.72E-02 |
| 3186 | TSSK1B | 0.26 | 2.72E-02 |
| 3187 | PITPNM3 | 0.21 | 2.72E-02 |
| 3188 | PCDH17 | -0.34 | 2.72E-02 |
| 3189 | ADRA1A | 0.21 | 2.72E-02 |
| 3190 | IQSEC2 | 0.28 | 2.72E-02 |
| 3191 | CNNM3 | -0.21 | 2.72E-02 |
| 3192 | RSPH10B | 0.28 | 2.72E-02 |
| 3193 | MYF6 | 0.17 | 2.72E-02 |
| 3194 | GSX2 | 0.22 | 2.72E-02 |
| 3195 | RPL6 | 0.30 | 2.72E-02 |
| 3196 | TMEM62 | -0.26 | 2.72E-02 |
| 3197 | ACR | 0.30 | 2.72E-02 |
| 3198 | TSPAN17 | -0.23 | 2.72E-02 |
| 3199 | GDI1 | -0.19 | 2.72E-02 |
| 3200 | ZNF585A | 0.30 | 2.72E-02 |
| 3201 | GNRHR2 | 0.26 | 2.72E-02 |
| 3202 | PVALB | -0.29 | 2.72E-02 |
| 3203 | NID2 | -0.24 | 2.72E-02 |
| 3204 | TRPS1 | -0.27 | 2.72E-02 |
| 3205 | VCP | -0.17 | 2.73E-02 |
| 3206 | DPYSL5 | 0.20 | 2.73E-02 |
| 3207 | hCG_19809 | 0.23 | 2.73E-02 |
| 3208 | RBM3 | 0.28 | 2.73E-02 |
| 3209 | FAM125A | -0.12 | 2.73E-02 |
| 3210 | CCT8 | 0.21 | 2.73E-02 |
| 3211 | C20orf32 | 0.19 | 2.73E-02 |
| 3212 | PA2G4 | -0.20 | 2.73E-02 |
| 3213 | FDFT1 | 0.13 | 2.74E-02 |
| 3214 | FZD8 | -0.29 | 2.74E-02 |
| 3215 | FUT11 | -0.18 | 2.74E-02 |
| 3216 | SYT9 | 0.29 | 2.74E-02 |
| 3217 | PCDHA5 | -0.45 | 2.74E-02 |
| 3218 | VANGL1 | -0.31 | 2.74E-02 |
| 3219 | NEU2 | 0.17 | 2.74E-02 |
| 3220 | SMURF1 | -0.28 | 2.74E-02 |
| 3221 | LOC203107 | 0.16 | 2.74E-02 |
| 3222 | GRIN2A | 0.27 | 2.74E-02 |
| 3223 | LOC494558 | 0.25 | 2.74E-02 |
| 3224 | KCNJ13 | 0.29 | 2.75E-02 |
| 3225 | WNT4 | 0.24 | 2.75E-02 |
| 3226 | ADCYAP1R1 | 0.21 | 2.75E-02 |
| 3227 | GKN1 | 0.43 | 2.75E-02 |
| 3228 | ZNF251 | -0.47 | 2.75E-02 |
| 3229 | EXOC5 | 0.17 | 2.75E-02 |
| 3230 | SLC7A13 | 0.19 | 2.75E-02 |
| 3231 | CHST1 | -0.19 | 2.75E-02 |
| 3232 | CLIC2 | -0.18 | 2.75E-02 |
| 3233 | PPT2 | -0.14 | 2.76E-02 |
| 3234 | DPT | 0.31 | 2.76E-02 |
| 3235 | LOC400986 | -0.46 | 2.76E-02 |
| 3236 | ERGIC1 | 0.15 | 2.76E-02 |
| 3237 | C21orf66 | -0.44 | 2.76E-02 |
| 3238 | MLLT10 | 0.43 | 2.76E-02 |
| 3239 | ZNF620 | 0.32 | 2.76E-02 |
| 3240 | OR1E1 | 0.31 | 2.76E-02 |
| 3241 | RWDD1 | 0.23 | 2.76E-02 |
| 3242 | APBA2BP | -0.16 | 2.76E-02 |
| 3243 | VMO1 | -0.28 | 2.76E-02 |
| 3244 | OR6Q1 | 0.53 | 2.77E-02 |
| 3245 | FAM111A | -0.17 | 2.77E-02 |
| 3246 | XKRX | 0.18 | 2.77E-02 |
| 3247 | ABCC2 | 0.24 | 2.77E-02 |
| 3248 | SMG1 | -0.35 | 2.77E-02 |
| 3249 | ZD77D08 | 0.19 | 2.77E-02 |
| 3250 | KLF3 | 0.16 | 2.77E-02 |
| 3251 | GATAD2B | -0.17 | 2.77E-02 |
| 3252 | CRYBA4 | 0.29 | 2.77E-02 |
| 3253 | HSPA1L | -0.31 | 2.77E-02 |
| 3254 | CSF1R | -0.39 | 2.77E-02 |
| 3255 | PABPC5 | 0.27 | 2.77E-02 |
| 3256 | NHEDC1 | 0.19 | 2.77E-02 |
| 3257 | CD6 | 0.12 | 2.77E-02 |
| 3258 | RNF145 | -0.27 | 2.78E-02 |
| 3259 | AQP4 | 0.16 | 2.78E-02 |
| 3260 | SERPINA1 | -0.24 | 2.78E-02 |
| 3261 | C1orf64 | 0.41 | 2.78E-02 |
| 3262 | SULT2B1 | 0.17 | 2.78E-02 |
| 3263 | SNORD57 | -0.31 | 2.79E-02 |
| 3264 | PRKRIP1 | -0.13 | 2.79E-02 |
| 3265 | PPP1R12B | 0.25 | 2.79E-02 |
| 3266 | TNFRSF1A | -0.22 | 2.79E-02 |
| 3267 | B3GNT3 | -0.36 | 2.79E-02 |
| 3268 | OR10A3 | 0.26 | 2.79E-02 |
| 3269 | CRYBA2 | 0.18 | 2.79E-02 |
| 3270 | SNORD45A | -0.34 | 2.79E-02 |
| 3271 | SNF8 | -0.14 | 2.79E-02 |
| 3272 | ENDOD1 | -0.42 | 2.79E-02 |
| 3273 | RAD51L1 | 0.44 | 2.79E-02 |
| 3274 | ATP2C2 | 0.15 | 2.79E-02 |
| 3275 | COL6A3 | -0.39 | 2.80E-02 |
| 3276 | EFNB1 | -0.36 | 2.80E-02 |
| 3277 | PRELP | 0.15 | 2.80E-02 |
| 3278 | ITGAD | 0.34 | 2.80E-02 |
| 3279 | C14orf54 | 0.14 | 2.80E-02 |
| 3280 | GNRHR | 0.24 | 2.80E-02 |
| 3281 | KCTD8 | 0.26 | 2.80E-02 |
| 3282 | SDC4 | -0.26 | 2.80E-02 |
| 3283 | CD1E | -0.18 | 2.80E-02 |
| 3284 | SNORD44 | -0.35 | 2.80E-02 |
| 3285 | CPT1A | -0.29 | 2.80E-02 |
| 3286 | OR1A1 | 0.10 | 2.80E-02 |
| 3287 | TACR2 | 0.24 | 2.80E-02 |
| 3288 | NFKBIL2 | 0.31 | 2.80E-02 |
| 3289 | TBC1D3B | -0.22 | 2.80E-02 |
| 3290 | TRIMP1 | 0.29 | 2.80E-02 |
| 3291 | CREB3L2 | -0.24 | 2.80E-02 |
| 3292 | SGTA | 0.31 | 2.80E-02 |
| 3293 | BMP5 | 0.17 | 2.80E-02 |
| 3294 | C20orf54 | -0.38 | 2.80E-02 |
| 3295 | POU4F2 | 0.24 | 2.80E-02 |
| 3296 | ZNF75A | -0.34 | 2.80E-02 |
| 3297 | EXOC7 | -0.23 | 2.80E-02 |
| 3298 | IL27RA | -0.24 | 2.80E-02 |
| 3299 | UBAC2 | -0.24 | 2.80E-02 |
| 3300 | TMF1 | -0.25 | 2.80E-02 |
| 3301 | D21S2091E | 0.18 | 2.80E-02 |
| 3302 | TNIK | 0.29 | 2.80E-02 |
| 3303 | ACTL6B | 0.21 | 2.80E-02 |
| 3304 | LOC283624 | 0.21 | 2.80E-02 |
| 3305 | PTPN7 | -0.32 | 2.80E-02 |
| 3306 | ZNF780A | 0.19 | 2.80E-02 |
| 3307 | HIGD1A | 0.20 | 2.80E-02 |
| 3308 | KIAA1467 | -0.29 | 2.80E-02 |
| 3309 | PSCD1 | -0.19 | 2.81E-02 |
| 3310 | GEMIN6 | 0.18 | 2.81E-02 |
| 3311 | C9orf116 | -0.30 | 2.81E-02 |
| 3312 | RPS27 | 0.22 | 2.81E-02 |
| 3313 | CD8B | -0.18 | 2.81E-02 |
| 3314 | ZNF551 | -0.32 | 2.81E-02 |
| 3315 | FZD4 | -0.42 | 2.81E-02 |
| 3316 | USP51 | -0.27 | 2.81E-02 |
| 3317 | SCFD2 | -0.24 | 2.81E-02 |
| 3318 | LOC641365 | 0.14 | 2.81E-02 |
| 3319 | DDX11 | -0.21 | 2.82E-02 |
| 3320 | PPP1R8 | 0.26 | 2.82E-02 |
| 3321 | LILRA6 | 0.16 | 2.82E-02 |
| 3322 | C9orf37 | -0.18 | 2.82E-02 |
| 3323 | MGAT4C | 0.23 | 2.82E-02 |
| 3324 | POLR3K | -0.26 | 2.82E-02 |
| 3325 | CIITA | 0.19 | 2.82E-02 |
| 3326 | SIRT5 | 0.29 | 2.82E-02 |
| 3327 | NUDT22 | 0.33 | 2.82E-02 |
| 3328 | PLCD1 | 0.19 | 2.82E-02 |
| 3329 | RNF216L | -0.16 | 2.82E-02 |
| 3330 | GPR37 | -0.39 | 2.82E-02 |
| 3331 | PLAGL1 | 0.22 | 2.82E-02 |
| 3332 | ZNF264 | -0.33 | 2.82E-02 |
| 3333 | FAM81B | 0.33 | 2.83E-02 |
| 3334 | FLOT2 | -0.17 | 2.83E-02 |
| 3335 | HIF3A | 0.24 | 2.83E-02 |
| 3336 | KRTAP10-1 | -0.16 | 2.83E-02 |
| 3337 | KIAA0556 | -0.20 | 2.83E-02 |
| 3338 | PCID2 | -0.39 | 2.83E-02 |
| 3339 | RXFP2 | 0.25 | 2.83E-02 |
| 3340 | SMEK3P | 0.52 | 2.83E-02 |
| 3341 | CLCN3 | -0.28 | 2.83E-02 |
| 3342 | TNFAIP8 | 0.23 | 2.83E-02 |
| 3343 | CPLX1 | 0.14 | 2.83E-02 |
| 3344 | TEX9 | 0.28 | 2.84E-02 |
| 3345 | HSPA5 | -0.36 | 2.84E-02 |
| 3346 | SLC35A2 | 0.21 | 2.84E-02 |
| 3347 | ZSCAN23 | 0.17 | 2.84E-02 |
| 3348 | LOC285095 | 0.12 | 2.84E-02 |
| 3349 | LOC286109 | -0.27 | 2.84E-02 |
| 3350 | PCNX | -0.25 | 2.84E-02 |
| 3351 | MBD1 | -0.26 | 2.84E-02 |
| 3352 | LOC339760 | 0.37 | 2.84E-02 |
| 3353 | CYP2W1 | 0.33 | 2.84E-02 |
| 3354 | ANGPTL2 | -0.31 | 2.84E-02 |
| 3355 | NUMA1 | -0.22 | 2.84E-02 |
| 3356 | MAN2C1 | -0.19 | 2.84E-02 |
| 3357 | AIPL1 | 0.18 | 2.85E-02 |
| 3358 | CYP4F22 | 0.25 | 2.85E-02 |
| 3359 | ONECUT3 | 0.26 | 2.85E-02 |
| 3360 | MRGPRX3 | -0.23 | 2.85E-02 |
| 3361 | TNPO1 | -0.25 | 2.85E-02 |
| 3362 | PNPLA2 | -0.24 | 2.85E-02 |
| 3363 | OR2V2 | 0.20 | 2.85E-02 |
| 3364 | LRRC25 | 0.35 | 2.86E-02 |
| 3365 | CCDC116 | 0.27 | 2.86E-02 |
| 3366 | SMAP1 | -0.21 | 2.86E-02 |
| 3367 | SLC4A4 | -0.36 | 2.86E-02 |
| 3368 | MFSD9 | -0.38 | 2.86E-02 |
| 3369 | CD3G | 0.30 | 2.86E-02 |
| 3370 | ZNF334 | 0.50 | 2.86E-02 |
| 3371 | ALB | 0.36 | 2.86E-02 |
| 3372 | SGCD | -0.19 | 2.86E-02 |
| 3373 | HMBOX1 | -0.25 | 2.86E-02 |
| 3374 | MOBKL2A | 0.31 | 2.86E-02 |
| 3375 | GJA4 | -0.39 | 2.86E-02 |
| 3376 | ALK | 0.23 | 2.86E-02 |
| 3377 | SLC2A1 | -0.26 | 2.87E-02 |
| 3378 | KRTAP23-1 | 0.40 | 2.87E-02 |
| 3379 | TMEM146 | 0.27 | 2.87E-02 |
| 3380 | APOBEC2 | 0.27 | 2.87E-02 |
| 3381 | SQSTM1 | -0.19 | 2.87E-02 |
| 3382 | NSL1 | 0.48 | 2.87E-02 |
| 3383 | MFAP3L | 0.24 | 2.87E-02 |
| 3384 | SLC26A8 | 0.24 | 2.88E-02 |
| 3385 | RNF5 | -0.13 | 2.88E-02 |
| 3386 | HBXIP | 0.24 | 2.88E-02 |
| 3387 | MLLT3 | 0.32 | 2.88E-02 |
| 3388 | HIF1A | -0.21 | 2.88E-02 |
| 3389 | ZNF528 | -0.26 | 2.88E-02 |
| 3390 | EYA3 | 0.42 | 2.88E-02 |
| 3391 | LOC400662 | 0.29 | 2.88E-02 |
| 3392 | NFYC | -0.26 | 2.88E-02 |
| 3393 | FGD5 | -0.28 | 2.88E-02 |
| 3394 | LOC401351 | 0.16 | 2.89E-02 |
| 3395 | LOC728353 | 0.28 | 2.89E-02 |
| 3396 | LOC440366 | 0.20 | 2.89E-02 |
| 3397 | NDE1 | -0.27 | 2.89E-02 |
| 3398 | LOC151234 | 0.14 | 2.89E-02 |
| 3399 | SP100 | 0.21 | 2.89E-02 |
| 3400 | hCG_1651889 | 0.14 | 2.89E-02 |
| 3401 | OSBPL3 | -0.33 | 2.90E-02 |
| 3402 | TGIF2LY | 0.09 | 2.90E-02 |
| 3403 | FLJ23834 | 0.23 | 2.90E-02 |
| 3404 | BCAP31 | -0.19 | 2.90E-02 |
| 3405 | ICA1L | 0.29 | 2.90E-02 |
| 3406 | GPR156 | 0.16 | 2.90E-02 |
| 3407 | ITGB1 | -0.32 | 2.90E-02 |
| 3408 | ZNF665 | -0.26 | 2.90E-02 |
| 3409 | FGF14 | 0.12 | 2.90E-02 |
| 3410 | LCAT | 0.38 | 2.90E-02 |
| 3411 | FSTL4 | 0.23 | 2.90E-02 |
| 3412 | RNPS1 | -0.19 | 2.90E-02 |
| 3413 | CLN8 | -0.17 | 2.91E-02 |
| 3414 | MID2 | 0.16 | 2.91E-02 |
| 3415 | DLX3 | 0.17 | 2.91E-02 |
| 3416 | TMEM168 | -0.35 | 2.91E-02 |
| 3417 | PLXNC1 | 0.45 | 2.91E-02 |
| 3418 | VPS13C | -0.23 | 2.91E-02 |
| 3419 | ART3 | 0.17 | 2.91E-02 |
| 3420 | FARP1 | -0.11 | 2.91E-02 |
| 3421 | LRIG1 | -0.36 | 2.91E-02 |
| 3422 | LOC390595 | 0.23 | 2.91E-02 |
| 3423 | ERLIN1 | -0.18 | 2.91E-02 |
| 3424 | C7orf49 | -0.15 | 2.91E-02 |
| 3425 | MKS1 | -0.20 | 2.91E-02 |
| 3426 | AGPAT7 | -0.15 | 2.91E-02 |
| 3427 | FAM47C | -0.10 | 2.91E-02 |
| 3428 | SLC29A1 | -0.29 | 2.91E-02 |
| 3429 | FLJ12825 | 0.13 | 2.91E-02 |
| 3430 | HNF4A | -0.34 | 2.91E-02 |
| 3431 | ZNF555 | -0.26 | 2.91E-02 |
| 3432 | LOC729378 | 0.10 | 2.91E-02 |
| 3433 | DSE | -0.25 | 2.91E-02 |
| 3434 | ARFGAP3 | -0.32 | 2.91E-02 |
| 3435 | SPCS2 | -0.21 | 2.91E-02 |
| 3436 | FCHO2 | 0.23 | 2.91E-02 |
| 3437 | LAMP1 | -0.26 | 2.91E-02 |
| 3438 | FAM47A | 0.33 | 2.91E-02 |
| 3439 | CNOT7 | -0.32 | 2.91E-02 |
| 3440 | OGG1 | 0.14 | 2.91E-02 |
| 3441 | NPM2 | -0.11 | 2.91E-02 |
| 3442 | UST | 0.27 | 2.91E-02 |
| 3443 | CDC14B | -0.21 | 2.92E-02 |
| 3444 | DISP1 | 0.28 | 2.92E-02 |
| 3445 | LONP1 | -0.18 | 2.92E-02 |
| 3446 | FILIP1L | 0.18 | 2.92E-02 |
| 3447 | CTBS | 0.30 | 2.92E-02 |
| 3448 | OPA3 | 0.20 | 2.93E-02 |
| 3449 | ALS2CR13 | 0.44 | 2.93E-02 |
| 3450 | HOXC5 | -0.25 | 2.93E-02 |
| 3451 | SLC6A2 | 0.32 | 2.93E-02 |
| 3452 | PDLIM5 | 0.49 | 2.93E-02 |
| 3453 | C15orf48 | 0.41 | 2.94E-02 |
| 3454 | UQCR | 0.14 | 2.94E-02 |
| 3455 | FABP3 | 0.25 | 2.94E-02 |
| 3456 | C14orf112 | 0.19 | 2.94E-02 |
| 3457 | ZC3H18 | -0.19 | 2.94E-02 |
| 3458 | CDK6 | -0.44 | 2.94E-02 |
| 3459 | LOC338328 | 0.26 | 2.94E-02 |
| 3460 | PELO | -0.20 | 2.94E-02 |
| 3461 | ITGA4 | -0.24 | 2.94E-02 |
| 3462 | POTE15 | 0.21 | 2.95E-02 |
| 3463 | CD248 | 0.16 | 2.95E-02 |
| 3464 | GH1 | 0.13 | 2.95E-02 |
| 3465 | PDE3A | 0.21 | 2.95E-02 |
| 3466 | ATP1B1 | -0.31 | 2.95E-02 |
| 3467 | INCA | 0.28 | 2.95E-02 |
| 3468 | TFCP2L1 | -0.45 | 2.95E-02 |
| 3469 | MUC5B | -0.17 | 2.96E-02 |
| 3470 | WDR82 | -0.16 | 2.96E-02 |
| 3471 | ADARB1 | -0.12 | 2.96E-02 |
| 3472 | PURB | -0.25 | 2.96E-02 |
| 3473 | HIST1H2AB | 0.18 | 2.96E-02 |
| 3474 | CCDC107 | -0.21 | 2.96E-02 |
| 3475 | HCG4P6 | 0.27 | 2.97E-02 |
| 3476 | KIAA0226 | -0.22 | 2.97E-02 |
| 3477 | DNAJB13 | 0.23 | 2.97E-02 |
| 3478 | CXXC4 | 0.16 | 2.97E-02 |
| 3479 | ALG8 | -0.23 | 2.97E-02 |
| 3480 | PRAC | 0.23 | 2.97E-02 |
| 3481 | AZGP1 | 0.14 | 2.97E-02 |
| 3482 | TOP2A | -0.33 | 2.98E-02 |
| 3483 | LRCH1 | 0.22 | 2.98E-02 |
| 3484 | SPRR1B | 0.32 | 2.98E-02 |
| 3485 | ACSM2A | 0.12 | 2.98E-02 |
| 3486 | DKK3 | -0.27 | 2.98E-02 |
| 3487 | KITLG | 0.14 | 2.98E-02 |
| 3488 | SLC1A4 | -0.25 | 2.98E-02 |
| 3489 | MAP7D1 | -0.19 | 2.98E-02 |
| 3490 | BLOC1S2 | 0.20 | 2.98E-02 |
| 3491 | PURA | -0.22 | 2.98E-02 |
| 3492 | TRIM24 | -0.22 | 2.98E-02 |
| 3493 | CCDC131 | -0.25 | 2.98E-02 |
| 3494 | RP11-167P23.2 | 0.13 | 2.98E-02 |
| 3495 | FLJ41993 | -0.11 | 2.98E-02 |
| 3496 | FLJ25778 | 0.39 | 2.98E-02 |
| 3497 | OLFM2 | 0.43 | 2.98E-02 |
| 3498 | RPS5 | 0.18 | 2.98E-02 |
| 3499 | MFI2 | -0.37 | 2.98E-02 |
| 3500 | HIGD1A | 0.22 | 2.99E-02 |
| 3501 | CENPO | -0.14 | 2.99E-02 |
| 3502 | C4orf36 | 0.27 | 2.99E-02 |
| 3503 | hCG_2045830 | -0.18 | 2.99E-02 |
| 3504 | C6orf184 | 0.30 | 2.99E-02 |
| 3505 | WHSC1 | -0.19 | 2.99E-02 |
| 3506 | SH3GL3 | -0.28 | 2.99E-02 |
| 3507 | ISL2 | -0.15 | 3.00E-02 |
| 3508 | ZFP1 | -0.21 | 3.00E-02 |
| 3509 | LOC151475 | 0.29 | 3.00E-02 |
| 3510 | CYP26C1 | 0.21 | 3.00E-02 |
| 3511 | DTNA | 0.41 | 3.00E-02 |
| 3512 | HS3ST4 | 0.21 | 3.00E-02 |
| 3513 | NUMBL | 0.31 | 3.00E-02 |
| 3514 | ADNP | 0.25 | 3.01E-02 |
| 3515 | LOC727710 | 0.24 | 3.01E-02 |
| 3516 | ANKMY2 | 0.18 | 3.01E-02 |
| 3517 | LOC731157 | 0.24 | 3.01E-02 |
| 3518 | ST7L | 0.19 | 3.01E-02 |
| 3519 | TMCO1 | -0.19 | 3.01E-02 |
| 3520 | C10orf44 | 0.19 | 3.01E-02 |
| 3521 | LOC610563 | 0.29 | 3.01E-02 |
| 3522 | MAPK8IP2 | 0.37 | 3.01E-02 |
| 3523 | KIAA0182 | -0.27 | 3.01E-02 |
| 3524 | CFHR4 | 0.24 | 3.01E-02 |
| 3525 | RPL14 | 0.23 | 3.01E-02 |
| 3526 | ADAM8 | -0.31 | 3.02E-02 |
| 3527 | C14orf49 | -0.13 | 3.02E-02 |
| 3528 | FLJ37464 | 0.32 | 3.02E-02 |
| 3529 | LOC729177 | 0.28 | 3.02E-02 |
| 3530 | LOC283357 | -0.19 | 3.02E-02 |
| 3531 | WDR33 | -0.34 | 3.02E-02 |
| 3532 | ZNF117 | 0.24 | 3.02E-02 |
| 3533 | IGHMBP2 | -0.15 | 3.02E-02 |
| 3534 | LOC642350 | 0.20 | 3.02E-02 |
| 3535 | C21orf45 | -0.24 | 3.03E-02 |
| 3536 | FAM87A | 0.21 | 3.03E-02 |
| 3537 | FLJ30428 | 0.30 | 3.03E-02 |
| 3538 | CLCNKB | 0.20 | 3.03E-02 |
| 3539 | PPM1L | -0.21 | 3.03E-02 |
| 3540 | LOC284263 | 0.13 | 3.03E-02 |
| 3541 | KCNAB2 | 0.13 | 3.04E-02 |
| 3542 | ADAT2 | -0.24 | 3.04E-02 |
| 3543 | C6orf47 | -0.25 | 3.04E-02 |
| 3544 | MARVELD2 | -0.27 | 3.04E-02 |
| 3545 | SOX11 | -0.31 | 3.04E-02 |
| 3546 | C19orf40 | 0.40 | 3.04E-02 |
| 3547 | ZNF800 | 0.16 | 3.04E-02 |
| 3548 | ALG1 | -0.33 | 3.04E-02 |
| 3549 | LOC389174 | 0.21 | 3.04E-02 |
| 3550 | C11orf3 | 0.30 | 3.04E-02 |
| 3551 | WDR1 | -0.16 | 3.04E-02 |
| 3552 | FLJ39531 | 0.16 | 3.04E-02 |
| 3553 | LEPROTL1 | -0.31 | 3.04E-02 |
| 3554 | SLC25A5 | 0.27 | 3.05E-02 |
| 3555 | C7orf47 | -0.20 | 3.05E-02 |
| 3556 | OPRD1 | 0.16 | 3.05E-02 |
| 3557 | BFAR | -0.14 | 3.05E-02 |
| 3558 | CBLN3 | 0.11 | 3.05E-02 |
| 3559 | FLJ44874 | 0.09 | 3.05E-02 |
| 3560 | KIAA1656 | 0.15 | 3.05E-02 |
| 3561 | GPR42 | 0.39 | 3.05E-02 |
| 3562 | RPL23AP7 | 0.18 | 3.05E-02 |
| 3563 | NPM1 | 0.19 | 3.06E-02 |
| 3564 | PTGDR | -0.15 | 3.06E-02 |
| 3565 | JAK1 | -0.42 | 3.06E-02 |
| 3566 | LOC479559 | 0.28 | 3.06E-02 |
| 3567 | PAFAH1B1 | -0.27 | 3.06E-02 |
| 3568 | HNRNPA1 | 0.27 | 3.06E-02 |
| 3569 | LSM14A | 0.13 | 3.06E-02 |
| 3570 | MTX1 | 0.23 | 3.06E-02 |
| 3571 | MYH9 | -0.24 | 3.06E-02 |
| 3572 | HHLA3 | -0.14 | 3.06E-02 |
| 3573 | VKORC1L1 | -0.29 | 3.07E-02 |
| 3574 | ECE2 | 0.26 | 3.07E-02 |
| 3575 | PLEKHA2 | -0.14 | 3.07E-02 |
| 3576 | UMPS | -0.15 | 3.07E-02 |
| 3577 | OR10H5 | 0.17 | 3.07E-02 |
| 3578 | PIAS1 | -0.39 | 3.07E-02 |
| 3579 | SLC46A1 | -0.10 | 3.07E-02 |
| 3580 | G6PC | 0.32 | 3.07E-02 |
| 3581 | CALCR | -0.18 | 3.07E-02 |
| 3582 | C3orf24 | 0.29 | 3.07E-02 |
| 3583 | NSUN5C | -0.18 | 3.07E-02 |
| 3584 | KLHL32 | 0.20 | 3.07E-02 |
| 3585 | GRIK3 | 0.38 | 3.08E-02 |
| 3586 | CTF1 | 0.22 | 3.08E-02 |
| 3587 | GRINL1A | 0.16 | 3.08E-02 |
| 3588 | LOC730961 | 0.16 | 3.08E-02 |
| 3589 | SLAMF9 | -0.27 | 3.08E-02 |
| 3590 | TMEM39B | -0.23 | 3.08E-02 |
| 3591 | DNAJB7 | 0.55 | 3.08E-02 |
| 3592 | TAOK2 | 0.25 | 3.08E-02 |
| 3593 | RPLP0 | 0.32 | 3.09E-02 |
| 3594 | MUC12 | 0.20 | 3.09E-02 |
| 3595 | CD47 | -0.36 | 3.09E-02 |
| 3596 | ALOXE3 | 0.19 | 3.09E-02 |
| 3597 | KCNA1 | 0.38 | 3.09E-02 |
| 3598 | PBX1 | 0.28 | 3.10E-02 |
| 3599 | ACSM3 | -0.27 | 3.10E-02 |
| 3600 | G3BP1 | -0.23 | 3.10E-02 |
| 3601 | SYCN | 0.25 | 3.10E-02 |
| 3602 | OR51E1 | 0.22 | 3.10E-02 |
| 3603 | PKLR | 0.19 | 3.10E-02 |
| 3604 | LOC91431 | 0.21 | 3.10E-02 |
| 3605 | RAG1 | -0.16 | 3.10E-02 |
| 3606 | SMTNL2 | 0.12 | 3.11E-02 |
| 3607 | CR1 | 0.13 | 3.11E-02 |
| 3608 | IRX2 | -0.25 | 3.11E-02 |
| 3609 | CNTN3 | 0.22 | 3.11E-02 |
| 3610 | C20orf59 | -0.27 | 3.11E-02 |
| 3611 | FAM83F | 0.25 | 3.11E-02 |
| 3612 | DDX43 | -0.56 | 3.11E-02 |
| 3613 | PIGK | -0.27 | 3.11E-02 |
| 3614 | KIR2DS2 | 0.19 | 3.12E-02 |
| 3615 | SERPINH1 | -0.24 | 3.12E-02 |
| 3616 | KIAA0506 | 0.51 | 3.12E-02 |
| 3617 | ST6GAL1 | -0.27 | 3.12E-02 |
| 3618 | PAX9 | 0.29 | 3.12E-02 |
| 3619 | LOC731789 | 0.39 | 3.12E-02 |
| 3620 | APOC3 | -0.21 | 3.13E-02 |
| 3621 | EEF1B2 | 0.20 | 3.13E-02 |
| 3622 | DPAGT1 | -0.28 | 3.13E-02 |
| 3623 | GCAT | -0.16 | 3.13E-02 |
| 3624 | CUEDC1 | -0.21 | 3.13E-02 |
| 3625 | KIR2DL1 | 0.20 | 3.13E-02 |
| 3626 | RGS3 | -0.22 | 3.13E-02 |
| 3627 | AIP | 0.39 | 3.13E-02 |
| 3628 | POU1F1 | 0.30 | 3.14E-02 |
| 3629 | NSUN5B | -0.17 | 3.14E-02 |
| 3630 | GDPD4 | 0.15 | 3.14E-02 |
| 3631 | DHX38 | -0.16 | 3.14E-02 |
| 3632 | FBN2 | -0.40 | 3.14E-02 |
| 3633 | ARAF | -0.10 | 3.14E-02 |
| 3634 | MTHFR | 0.22 | 3.15E-02 |
| 3635 | STRA6 | -0.22 | 3.15E-02 |
| 3636 | TXNDC15 | -0.30 | 3.15E-02 |
| 3637 | C6orf168 | 0.13 | 3.15E-02 |
| 3638 | TAP1 | -0.26 | 3.15E-02 |
| 3639 | CEACAM20 | 0.17 | 3.15E-02 |
| 3640 | C10orf27 | 0.23 | 3.16E-02 |
| 3641 | RNF144B | -0.33 | 3.16E-02 |
| 3642 | KIAA0082 | -0.26 | 3.16E-02 |
| 3643 | LOC388458 | 0.18 | 3.16E-02 |
| 3644 | SFRS14 | -0.24 | 3.16E-02 |
| 3645 | TMEM50A | -0.29 | 3.16E-02 |
| 3646 | ELOVL1 | -0.22 | 3.16E-02 |
| 3647 | TMEM180 | -0.19 | 3.17E-02 |
| 3648 | FLJ25439 | 0.12 | 3.17E-02 |
| 3649 | VSX1 | 0.14 | 3.17E-02 |
| 3650 | PELI2 | 0.22 | 3.17E-02 |
| 3651 | C1orf167 | 0.24 | 3.17E-02 |
| 3652 | RIC8A | -0.18 | 3.17E-02 |
| 3653 | LOC152118 | 0.15 | 3.18E-02 |
| 3654 | TGOLN2 | -0.15 | 3.18E-02 |
| 3655 | ADAMTSL4 | -0.13 | 3.18E-02 |
| 3656 | NUP188 | -0.19 | 3.18E-02 |
| 3657 | CFB | -0.30 | 3.18E-02 |
| 3658 | RAD9A | 0.33 | 3.18E-02 |
| 3659 | CHRNG | -0.13 | 3.18E-02 |
| 3660 | SLC26A8 | 0.22 | 3.18E-02 |
| 3661 | CNTNAP4 | 0.20 | 3.18E-02 |
| 3662 | ZNF793 | 0.19 | 3.18E-02 |
| 3663 | EGR4 | -0.22 | 3.18E-02 |
| 3664 | RPL7A | 0.25 | 3.18E-02 |
| 3665 | LOC222699 | -0.10 | 3.18E-02 |
| 3666 | SPATA20 | -0.16 | 3.18E-02 |
| 3667 | FAM129A | -0.35 | 3.18E-02 |
| 3668 | MGC33407 | -0.14 | 3.19E-02 |
| 3669 | LOC729436 | -0.20 | 3.19E-02 |
| 3670 | CCDC144A | 0.20 | 3.19E-02 |
| 3671 | CTNND2 | 0.35 | 3.19E-02 |
| 3672 | GPR182 | 0.33 | 3.19E-02 |
| 3673 | AMY2B | -0.34 | 3.19E-02 |
| 3674 | PPIF | -0.22 | 3.19E-02 |
| 3675 | WDR62 | -0.24 | 3.19E-02 |
| 3676 | IFNAR2 | -0.31 | 3.19E-02 |
| 3677 | GLRA2 | 0.20 | 3.19E-02 |
| 3678 | TFPI | -0.19 | 3.19E-02 |
| 3679 | STARD4 | 0.45 | 3.19E-02 |
| 3680 | STX3 | -0.21 | 3.19E-02 |
| 3681 | C14orf106 | -0.29 | 3.19E-02 |
| 3682 | SLC11A2 | -0.22 | 3.19E-02 |
| 3683 | MARK3 | -0.22 | 3.20E-02 |
| 3684 | KRT6C | 0.29 | 3.20E-02 |
| 3685 | RPL39 | 0.19 | 3.20E-02 |
| 3686 | LOC644135 | 0.18 | 3.20E-02 |
| 3687 | RBMS3 | 0.34 | 3.20E-02 |
| 3688 | FLJ39080 | 0.29 | 3.20E-02 |
| 3689 | CSHL1 | 0.22 | 3.20E-02 |
| 3690 | TRPV4 | -0.23 | 3.21E-02 |
| 3691 | MORG1 | -0.15 | 3.21E-02 |
| 3692 | PLEKHG5 | -0.27 | 3.21E-02 |
| 3693 | SUMO2 | 0.23 | 3.21E-02 |
| 3694 | KLHL18 | -0.20 | 3.22E-02 |
| 3695 | LOC283079 | 0.18 | 3.22E-02 |
| 3696 | PCDH12 | -0.26 | 3.22E-02 |
| 3697 | CCDC144B | 0.54 | 3.22E-02 |
| 3698 | CNPY3 | -0.17 | 3.22E-02 |
| 3699 | KIAA1553 | -0.19 | 3.22E-02 |
| 3700 | ATP6V1B2 | -0.25 | 3.22E-02 |
| 3701 | ATF7IP2 | -0.24 | 3.22E-02 |
| 3702 | SLC17A3 | 0.39 | 3.23E-02 |
| 3703 | GPR15 | 0.22 | 3.23E-02 |
| 3704 | GLUD2 | -0.20 | 3.23E-02 |
| 3705 | PDE8B | -0.24 | 3.23E-02 |
| 3706 | SORT1 | -0.21 | 3.24E-02 |
| 3707 | SUMF1 | -0.29 | 3.24E-02 |
| 3708 | TULP2 | 0.19 | 3.24E-02 |
| 3709 | SLC25A21 | 0.32 | 3.24E-02 |
| 3710 | RAD51L3 | 0.41 | 3.24E-02 |
| 3711 | ATP2A1 | -0.10 | 3.24E-02 |
| 3712 | TBC1D22B | -0.15 | 3.24E-02 |
| 3713 | CCDC36 | 0.22 | 3.24E-02 |
| 3714 | POLQ | 0.22 | 3.24E-02 |
| 3715 | MLLT4 | -0.34 | 3.24E-02 |
| 3716 | PTPRK | -0.31 | 3.24E-02 |
| 3717 | P2RY6 | -0.14 | 3.25E-02 |
| 3718 | SH2D5 | 0.28 | 3.25E-02 |
| 3719 | PPP1R16B | -0.21 | 3.25E-02 |
| 3720 | HSPC105 | 0.17 | 3.25E-02 |
| 3721 | TRMT5 | 0.17 | 3.25E-02 |
| 3722 | MXRA5 | 0.16 | 3.25E-02 |
| 3723 | DLC1 | 0.23 | 3.25E-02 |
| 3724 | TYMS | 0.21 | 3.25E-02 |
| 3725 | ZDHHC11 | -0.26 | 3.25E-02 |
| 3726 | MEF2D | -0.23 | 3.25E-02 |
| 3727 | TGM2 | -0.17 | 3.25E-02 |
| 3728 | SCN3A | 0.29 | 3.25E-02 |
| 3729 | COL27A1 | 0.34 | 3.25E-02 |
| 3730 | TncRNA | -0.20 | 3.25E-02 |
| 3731 | FSTL3 | -0.11 | 3.25E-02 |
| 3732 | ANLN | -0.29 | 3.25E-02 |
| 3733 | C16orf62 | 0.35 | 3.25E-02 |
| 3734 | TBL1X | -0.21 | 3.25E-02 |
| 3735 | PCDHAC1 | 0.21 | 3.25E-02 |
| 3736 | MUSTN1 | 0.10 | 3.25E-02 |
| 3737 | CLDN16 | 0.43 | 3.26E-02 |
| 3738 | FERD3L | 0.36 | 3.26E-02 |
| 3739 | LOC400960 | 0.24 | 3.26E-02 |
| 3740 | KRT72 | 0.17 | 3.26E-02 |
| 3741 | WIZ | 0.17 | 3.26E-02 |
| 3742 | LRP1 | -0.27 | 3.26E-02 |
| 3743 | LOC729026 | -0.29 | 3.26E-02 |
| 3744 | HTR5B | 0.20 | 3.27E-02 |
| 3745 | OR6K2 | 0.26 | 3.27E-02 |
| 3746 | ANP32D | -0.15 | 3.27E-02 |
| 3747 | MYCBPAP | 0.14 | 3.27E-02 |
| 3748 | LIG4 | -0.37 | 3.27E-02 |
| 3749 | LOC286083 | 0.14 | 3.27E-02 |
| 3750 | C22orf30 | -0.16 | 3.27E-02 |
| 3751 | TNPO3 | -0.27 | 3.27E-02 |
| 3752 | CACNA1E | 0.41 | 3.27E-02 |
| 3753 | DGCR6 | 0.10 | 3.27E-02 |
| 3754 | KLRG2 | 0.29 | 3.27E-02 |
| 3755 | ZNF530 | -0.20 | 3.27E-02 |
| 3756 | OR9A4 | 0.26 | 3.27E-02 |
| 3757 | GNRH2 | -0.15 | 3.27E-02 |
| 3758 | KRT38 | 0.18 | 3.27E-02 |
| 3759 | HAS2 | 0.20 | 3.28E-02 |
| 3760 | C10orf26 | 0.31 | 3.28E-02 |
| 3761 | LOC91149 | 0.33 | 3.28E-02 |
| 3762 | COL18A1 | -0.30 | 3.28E-02 |
| 3763 | ADCY4 | -0.27 | 3.29E-02 |
| 3764 | HMGB1 | 0.29 | 3.29E-02 |
| 3765 | CHRNB2 | 0.21 | 3.29E-02 |
| 3766 | SNTA1 | 0.36 | 3.29E-02 |
| 3767 | HIPK2 | -0.29 | 3.29E-02 |
| 3768 | PRKCSH | -0.19 | 3.29E-02 |
| 3769 | BCL9 | -0.23 | 3.29E-02 |
| 3770 | RABAC1 | -0.21 | 3.29E-02 |
| 3771 | KIAA0427 | 0.42 | 3.29E-02 |
| 3772 | NCOR1 | -0.29 | 3.30E-02 |
| 3773 | ERG | 0.24 | 3.30E-02 |
| 3774 | LOC646708 | 0.25 | 3.30E-02 |
| 3775 | C1orf51 | -0.37 | 3.30E-02 |
| 3776 | ABLIM1 | 0.22 | 3.30E-02 |
| 3777 | OR2T12 | 0.24 | 3.30E-02 |
| 3778 | NDUFA8 | 0.16 | 3.30E-02 |
| 3779 | SH3GL1 | -0.15 | 3.30E-02 |
| 3780 | FBXL22 | -0.12 | 3.31E-02 |
| 3781 | LOC147791 | 0.26 | 3.31E-02 |
| 3782 | TMEM80 | -0.16 | 3.31E-02 |
| 3783 | CLCN5 | -0.36 | 3.31E-02 |
| 3784 | PRDM16 | -0.24 | 3.31E-02 |
| 3785 | LOC153577 | -0.34 | 3.31E-02 |
| 3786 | ZNF705A | -0.12 | 3.31E-02 |
| 3787 | CCDC11 | 0.16 | 3.32E-02 |
| 3788 | LOC284244 | 0.14 | 3.32E-02 |
| 3789 | ITSN1 | 0.20 | 3.32E-02 |
| 3790 | TAGAP | -0.16 | 3.32E-02 |
| 3791 | DOLPP1 | -0.32 | 3.32E-02 |
| 3792 | DR1 | 0.14 | 3.32E-02 |
| 3793 | THSD1P | 0.16 | 3.32E-02 |
| 3794 | SLC27A1 | -0.27 | 3.32E-02 |
| 3795 | ELF2 | 0.39 | 3.32E-02 |
| 3796 | SHANK3 | 0.15 | 3.33E-02 |
| 3797 | MFSD7 | 0.22 | 3.33E-02 |
| 3798 | BAI1 | 0.20 | 3.33E-02 |
| 3799 | RPS3A | 0.32 | 3.33E-02 |
| 3800 | C4B | -0.34 | 3.33E-02 |
| 3801 | TSPAN7 | -0.22 | 3.33E-02 |
| 3802 | ATP6V0C | -0.15 | 3.33E-02 |
| 3803 | CRY2 | -0.24 | 3.33E-02 |
| 3804 | PPAP2B | -0.29 | 3.34E-02 |
| 3805 | POLS | -0.21 | 3.34E-02 |
| 3806 | ATG16L2 | 0.26 | 3.34E-02 |
| 3807 | PRR17 | 0.29 | 3.34E-02 |
| 3808 | DCBLD2 | -0.27 | 3.34E-02 |
| 3809 | CUL1 | -0.24 | 3.34E-02 |
| 3810 | NXNL1 | 0.19 | 3.34E-02 |
| 3811 | SNORD36C | -0.31 | 3.34E-02 |
| 3812 | FLJ20309 | 0.14 | 3.34E-02 |
| 3813 | OSTM1 | -0.22 | 3.34E-02 |
| 3814 | PGF | -0.23 | 3.35E-02 |
| 3815 | LOC283901 | 0.21 | 3.35E-02 |
| 3816 | PPP2R5A | -0.16 | 3.35E-02 |
| 3817 | GMPPA | -0.14 | 3.35E-02 |
| 3818 | CARD14 | -0.21 | 3.35E-02 |
| 3819 | EDEM1 | -0.28 | 3.35E-02 |
| 3820 | RSC1A1 | -0.32 | 3.35E-02 |
| 3821 | LOC196541 | 0.18 | 3.35E-02 |
| 3822 | TMEM17 | -0.33 | 3.35E-02 |
| 3823 | SLC20A1 | -0.21 | 3.35E-02 |
| 3824 | AGPAT5 | -0.23 | 3.35E-02 |
| 3825 | KIAA0500 | -0.43 | 3.35E-02 |
| 3826 | EIF4E2 | 0.12 | 3.35E-02 |
| 3827 | TRA2A | 0.53 | 3.35E-02 |
| 3828 | TMEM38B | 0.51 | 3.35E-02 |
| 3829 | XRCC1 | -0.16 | 3.35E-02 |
| 3830 | PTH | 0.20 | 3.35E-02 |
| 3831 | TRIM60 | 0.20 | 3.35E-02 |
| 3832 | CSNK1G1 | 0.20 | 3.36E-02 |
| 3833 | TALDO1 | -0.14 | 3.36E-02 |
| 3834 | CNTD1 | 0.12 | 3.36E-02 |
| 3835 | NICN1 | -0.20 | 3.36E-02 |
| 3836 | COASY | -0.23 | 3.36E-02 |
| 3837 | SLC2A1 | -0.25 | 3.36E-02 |
| 3838 | FUT8 | -0.35 | 3.36E-02 |
| 3839 | THSD7A | -0.25 | 3.36E-02 |
| 3840 | LOC221814 | 0.32 | 3.36E-02 |
| 3841 | ARHGEF2 | -0.12 | 3.36E-02 |
| 3842 | TMUB2 | -0.28 | 3.37E-02 |
| 3843 | MYO1A | 0.20 | 3.37E-02 |
| 3844 | C12orf24 | 0.16 | 3.37E-02 |
| 3845 | P4HA1 | -0.33 | 3.37E-02 |
| 3846 | LZTS1 | -0.24 | 3.37E-02 |
| 3847 | SERPINA9///SERPINA11 | 0.20 | 3.37E-02 |
| 3848 | LOC554235 | 0.15 | 3.37E-02 |
| 3849 | ARHGEF11 | 0.32 | 3.37E-02 |
| 3850 | LRP8 | -0.20 | 3.37E-02 |
| 3851 | TAP1 | -0.33 | 3.37E-02 |
| 3852 | HNRPA1L-2///HNRNPA1 | 0.25 | 3.37E-02 |
| 3853 | AGER | 0.30 | 3.37E-02 |
| 3854 | ZGPAT | -0.21 | 3.38E-02 |
| 3855 | ABTB1 | -0.21 | 3.38E-02 |
| 3856 | ELOVL3 | 0.17 | 3.38E-02 |
| 3857 | FLJ37464 | -0.17 | 3.38E-02 |
| 3858 | DTX4 | -0.28 | 3.38E-02 |
| 3859 | FGFBP3 | 0.21 | 3.38E-02 |
| 3860 | ABCB9 | 0.16 | 3.38E-02 |
| 3861 | EPRS | -0.23 | 3.39E-02 |
| 3862 | RAC1 | -0.19 | 3.39E-02 |
| 3863 | PLIN | 0.27 | 3.39E-02 |
| 3864 | LASS6 | -0.26 | 3.39E-02 |
| 3865 | OIT3 | 0.26 | 3.40E-02 |
| 3866 | PLCG1 | -0.18 | 3.40E-02 |
| 3867 | XYLT1 | -0.19 | 3.40E-02 |
| 3868 | SLC39A7 | -0.18 | 3.40E-02 |
| 3869 | IGFBP5 | -0.23 | 3.40E-02 |
| 3870 | APOOL | 0.21 | 3.40E-02 |
| 3871 | UGT1A6 | -0.32 | 3.40E-02 |
| 3872 | ISYNA1 | -0.21 | 3.40E-02 |
| 3873 | AP4S1 | -0.13 | 3.40E-02 |
| 3874 | RPS21 | 0.20 | 3.40E-02 |
| 3875 | FXYD2 | 0.24 | 3.41E-02 |
| 3876 | SMPD1 | -0.14 | 3.41E-02 |
| 3877 | PRRG4 | -0.16 | 3.41E-02 |
| 3878 | LOC440737 | 0.20 | 3.41E-02 |
| 3879 | HSP90AA5P | -0.37 | 3.41E-02 |
| 3880 | HSD17B7 | -0.23 | 3.41E-02 |
| 3881 | AP3B1 | 0.18 | 3.41E-02 |
| 3882 | B4GALNT3 | 0.19 | 3.41E-02 |
| 3883 | FLJ30375 | 0.36 | 3.41E-02 |
| 3884 | POLR3E | -0.26 | 3.41E-02 |
| 3885 | PTK6 | 0.18 | 3.41E-02 |
| 3886 | SPN | 0.18 | 3.41E-02 |
| 3887 | ZNF592 | 0.16 | 3.41E-02 |
| 3888 | FOXC2 | 0.19 | 3.41E-02 |
| 3889 | RABGGTA | -0.15 | 3.41E-02 |
| 3890 | TAF12 | 0.39 | 3.41E-02 |
| 3891 | NRK | 0.34 | 3.41E-02 |
| 3892 | ZNF746 | -0.16 | 3.41E-02 |
| 3893 | SNX27 | -0.24 | 3.41E-02 |
| 3894 | FGF22 | 0.17 | 3.41E-02 |
| 3895 | SLC6A12 | 0.24 | 3.41E-02 |
| 3896 | FAM38B | 0.26 | 3.41E-02 |
| 3897 | A1BG | -0.13 | 3.41E-02 |
| 3898 | DPP6 | 0.37 | 3.42E-02 |
| 3899 | EFCAB4A | -0.18 | 3.42E-02 |
| 3900 | ACRBP | 0.15 | 3.42E-02 |
| 3901 | HDAC4 | -0.18 | 3.42E-02 |
| 3902 | BRSK1 | 0.26 | 3.42E-02 |
| 3903 | POFUT2 | 0.18 | 3.42E-02 |
| 3904 | COL10A1 | -0.58 | 3.42E-02 |
| 3905 | BRCA2 | -0.21 | 3.42E-02 |
| 3906 | LOC285084 | 0.14 | 3.42E-02 |
| 3907 | DNASE1L2 | 0.15 | 3.42E-02 |
| 3908 | KLRC1 | 0.42 | 3.42E-02 |
| 3909 | KIAA1549 | -0.32 | 3.43E-02 |
| 3910 | ATPAF2 | -0.22 | 3.43E-02 |
| 3911 | GLIS2 | 0.29 | 3.43E-02 |
| 3912 | NT5C3 | -0.27 | 3.43E-02 |
| 3913 | C4A | -0.14 | 3.43E-02 |
| 3914 | TIMM17A | -0.28 | 3.43E-02 |
| 3915 | SAFB2 | -0.19 | 3.43E-02 |
| 3916 | IER5 | -0.23 | 3.43E-02 |
| 3917 | TMEM147 | -0.18 | 3.43E-02 |
| 3918 | ATXN1 | -0.24 | 3.43E-02 |
| 3919 | FKBP1A | -0.17 | 3.43E-02 |
| 3920 | WHSC1 | 0.17 | 3.43E-02 |
| 3921 | ZNF141 | 0.18 | 3.43E-02 |
| 3922 | ZC3H11A | -0.12 | 3.43E-02 |
| 3923 | OTOA | 0.18 | 3.43E-02 |
| 3924 | C3orf17 | 0.32 | 3.44E-02 |
| 3925 | KRT7 | -0.16 | 3.44E-02 |
| 3926 | MFSD5 | -0.17 | 3.44E-02 |
| 3927 | HHEX | -0.17 | 3.44E-02 |
| 3928 | GUCA1B | -0.31 | 3.44E-02 |
| 3929 | MRPS33 | 0.13 | 3.44E-02 |
| 3930 | COL23A1 | -0.32 | 3.44E-02 |
| 3931 | SRY | 0.29 | 3.44E-02 |
| 3932 | NUDT16P | 0.17 | 3.44E-02 |
| 3933 | ZDHHC2 | -0.42 | 3.44E-02 |
| 3934 | LOC147299 | 0.33 | 3.45E-02 |
| 3935 | SLC41A2 | -0.38 | 3.45E-02 |
| 3936 | RSPH3 | -0.28 | 3.45E-02 |
| 3937 | LRRC52 | 0.24 | 3.45E-02 |
| 3938 | MBD3 | -0.12 | 3.45E-02 |
| 3939 | CTTNBP2NL | -0.44 | 3.45E-02 |
| 3940 | GPR37L1 | 0.22 | 3.45E-02 |
| 3941 | LGI3 | 0.19 | 3.45E-02 |
| 3942 | CD274 | 0.17 | 3.45E-02 |
| 3943 | GSTM5 | 0.20 | 3.45E-02 |
| 3944 | C5AR1 | 0.29 | 3.45E-02 |
| 3945 | ANKRD47 | 0.23 | 3.45E-02 |
| 3946 | SLC38A3 | 0.20 | 3.45E-02 |
| 3947 | SPAG11B | 0.22 | 3.46E-02 |
| 3948 | STK17A | 0.15 | 3.46E-02 |
| 3949 | SMARCAD1 | -0.16 | 3.46E-02 |
| 3950 | LOC651721 | -0.26 | 3.46E-02 |
| 3951 | RBPJL | 0.26 | 3.46E-02 |
| 3952 | LOC285043 | 0.24 | 3.47E-02 |
| 3953 | OR5J2 | 0.12 | 3.47E-02 |
| 3954 | TRIM31 | 0.34 | 3.47E-02 |
| 3955 | GDF15 | -0.20 | 3.47E-02 |
| 3956 | MXI1 | 0.30 | 3.47E-02 |
| 3957 | SYNJ2 | -0.22 | 3.47E-02 |
| 3958 | MAK | -0.19 | 3.47E-02 |
| 3959 | RNF150 | -0.29 | 3.47E-02 |
| 3960 | KEAP1 | -0.24 | 3.47E-02 |
| 3961 | ARNT | 0.36 | 3.48E-02 |
| 3962 | C1GALT1C1 | -0.27 | 3.48E-02 |
| 3963 | NFATC3 | -0.17 | 3.48E-02 |
| 3964 | LOC285181 | -0.19 | 3.48E-02 |
| 3965 | TGM3 | 0.27 | 3.48E-02 |
| 3966 | NF-E4 | 0.14 | 3.49E-02 |
| 3967 | IGF2AS | 0.49 | 3.49E-02 |
| 3968 | LHX3 | 0.38 | 3.49E-02 |
| 3969 | ATP11C | 0.42 | 3.49E-02 |
| 3970 | PROP1 | 0.19 | 3.49E-02 |
| 3971 | RGL2 | -0.18 | 3.49E-02 |
| 3972 | PDGFRB | -0.38 | 3.49E-02 |
| 3973 | MST1R | -0.29 | 3.49E-02 |
| 3974 | AGXT | 0.31 | 3.50E-02 |
| 3975 | IQCK | -0.25 | 3.50E-02 |
| 3976 | ITGA10 | 0.29 | 3.50E-02 |
| 3977 | EML4 | -0.28 | 3.51E-02 |
| 3978 | MSI2 | 0.37 | 3.51E-02 |
| 3979 | DGKH | -0.38 | 3.51E-02 |
| 3980 | CPT1A | -0.33 | 3.51E-02 |
| 3981 | VWA3B | 0.24 | 3.51E-02 |
| 3982 | NPAS3 | 0.20 | 3.51E-02 |
| 3983 | EDC4 | -0.18 | 3.51E-02 |
| 3984 | ALAD | -0.17 | 3.51E-02 |
| 3985 | HRG | 0.15 | 3.51E-02 |
| 3986 | SNAPC5 | 0.26 | 3.51E-02 |
| 3987 | CCDC69 | 0.14 | 3.51E-02 |
| 3988 | LOC400655 | 0.19 | 3.51E-02 |
| 3989 | F2RL2 | 0.16 | 3.52E-02 |
| 3990 | psiTPTE22 | -0.20 | 3.52E-02 |
| 3991 | ANP32E | 0.18 | 3.52E-02 |
| 3992 | STXBP2 | -0.12 | 3.52E-02 |
| 3993 | CYP2B7P1 | 0.15 | 3.52E-02 |
| 3994 | KIAA1024 | -0.28 | 3.52E-02 |
| 3995 | C16orf78 | 0.17 | 3.52E-02 |
| 3996 | RGS6 | 0.22 | 3.52E-02 |
| 3997 | ROBO3 | 0.24 | 3.52E-02 |
| 3998 | KCNJ1 | 0.21 | 3.52E-02 |
| 3999 | SLC26A2 | -0.24 | 3.52E-02 |
| 4000 | HSH2D | 0.09 | 3.52E-02 |
| 4001 | TMC1 | 0.32 | 3.52E-02 |
| 4002 | LOC407835 | 0.24 | 3.52E-02 |
| 4003 | EPS8L1 | 0.21 | 3.52E-02 |
| 4004 | CLIP2 | -0.21 | 3.52E-02 |
| 4005 | TRIM71 | -0.27 | 3.52E-02 |
| 4006 | NFATC3 | -0.29 | 3.52E-02 |
| 4007 | PLCH1 | -0.35 | 3.52E-02 |
| 4008 | SLC31A1 | -0.30 | 3.52E-02 |
| 4009 | CLEC4D | 0.16 | 3.52E-02 |
| 4010 | TMEM132E | 0.15 | 3.52E-02 |
| 4011 | PSME3 | -0.20 | 3.52E-02 |
| 4012 | GPR78 | 0.20 | 3.53E-02 |
| 4013 | IQSEC1 | -0.16 | 3.53E-02 |
| 4014 | VKORC1 | -0.16 | 3.53E-02 |
| 4015 | VPS33B | -0.13 | 3.53E-02 |
| 4016 | NTN2L | -0.24 | 3.53E-02 |
| 4017 | SCARB2 | -0.23 | 3.53E-02 |
| 4018 | CDC42BPA | -0.37 | 3.53E-02 |
| 4019 | AGPAT3 | -0.37 | 3.53E-02 |
| 4020 | ECHDC2 | -0.14 | 3.54E-02 |
| 4021 | FLJ13236 | -0.23 | 3.54E-02 |
| 4022 | PROK2 | 0.47 | 3.54E-02 |
| 4023 | ITPR3 | -0.28 | 3.54E-02 |
| 4024 | HES4 | 0.29 | 3.54E-02 |
| 4025 | BTF3 | 0.26 | 3.54E-02 |
| 4026 | TLR6 | 0.28 | 3.54E-02 |
| 4027 | ZNF148 | -0.27 | 3.55E-02 |
| 4028 | PPM1D | 0.25 | 3.55E-02 |
| 4029 | MICB | -0.21 | 3.55E-02 |
| 4030 | EHMT2 | -0.19 | 3.55E-02 |
| 4031 | NHLRC2 | -0.22 | 3.55E-02 |
| 4032 | RAMP3 | -0.12 | 3.55E-02 |
| 4033 | C7orf54 | -0.22 | 3.55E-02 |
| 4034 | AR | -0.32 | 3.55E-02 |
| 4035 | FRZB | -0.30 | 3.55E-02 |
| 4036 | KIAA0355 | -0.23 | 3.55E-02 |
| 4037 | UPB1 | 0.26 | 3.55E-02 |
| 4038 | LOC128322 | -0.24 | 3.56E-02 |
| 4039 | LOC728573 | 0.21 | 3.56E-02 |
| 4040 | TNC | -0.24 | 3.56E-02 |
| 4041 | LOC400794 | 0.23 | 3.56E-02 |
| 4042 | ARF4 | 0.27 | 3.56E-02 |
| 4043 | EP400 | 0.15 | 3.56E-02 |
| 4044 | FGF8 | 0.25 | 3.56E-02 |
| 4045 | TMEM51 | -0.23 | 3.57E-02 |
| 4046 | COL1A1 | -0.41 | 3.57E-02 |
| 4047 | SET | -0.23 | 3.57E-02 |
| 4048 | MED14 | 0.10 | 3.57E-02 |
| 4049 | CMIP | -0.22 | 3.57E-02 |
| 4050 | CACNA1E | 0.18 | 3.57E-02 |
| 4051 | PLA2G4E | 0.16 | 3.58E-02 |
| 4052 | MGC70857 | -0.12 | 3.58E-02 |
| 4053 | GABRA1 | 0.46 | 3.58E-02 |
| 4054 | TTC9B | 0.33 | 3.58E-02 |
| 4055 | CLC | 0.17 | 3.59E-02 |
| 4056 | B4GALT5 | -0.26 | 3.59E-02 |
| 4057 | NR2E1 | -0.26 | 3.59E-02 |
| 4058 | TSPAN4 | -0.20 | 3.59E-02 |
| 4059 | SLC7A6 | -0.18 | 3.59E-02 |
| 4060 | CCNL2 | 0.16 | 3.59E-02 |
| 4061 | FAU | 0.13 | 3.59E-02 |
| 4062 | CCDC88A | -0.32 | 3.59E-02 |
| 4063 | CRY1 | -0.35 | 3.59E-02 |
| 4064 | ENTPD6 | -0.24 | 3.59E-02 |
| 4065 | C6orf50 | 0.37 | 3.59E-02 |
| 4066 | LOC283454 | 0.13 | 3.59E-02 |
| 4067 | BAI3 | 0.18 | 3.59E-02 |
| 4068 | HTRA2 | 0.29 | 3.59E-02 |
| 4069 | CCDC130 | -0.18 | 3.59E-02 |
| 4070 | HIST1H2BO | -0.21 | 3.59E-02 |
| 4071 | CD46 | -0.28 | 3.59E-02 |
| 4072 | LOC642924 | 0.20 | 3.60E-02 |
| 4073 | CUL7 | -0.18 | 3.60E-02 |
| 4074 | ACAD11 | -0.32 | 3.60E-02 |
| 4075 | GDPD1 | -0.21 | 3.60E-02 |
| 4076 | KRT37 | 0.15 | 3.60E-02 |
| 4077 | ACSF2 | -0.29 | 3.60E-02 |
| 4078 | GTPBP2 | -0.29 | 3.60E-02 |
| 4079 | C15orf21 | 0.15 | 3.60E-02 |
| 4080 | TARDBP | -0.11 | 3.60E-02 |
| 4081 | LRP5 | -0.28 | 3.60E-02 |
| 4082 | C14orf100 | -0.30 | 3.60E-02 |
| 4083 | CRYBB2 | -0.17 | 3.60E-02 |
| 4084 | CDKN2C | 0.24 | 3.60E-02 |
| 4085 | ALKBH7 | 0.36 | 3.60E-02 |
| 4086 | THOC6 | 0.38 | 3.60E-02 |
| 4087 | CHPF | 0.36 | 3.61E-02 |
| 4088 | ZNF646 | -0.21 | 3.61E-02 |
| 4089 | RPL3 | 0.17 | 3.61E-02 |
| 4090 | ZNF565 | 0.24 | 3.61E-02 |
| 4091 | hCG_1646471 | 0.27 | 3.61E-02 |
| 4092 | LYPD2 | 0.18 | 3.61E-02 |
| 4093 | OR1F1 | 0.20 | 3.61E-02 |
| 4094 | LOXL1 | -0.25 | 3.61E-02 |
| 4095 | LOC441177 | 0.19 | 3.61E-02 |
| 4096 | CHAT | 0.14 | 3.61E-02 |
| 4097 | ACSF2 | -0.18 | 3.61E-02 |
| 4098 | CECR6 | 0.15 | 3.61E-02 |
| 4099 | TCTE3 | 0.17 | 3.62E-02 |
| 4100 | TUFT1 | 0.19 | 3.62E-02 |
| 4101 | CADPS | 0.20 | 3.62E-02 |
| 4102 | TESSP2 | 0.46 | 3.62E-02 |
| 4103 | CCDC7 | 0.21 | 3.62E-02 |
| 4104 | C1orf49 | 0.23 | 3.62E-02 |
| 4105 | GM2A | -0.23 | 3.62E-02 |
| 4106 | SIAE | -0.35 | 3.62E-02 |
| 4107 | PIGY | 0.22 | 3.62E-02 |
| 4108 | NUP153 | -0.15 | 3.62E-02 |
| 4109 | ST6GAL2 | 0.15 | 3.62E-02 |
| 4110 | PRRX1 | 0.40 | 3.62E-02 |
| 4111 | TRAF1 | -0.23 | 3.62E-02 |
| 4112 | NME2 | 0.23 | 3.62E-02 |
| 4113 | CLASP2 | -0.30 | 3.62E-02 |
| 4114 | CYP4F22 | 0.26 | 3.62E-02 |
| 4115 | SPANXB2 | 0.23 | 3.62E-02 |
| 4116 | RP11-297H3.4 | 0.19 | 3.62E-02 |
| 4117 | WDR22 | 0.38 | 3.63E-02 |
| 4118 | SULF2 | -0.30 | 3.63E-02 |
| 4119 | NLRP14 | 0.22 | 3.63E-02 |
| 4120 | CRELD1 | -0.14 | 3.63E-02 |
| 4121 | OR2AG2 | 0.37 | 3.63E-02 |
| 4122 | ATF1 | -0.23 | 3.63E-02 |
| 4123 | OR6C4 | 0.16 | 3.63E-02 |
| 4124 | OSTF1 | 0.19 | 3.63E-02 |
| 4125 | GABBR2 | 0.17 | 3.63E-02 |
| 4126 | ENTPD7 | -0.23 | 3.64E-02 |
| 4127 | SLC6A3 | 0.16 | 3.64E-02 |
| 4128 | OR5K4 | 0.20 | 3.64E-02 |
| 4129 | LOC339807 | 0.34 | 3.64E-02 |
| 4130 | USP28 | -0.29 | 3.64E-02 |
| 4131 | STK4 | 0.38 | 3.64E-02 |
| 4132 | CCDC53 | -0.18 | 3.64E-02 |
| 4133 | LOC283761 | 0.15 | 3.64E-02 |
| 4134 | BAGE3 | -0.18 | 3.65E-02 |
| 4135 | NHEDC2 | -0.42 | 3.65E-02 |
| 4136 | KDELC1 | -0.27 | 3.65E-02 |
| 4137 | LOC653712 | 0.21 | 3.65E-02 |
| 4138 | DRG2 | -0.11 | 3.65E-02 |
| 4139 | WWC2 | -0.17 | 3.65E-02 |
| 4140 | LOC652128 | -0.41 | 3.65E-02 |
| 4141 | FLJ37798 | 0.24 | 3.65E-02 |
| 4142 | LOC349408 | 0.22 | 3.65E-02 |
| 4143 | TLR5 | 0.25 | 3.65E-02 |
| 4144 | SGMS2 | 0.26 | 3.66E-02 |
| 4145 | SSR1 | -0.20 | 3.66E-02 |
| 4146 | MYOCD | 0.23 | 3.66E-02 |
| 4147 | LOC340178 | 0.20 | 3.67E-02 |
| 4148 | OR9G9 | 0.16 | 3.67E-02 |
| 4149 | GUCY2F | 0.14 | 3.67E-02 |
| 4150 | CDR2L | -0.22 | 3.67E-02 |
| 4151 | SLC34A2 | -0.12 | 3.67E-02 |
| 4152 | SNCA | 0.26 | 3.67E-02 |
| 4153 | GAL3ST4 | -0.33 | 3.67E-02 |
| 4154 | XPR1 | -0.29 | 3.67E-02 |
| 4155 | HCG9 | 0.14 | 3.68E-02 |
| 4156 | FLJ39534 | 0.26 | 3.68E-02 |
| 4157 | KRTAP4-10 | 0.22 | 3.68E-02 |
| 4158 | SCML2 | -0.10 | 3.68E-02 |
| 4159 | C1orf77 | -0.22 | 3.68E-02 |
| 4160 | TNFAIP8L2 | 0.27 | 3.68E-02 |
| 4161 | SNTG1 | -0.13 | 3.68E-02 |
| 4162 | KIAA1797 | 0.42 | 3.68E-02 |
| 4163 | HIST1H2BI | 0.24 | 3.68E-02 |
| 4164 | LOC222070 | 0.28 | 3.68E-02 |
| 4165 | HDAC7A | -0.18 | 3.68E-02 |
| 4166 | LIMCH1 | -0.27 | 3.68E-02 |
| 4167 | YPEL1 | 0.22 | 3.69E-02 |
| 4168 | IGSF9 | -0.14 | 3.69E-02 |
| 4169 | TEX28 | 0.18 | 3.69E-02 |
| 4170 | TLX2 | 0.33 | 3.69E-02 |
| 4171 | C6orf167 | -0.26 | 3.69E-02 |
| 4172 | LOC149832 | -0.27 | 3.69E-02 |
| 4173 | TRAF3 | -0.23 | 3.69E-02 |
| 4174 | P2RY4 | 0.22 | 3.69E-02 |
| 4175 | NCOR2 | -0.17 | 3.70E-02 |
| 4176 | PLK3 | -0.19 | 3.70E-02 |
| 4177 | TSPAN31 | -0.20 | 3.70E-02 |
| 4178 | OR2F1 | 0.19 | 3.70E-02 |
| 4179 | PDSS2 | 0.16 | 3.70E-02 |
| 4180 | CRADD | 0.17 | 3.70E-02 |
| 4181 | RPP21 | -0.13 | 3.70E-02 |
| 4182 | TMEM88 | 0.22 | 3.70E-02 |
| 4183 | GSTA3 | 0.21 | 3.70E-02 |
| 4184 | URB1 | 0.33 | 3.71E-02 |
| 4185 | DHDDS | -0.16 | 3.71E-02 |
| 4186 | ZBTB43 | -0.27 | 3.71E-02 |
| 4187 | GANC | 0.32 | 3.71E-02 |
| 4188 | ANKRD11 | -0.32 | 3.71E-02 |
| 4189 | C7orf42 | -0.24 | 3.71E-02 |
| 4190 | SLC9A6 | -0.32 | 3.71E-02 |
| 4191 | HGS | 0.22 | 3.71E-02 |
| 4192 | FOXC1 | -0.28 | 3.71E-02 |
| 4193 | SFRS18 | -0.14 | 3.71E-02 |
| 4194 | AKNA | 0.15 | 3.71E-02 |
| 4195 | FOXK1 | 0.28 | 3.71E-02 |
| 4196 | MST1 | -0.17 | 3.71E-02 |
| 4197 | LOC552889 | -0.19 | 3.71E-02 |
| 4198 | ATP9A | -0.30 | 3.71E-02 |
| 4199 | UBL5 | 0.17 | 3.71E-02 |
| 4200 | RNF207 | 0.18 | 3.72E-02 |
| 4201 | SLC22A11 | 0.42 | 3.72E-02 |
| 4202 | SLC13A2 | 0.21 | 3.72E-02 |
| 4203 | C6orf182 | 0.34 | 3.72E-02 |
| 4204 | TMEM57 | -0.12 | 3.72E-02 |
| 4205 | SBF1 | -0.22 | 3.72E-02 |
| 4206 | PCDHA10 | -0.25 | 3.72E-02 |
| 4207 | BRD3 | -0.21 | 3.72E-02 |
| 4208 | RAE1 | -0.17 | 3.72E-02 |
| 4209 | KIAA1648 | 0.25 | 3.72E-02 |
| 4210 | LOC284964 | 0.17 | 3.73E-02 |
| 4211 | CLEC2D | -0.25 | 3.74E-02 |
| 4212 | MPDU1 | -0.22 | 3.74E-02 |
| 4213 | GEN1 | -0.23 | 3.74E-02 |
| 4214 | KLC4 | -0.33 | 3.74E-02 |
| 4215 | TMEM95 | 0.18 | 3.74E-02 |
| 4216 | HPS4 | -0.16 | 3.74E-02 |
| 4217 | CCRL2 | -0.34 | 3.75E-02 |
| 4218 | CDC42BPG | 0.27 | 3.75E-02 |
| 4219 | ZNF354C | 0.26 | 3.75E-02 |
| 4220 | GNAS | -0.22 | 3.75E-02 |
| 4221 | ERICH1 | -0.14 | 3.75E-02 |
| 4222 | ZNF343 | -0.25 | 3.75E-02 |
| 4223 | SMTN | -0.13 | 3.75E-02 |
| 4224 | IL17RB | -0.15 | 3.75E-02 |
| 4225 | TMEM86B | -0.22 | 3.75E-02 |
| 4226 | SAPS3 | 0.15 | 3.75E-02 |
| 4227 | FOXA3 | 0.16 | 3.75E-02 |
| 4228 | CNNM2 | -0.12 | 3.75E-02 |
| 4229 | STARD4 | 0.20 | 3.76E-02 |
| 4230 | NOS1 | 0.19 | 3.76E-02 |
| 4231 | FXN | 0.28 | 3.76E-02 |
| 4232 | HIST1H4C | 0.31 | 3.76E-02 |
| 4233 | C1R | -0.29 | 3.76E-02 |
| 4234 | C9 | 0.17 | 3.76E-02 |
| 4235 | RGPD8 | -0.27 | 3.76E-02 |
| 4236 | C1GALT1 | -0.32 | 3.76E-02 |
| 4237 | ICA1L | 0.22 | 3.76E-02 |
| 4238 | MRAP | 0.18 | 3.76E-02 |
| 4239 | SLC5A10 | 0.18 | 3.77E-02 |
| 4240 | INSL3 | 0.24 | 3.77E-02 |
| 4241 | CALML5 | 0.28 | 3.77E-02 |
| 4242 | IL31RA | -0.25 | 3.77E-02 |
| 4243 | YOD1 | -0.23 | 3.77E-02 |
| 4244 | APP | -0.22 | 3.77E-02 |
| 4245 | C7orf42 | -0.13 | 3.77E-02 |
| 4246 | LRRC27 | 0.16 | 3.77E-02 |
| 4247 | KRT8 | -0.21 | 3.77E-02 |
| 4248 | SLC15A4 | -0.19 | 3.77E-02 |
| 4249 | TMEM145 | 0.29 | 3.77E-02 |
| 4250 | PHF7 | 0.14 | 3.77E-02 |
| 4251 | SMARCB1 | -0.14 | 3.77E-02 |
| 4252 | OR51B6 | 0.17 | 3.77E-02 |
| 4253 | LPAL2 | 0.17 | 3.77E-02 |
| 4254 | MINA | 0.12 | 3.78E-02 |
| 4255 | KCNJ12 | -0.24 | 3.78E-02 |
| 4256 | MGC45438 | 0.29 | 3.78E-02 |
| 4257 | EFEMP1 | 0.32 | 3.78E-02 |
| 4258 | P2RX3 | 0.17 | 3.78E-02 |
| 4259 | SLC38A4 | 0.22 | 3.78E-02 |
| 4260 | LOC388630 | 0.13 | 3.78E-02 |
| 4261 | MYH6 | 0.28 | 3.79E-02 |
| 4262 | C10orf75 | -0.23 | 3.79E-02 |
| 4263 | SNORD48 | -0.14 | 3.79E-02 |
| 4264 | HMP19 | -0.22 | 3.79E-02 |
| 4265 | LAMP3 | -0.32 | 3.79E-02 |
| 4266 | JARID1B | -0.18 | 3.79E-02 |
| 4267 | TMBIM1 | -0.28 | 3.79E-02 |
| 4268 | SCUBE3 | 0.19 | 3.80E-02 |
| 4269 | LOC220077 | -0.12 | 3.80E-02 |
| 4270 | LDHD | 0.27 | 3.80E-02 |
| 4271 | UBE2H | -0.20 | 3.80E-02 |
| 4272 | MRPL46 | 0.14 | 3.81E-02 |
| 4273 | C4B | -0.22 | 3.81E-02 |
| 4274 | CTRL | -0.25 | 3.81E-02 |
| 4275 | PRKACB | 0.19 | 3.81E-02 |
| 4276 | TMEM140 | -0.31 | 3.81E-02 |
| 4277 | RAVER2 | 0.17 | 3.81E-02 |
| 4278 | LOC131909 | 0.37 | 3.82E-02 |
| 4279 | DCTN5 | -0.16 | 3.82E-02 |
| 4280 | TRPM7 | -0.24 | 3.82E-02 |
| 4281 | OR5AP2 | 0.24 | 3.82E-02 |
| 4282 | LRP6 | -0.34 | 3.82E-02 |
| 4283 | SPEF1 | -0.14 | 3.82E-02 |
| 4284 | FASLG | -0.34 | 3.82E-02 |
| 4285 | PGLYRP1 | 0.23 | 3.83E-02 |
| 4286 | EPHB3 | -0.29 | 3.83E-02 |
| 4287 | ATP5O | 0.16 | 3.83E-02 |
| 4288 | ZIC2 | 0.20 | 3.83E-02 |
| 4289 | PDE3B | 0.33 | 3.83E-02 |
| 4290 | HCFC1R1 | 0.19 | 3.83E-02 |
| 4291 | TM7SF2 | -0.14 | 3.83E-02 |
| 4292 | SCOC | 0.21 | 3.83E-02 |
| 4293 | C6orf112 | 0.17 | 3.83E-02 |
| 4294 | BLID | 0.25 | 3.83E-02 |
| 4295 | TSSK3 | 0.18 | 3.83E-02 |
| 4296 | ABCC5 | -0.38 | 3.83E-02 |
| 4297 | CCDC78 | -0.31 | 3.84E-02 |
| 4298 | L3MBTL2 | -0.13 | 3.84E-02 |
| 4299 | MYL4 | -0.16 | 3.84E-02 |
| 4300 | WDR16 | 0.18 | 3.84E-02 |
| 4301 | FGFR1 | -0.23 | 3.84E-02 |
| 4302 | SNORD58A | -0.36 | 3.84E-02 |
| 4303 | ZNF41 | -0.23 | 3.84E-02 |
| 4304 | CDS2 | -0.31 | 3.84E-02 |
| 4305 | B3GALNT2 | -0.20 | 3.85E-02 |
| 4306 | RHBDF2 | -0.31 | 3.85E-02 |
| 4307 | C10orf65 | -0.27 | 3.85E-02 |
| 4308 | CALM2 | 0.23 | 3.85E-02 |
| 4309 | DLSTP | -0.17 | 3.85E-02 |
| 4310 | LMNA | -0.13 | 3.85E-02 |
| 4311 | ZSCAN4 | -0.17 | 3.85E-02 |
| 4312 | C3orf44 | 0.19 | 3.85E-02 |
| 4313 | CEACAM4 | 0.18 | 3.85E-02 |
| 4314 | E2F2 | 0.16 | 3.85E-02 |
| 4315 | FUT2 | 0.15 | 3.85E-02 |
| 4316 | OR6S1 | 0.20 | 3.85E-02 |
| 4317 | HCN3 | -0.19 | 3.85E-02 |
| 4318 | SIGLEC1 | 0.11 | 3.85E-02 |
| 4319 | GUCA1C | 0.14 | 3.85E-02 |
| 4320 | TLE3 | -0.20 | 3.85E-02 |
| 4321 | KRT37 | 0.26 | 3.85E-02 |
| 4322 | ZNF582 | 0.38 | 3.85E-02 |
| 4323 | CENPK | 0.24 | 3.85E-02 |
| 4324 | FEN1 | -0.12 | 3.85E-02 |
| 4325 | CHST8 | 0.20 | 3.86E-02 |
| 4326 | C1orf141 | 0.16 | 3.86E-02 |
| 4327 | MERTK | -0.14 | 3.86E-02 |
| 4328 | FYN | -0.20 | 3.86E-02 |
| 4329 | NR2C2 | -0.21 | 3.86E-02 |
| 4330 | TNPO1 | -0.26 | 3.86E-02 |
| 4331 | FOXD4L2 | 0.38 | 3.86E-02 |
| 4332 | TCP11L1 | 0.29 | 3.86E-02 |
| 4333 | C15orf17 | -0.23 | 3.86E-02 |
| 4334 | PCDH18 | 0.15 | 3.86E-02 |
| 4335 | PAX3 | 0.16 | 3.86E-02 |
| 4336 | RAG1AP1 | -0.16 | 3.86E-02 |
| 4337 | GABARAPL2 | -0.17 | 3.86E-02 |
| 4338 | TRIM9 | 0.25 | 3.86E-02 |
| 4339 | CRKL | -0.16 | 3.86E-02 |
| 4340 | DGKD | -0.25 | 3.86E-02 |
| 4341 | PLA2G6 | 0.36 | 3.86E-02 |
| 4342 | C1orf81 | 0.12 | 3.86E-02 |
| 4343 | FCAMR | -0.20 | 3.86E-02 |
| 4344 | RAET1K | 0.29 | 3.86E-02 |
| 4345 | GCM2 | 0.17 | 3.87E-02 |
| 4346 | LUZPP1 | 0.24 | 3.87E-02 |
| 4347 | SIX3 | 0.19 | 3.87E-02 |
| 4348 | OR51I1 | -0.10 | 3.87E-02 |
| 4349 | RHOC | -0.20 | 3.87E-02 |
| 4350 | KRT222P | 0.15 | 3.87E-02 |
| 4351 | CCDC94 | 0.19 | 3.87E-02 |
| 4352 | RNASET2 | -0.19 | 3.87E-02 |
| 4353 | REXO1L1 | -0.14 | 3.87E-02 |
| 4354 | ROR1 | -0.49 | 3.87E-02 |
| 4355 | VTI1A | -0.24 | 3.87E-02 |
| 4356 | C4orf30 | -0.21 | 3.87E-02 |
| 4357 | LOC440292 | 0.20 | 3.88E-02 |
| 4358 | LENG4 | -0.20 | 3.88E-02 |
| 4359 | GRB2 | -0.21 | 3.88E-02 |
| 4360 | CASP8 | 0.12 | 3.89E-02 |
| 4361 | HYPE | -0.16 | 3.89E-02 |
| 4362 | DGCR8 | -0.20 | 3.89E-02 |
| 4363 | HGSNAT | -0.45 | 3.89E-02 |
| 4364 | GMIP | -0.21 | 3.89E-02 |
| 4365 | ZNF616 | -0.29 | 3.90E-02 |
| 4366 | KIAA1822 | 0.22 | 3.90E-02 |
| 4367 | NACA2 | 0.20 | 3.90E-02 |
| 4368 | TMEM112 | -0.16 | 3.90E-02 |
| 4369 | COPA | -0.23 | 3.90E-02 |
| 4370 | UNC50 | -0.22 | 3.90E-02 |
| 4371 | C10orf62 | 0.15 | 3.90E-02 |
| 4372 | GNB2 | -0.14 | 3.90E-02 |
| 4373 | RBM24 | 0.21 | 3.90E-02 |
| 4374 | BSND | 0.15 | 3.90E-02 |
| 4375 | PPIAL4 | 0.22 | 3.90E-02 |
| 4376 | TMTC1 | -0.40 | 3.90E-02 |
| 4377 | MAP3K7IP3 | -0.14 | 3.90E-02 |
| 4378 | PPM1B | -0.19 | 3.90E-02 |
| 4379 | GPR124 | -0.33 | 3.91E-02 |
| 4380 | CXCR7 | -0.34 | 3.92E-02 |
| 4381 | FOXD4L4 | 0.15 | 3.92E-02 |
| 4382 | FBN1 | -0.36 | 3.92E-02 |
| 4383 | SPANXB1 | 0.23 | 3.92E-02 |
| 4384 | LOC161635 | 0.20 | 3.92E-02 |
| 4385 | ITGA4 | 0.24 | 3.92E-02 |
| 4386 | DAZ3 | 0.29 | 3.92E-02 |
| 4387 | AMY1B///AMY1A | -0.33 | 3.92E-02 |
| 4388 | WDR4 | -0.21 | 3.92E-02 |
| 4389 | LRRC28 | 0.22 | 3.93E-02 |
| 4390 | KIAA1542 | -0.17 | 3.93E-02 |
| 4391 | LOC728145 | 0.16 | 3.93E-02 |
| 4392 | ANXA8L2 | 0.26 | 3.93E-02 |
| 4393 | GBF1 | -0.19 | 3.93E-02 |
| 4394 | SESN3 | -0.37 | 3.93E-02 |
| 4395 | RNF113B | 0.10 | 3.93E-02 |
| 4396 | C9orf64 | -0.27 | 3.93E-02 |
| 4397 | FAM20C | -0.19 | 3.93E-02 |
| 4398 | FAM116B | -0.17 | 3.93E-02 |
| 4399 | SYCP1 | 0.31 | 3.93E-02 |
| 4400 | ARID1B | 0.30 | 3.93E-02 |
| 4401 | LOC388284 | 0.19 | 3.93E-02 |
| 4402 | CUL4A | 0.17 | 3.93E-02 |
| 4403 | RALGPS2 | 0.13 | 3.94E-02 |
| 4404 | RPS6KA2 | -0.14 | 3.94E-02 |
| 4405 | ZFP28 | 0.35 | 3.94E-02 |
| 4406 | MGC4655 | -0.31 | 3.94E-02 |
| 4407 | LOC387876 | 0.27 | 3.94E-02 |
| 4408 | TFR2 | 0.20 | 3.94E-02 |
| 4409 | PCYT2 | -0.19 | 3.94E-02 |
| 4410 | SPANXF1 | 0.50 | 3.94E-02 |
| 4411 | VLDLR | -0.26 | 3.94E-02 |
| 4412 | SMPD3 | 0.14 | 3.94E-02 |
| 4413 | SEMA3E | 0.21 | 3.94E-02 |
| 4414 | RTN3 | -0.22 | 3.94E-02 |
| 4415 | CXCL11 | 0.28 | 3.95E-02 |
| 4416 | TEX261 | -0.12 | 3.95E-02 |
| 4417 | OGG1 | 0.13 | 3.95E-02 |
| 4418 | PRR12 | 0.18 | 3.95E-02 |
| 4419 | DAPK1 | -0.25 | 3.95E-02 |
| 4420 | C18orf25 | 0.31 | 3.95E-02 |
| 4421 | FLJ16478 | 0.35 | 3.95E-02 |
| 4422 | KRT39 | 0.21 | 3.95E-02 |
| 4423 | RNASE10 | 0.14 | 3.95E-02 |
| 4424 | PLA2G10 | -0.42 | 3.95E-02 |
| 4425 | TTR | -0.24 | 3.95E-02 |
| 4426 | C9orf71 | 0.23 | 3.95E-02 |
| 4427 | C1orf96 | -0.27 | 3.96E-02 |
| 4428 | GPRC5A | -0.37 | 3.96E-02 |
| 4429 | C2orf18 | 0.20 | 3.96E-02 |
| 4430 | KDELR3 | -0.38 | 3.96E-02 |
| 4431 | BDKRB2 | 0.26 | 3.96E-02 |
| 4432 | BZW2 | 0.18 | 3.96E-02 |
| 4433 | STEAP2 | -0.49 | 3.96E-02 |
| 4434 | SUSD2 | 0.25 | 3.96E-02 |
| 4435 | HNRNPA2B1 | -0.27 | 3.96E-02 |
| 4436 | SDF2L1 | -0.17 | 3.96E-02 |
| 4437 | TCL1A | 0.15 | 3.96E-02 |
| 4438 | PRMT7///SLC7A6 | 0.22 | 3.97E-02 |
| 4439 | MYH13 | 0.19 | 3.97E-02 |
| 4440 | DPYS | 0.21 | 3.98E-02 |
| 4441 | UBN1 | -0.17 | 3.98E-02 |
| 4442 | DBI | 0.20 | 3.98E-02 |
| 4443 | YEATS4 | 0.34 | 3.98E-02 |
| 4444 | PRODH | -0.08 | 3.98E-02 |
| 4445 | PPP1R3D | -0.16 | 3.98E-02 |
| 4446 | SLC23A2 | -0.15 | 3.98E-02 |
| 4447 | SLC5A10 | -0.14 | 3.98E-02 |
| 4448 | ZNF562 | -0.18 | 3.99E-02 |
| 4449 | APH1A | 0.25 | 3.99E-02 |
| 4450 | EFCAB1 | 0.17 | 3.99E-02 |
| 4451 | KRT71 | 0.12 | 3.99E-02 |
| 4452 | C5orf36 | 0.50 | 3.99E-02 |
| 4453 | SLC22A13 | 0.15 | 3.99E-02 |
| 4454 | TSKS | 0.14 | 3.99E-02 |
| 4455 | SLC26A2 | 0.17 | 3.99E-02 |
| 4456 | GTF3C6 | 0.18 | 3.99E-02 |
| 4457 | LRAT | 0.17 | 3.99E-02 |
| 4458 | ABCG1 | 0.33 | 4.00E-02 |
| 4459 | CCDC21 | -0.13 | 4.00E-02 |
| 4460 | NID1 | -0.13 | 4.00E-02 |
| 4461 | ARFIP1 | 0.35 | 4.00E-02 |
| 4462 | HOXC11 | 0.27 | 4.00E-02 |
| 4463 | LOC441377 | 0.08 | 4.00E-02 |
| 4464 | CLEC3A | 0.22 | 4.00E-02 |
| 4465 | MBTPS2 | -0.26 | 4.00E-02 |
| 4466 | OR4N2 | 0.15 | 4.00E-02 |
| 4467 | C17orf51 | -0.28 | 4.00E-02 |
| 4468 | NOVA2 | -0.17 | 4.00E-02 |
| 4469 | GTF3C3 | -0.15 | 4.00E-02 |
| 4470 | SLC22A18 | -0.18 | 4.00E-02 |
| 4471 | C19orf42///MED26 | -0.34 | 4.00E-02 |
| 4472 | CLU | -0.26 | 4.01E-02 |
| 4473 | POU2F1 | 0.41 | 4.01E-02 |
| 4474 | WDR3 | -0.13 | 4.01E-02 |
| 4475 | GPR175 | -0.21 | 4.01E-02 |
| 4476 | KIF17 | 0.13 | 4.01E-02 |
| 4477 | C4orf6 | 0.42 | 4.01E-02 |
| 4478 | LOC283693 | 0.13 | 4.01E-02 |
| 4479 | SPRED3 | 0.20 | 4.01E-02 |
| 4480 | WBP2NL | 0.28 | 4.02E-02 |
| 4481 | ATG9B | 0.22 | 4.02E-02 |
| 4482 | EMILIN3 | -0.16 | 4.02E-02 |
| 4483 | GZF1 | -0.25 | 4.02E-02 |
| 4484 | PTPN2 | 0.18 | 4.02E-02 |
| 4485 | SPZ1 | 0.20 | 4.02E-02 |
| 4486 | SHROOM1 | 0.25 | 4.03E-02 |
| 4487 | ZNF605 | -0.24 | 4.03E-02 |
| 4488 | C6orf89 | -0.28 | 4.03E-02 |
| 4489 | MXD4 | -0.14 | 4.03E-02 |
| 4490 | MBOAT1 | 0.18 | 4.03E-02 |
| 4491 | RTBDN | 0.22 | 4.03E-02 |
| 4492 | LOC400620 | 0.22 | 4.04E-02 |
| 4493 | CACNG7 | 0.20 | 4.04E-02 |
| 4494 | ACVR2B | -0.17 | 4.04E-02 |
| 4495 | MECP2 | -0.23 | 4.04E-02 |
| 4496 | CABIN1 | 0.29 | 4.05E-02 |
| 4497 | SPRR4 | 0.15 | 4.05E-02 |
| 4498 | C12orf36 | 0.22 | 4.05E-02 |
| 4499 | RXRG | -0.12 | 4.05E-02 |
| 4500 | OR8D1 | 0.45 | 4.05E-02 |
| 4501 | RBM35B | -0.20 | 4.05E-02 |
| 4502 | FES | 0.15 | 4.05E-02 |
| 4503 | C6orf106 | -0.15 | 4.05E-02 |
| 4504 | RPS3A | 0.18 | 4.05E-02 |
| 4505 | LAMP2 | -0.33 | 4.06E-02 |
| 4506 | C5orf22 | -0.20 | 4.06E-02 |
| 4507 | GPC4 | -0.30 | 4.06E-02 |
| 4508 | GHRHR | 0.15 | 4.06E-02 |
| 4509 | CABP5 | 0.33 | 4.06E-02 |
| 4510 | PSMD8 | -0.19 | 4.06E-02 |
| 4511 | LOC116349 | -0.32 | 4.06E-02 |
| 4512 | TCAM1 | 0.24 | 4.06E-02 |
| 4513 | LOC283575 | 0.20 | 4.07E-02 |
| 4514 | CACNB3 | -0.10 | 4.07E-02 |
| 4515 | RHBDF1 | -0.23 | 4.07E-02 |
| 4516 | ARHGAP24 | 0.15 | 4.07E-02 |
| 4517 | SGCD | -0.31 | 4.07E-02 |
| 4518 | TMEM45A | 0.28 | 4.07E-02 |
| 4519 | OR13H1 | 0.22 | 4.08E-02 |
| 4520 | VWC2 | 0.16 | 4.08E-02 |
| 4521 | ESF1 | -0.20 | 4.08E-02 |
| 4522 | FOXN3 | -0.23 | 4.08E-02 |
| 4523 | CPNE6 | 0.13 | 4.08E-02 |
| 4524 | RPL36 | 0.16 | 4.08E-02 |
| 4525 | MANSC1 | -0.33 | 4.08E-02 |
| 4526 | SUSD4 | -0.13 | 4.09E-02 |
| 4527 | PACSIN2 | -0.23 | 4.09E-02 |
| 4528 | GOLIM4 | -0.30 | 4.09E-02 |
| 4529 | LRFN3 | -0.11 | 4.09E-02 |
| 4530 | ACRC | -0.26 | 4.09E-02 |
| 4531 | TMEM110 | 0.20 | 4.10E-02 |
| 4532 | HINT1 | 0.18 | 4.10E-02 |
| 4533 | LRCH3 | -0.27 | 4.10E-02 |
| 4534 | SC65 | -0.26 | 4.10E-02 |
| 4535 | OR2AK2 | -0.17 | 4.10E-02 |
| 4536 | ADCY9 | -0.24 | 4.10E-02 |
| 4537 | KALRN | 0.30 | 4.10E-02 |
| 4538 | XKR6 | -0.33 | 4.10E-02 |
| 4539 | CNIH2 | 0.26 | 4.10E-02 |
| 4540 | FAM130A2 | 0.21 | 4.10E-02 |
| 4541 | ALDH1L2 | 0.13 | 4.11E-02 |
| 4542 | CELSR3 | -0.17 | 4.11E-02 |
| 4543 | IQGAP1 | -0.22 | 4.11E-02 |
| 4544 | ADRA1D | 0.23 | 4.11E-02 |
| 4545 | XRCC6 | -0.16 | 4.11E-02 |
| 4546 | GPRC5D | 0.22 | 4.11E-02 |
| 4547 | PHCA | -0.30 | 4.12E-02 |
| 4548 | GP2 | 0.23 | 4.12E-02 |
| 4549 | NOL8 | -0.28 | 4.12E-02 |
| 4550 | SREBF2 | -0.14 | 4.12E-02 |
| 4551 | C20orf57 | 0.15 | 4.12E-02 |
| 4552 | ZNF764 | -0.15 | 4.12E-02 |
| 4553 | TFPI | -0.37 | 4.12E-02 |
| 4554 | TMC2 | 0.15 | 4.12E-02 |
| 4555 | NCLN | -0.16 | 4.12E-02 |
| 4556 | ABAT | -0.18 | 4.12E-02 |
| 4557 | LBP | 0.23 | 4.12E-02 |
| 4558 | LOC732014///LOC728170 | 0.16 | 4.12E-02 |
| 4559 | LOC150381 | -0.15 | 4.12E-02 |
| 4560 | TRRAP | -0.18 | 4.12E-02 |
| 4561 | FDFT1 | 0.17 | 4.12E-02 |
| 4562 | ARHGAP26 | 0.14 | 4.12E-02 |
| 4563 | OR8A1 | 0.23 | 4.12E-02 |
| 4564 | FLJ20433 | -0.16 | 4.13E-02 |
| 4565 | MT3 | -0.15 | 4.13E-02 |
| 4566 | KIAA0100 | -0.20 | 4.13E-02 |
| 4567 | WBP4 | -0.21 | 4.13E-02 |
| 4568 | KIAA1706 | 0.18 | 4.13E-02 |
| 4569 | SYN1 | 0.32 | 4.13E-02 |
| 4570 | DLEC1 | -0.19 | 4.14E-02 |
| 4571 | LOC646324 | 0.19 | 4.14E-02 |
| 4572 | PPP1R3F | 0.12 | 4.14E-02 |
| 4573 | WDR52 | 0.14 | 4.14E-02 |
| 4574 | EPHX1 | 0.26 | 4.14E-02 |
| 4575 | PSMD13 | -0.14 | 4.14E-02 |
| 4576 | HSD17B6 | -0.28 | 4.14E-02 |
| 4577 | LOC652993 | -0.23 | 4.14E-02 |
| 4578 | SFRS15 | -0.18 | 4.14E-02 |
| 4579 | SCYL1 | -0.15 | 4.14E-02 |
| 4580 | CATSPER2P1 | -0.23 | 4.14E-02 |
| 4581 | WIPF2 | -0.18 | 4.14E-02 |
| 4582 | HLA-DOB | 0.29 | 4.14E-02 |
| 4583 | KIAA1244 | 0.24 | 4.15E-02 |
| 4584 | LOC284576 | 0.18 | 4.15E-02 |
| 4585 | LRRFIP2 | 0.26 | 4.15E-02 |
| 4586 | ARL6IP5 | -0.25 | 4.15E-02 |
| 4587 | UBA52 | 0.15 | 4.15E-02 |
| 4588 | SAA4 | -0.24 | 4.15E-02 |
| 4589 | STT3B | -0.25 | 4.15E-02 |
| 4590 | LOC440981 | 0.19 | 4.15E-02 |
| 4591 | TMTC3 | -0.29 | 4.16E-02 |
| 4592 | TMEM47 | -0.30 | 4.16E-02 |
| 4593 | FASTK | 0.28 | 4.16E-02 |
| 4594 | LGMN | -0.24 | 4.16E-02 |
| 4595 | ZNF608 | -0.36 | 4.16E-02 |
| 4596 | LOC348817 | 0.17 | 4.16E-02 |
| 4597 | NLRP8 | 0.20 | 4.16E-02 |
| 4598 | RPL24 | 0.19 | 4.17E-02 |
| 4599 | EIF2B5 | 0.37 | 4.17E-02 |
| 4600 | SPSB1 | -0.25 | 4.17E-02 |
| 4601 | FYB | -0.38 | 4.17E-02 |
| 4602 | FSTL1 | -0.25 | 4.18E-02 |
| 4603 | DNAJB11 | -0.15 | 4.18E-02 |
| 4604 | PKN2 | -0.31 | 4.18E-02 |
| 4605 | SERPINB11 | 0.20 | 4.18E-02 |
| 4606 | USP15 | -0.26 | 4.18E-02 |
| 4607 | UGT3A2 | 0.18 | 4.18E-02 |
| 4608 | PDE4D | 0.19 | 4.18E-02 |
| 4609 | LOC338579 | 0.16 | 4.18E-02 |
| 4610 | FLJ13305 | -0.26 | 4.18E-02 |
| 4611 | ZNF343 | -0.27 | 4.18E-02 |
| 4612 | LMLN | -0.19 | 4.18E-02 |
| 4613 | FBXO25 | -0.20 | 4.19E-02 |
| 4614 | SLC27A3 | -0.23 | 4.19E-02 |
| 4615 | ANKS3 | -0.19 | 4.19E-02 |
| 4616 | SLC17A7 | 0.33 | 4.19E-02 |
| 4617 | PPP2R1B | 0.16 | 4.20E-02 |
| 4618 | C20orf20 | -0.16 | 4.20E-02 |
| 4619 | C11orf45 | 0.18 | 4.20E-02 |
| 4620 | SLC1A2 | -0.34 | 4.20E-02 |
| 4621 | PCDH1 | -0.14 | 4.20E-02 |
| 4622 | SPINK7 | 0.34 | 4.20E-02 |
| 4623 | B4GALT7 | 0.24 | 4.21E-02 |
| 4624 | LHX8 | 0.19 | 4.21E-02 |
| 4625 | LOC730101 | -0.17 | 4.21E-02 |
| 4626 | FAM43B | 0.21 | 4.21E-02 |
| 4627 | INPPL1 | -0.19 | 4.21E-02 |
| 4628 | SLC7A1 | -0.35 | 4.21E-02 |
| 4629 | DHTKD1 | -0.19 | 4.22E-02 |
| 4630 | H2BFM | -0.16 | 4.22E-02 |
| 4631 | LYSMD4 | 0.49 | 4.22E-02 |
| 4632 | SLC4A7 | -0.17 | 4.22E-02 |
| 4633 | HEJ1 | 0.14 | 4.22E-02 |
| 4634 | PLG | 0.17 | 4.22E-02 |
| 4635 | ADARB2 | 0.16 | 4.22E-02 |
| 4636 | VCL | -0.23 | 4.22E-02 |
| 4637 | USP26 | 0.16 | 4.22E-02 |
| 4638 | LOC339123 | -0.11 | 4.22E-02 |
| 4639 | ARID5B | -0.31 | 4.23E-02 |
| 4640 | SH3BP5L | -0.21 | 4.23E-02 |
| 4641 | WDR48 | 0.26 | 4.23E-02 |
| 4642 | GP5 | 0.17 | 4.23E-02 |
| 4643 | LOC728315 | 0.16 | 4.23E-02 |
| 4644 | WNT4 | 0.26 | 4.23E-02 |
| 4645 | BAG5 | -0.12 | 4.23E-02 |
| 4646 | GUSB | -0.22 | 4.23E-02 |
| 4647 | KCNJ9 | 0.11 | 4.23E-02 |
| 4648 | PVR | -0.19 | 4.23E-02 |
| 4649 | ERCC4 | 0.17 | 4.23E-02 |
| 4650 | ABHD10 | -0.32 | 4.23E-02 |
| 4651 | CSF2 | 0.41 | 4.24E-02 |
| 4652 | TMEM106A | -0.26 | 4.24E-02 |
| 4653 | LOC401324 | 0.19 | 4.24E-02 |
| 4654 | BCL6B | 0.25 | 4.24E-02 |
| 4655 | ESR1 | 0.35 | 4.24E-02 |
| 4656 | C17orf85 | -0.27 | 4.24E-02 |
| 4657 | CANX | -0.21 | 4.24E-02 |
| 4658 | LRP8 | -0.21 | 4.24E-02 |
| 4659 | hCG_2044152 | 0.16 | 4.25E-02 |
| 4660 | ATF7 | 0.22 | 4.25E-02 |
| 4661 | TRA16 | -0.12 | 4.25E-02 |
| 4662 | MMD2 | 0.11 | 4.25E-02 |
| 4663 | ACTN1 | 0.24 | 4.25E-02 |
| 4664 | ZFHX3 | -0.25 | 4.25E-02 |
| 4665 | GRINA | -0.16 | 4.25E-02 |
| 4666 | PLGLB1 | 0.13 | 4.26E-02 |
| 4667 | LOC147004 | 0.31 | 4.26E-02 |
| 4668 | KLHL14 | 0.19 | 4.26E-02 |
| 4669 | NKX3-1 | -0.15 | 4.26E-02 |
| 4670 | GPX5 | 0.31 | 4.26E-02 |
| 4671 | PRICKLE1 | -0.19 | 4.27E-02 |
| 4672 | LOC284120 | 0.27 | 4.27E-02 |
| 4673 | GNG12 | -0.26 | 4.27E-02 |
| 4674 | HBG2 | 0.15 | 4.27E-02 |
| 4675 | PRPH | -0.11 | 4.27E-02 |
| 4676 | TM2D2 | -0.26 | 4.27E-02 |
| 4677 | UNC5CL | -0.27 | 4.27E-02 |
| 4678 | CDC14A | 0.13 | 4.27E-02 |
| 4679 | CUL5 | -0.18 | 4.27E-02 |
| 4680 | FLJ11184 | -0.31 | 4.27E-02 |
| 4681 | CDKN2D | 0.16 | 4.27E-02 |
| 4682 | SPIRE2 | 0.10 | 4.27E-02 |
| 4683 | GDPD1 | -0.29 | 4.27E-02 |
| 4684 | EME1 | -0.25 | 4.27E-02 |
| 4685 | SLC37A2 | -0.18 | 4.28E-02 |
| 4686 | NRL | -0.27 | 4.28E-02 |
| 4687 | GOT1L1 | 0.21 | 4.28E-02 |
| 4688 | SENP7 | -0.25 | 4.28E-02 |
| 4689 | MAP1LC3A | -0.16 | 4.28E-02 |
| 4690 | OR1J4 | 0.30 | 4.28E-02 |
| 4691 | PGAM2 | 0.19 | 4.28E-02 |
| 4692 | TMIGD2 | 0.22 | 4.28E-02 |
| 4693 | LOC554251 | 0.27 | 4.28E-02 |
| 4694 | NSUN5 | -0.15 | 4.28E-02 |
| 4695 | OTOG | 0.18 | 4.28E-02 |
| 4696 | TTC15 | -0.21 | 4.29E-02 |
| 4697 | POU5F1P4 | -0.15 | 4.29E-02 |
| 4698 | RPL9 | 0.24 | 4.29E-02 |
| 4699 | CST7 | -0.28 | 4.29E-02 |
| 4700 | SRD5A3 | -0.41 | 4.29E-02 |
| 4701 | FCMD | -0.34 | 4.29E-02 |
| 4702 | MTCH2 | 0.19 | 4.30E-02 |
| 4703 | ODF3 | 0.11 | 4.30E-02 |
| 4704 | STT3A | -0.15 | 4.30E-02 |
| 4705 | OTOP2 | 0.20 | 4.30E-02 |
| 4706 | TMEM170 | 0.25 | 4.30E-02 |
| 4707 | MYL6 | 0.18 | 4.30E-02 |
| 4708 | REEP2 | 0.18 | 4.30E-02 |
| 4709 | ARHGAP1 | -0.26 | 4.31E-02 |
| 4710 | FLJ14803 | -0.17 | 4.31E-02 |
| 4711 | C8orf30A | 0.21 | 4.31E-02 |
| 4712 | EPHB6 | -0.11 | 4.31E-02 |
| 4713 | MORN1 | 0.31 | 4.31E-02 |
| 4714 | LRRC37A2 | -0.36 | 4.31E-02 |
| 4715 | CNP | -0.16 | 4.32E-02 |
| 4716 | C1orf35 | -0.16 | 4.32E-02 |
| 4717 | DNAJC5B | 0.22 | 4.32E-02 |
| 4718 | ERN1 | 0.12 | 4.32E-02 |
| 4719 | KIAA0195 | -0.22 | 4.33E-02 |
| 4720 | MBNL1 | -0.35 | 4.33E-02 |
| 4721 | GAS7 | 0.16 | 4.33E-02 |
| 4722 | TMPRSS11A | 0.37 | 4.33E-02 |
| 4723 | CA5A | 0.17 | 4.33E-02 |
| 4724 | ROBO4 | -0.48 | 4.33E-02 |
| 4725 | POLR2J2 | 0.11 | 4.33E-02 |
| 4726 | BTN2A1 | -0.21 | 4.33E-02 |
| 4727 | NFKBIB | 0.35 | 4.33E-02 |
| 4728 | ABCC9 | -0.09 | 4.33E-02 |
| 4729 | PLDN | -0.13 | 4.33E-02 |
| 4730 | TBC1D16 | 0.17 | 4.33E-02 |
| 4731 | XPO5 | -0.20 | 4.33E-02 |
| 4732 | EXOSC6 | -0.11 | 4.33E-02 |
| 4733 | HDAC9 | 0.20 | 4.33E-02 |
| 4734 | SH2D3C | 0.22 | 4.33E-02 |
| 4735 | LOC643623 | 0.22 | 4.33E-02 |
| 4736 | FCHO1 | 0.36 | 4.34E-02 |
| 4737 | AAA1 | 0.23 | 4.34E-02 |
| 4738 | ZNF236 | -0.38 | 4.34E-02 |
| 4739 | IQGAP3 | 0.17 | 4.34E-02 |
| 4740 | APEX2 | -0.14 | 4.34E-02 |
| 4741 | PTGES3 | 0.25 | 4.34E-02 |
| 4742 | KRTAP9-4 | 0.13 | 4.34E-02 |
| 4743 | HMGN2 | 0.15 | 4.34E-02 |
| 4744 | CCDC70 | 0.11 | 4.35E-02 |
| 4745 | EPHA2 | -0.38 | 4.35E-02 |
| 4746 | ATP6V0A4 | -0.12 | 4.35E-02 |
| 4747 | LOC646543 | 0.23 | 4.35E-02 |
| 4748 | PDXK | -0.26 | 4.35E-02 |
| 4749 | STOM | -0.28 | 4.35E-02 |
| 4750 | ZFP36L2 | 0.17 | 4.35E-02 |
| 4751 | RUNDC3A | 0.13 | 4.35E-02 |
| 4752 | SLC2A7 | 0.33 | 4.35E-02 |
| 4753 | KIAA1407 | -0.39 | 4.35E-02 |
| 4754 | OR5L1 | 0.13 | 4.36E-02 |
| 4755 | PACS1 | 0.38 | 4.36E-02 |
| 4756 | HDAC7A | -0.12 | 4.36E-02 |
| 4757 | PDAP1 | -0.13 | 4.36E-02 |
| 4758 | TCF20 | -0.15 | 4.36E-02 |
| 4759 | PSMD1 | -0.20 | 4.36E-02 |
| 4760 | LRRC7 | 0.17 | 4.36E-02 |
| 4761 | PRTG | 0.17 | 4.36E-02 |
| 4762 | TMEM71 | 0.19 | 4.36E-02 |
| 4763 | C1D | 0.18 | 4.37E-02 |
| 4764 | GABRB1 | -0.40 | 4.37E-02 |
| 4765 | TNFSF12 | 0.16 | 4.37E-02 |
| 4766 | IFNAR1 | 0.20 | 4.37E-02 |
| 4767 | TMEM108 | 0.17 | 4.37E-02 |
| 4768 | PI4K2A | 0.22 | 4.38E-02 |
| 4769 | TRA@ | 0.19 | 4.38E-02 |
| 4770 | PCNP | 0.24 | 4.38E-02 |
| 4771 | SCFD1 | 0.16 | 4.38E-02 |
| 4772 | FOXB2 | -0.11 | 4.38E-02 |
| 4773 | LOC552891 | 0.22 | 4.38E-02 |
| 4774 | RPSA | 0.26 | 4.38E-02 |
| 4775 | FAM70A | 0.26 | 4.38E-02 |
| 4776 | R3HCC1 | 0.16 | 4.38E-02 |
| 4777 | LOC285902 | 0.15 | 4.38E-02 |
| 4778 | HSDL1 | -0.19 | 4.38E-02 |
| 4779 | KIF6 | 0.16 | 4.39E-02 |
| 4780 | DIDO1 | -0.26 | 4.39E-02 |
| 4781 | C1orf182 | 0.34 | 4.39E-02 |
| 4782 | EPPK1 | -0.38 | 4.39E-02 |
| 4783 | C1R | -0.31 | 4.40E-02 |
| 4784 | MICAL1 | -0.22 | 4.40E-02 |
| 4785 | C9orf32 | -0.15 | 4.40E-02 |
| 4786 | EEF1A1 | 0.26 | 4.41E-02 |
| 4787 | CLEC4F | 0.14 | 4.41E-02 |
| 4788 | PPP1CA | -0.16 | 4.41E-02 |
| 4789 | C22orf32 | 0.18 | 4.41E-02 |
| 4790 | FRMPD1 | 0.32 | 4.41E-02 |
| 4791 | RSC1A1 | 0.15 | 4.41E-02 |
| 4792 | C1orf63 | -0.18 | 4.41E-02 |
| 4793 | FAM113A | -0.13 | 4.41E-02 |
| 4794 | C7 | 0.23 | 4.42E-02 |
| 4795 | ABCB6 | -0.19 | 4.42E-02 |
| 4796 | ZNF445 | 0.23 | 4.42E-02 |
| 4797 | RIMS4 | 0.16 | 4.42E-02 |
| 4798 | NUP210 | -0.22 | 4.42E-02 |
| 4799 | BCAT1 | 0.25 | 4.42E-02 |
| 4800 | OR2J2 | 0.18 | 4.42E-02 |
| 4801 | ACVRL1 | 0.17 | 4.42E-02 |
| 4802 | ITGA2 | -0.43 | 4.42E-02 |
| 4803 | CLSTN2 | 0.21 | 4.42E-02 |
| 4804 | NFKBIZ | -0.24 | 4.42E-02 |
| 4805 | MUC3A | 0.19 | 4.43E-02 |
| 4806 | NADSYN1 | -0.21 | 4.43E-02 |
| 4807 | C10orf129 | 0.19 | 4.43E-02 |
| 4808 | FABP5L3 | 0.18 | 4.43E-02 |
| 4809 | INPP4A | -0.24 | 4.43E-02 |
| 4810 | ACVR2A | -0.15 | 4.43E-02 |
| 4811 | TRPV2 | -0.18 | 4.43E-02 |
| 4812 | APOC2 | -0.11 | 4.43E-02 |
| 4813 | OSTalpha | 0.47 | 4.43E-02 |
| 4814 | TRIM11 | -0.14 | 4.43E-02 |
| 4815 | ITK | 0.15 | 4.43E-02 |
| 4816 | MUC20 | -0.24 | 4.43E-02 |
| 4817 | NEDD1 | -0.28 | 4.43E-02 |
| 4818 | EIF4G1 | -0.10 | 4.43E-02 |
| 4819 | hCG_1988300 | -0.21 | 4.43E-02 |
| 4820 | RHOT2 | -0.17 | 4.43E-02 |
| 4821 | CYP4F11 | 0.25 | 4.43E-02 |
| 4822 | EML1 | -0.21 | 4.43E-02 |
| 4823 | LRRC16 | -0.25 | 4.43E-02 |
| 4824 | OPN1LW | 0.27 | 4.43E-02 |
| 4825 | UNQ846 | -0.09 | 4.43E-02 |
| 4826 | FTH1 | 0.35 | 4.44E-02 |
| 4827 | LIMD1 | 0.23 | 4.44E-02 |
| 4828 | PIM1 | -0.33 | 4.44E-02 |
| 4829 | ILDR1 | 0.40 | 4.44E-02 |
| 4830 | SSRP1 | -0.11 | 4.44E-02 |
| 4831 | PAXIP1 | -0.14 | 4.44E-02 |
| 4832 | GMFB | 0.20 | 4.44E-02 |
| 4833 | PMCH | 0.29 | 4.45E-02 |
| 4834 | BPHL | 0.11 | 4.45E-02 |
| 4835 | MCOLN2 | -0.27 | 4.45E-02 |
| 4836 | LMBRD1 | -0.16 | 4.45E-02 |
| 4837 | NESPAS | -0.17 | 4.45E-02 |
| 4838 | ZNF224 | -0.13 | 4.45E-02 |
| 4839 | CYP4F8 | -0.11 | 4.45E-02 |
| 4840 | C9orf84 | -0.17 | 4.45E-02 |
| 4841 | SGCZ | 0.35 | 4.45E-02 |
| 4842 | ATG2B | -0.21 | 4.45E-02 |
| 4843 | FUT2 | 0.17 | 4.45E-02 |
| 4844 | SH2D4A | -0.09 | 4.45E-02 |
| 4845 | LOC645257 | -0.12 | 4.45E-02 |
| 4846 | FMOD | -0.42 | 4.45E-02 |
| 4847 | BDKRB1 | 0.22 | 4.45E-02 |
| 4848 | SPECC1 | 0.23 | 4.45E-02 |
| 4849 | C8orf79 | 0.10 | 4.46E-02 |
| 4850 | PHIP | -0.28 | 4.46E-02 |
| 4851 | RAB7L1 | -0.18 | 4.46E-02 |
| 4852 | HS6ST1 | 0.24 | 4.46E-02 |
| 4853 | SLC30A7 | -0.27 | 4.46E-02 |
| 4854 | PGD | -0.25 | 4.46E-02 |
| 4855 | SH3PXD2B | -0.12 | 4.46E-02 |
| 4856 | PCGF2 | 0.24 | 4.46E-02 |
| 4857 | TNPO2 | 0.13 | 4.46E-02 |
| 4858 | CENPT | 0.30 | 4.46E-02 |
| 4859 | DNAH8 | 0.33 | 4.46E-02 |
| 4860 | LOC144874 | -0.24 | 4.46E-02 |
| 4861 | TGIF2LX | 0.21 | 4.46E-02 |
| 4862 | SUGT1 | -0.30 | 4.46E-02 |
| 4863 | RENBP | 0.13 | 4.46E-02 |
| 4864 | SLC28A2 | 0.17 | 4.46E-02 |
| 4865 | PLCB2 | -0.17 | 4.46E-02 |
| 4866 | LOC391322 | -0.14 | 4.46E-02 |
| 4867 | LOC152485 | -0.14 | 4.47E-02 |
| 4868 | YES1 | 0.20 | 4.47E-02 |
| 4869 | PALM | -0.14 | 4.47E-02 |
| 4870 | C8orf37 | 0.24 | 4.47E-02 |
| 4871 | SLC35B4 | -0.18 | 4.48E-02 |
| 4872 | ZNF253 | -0.23 | 4.48E-02 |
| 4873 | SLC12A6 | -0.27 | 4.48E-02 |
| 4874 | KRT6B | -0.19 | 4.48E-02 |
| 4875 | C1orf183 | 0.25 | 4.48E-02 |
| 4876 | USPL1 | -0.19 | 4.48E-02 |
| 4877 | GDF5 | 0.31 | 4.48E-02 |
| 4878 | GINS1 | -0.13 | 4.48E-02 |
| 4879 | SOX12 | 0.16 | 4.48E-02 |
| 4880 | HK1 | -0.15 | 4.48E-02 |
| 4881 | MAT1A | 0.25 | 4.48E-02 |
| 4882 | CXorf18 | 0.15 | 4.48E-02 |
| 4883 | RNF121 | 0.42 | 4.48E-02 |
| 4884 | SLC26A4 | -0.23 | 4.49E-02 |
| 4885 | PRUNE | -0.17 | 4.49E-02 |
| 4886 | SMAP1L | -0.22 | 4.49E-02 |
| 4887 | PIM3 | -0.17 | 4.49E-02 |
| 4888 | C11orf10 | 0.10 | 4.49E-02 |
| 4889 | HOMER3 | -0.21 | 4.49E-02 |
| 4890 | RDBP | -0.17 | 4.49E-02 |
| 4891 | PTAFR | 0.20 | 4.49E-02 |
| 4892 | COL10A1 | 0.21 | 4.49E-02 |
| 4893 | HEATR5B | -0.25 | 4.50E-02 |
| 4894 | PRO2852 | -0.20 | 4.50E-02 |
| 4895 | C1orf100 | 0.16 | 4.50E-02 |
| 4896 | APOM | 0.17 | 4.50E-02 |
| 4897 | NEK6 | 0.19 | 4.50E-02 |
| 4898 | TECTB | 0.17 | 4.50E-02 |
| 4899 | WDR90 | -0.20 | 4.50E-02 |
| 4900 | DNAJB12 | -0.27 | 4.50E-02 |
| 4901 | EFNB3 | 0.14 | 4.50E-02 |
| 4902 | AYTL2 | -0.18 | 4.50E-02 |
| 4903 | LENG9 | 0.23 | 4.50E-02 |
| 4904 | NOX5 | 0.15 | 4.50E-02 |
| 4905 | MXRA8 | -0.22 | 4.50E-02 |
| 4906 | LOC90835 | 0.13 | 4.50E-02 |
| 4907 | ZNF806 | 0.14 | 4.51E-02 |
| 4908 | GJA10 | 0.13 | 4.51E-02 |
| 4909 | ZC3H12A | -0.23 | 4.51E-02 |
| 4910 | RPS7 | 0.16 | 4.51E-02 |
| 4911 | KRTAP1-1 | 0.12 | 4.51E-02 |
| 4912 | RIOK3 | -0.36 | 4.51E-02 |
| 4913 | FARSB | -0.29 | 4.51E-02 |
| 4914 | LONP2 | -0.25 | 4.51E-02 |
| 4915 | PLA2G10 | -0.34 | 4.52E-02 |
| 4916 | SEMA4D | -0.30 | 4.52E-02 |
| 4917 | CYP24A1 | -0.30 | 4.52E-02 |
| 4918 | KLF6 | -0.23 | 4.52E-02 |
| 4919 | C12orf48 | -0.22 | 4.53E-02 |
| 4920 | C14orf155 | 0.17 | 4.53E-02 |
| 4921 | MMP15 | -0.17 | 4.53E-02 |
| 4922 | TIMELESS | -0.20 | 4.53E-02 |
| 4923 | NMBR | 0.28 | 4.53E-02 |
| 4924 | PHLPP | -0.31 | 4.53E-02 |
| 4925 | C1orf159 | -0.13 | 4.53E-02 |
| 4926 | FAM92A1 | 0.17 | 4.53E-02 |
| 4927 | SKIP | -0.17 | 4.53E-02 |
| 4928 | FAM80B | -0.26 | 4.53E-02 |
| 4929 | GRM4 | 0.16 | 4.53E-02 |
| 4930 | TCEB3C | 0.12 | 4.54E-02 |
| 4931 | FLJ40759 | 0.18 | 4.54E-02 |
| 4932 | TM4SF1 | -0.27 | 4.54E-02 |
| 4933 | SCN2A | 0.19 | 4.54E-02 |
| 4934 | STK17B | -0.20 | 4.54E-02 |
| 4935 | UBE1DC1 | -0.23 | 4.54E-02 |
| 4936 | TBC1D5 | -0.44 | 4.54E-02 |
| 4937 | RRAD | -0.30 | 4.55E-02 |
| 4938 | XPO6 | -0.17 | 4.55E-02 |
| 4939 | KCTD18 | 0.19 | 4.55E-02 |
| 4940 | SGCB | -0.24 | 4.56E-02 |
| 4941 | HABP2 | -0.10 | 4.56E-02 |
| 4942 | NFS1 | -0.11 | 4.56E-02 |
| 4943 | FGF14 | 0.19 | 4.56E-02 |
| 4944 | PPAP2C | -0.35 | 4.56E-02 |
| 4945 | TAF15 | -0.19 | 4.57E-02 |
| 4946 | SYNGR1 | -0.23 | 4.57E-02 |
| 4947 | PHLDB1 | -0.23 | 4.57E-02 |
| 4948 | MYO1G | -0.11 | 4.58E-02 |
| 4949 | CFB | -0.31 | 4.58E-02 |
| 4950 | KRT9 | 0.29 | 4.58E-02 |
| 4951 | RNF14 | 0.18 | 4.58E-02 |
| 4952 | TRAM1 | -0.19 | 4.58E-02 |
| 4953 | OR52B2 | 0.12 | 4.58E-02 |
| 4954 | SLC17A5 | -0.25 | 4.58E-02 |
| 4955 | TSGA10IP | 0.15 | 4.58E-02 |
| 4956 | VEGFC | -0.24 | 4.59E-02 |
| 4957 | TMED1 | -0.17 | 4.60E-02 |
| 4958 | COL5A1 | -0.20 | 4.60E-02 |
| 4959 | LOC284702 | -0.22 | 4.60E-02 |
| 4960 | ARSB | -0.17 | 4.61E-02 |
| 4961 | CTSB | -0.24 | 4.61E-02 |
| 4962 | UBL4B | 0.14 | 4.61E-02 |
| 4963 | BAP1 | -0.11 | 4.61E-02 |
| 4964 | VRK3 | 0.17 | 4.61E-02 |
| 4965 | C1orf9 | -0.23 | 4.62E-02 |
| 4966 | LOC55908 | 0.33 | 4.62E-02 |
| 4967 | EPHX1 | 0.24 | 4.62E-02 |
| 4968 | RBM33 | -0.29 | 4.63E-02 |
| 4969 | DCTN2 | -0.17 | 4.63E-02 |
| 4970 | ERBB4 | 0.27 | 4.63E-02 |
| 4971 | SNN | -0.26 | 4.63E-02 |
| 4972 | IGFL3 | 0.20 | 4.64E-02 |
| 4973 | OSTM1 | -0.18 | 4.64E-02 |
| 4974 | TNRC8 | -0.33 | 4.64E-02 |
| 4975 | THSD1 | -0.30 | 4.64E-02 |
| 4976 | ASH1L | 0.14 | 4.64E-02 |
| 4977 | ILF3 | -0.17 | 4.64E-02 |
| 4978 | TMEM33 | -0.25 | 4.64E-02 |
| 4979 | KIF6 | 0.17 | 4.64E-02 |
| 4980 | RPN2 | -0.32 | 4.64E-02 |
| 4981 | KRTAP13-3 | 0.13 | 4.64E-02 |
| 4982 | PBRM1 | -0.34 | 4.64E-02 |
| 4983 | LRWD1 | 0.24 | 4.64E-02 |
| 4984 | OR4D1 | 0.10 | 4.64E-02 |
| 4985 | PHKG2 | -0.11 | 4.64E-02 |
| 4986 | TTYH3 | -0.17 | 4.64E-02 |
| 4987 | C21orf51 | 0.16 | 4.64E-02 |
| 4988 | RPL23 | 0.26 | 4.64E-02 |
| 4989 | PHF8 | -0.15 | 4.64E-02 |
| 4990 | IL12RB1 | -0.11 | 4.65E-02 |
| 4991 | SMC4 | -0.21 | 4.65E-02 |
| 4992 | LOC642587 | 0.20 | 4.65E-02 |
| 4993 | SLC20A1 | -0.31 | 4.65E-02 |
| 4994 | GPATCH8 | 0.23 | 4.65E-02 |
| 4995 | FLJ31222 | 0.17 | 4.65E-02 |
| 4996 | ARSH | 0.19 | 4.65E-02 |
| 4997 | GALNT4 | -0.31 | 4.65E-02 |
| 4998 | CYP2E1 | -0.24 | 4.65E-02 |
| 4999 | DPYSL3 | 0.23 | 4.65E-02 |
| 5000 | PUSL1 | -0.20 | 4.65E-02 |
| 5001 | KIAA0692 | -0.26 | 4.65E-02 |
| 5002 | SSTR5 | 0.12 | 4.66E-02 |
| 5003 | FZD10 | -0.17 | 4.66E-02 |
| 5004 | C4A | -0.31 | 4.66E-02 |
| 5005 | MRE11A | -0.19 | 4.66E-02 |
| 5006 | TIAM1 | -0.16 | 4.66E-02 |
| 5007 | OSBPL10 | -0.23 | 4.66E-02 |
| 5008 | LOC283710 | 0.17 | 4.66E-02 |
| 5009 | PTPLB | -0.23 | 4.66E-02 |
| 5010 | EDG3 | 0.11 | 4.67E-02 |
| 5011 | NPAS2 | -0.30 | 4.67E-02 |
| 5012 | PCDHB13 | -0.15 | 4.67E-02 |
| 5013 | CD34 | -0.45 | 4.67E-02 |
| 5014 | C9orf165 | 0.17 | 4.68E-02 |
| 5015 | POLE | 0.18 | 4.68E-02 |
| 5016 | GPR75 | -0.14 | 4.68E-02 |
| 5017 | PRRT2 | -0.23 | 4.68E-02 |
| 5018 | PSCD3 | 0.45 | 4.69E-02 |
| 5019 | MIER1 | 0.29 | 4.69E-02 |
| 5020 | KIDINS220 | -0.26 | 4.69E-02 |
| 5021 | ZNF175 | -0.13 | 4.69E-02 |
| 5022 | UNQ9391 | -0.13 | 4.69E-02 |
| 5023 | CUEDC1 | -0.26 | 4.69E-02 |
| 5024 | MGC34829 | -0.11 | 4.69E-02 |
| 5025 | GJA1 | -0.36 | 4.69E-02 |
| 5026 | SKI | 0.17 | 4.69E-02 |
| 5027 | ERLIN2 | -0.25 | 4.70E-02 |
| 5028 | HAL | 0.25 | 4.70E-02 |
| 5029 | ZCCHC13 | 0.18 | 4.70E-02 |
| 5030 | HNT | 0.23 | 4.70E-02 |
| 5031 | SLC13A4 | 0.19 | 4.70E-02 |
| 5032 | L3MBTL | 0.32 | 4.71E-02 |
| 5033 | ADAM33 | 0.29 | 4.71E-02 |
| 5034 | FGD4 | -0.30 | 4.71E-02 |
| 5035 | RPL32P3 | 0.25 | 4.71E-02 |
| 5036 | ETNK1 | -0.37 | 4.71E-02 |
| 5037 | PLCB3 | -0.14 | 4.71E-02 |
| 5038 | EMG1 | -0.34 | 4.71E-02 |
| 5039 | SLC6A12 | 0.36 | 4.71E-02 |
| 5040 | KIF5A | 0.15 | 4.71E-02 |
| 5041 | ZNF160 | -0.39 | 4.71E-02 |
| 5042 | ZNF294 | -0.21 | 4.71E-02 |
| 5043 | SLC13A4 | 0.23 | 4.71E-02 |
| 5044 | MAGI3 | -0.18 | 4.71E-02 |
| 5045 | HAP1 | 0.16 | 4.72E-02 |
| 5046 | RGPD5 | -0.26 | 4.72E-02 |
| 5047 | GPR177 | -0.29 | 4.72E-02 |
| 5048 | TULP4 | -0.29 | 4.72E-02 |
| 5049 | ALX4 | -0.15 | 4.72E-02 |
| 5050 | KRTAP2-2 | 0.16 | 4.72E-02 |
| 5051 | C1QTNF8 | 0.13 | 4.72E-02 |
| 5052 | TRAF3 | -0.35 | 4.72E-02 |
| 5053 | PRX | 0.30 | 4.72E-02 |
| 5054 | FAM8A1 | -0.25 | 4.72E-02 |
| 5055 | MICAL2 | -0.25 | 4.73E-02 |
| 5056 | OR52W1 | 0.12 | 4.73E-02 |
| 5057 | NOX4 | -0.34 | 4.73E-02 |
| 5058 | INHBC | 0.18 | 4.73E-02 |
| 5059 | ASB16 | 0.22 | 4.74E-02 |
| 5060 | PDPN | -0.34 | 4.74E-02 |
| 5061 | RAB4B | -0.13 | 4.74E-02 |
| 5062 | GPR151 | 0.23 | 4.74E-02 |
| 5063 | KIR2DS4 | 0.14 | 4.74E-02 |
| 5064 | DPH5 | -0.24 | 4.74E-02 |
| 5065 | LOC644090 | 0.18 | 4.74E-02 |
| 5066 | PDE9A | -0.18 | 4.75E-02 |
| 5067 | KIAA0513 | -0.29 | 4.75E-02 |
| 5068 | FLJ31945 | 0.22 | 4.75E-02 |
| 5069 | TM4SF1 | -0.27 | 4.75E-02 |
| 5070 | APOE | -0.17 | 4.75E-02 |
| 5071 | BEGAIN | 0.10 | 4.75E-02 |
| 5072 | PIGV | 0.25 | 4.75E-02 |
| 5073 | CDC26 | 0.18 | 4.75E-02 |
| 5074 | NLGN2 | -0.19 | 4.76E-02 |
| 5075 | PRSSL1 | 0.19 | 4.76E-02 |
| 5076 | ANKRD20A1 | -0.18 | 4.76E-02 |
| 5077 | CST4 | -0.16 | 4.76E-02 |
| 5078 | HSPA1B | -0.25 | 4.76E-02 |
| 5079 | MACROD1 | -0.17 | 4.76E-02 |
| 5080 | TNFSF12 | 0.16 | 4.76E-02 |
| 5081 | FNDC7 | 0.22 | 4.76E-02 |
| 5082 | ZNF676 | -0.22 | 4.76E-02 |
| 5083 | ST8SIA5 | 0.25 | 4.76E-02 |
| 5084 | LOC339666 | 0.18 | 4.76E-02 |
| 5085 | C2orf28 | -0.13 | 4.76E-02 |
| 5086 | ZNF304 | -0.34 | 4.76E-02 |
| 5087 | SCGB2A1 | 0.18 | 4.76E-02 |
| 5088 | ZNF331 | -0.30 | 4.76E-02 |
| 5089 | C18orf54 | 0.17 | 4.76E-02 |
| 5090 | RARRES1 | 0.38 | 4.77E-02 |
| 5091 | UNC13A | -0.18 | 4.77E-02 |
| 5092 | ANK3 | -0.21 | 4.77E-02 |
| 5093 | ST7 | -0.27 | 4.77E-02 |
| 5094 | FRAP1 | -0.22 | 4.77E-02 |
| 5095 | CLK3 | -0.18 | 4.77E-02 |
| 5096 | SULT2B1 | 0.33 | 4.78E-02 |
| 5097 | FKBP6 | 0.16 | 4.78E-02 |
| 5098 | M-RIP | -0.25 | 4.78E-02 |
| 5099 | PTPRU | -0.10 | 4.78E-02 |
| 5100 | CALML3 | 0.39 | 4.79E-02 |
| 5101 | MSI2 | -0.18 | 4.79E-02 |
| 5102 | MTCH1 | -0.14 | 4.79E-02 |
| 5103 | RIMS3 | 0.19 | 4.79E-02 |
| 5104 | VANGL2 | -0.24 | 4.79E-02 |
| 5105 | GPR101 | 0.17 | 4.79E-02 |
| 5106 | URLC9 | -0.18 | 4.79E-02 |
| 5107 | MIZF | -0.17 | 4.79E-02 |
| 5108 | ANKRD15 | 0.13 | 4.79E-02 |
| 5109 | ABCA10 | -0.54 | 4.79E-02 |
| 5110 | CHRND | 0.41 | 4.79E-02 |
| 5111 | PCCA | 0.13 | 4.79E-02 |
| 5112 | SLC19A2 | -0.29 | 4.79E-02 |
| 5113 | FAM40A | -0.20 | 4.79E-02 |
| 5114 | CHRNA2 | 0.16 | 4.79E-02 |
| 5115 | ACTL8 | 0.18 | 4.79E-02 |
| 5116 | TOB1 | 0.29 | 4.79E-02 |
| 5117 | NCALD | 0.13 | 4.79E-02 |
| 5118 | NRP1 | -0.34 | 4.79E-02 |
| 5119 | FLJ40244 | 0.17 | 4.79E-02 |
| 5120 | WNK2 | 0.21 | 4.79E-02 |
| 5121 | ZNF486 | -0.45 | 4.79E-02 |
| 5122 | FOSL2 | 0.23 | 4.79E-02 |
| 5123 | CALN1 | 0.13 | 4.80E-02 |
| 5124 | EHMT1 | -0.16 | 4.80E-02 |
| 5125 | LOC284274 | 0.19 | 4.80E-02 |
| 5126 | JMJD2B | -0.31 | 4.81E-02 |
| 5127 | MEX3A | -0.25 | 4.81E-02 |
| 5128 | PLSCR2 | 0.35 | 4.81E-02 |
| 5129 | LSR | -0.29 | 4.81E-02 |
| 5130 | FIZ1 | 0.24 | 4.81E-02 |
| 5131 | MSGN1 | 0.19 | 4.81E-02 |
| 5132 | LACTB | 0.23 | 4.81E-02 |
| 5133 | L2HGDH | -0.33 | 4.82E-02 |
| 5134 | ASB11 | 0.17 | 4.82E-02 |
| 5135 | PRDX3 | 0.20 | 4.82E-02 |
| 5136 | CALM3 | -0.14 | 4.82E-02 |
| 5137 | CLTC | -0.12 | 4.82E-02 |
| 5138 | SMAD2 | 0.15 | 4.82E-02 |
| 5139 | RBM32A | 0.11 | 4.83E-02 |
| 5140 | SMARCE1 | -0.21 | 4.83E-02 |
| 5141 | DPP8 | -0.21 | 4.83E-02 |
| 5142 | COL9A1 | -0.27 | 4.83E-02 |
| 5143 | OR6C65 | 0.23 | 4.83E-02 |
| 5144 | COL15A1 | -0.42 | 4.83E-02 |
| 5145 | C1orf106 | -0.23 | 4.83E-02 |
| 5146 | HSD17B14 | 0.23 | 4.83E-02 |
| 5147 | HDAC9 | 0.16 | 4.83E-02 |
| 5148 | ASF1A | 0.45 | 4.83E-02 |
| 5149 | LRAP | -0.43 | 4.83E-02 |
| 5150 | LOC645733 | -0.33 | 4.84E-02 |
| 5151 | PEA15 | -0.18 | 4.84E-02 |
| 5152 | SFTPD | 0.23 | 4.84E-02 |
| 5153 | LOC388444 | 0.17 | 4.84E-02 |
| 5154 | RNMT | -0.19 | 4.84E-02 |
| 5155 | TMEM49 | -0.26 | 4.85E-02 |
| 5156 | UGT1A8 | -0.42 | 4.85E-02 |
| 5157 | KRT6A | -0.23 | 4.85E-02 |
| 5158 | CLGN | 0.16 | 4.85E-02 |
| 5159 | GAS5 | -0.22 | 4.85E-02 |
| 5160 | SLC25A28 | -0.16 | 4.85E-02 |
| 5161 | DAPL1 | 0.13 | 4.85E-02 |
| 5162 | IFI6 | -0.31 | 4.85E-02 |
| 5163 | C20orf85 | 0.22 | 4.85E-02 |
| 5164 | UBR1 | -0.28 | 4.85E-02 |
| 5165 | KRTAP2-2 | 0.24 | 4.85E-02 |
| 5166 | SNX21 | 0.39 | 4.85E-02 |
| 5167 | PSME1 | 0.31 | 4.86E-02 |
| 5168 | SOS1 | 0.32 | 4.86E-02 |
| 5169 | PI4KAP2 | -0.10 | 4.86E-02 |
| 5170 | LNPEP | -0.24 | 4.87E-02 |
| 5171 | PLEKHG2 | -0.23 | 4.87E-02 |
| 5172 | PCNA | -0.19 | 4.87E-02 |
| 5173 | SEMG2 | 0.14 | 4.87E-02 |
| 5174 | C8orf55 | -0.24 | 4.87E-02 |
| 5175 | SHOX | 0.19 | 4.88E-02 |
| 5176 | HPSE | -0.38 | 4.88E-02 |
| 5177 | TRIM59 | 0.19 | 4.88E-02 |
| 5178 | HIST1H4D | -0.32 | 4.88E-02 |
| 5179 | OR2T8 | 0.22 | 4.89E-02 |
| 5180 | WFIKKN2 | -0.13 | 4.89E-02 |
| 5181 | C3orf16 | 0.11 | 4.89E-02 |
| 5182 | P11 | 0.17 | 4.89E-02 |
| 5183 | CCL23 | -0.40 | 4.90E-02 |
| 5184 | DAXX | -0.10 | 4.90E-02 |
| 5185 | C8orf73 | 0.20 | 4.90E-02 |
| 5186 | C10orf67 | 0.16 | 4.90E-02 |
| 5187 | PPAP2B | -0.22 | 4.90E-02 |
| 5188 | STAT6 | -0.22 | 4.90E-02 |
| 5189 | ATF2 | -0.27 | 4.90E-02 |
| 5190 | LOC441086 | 0.08 | 4.90E-02 |
| 5191 | CDH3 | -0.43 | 4.91E-02 |
| 5192 | PCDH15 | 0.10 | 4.91E-02 |
| 5193 | hCG_40738 | -0.17 | 4.91E-02 |
| 5194 | DNAJC18 | -0.26 | 4.92E-02 |
| 5195 | GRIK5 | 0.19 | 4.92E-02 |
| 5196 | CRTC3 | -0.14 | 4.92E-02 |
| 5197 | ZBTB45 | -0.15 | 4.92E-02 |
| 5198 | VSTM1 | 0.15 | 4.92E-02 |
| 5199 | RPS4X | 0.22 | 4.92E-02 |
| 5200 | TNFAIP2 | -0.26 | 4.92E-02 |
| 5201 | SLC37A1 | -0.23 | 4.93E-02 |
| 5202 | FAM133A | 0.16 | 4.93E-02 |
| 5203 | SMNDC1 | 0.20 | 4.93E-02 |
| 5204 | MAGED2 | -0.30 | 4.93E-02 |
| 5205 | DIDO1 | -0.26 | 4.93E-02 |
| 5206 | DNAJC7 | -0.41 | 4.93E-02 |
| 5207 | LRRC17 | -0.22 | 4.93E-02 |
| 5208 | FLJ11827 | 0.24 | 4.93E-02 |
| 5209 | SPIB | 0.34 | 4.93E-02 |
| 5210 | GTF2H5 | 0.15 | 4.94E-02 |
| 5211 | KRT83 | 0.17 | 4.94E-02 |
| 5212 | NMRAL1 | -0.14 | 4.94E-02 |
| 5213 | CCNB3 | -0.28 | 4.94E-02 |
| 5214 | MLL4 | -0.15 | 4.94E-02 |
| 5215 | PAPPA | 0.22 | 4.94E-02 |
| 5216 | CAPN12 | 0.15 | 4.94E-02 |
| 5217 | LOC338620 | -0.17 | 4.94E-02 |
| 5218 | LOC647309 | 0.23 | 4.94E-02 |
| 5219 | ITGA6 | -0.23 | 4.94E-02 |
| 5220 | METTL1 | 0.14 | 4.94E-02 |
| 5221 | COX7A2L | 0.16 | 4.94E-02 |
| 5222 | IGHV3-48 | -0.22 | 4.94E-02 |
| 5223 | CNTNAP2 | 0.32 | 4.94E-02 |
| 5224 | CCL16 | 0.30 | 4.94E-02 |
| 5225 | DHX35 | -0.28 | 4.94E-02 |
| 5226 | GRK7 | -0.23 | 4.94E-02 |
| 5227 | UHMK1 | -0.33 | 4.94E-02 |
| 5228 | NF2 | 0.16 | 4.94E-02 |
| 5229 | GZMA | 0.20 | 4.95E-02 |
| 5230 | LOC645478 | 0.35 | 4.95E-02 |
| 5231 | EGFL11 | 0.18 | 4.95E-02 |
| 5232 | GUCA2A | 0.27 | 4.95E-02 |
| 5233 | RNF7 | 0.18 | 4.95E-02 |
| 5234 | F13B | 0.14 | 4.95E-02 |
| 5235 | HTRA3 | -0.23 | 4.95E-02 |
| 5236 | C9orf167 | -0.26 | 4.95E-02 |
| 5237 | FEM1A | -0.16 | 4.95E-02 |
| 5238 | FOXN1 | 0.18 | 4.95E-02 |
| 5239 | SLC19A2 | -0.27 | 4.95E-02 |
| 5240 | PEA15 | -0.24 | 4.95E-02 |
| 5241 | LEPREL1 | -0.27 | 4.95E-02 |
| 5242 | RSPRY1 | -0.24 | 4.95E-02 |
| 5243 | TCF7L2 | -0.26 | 4.95E-02 |
| 5244 | LOC493754 | -0.30 | 4.95E-02 |
| 5245 | TEX14 | 0.20 | 4.95E-02 |
| 5246 | PTPN9 | -0.20 | 4.95E-02 |
| 5247 | GLG1 | -0.12 | 4.95E-02 |
| 5248 | CCDC109B | 0.26 | 4.95E-02 |
| 5249 | ZSCAN18 | -0.18 | 4.95E-02 |
| 5250 | TAL2 | 0.20 | 4.96E-02 |
| 5251 | HDGF2 | -0.14 | 4.96E-02 |
| 5252 | SERAC1 | -0.26 | 4.96E-02 |
| 5253 | PI4KA | -0.11 | 4.97E-02 |
| 5254 | CATR1 | 0.18 | 4.97E-02 |
| 5255 | PLGLA1 | 0.35 | 4.97E-02 |
| 5256 | SHH | 0.44 | 4.97E-02 |
| 5257 | ECEL1 | 0.22 | 4.97E-02 |
| 5258 | NUP50 | -0.19 | 4.97E-02 |
| 5259 | ACTR8 | 0.37 | 4.97E-02 |
| 5260 | OTUD7B | -0.16 | 4.97E-02 |
| 5261 | CD1B | -0.20 | 4.97E-02 |
| 5262 | MGC12982 | 0.30 | 4.97E-02 |
| 5263 | TESK1 | -0.20 | 4.97E-02 |
| 5264 | FXC1 | 0.16 | 4.97E-02 |
| 5265 | SLC34A3 | -0.10 | 4.97E-02 |
| 5266 | C16orf82 | 0.16 | 4.97E-02 |
| 5267 | BAG4 | -0.14 | 4.97E-02 |
| 5268 | HSP90AA6P | 0.43 | 4.98E-02 |
| 5269 | MACF1 | -0.24 | 4.98E-02 |
| 5270 | RCOR3 | -0.23 | 4.98E-02 |
| 5271 | DNAJB6 | -0.35 | 4.98E-02 |
| 5272 | GPLD1 | -0.48 | 4.98E-02 |
| 5273 | LOC731725///LOC644035 | -0.10 | 4.98E-02 |
| 5274 | SH3TC1 | -0.18 | 4.98E-02 |
| 5275 | CCPG1 | -0.17 | 4.98E-02 |
| 5276 | KRTAP11-1 | 0.13 | 4.98E-02 |
| 5277 | RPS6KA3 | -0.27 | 4.98E-02 |
| 5278 | MKRN3 | -0.21 | 4.98E-02 |
| 5279 | OR2L2 | 0.16 | 4.98E-02 |
| 5280 | SLC16A6 | -0.22 | 4.98E-02 |
| 5281 | LMAN1L | 0.25 | 4.98E-02 |
| 5282 | ADNP2 | -0.14 | 4.98E-02 |
| 5283 | APCS | 0.17 | 4.99E-02 |
| 5284 | C1QL2 | 0.27 | 4.99E-02 |
| 5285 | PSG8 | 0.16 | 4.99E-02 |
| 5286 | CRKRS | 0.38 | 5.00E-02 |
| 5287 | ZBTB20 | -0.20 | 5.00E-02 |
| 5288 | LOC728102 | 0.13 | 5.00E-02 |
| 5289 | COL6A2 | -0.22 | 5.00E-02 |
| 5290 | FAM79B | -0.14 | 5.00E-02 |
| 5291 | PVRL1 | -0.17 | 5.00E-02 |
