# Supplementary material for: Novel metastatic models of esophageal adenocarcinoma derived from FLO-1 cells highlight the importance of E-cadherin in cancer metastasis
Source: Oncotarget. 2016 Nov 16;7(50):83342–58. doi: 10.18632/oncotarget.13391 (PMC5347774; doi:10.18632/oncotarget.13391)
Supplement: Supplementary file 6 [file oncotarget-07-83342-s006.docx]

**Supplementary Table S6.** Overlapping GO processes enriched in FLO-1^LM^ and *CDH1* low tumors

| **Enriched overlapping GO processes** | **FLO-1^LM^ vs. FLO-1^Par^**  **RNAseq FDR p-value** | ***CDH1* Low vs. High**  **GSE19417 FDR p-value** |
| --- | --- | --- |
| single-organism cellular process | 7.82E-10 | 2.83E-52 |
| response to organic substance | 7.62E-09 | 3.12E-35 |
| response to chemical | 1.93E-08 | 6.75E-28 |
| multicellular organism development | 2.88E-08 | 6.03E-44 |
| regulation of cell migration | 2.88E-08 | 8.55E-13 |
| response to stress | 3.57E-08 | 4.33E-31 |
| localization | 3.87E-08 | 4.34E-52 |
| system development | 3.87E-08 | 8.45E-45 |
| cell differentiation | 3.87E-08 | 6.66E-36 |
| immune system process | 3.87E-08 | 1.99E-18 |
| regulation of locomotion | 3.87E-08 | 1.03E-17 |
| regulation of cellular component movement | 3.87E-08 | 2.90E-16 |
| regulation of cell motility | 5.50E-08 | 8.19E-15 |
| cellular developmental process | 6.93E-08 | 1.07E-32 |
| developmental process | 1.15E-07 | 2.16E-39 |
| embryo development | 1.24E-07 | 2.24E-09 |
| cellular response to chemical stimulus | 1.45E-07 | 2.22E-26 |
| regulation of localization | 1.90E-07 | 1.07E-29 |
| anatomical structure development | 2.02E-07 | 1.05E-39 |
| single-organism developmental process | 2.02E-07 | 8.48E-39 |
| regulation of cellular component organization | 2.02E-07 | 1.12E-19 |
| anatomical structure morphogenesis | 2.10E-07 | 9.23E-28 |
| response to external stimulus | 2.70E-07 | 1.52E-24 |
| response to lipid | 3.06E-07 | 2.25E-14 |
| response to oxygen-containing compound | 3.08E-07 | 9.99E-19 |
| single-multicellular organism process | 3.09E-07 | 1.25E-52 |
| response to organic cyclic compound | 3.26E-07 | 1.50E-21 |
| animal organ development | 4.67E-07 | 3.72E-35 |
| cell migration | 4.93E-07 | 1.35E-18 |
| response to inorganic substance | 5.38E-07 | 4.34E-09 |
| response to endogenous stimulus | 5.59E-07 | 6.34E-25 |
| cellular response to organic substance | 6.45E-07 | 4.57E-23 |
| negative regulation of biological process | 7.54E-07 | 6.38E-24 |
| cellular process | 1.31E-06 | 3.59E-42 |
| single-organism process | 1.35E-06 | 1.59E-58 |
| cell motility | 1.59E-06 | 1.85E-19 |
| localization of cell | 1.59E-06 | 1.85E-19 |
| cellular component organization | 1.94E-06 | 3.38E-29 |
| negative regulation of cellular process | 1.94E-06 | 4.01E-23 |
| response to stimulus | 2.41E-06 | 9.49E-31 |
| cellular response to cytokine stimulus | 2.41E-06 | 5.05E-11 |
| cellular component organization or biogenesis | 3.18E-06 | 6.08E-27 |
| blood vessel morphogenesis | 4.45E-06 | 9.24E-16 |
| response to steroid hormone | 5.04E-06 | 1.36E-11 |
| locomotion | 6.42E-06 | 2.18E-30 |
| response to abiotic stimulus | 7.11E-06 | 1.33E-21 |
| regulation of biological quality | 7.90E-06 | 2.15E-57 |
| cellular localization | 9.77E-06 | 7.70E-22 |
| cellular component morphogenesis | 9.77E-06 | 2.72E-14 |
| immune response | 9.77E-06 | 2.23E-09 |
| multi-organism process | 1.02E-05 | 4.99E-22 |
| hematopoietic or lymphoid organ development | 1.38E-05 | 1.21E-07 |
| small molecule metabolic process | 1.45E-05 | 2.30E-16 |
| cell morphogenesis involved in differentiation | 1.52E-05 | 9.08E-19 |
| type I interferon signaling pathway | 1.60E-05 | 5.06E-07 |
| response to alcohol | 1.63E-05 | 2.67E-14 |
| movement of cell or subcellular component | 1.93E-05 | 1.50E-26 |
| cell development | 2.07E-05 | 8.75E-30 |
| cytokine-mediated signaling pathway | 2.13E-05 | 1.33E-07 |
| nervous system development | 2.29E-05 | 2.70E-36 |
| blood vessel development | 2.29E-05 | 3.50E-15 |
| response to type I interferon | 2.43E-05 | 1.46E-07 |
| hemopoiesis | 2.79E-05 | 7.40E-08 |
| cell morphogenesis | 3.02E-05 | 2.54E-15 |
| immune system development | 3.29E-05 | 4.85E-08 |
| regulation of cell adhesion | 3.51E-05 | 1.57E-08 |
| neurogenesis | 3.61E-05 | 5.63E-31 |
| vasculature development | 5.60E-05 | 2.54E-15 |
| response to cytokine | 5.60E-05 | 8.02E-11 |
| regulation of anatomical structure size | 5.86E-05 | 1.60E-08 |
| anatomical structure formation involved in morphogenesis | 6.81E-05 | 9.45E-17 |
| positive regulation of cellular component organization | 7.00E-05 | 8.31E-11 |
| cellular protein localization | 7.54E-05 | 1.33E-17 |
| cellular macromolecule localization | 8.64E-05 | 1.45E-17 |
| organonitrogen compound metabolic process | 9.84E-05 | 2.75E-09 |
| single-organism cellular localization | 9.99E-05 | 7.81E-15 |
| establishment of localization | 1.05E-04 | 6.03E-40 |
| innate immune response | 1.05E-04 | 1.14E-09 |
| cell death | 1.05E-04 | 1.64E-09 |
| positive regulation of locomotion | 1.28E-04 | 1.82E-10 |
| actin filament-based process | 1.34E-04 | 8.23E-08 |
| negative regulation of cell proliferation | 1.39E-04 | 2.46E-09 |
| negative regulation of locomotion | 1.41E-04 | 1.41E-13 |
| response to metal ion | 1.41E-04 | 1.68E-08 |
| cellular response to endogenous stimulus | 1.50E-04 | 6.84E-13 |
| positive regulation of cell migration | 1.53E-04 | 1.12E-09 |
| regulation of anatomical structure morphogenesis | 1.93E-04 | 1.58E-17 |
| vesicle-mediated transport | 1.93E-04 | 6.41E-12 |
| response to hormone | 1.97E-04 | 6.65E-19 |
| positive regulation of cellular process | 2.21E-04 | 1.55E-32 |
| angiogenesis | 2.21E-04 | 9.99E-13 |
| positive regulation of cell motility | 2.21E-04 | 3.62E-10 |
| programmed cell death | 2.21E-04 | 2.18E-08 |
| membrane organization | 2.24E-04 | 1.34E-13 |
| cell surface receptor signaling pathway | 2.37E-04 | 9.69E-31 |
| protein localization | 2.52E-04 | 1.62E-22 |
| response to oxygen levels | 2.52E-04 | 4.91E-07 |
| regulation of intracellular transport | 2.70E-04 | 3.89E-08 |
| regulation of cell morphogenesis | 2.76E-04 | 1.67E-09 |
| positive regulation of cellular component movement | 2.77E-04 | 5.08E-10 |
| phosphate-containing compound metabolic process | 2.87E-04 | 4.62E-12 |
| regulation of cellular localization | 3.02E-04 | 2.07E-15 |
| defense response | 3.25E-04 | 2.59E-13 |
| generation of neurons | 3.41E-04 | 3.62E-30 |
| macromolecule localization | 3.44E-04 | 2.70E-23 |
| apoptotic process | 3.46E-04 | 4.20E-08 |
| protein localization to membrane | 3.61E-04 | 1.27E-14 |
| interspecies interaction between organisms | 3.66E-04 | 6.31E-15 |
| symbiosis, encompassing mutualism through parasitism | 3.66E-04 | 6.31E-15 |
| striated muscle contraction | 4.05E-04 | 4.90E-09 |
| circulatory system process | 4.05E-04 | 1.48E-15 |
| establishment of protein localization to organelle | 4.37E-04 | 2.11E-11 |
| cellular component assembly | 4.58E-04 | 1.83E-07 |
| negative regulation of cell death | 4.61E-04 | 1.50E-09 |
| negative regulation of cellular component organization | 4.61E-04 | 1.60E-08 |
| regulation of developmental process | 5.10E-04 | 1.49E-31 |
| negative regulation of cell migration | 5.37E-04 | 5.13E-08 |
| phosphorus metabolic process | 5.56E-04 | 4.62E-12 |
| regulation of multicellular organismal process | 5.80E-04 | 6.03E-44 |
| negative regulation of cellular component movement | 5.95E-04 | 1.17E-11 |
| cardiovascular system development | 5.97E-04 | 1.06E-16 |
| circulatory system development | 5.97E-04 | 1.06E-16 |
| response to nitrogen compound | 6.28E-04 | 5.75E-13 |
| negative regulation of programmed cell death | 6.78E-04 | 3.64E-07 |
| single-organism localization | 6.96E-04 | 2.06E-41 |
| biological adhesion | 7.57E-04 | 4.81E-20 |
| response to hypoxia | 7.59E-04 | 3.19E-09 |
| negative regulation of cell motility | 7.59E-04 | 4.68E-09 |
| epithelium development | 8.01E-04 | 8.67E-14 |
| establishment of protein localization | 8.04E-04 | 2.16E-17 |
| response to organonitrogen compound | 8.04E-04 | 2.07E-12 |
| regulation of molecular function | 8.15E-04 | 3.48E-20 |
